# Elevated Extinction Rates as a Trigger for Diversification Rate Shifts: Early Amniotes as a Case Study

**DOI:** 10.1038/srep17104

**Published:** 2015-11-23

**Authors:** Neil Brocklehurst, Marcello Ruta, Johannes Müller, Jörg Fröbisch

**Affiliations:** 1 Museum für Naturkunde, Leibniz-Institut für Evolutions- und Biodiversitätsforschung, Invalidenstraße 43, D-10115 Berlin, Germany; 2 Institut für Biologie, Humboldt-Universität zu Berlin, Invalidenstraße 110, Berlin D-10115, Germany; 3 School of Life Sciences, University of Lincoln, Joseph Banks Laboratories, Green Lane, Lincoln LN6 7DL, UK; 4 Berlin-Brandenburg Institute of Advanced Biodiversity Research, Altensteinstrasse 6, Berlin D-14195, Germany

## Abstract

Tree shape analyses are frequently used to infer the location of shifts in diversification rate within the Tree of Life. Many studies have supported a causal relationship between shifts and temporally coincident events such as the evolution of “key innovations”. However, the evidence for such relationships is circumstantial. We investigated patterns of diversification during the early evolution of Amniota from the Carboniferous to the Triassic, subjecting a new supertree to analyses of tree balance in order to infer the timing and location of diversification shifts. We investigated how uneven origination and extinction rates drive diversification shifts, and use two case studies (herbivory and an aquatic lifestyle) to examine whether shifts tend to be contemporaneous with evolutionary novelties. Shifts within amniotes tend to occur during periods of elevated extinction, with mass extinctions coinciding with numerous and larger shifts. Diversification shifts occurring in clades that possess evolutionary innovations do not coincide temporally with the appearance of those innovations, but are instead deferred to periods of high extinction rate. We suggest such innovations did not cause increases in the rate of cladogenesis, but allowed clades to survive extinction events. We highlight the importance of examining general patterns of diversification before interpreting specific shifts.

The shape of evolutionary trees provides unique insights into models of group origination and extinction, the impact of structural, functional and ecological innovations on diversification, and the dynamics of clade turnover. Analyses of significant shifts in diversification rates across phylogenies are central to macroevolutionary studies, as they cast direct light on patterns of lineage appearance and disappearance, which ultimately shaped the Tree of Life. When asymmetric phylogenetic branching is associated with a significant increase in diversification rate, it becomes axiomatic to ask whether such a shift is underpinned by a key evolutionary novelty, and whether the novelty in question can explain the success of a clade. Numerous methods are now available for detecting unevenness in diversification rates, in terms of both their chronology and their position in a phylogeny^1^,^2^,^3^,^4^,^5^,^6^,^7^,^8^. One category of methods seeks to locate diversification rate shifts using exclusively information on tree topology rather than character or branch length data^2^,^3^,^4^. Shifts are significant departures from a null model of clade growth, such that evolutionary lineages are more diverse than expected. For example, in a null equal-rates Markov model^4^,^9^, the speciation rate is constant over time and each taxon has equal probability of splitting.

Although primarily designed to assess variation in diversification rates, analyses of shifts have also been used to link exceptional diversifications to extrinsic (physical) and/or intrinsic (biological) factors. For instance, a shift may coincide temporally with a climatic or environmental change^10^,^11^,^12^, may occur in the aftermath of a large-scale crisis (e.g. a mass extinction^13^), or may be associated with the origin of a key evolutionary innovation^14^,^15^,^16^,^17^,^18^,^19^,^20^. Causal connections between diversification shifts and innovations are at the core of Simpsonian adaptive radiation models^21^, whereby key innovations either provide a group with a selective advantage or allow it to access new ecological niches. This in turn may lead to a remarkable increase in diversification rates. However, inferring a causal link between a rate increase and a key evolutionary novelty may be problematic, as such inferences are often circumstantial, rely solely on the temporal (or otherwise) coincidence of rate and novelty, and focus on limited temporal scales and/or taxonomic groups, and therefore preclude inference on general patterns of diversification in large clade over long time intervals. These causal inferences also make an implicit assumption that a diversification shift results from an increase in rate of cladogenesis, i.e. the proliferation of evolutionary lineages. However, diversification is a function of both origination and extinction^22^. As a result, a rate shift in a clade could imply either an increase in its origination rate and/or an increase in the extinction rate of its sister clade^23^.

The present study focuses on the early evolution of the crown-group Amniota, the clade encompassing the latest common ancestor of Synapsida (“mammal lineage”) and Sauropsida (“reptile-bird lineage”)^24^ and all its descendants. As a speciose and successful vertebrate radiation, amniotes are an excellent group for inferring general patterns of diversification over an extensive time period that includes some of the most profound environmental and climatic changes on a global scale. Amniotes first appeared in the fossil record in the Late Carboniferous^24^,^25^ and include more than one-third of extant vertebrate diversity. During the Carboniferous, Permian, and Triassic, they occupied a wide range of niches and evolved a large variety of ecological adaptations, including herbivory^26^, fossoriality^27^,^28^, arboreality^29^,^30^, and secondarily aquatic lifestyles^31^,^32^. Amniotes went through multiple radiations and extinctions, including the most catastrophic of all biological crises in Earth’s history at the Permo-Triassic boundary^33^,^34^,^35^.

This study presents a new supertree of Carboniferous–Triassic crown-group Amniota plus its sister clade, the Diadectomorpha^36^ (Fig. 1). Our supertree maximizes the taxonomic scope and sample size of our investigation, and is used to address three major questions: (1) Which portions of the Palaeozoic and early Mesozoic amniote tree underwent significant shifts in diversification rate? (2) Did shifts coincide temporally with the acquisition of morphological innovations? (3) What is the influence of uneven extinction rates on diversification rates? In order to address these questions, the supertree was subjected to analyses of topology-dependent shifts using SymmeTREE^4^,^37^,^38^. This program compares the observed phylogeny with phylogenies simulated under both equal and heterogeneous diversification rate models, in order to assess which groups have experiences a significant increase in diversification rate relative to their sister taxa. The Δ_2_ shift statistic calculated by SymmeTREE for each tree node^4^,^37^ represents the magnitude of the diversification rate shifts at the relevant node, and its associated p-value measures the statistical significance of the shift (i.e., the probability that a shift of a given magnitude could have occurred at random). Species richness, origination rates, and extinction rates were calculated from the supertree after incorporating correction for heterogeneous sampling, and compared to the timing of both shifts and key innovations.

## Results and Discussion

### Extinction and origination rates compared to diversification statistics

Analyses of the correlation between the Δ_2_ values over time and the rates of origination and extinction using Generalised Least Squares (GLS) regression produced a surprising result. Based on the Akaike Weights criterion for model selection (Table 1), extinction is found to provide the best fit for the Δ_2_ values of amniotes, substantially better than a multivariate model that incorporates both origination and extinction. This suggests that many of the largest diversification rate shifts tend to occur preferentially during periods of elevated extinction rates, rather than periods of high origination rates.

The diversification rate shifts identified by SymmeTREE represent areas of tree imbalance. If multiple large diversification rate shifts (represented by peaks in Δ_2_) coincide temporally with peaks in extinction rate, this would imply that the extinction events are spread unevenly over the tree, producing the imbalance. We propose that elevated extinction levels experienced by certain lineages relative to their sister groups may have acted as a trigger for such large shifts. The fact that the Δ_2_ curve fits the origination rate curve less well indicates that peaks in origination rate do not tend to correspond to periods of great tree imbalance. Overall, increases in origination rate seem to be more evenly spread over the tree than increases in extinction rate. Thus, increased cladogenesis may not have been the sole or main driving force behind the diversification rate shifts. In early amniotes at least, uneven extinction rates may have exerted a stronger influence on clade dynamics by ‘facilitating’ high levels of lineage splitting.

In addition to GLS regression, we applied a Random Forest (RF) regression model to our data, and found similar results. Specifically, the variable with the highest “importance”, or highest percentage mean square error (%MSE) value, is extinction rate (Table 1). In fact, origination rate has a negative %MSE score, indicating that a random model better explains the Δ_2_ curve than the curve of origination rate. Because the percentage of the Δ_2_ variance explained by the variables tested is not particularly high (52%), there are clearly other variables impacting on the Δ_2_ signal that have not been tested. This is also supported by the GLS model; the akaike weight score of the extinction model, while high, is not overwhelming. Nevertheless, extinction rate is the most important variable among those we tested, suggesting that several large diversification rate shifts tend to occur during periods of high extinction rate, more so than during periods of high origination rate. We therefore propose that, in the case of early amniotes, uneven extinction primarily drives diversification rate shifts, with episodes of cladogenesis being evenly spread across the tree.

Further evidence for the proposed scenario can be gleaned from inspection of plots of mean Δ_2_ through time (Figs 2 and 3). Peaks in mean Δ_2_, representing both the magnitude and the number of shifts, often coincide with, or follow immediately after, large drops in diversity and peaks in extinction rate, the latter representing mass extinctions. Among synapsids (Figs 2A and 3A), the two largest peaks in mean Δ_2_ values occur immediately after two mass extinctions: Olson’s Extinction in the Kungurian and Roadian^34^,^39^ and the end-Permian event^33^,^34^,^35^. Olson’s Extinction included the decline of pelycosaurian-grade synapsids, and was followed immediately by the radiation of therapsids. The calculated extinction rates are consistent with the hypothesis that Olson’s Extinction impacted severely on synapsids (Fig. 3A). Immediately after Olson’s Extinction, a diversification shift occurred at the node subtending therapsids. This shift resulted both from the massive increase in therapsid diversity after Olson’s Extinction, and from a high extinction rate among pelycosaurian outgroups.

Of all extinctions, the end-Permian event had the most severe effects on early synapsids. The two curves of extinction rates and phylogenetic diversity estimate (PDE; a diversity measure that factors in the extent of ghost lineages and range extensions estimated from a phylogeny) indicate a two-phase event, with peaks in synapsid extinction rate at the end of the Changhsingian and at the end of the Induan (Figs 2A and 3A). However, the end-Permian event coincides with the largest peak in synapsid Δ_2_ and a diversification rate shift in the Lystrosaurinae. A further peak in Δ_2_ occurs in the Norian, during another period of high synapsid extinction rate and coinciding with a diversification shift in the clade containing Mammaliaformes and Trithelodontidae.

In parareptiles, an early Artinskian peak in mean Δ_2_ coincides with the sudden appearance of multiple lineages in the fossil record over a short time interval (Figs 2B and 3B), as attested by a diversification rate shift within the Ankyramorpha at this time. However, this also coincides with a peak in extinction rate, implying the shift was driven by the interplay of increased origination and extinction rates. The next two largest peaks in mean parareptile Δ_2_ values followed the two largest Permian peaks in parareptile extinction rate (Fig. 3B), one at the end of the Wordian and one at the very end of the Changsingian. The extinction peak at the end of the Wordian is associated with the disappearance of bolosaurids and the decline of nycteroleterids, resulting in a substantial shift in diversification rate within the clade containing Pareiasauridae and Procolophonoidea. Meanwhile, during the end-Permian mass extinction Pareiasauridae, Nycteroleteridae, and Millerettidae died out, and a diversification shift occurred within the procolophonid clade containing Leptopleuroninae and Procolophoninae. As in the case of synapsids, shifts in diversification rate among parareptiles were driven not only by increases in cladogenesis, but also by uneven extinction rates among clades.

The largest peak in extinction rate among eureptiles occurred during Olson’s Extinction, and exceeded the rate observed during the end-Permian mass extinction. At this time, Captorhinidae, the most diverse sauropsid family of their time, suffered a conspicuous decline in diversity. A second peak in extinction rate at the end of the Permian was again followed by a peak in mean Δ_2_ (Fig. 3C) and by a shift in diversification rates in Sauria (the clade of Lepidosauriformes plus Archosauriformes). This shift was underpinned by the extinction of the more basal sauropsid clades such as captorhinids and basal diapsids e.g. Younginiformes, but also cladogenesis within saurians. In the immediate aftermath of the end-Permian event, Lepidosauromorpha^40^, Ichthyopterygia, Sauropterygia^41^,^42^ and Archosauromorpha^43^,^44^,^45^ appear and diversify. This provides a further example of a selective extinction followed by increased cladogenesis among the survivors. The biggest peak in eureptile Δ_2_ values occur during the Anisian, where diversification rate shifts are identified within the Sauropterygia and the ichthyosaur clade the Henosauria, as well as multiple archosauromorph clades. As well as a period of radiation for these clades, this is also a time of elevated extinction rates. In fact, extinction rate and Δ_2_ values remain high for most of the middle and late Triassic, indicating the continuous influence of uneven extinction creating further tree imbalance among archosaurs. The fact that the number and magnitude of substantial shifts increased during times of elevated extinction in all three amniote subclades indicates that extinction selectivity may have been as important as uneven origination rates in producing tree imbalance.

### Key innovations among amniotes

There are several examples of shifts coinciding with the emergence of key ecological and functional innovations, but their status as “adaptive radiations” are called into question based on the above analyses. For instance, the highly significant diversification shift observed in therapsids coincided with several physiological and morphological innovations, which allowed for more effective food processing, ventilation, and environmental tolerance^46^,^47^. Diversification shifts within Kannemeyeriiformes coincided with the evolution of large body size, and a shift at the very base of Amniota may have been related to the appearance of the amniotic egg (although this shift at the very base of the tree should be interpreted with care; the absence of further outgroups makes it difficult to ascertain the precise location of the shift^37^). There are further examples of diversification shifts coinciding with evolutionary novelties. We examine in detail two case studies: a secondary return to an aquatic lifestyle and the acquisition of herbivory. A secondary return to an aquatic lifestyle coincided with diversification shifts at the base of Sauropterygia and Phytosauria. Cranial and mandibular re-modelling accompanying increased specializations towards herbivory are marked by shifts in distinct groups, such as dicynodonts (a keratinous beak and propalinal lower jaw movements)^48^, Triassic procolophonids (chisel-shaped teeth for processing tough vegetation)^48^, and plateosaurian sauropodomorph dinosaurs (increase in body size and high browsing)^49^. However tempting it may be to view these shifts as adaptive radiations (the evolutionary novelty provides a selective advantage and access to a new niche, resulting in increased rate of cladogenesis), one should exert caution in drawing inference. The results discussed so far cast doubt on the hypothesis of a causal relation, given that increased cladogenesis was not the driving force behind diversification rate shifts.

Although arthropod herbivores were present in the terrestrial realm before the appearance of amniotes, the vast majority of primary consumers in Carboniferous and earliest Permian terrestrial ecosystems were arthropod detritivores^50^. Those amniotes that first adopted the high-fibre herbivorous diet were therefore entering a taxonomically depauperate region of ecospace. However, there is no evidence for an adaptive radiation in these earliest herbivores, such as *Edaphosaurus*, Caseidae, and Diadectidae. We could not identify diversification rate shifts in these clades; instead, shifts occurred in later Permian and Triassic herbivore specialists, such as dicynodonts, plateosaurians and procolophonids. The origination rates of herbivores were not consistently higher than those of other taxa during the Carboniferous and earliest Permian (Fig. 4A), providing further evidence against an adaptive radiation of herbivores at this time. Diversification shifts are observed in dicynodonts and Triassic procolophonids during the Middle and Late Permian and accorss the Permian-Triassic boundary, a time of consistently high extinction rates among amniotes (Fig. 3). The timing of the shifts is interesting. Despite the fact that dicynodonts and their evolutionary innovations first appeared in the Wordian, the shift in their diversification rate occurs in the late Capitanian. The first members of Plateosauria appeared in the Carnian, but did not experience a shift in diversification rate until the Norian. Both the late Capitanian and the Norian are characterised by high extinction rates (Fig. 3). However, the appearance of “key innovations” in these herbivore specialists did not appear to be associated with a major diversification, but rather with high extinction rate, as shown above. We propose a mechanism whereby key innovations have the potential to buffer against extinction. The selective extinction of groups without the innovation, and the subsequent radiation of survivors, appear to be at the root of the observed patterns.

Unlike the example of herbivores, amniotes that evolved a secondarily aquatic lifestyle did not colonise under-filled ecospace. Aquatic or semi-aquatic taxa, such as mesosaurid parareptiles and various Late Permian diapsids, probably faced competition from other medium and large-sized vertebrates (e.g. amphibians; fish). Interestingly, those secondarily aquatic taxa showed low origination rates during the Carboniferous and Early Permian (Fig. 4B), and no diversification rate shifts occurred during this time, again providing evidence against the adaptive radiation scenario. It was not until the earliest Triassic that origination rates in those aquatic taxa exceeded those in other groups. Furthermore, it was also not until the Triassic that diversification rate increases coinciding with the evolution of an aquatic bauplan could be detected, specifically at the base of Sauropterygia and Phytosauria.

One could again infer the influence of extinction on these diversification shifts. The shift observed in sauropterygians did not coincide with their origin or with the increase in origination rate of aquatic amniotes during the earliest Triassic (Fig. 4B); instead it occurred during the Anisian, a time when amniote extinction rates rose preceding the Ladinian diversity decrease. Also at this time, reduced diversity of potential competitors such as fish and marine trematosaurid temnospondyls is observed^51^,^52^. The shift observed in phytosaurs occurred at the end of the Carnian, post-dating their first appearance, but coinciding with a period of high extinction rate and declining diversity not only amongst amniotes (Figs 2 and 3) but also among temnospondyl amphibians^52^. Once again, we do not see an adaptive radiation of phytosaurs and sauropterygians coinciding with the first appearance of their novel bauplan, but instead the diversification shift is deferred to a period of high extinction rate amongst other, and possibly competing, groups. One might argue that observed increase in diversification rate coinciding with the adoption of an aquatic bauplan are in part influenced by increases in preservation potential in aquatic environments. However, the lack of increases in diversification rate of Permian aquatic amniotes relative to their terrestrial relatives would argue against this; there is no reason to suggest that preservation potential in Mesozoic aquatic environments was substantially better than in Paleozoic aquatic environments

Simultaneously co-occurring diversification shifts and evolutionary novelties have been seen as being causally linked^14^,^15^,^16^,^17^,^18^,^19^,^20^. However such an interpretation is risky; studies that have posited such links often focus on a limited temporal range and taxonomic sample, and do not investigate other possible patterns and correlations. Our broader scale analysis indicates that the patterns of radiation within early amniotes are heavily connected to the extinction events occurring during this time. The strong correlation of extinction rate and the number of substantial diversification shifts illustrates that uneven extinction rates within amniotes have had just as significant effect on tree topology as the pattern of origination. Some previous studies have suggested that shifts co-occur with extinction events^13^ or other extrinsic factors such as climate or geographic changes^10^,^11^,^12^. However, the present study suggests that, at least among early amniotes, there is a more complex relationship between diversification and evolutionary novelties. Extinction selectivity based on morphology and ecology has been documented in a number of clades^6^,^53^,^54^. In early amniotes, the appearance of evolutionary novelties, such as herbivory and an aquatic body plan, and often the expansion into almost unoccupied ecospace (as in the earliest herbivores) did not on its own cause significant shifts in diversification rate. Instead, it appears that diversification shifts were underpinned by selective elimination of taxa and subsequent radiation of survivors, whereby taxa that had already acquired innovations enabling them to exploit new resources were buffered against extinction and subsequently experienced a ‘deferred’ diversification. We propose the phylogeny of early amniotes was shaped by the interaction between evolutionary innovation and extinction.

## Material and Methods

### Supertree assembly

**Figure 1:**
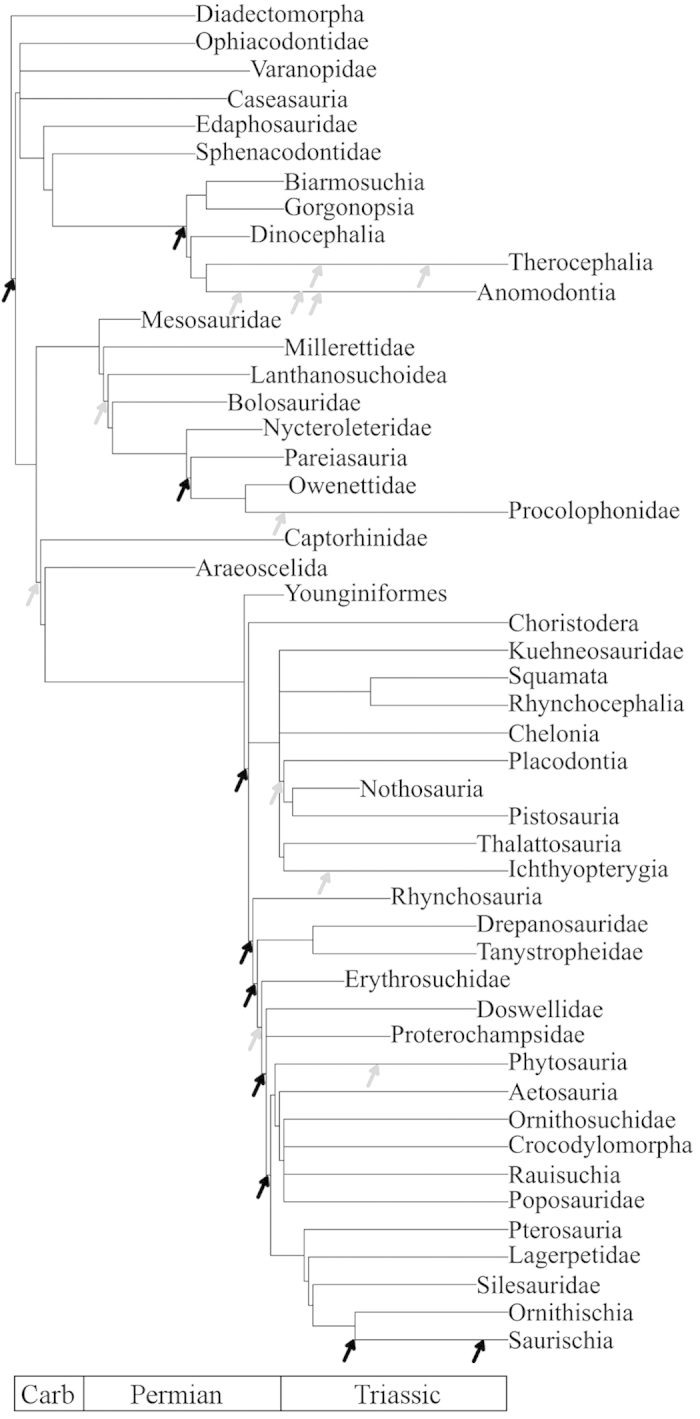
A summary version of the strict consensus supertree (see Supplementary Data 3 for the full tree). Grey arrows indicate substantial diversification shifts; black arrows significant shifts. Shifts occurring along branches are placed to represent the time of the shift (see Supplementary Data 7 for the precise time and location of the shifts).

**Figure 2:**
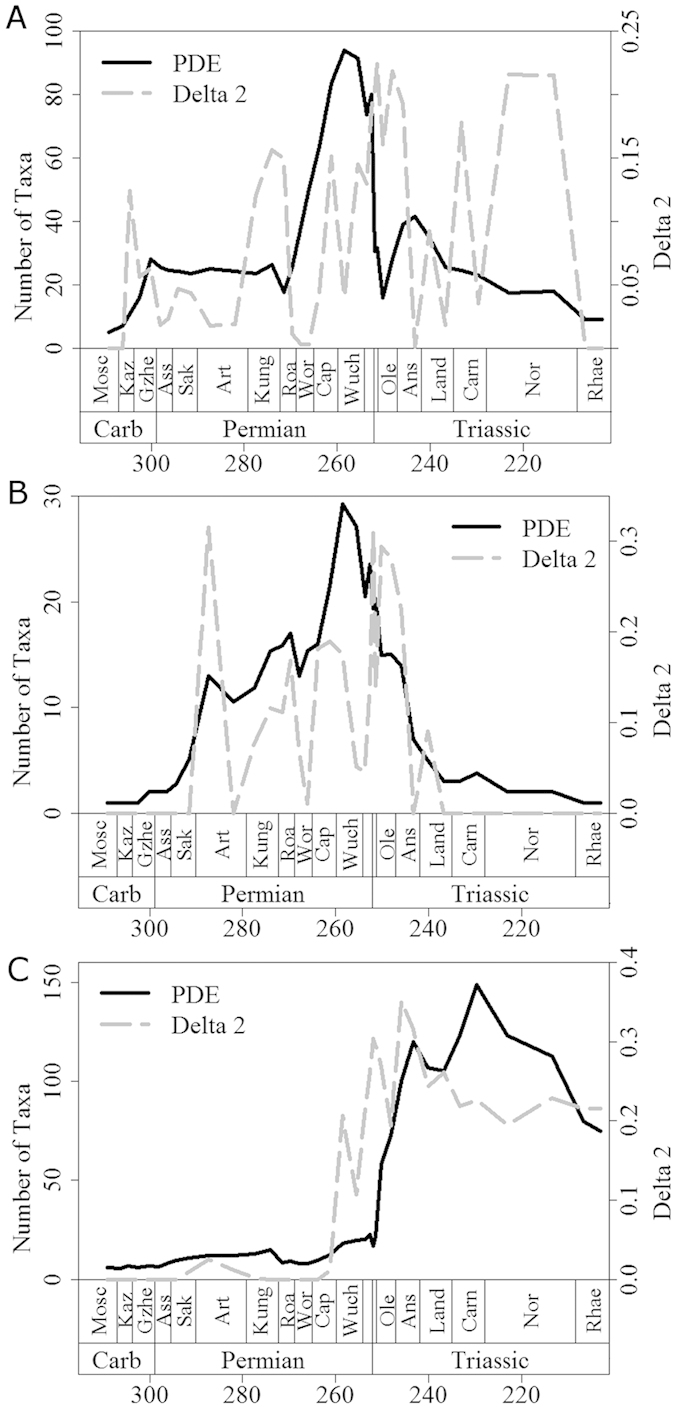
A comparison of the phylogenetic diversity estimate (black solid line) and mean Δ2 values (grey dashed) for (a) Synapsida; (b) Parareptilia and (c) Eureptilia.

**Figure 3:**
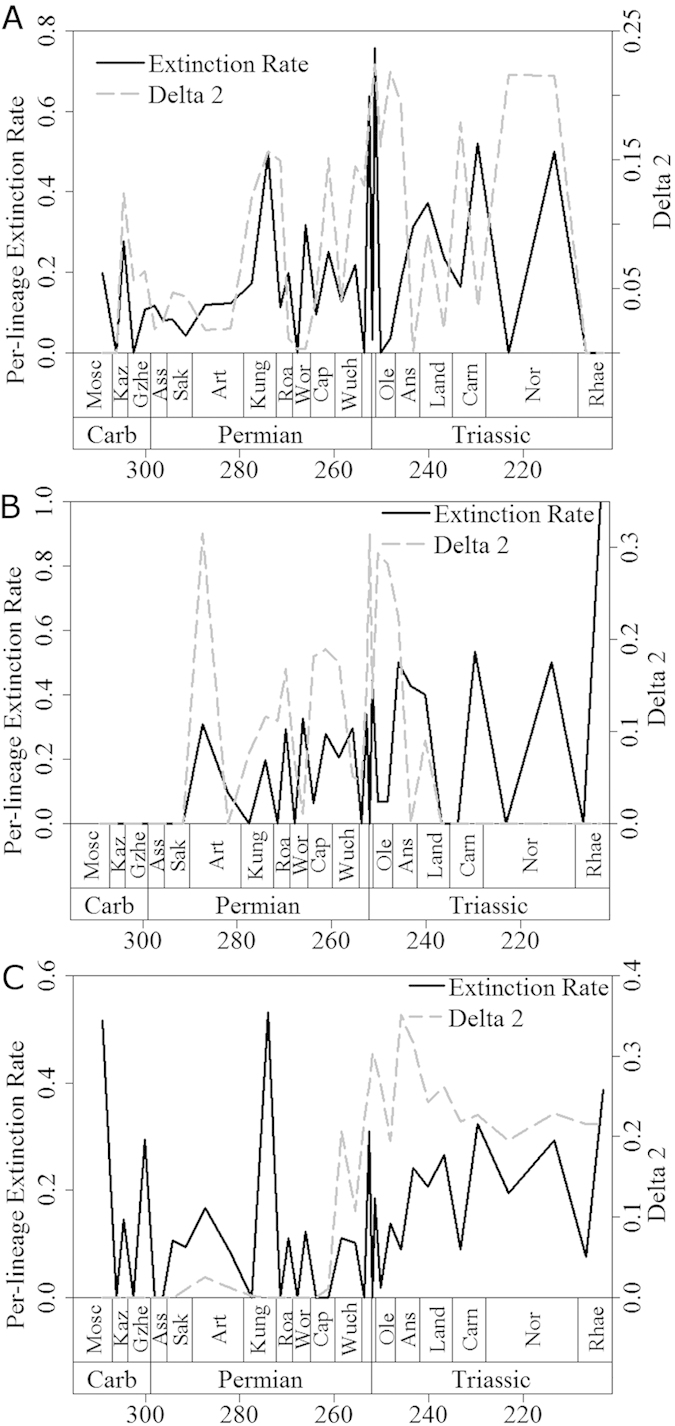
A comparison of per-lineage extinction rate (black solid) and mean Δ2 values (grey dashed) for (a) Synapsida; (b) Parareptilia and (c) Eureptilia.

**Figure 4:**
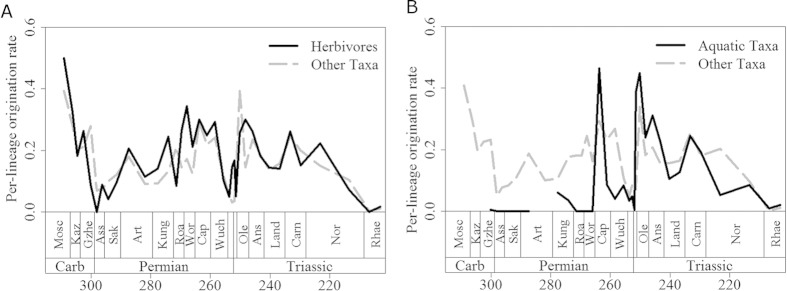
A comparison of mean per-lineage origination rates of (a) herbivorous, and (b) aquatic lineages with other lineages.

The supertree was built from 177 published phylogenies, using “matrix representation with parsimony”^55^,^56^. After removing poorly resolved taxa and collapsing all nodes containing no taxa from the time period under study (Carboniferous–Triassic), such that a ghost lineage was retained in that period, a tree with 686 terminals was obtained. This was time-calibrated according to the method of Brusatte *et al.*^57^ (summary presented in Fig. 1, full tree in Supplementary Data 3). Support for each node was calculated using the V index^58^ (see Supplementary Methods for more details of supertree construction, time calibration, list of input trees and measures of node support).

### Diversification shift analyses

SymmeTREE v. 1.1^4^,^38^ implements tree topology-based methods for detecting diversification shifts. The program calculates two statistics, Δ_1_ and Δ_2_, which assess differences in diversity and topology between the two branches subtended by each internal node. It then compares such differences to those obtained under an equal rate diversification model and a heterogeneous diversification model, and calculates the probability of the observed topology under each model. A shift is detected along the branch leading to the more speciose of the two descendant lineages of those nodes that do not fit the equal rates model^4^,^37^.

In order to resolve polytomies in the supertree, 10 randomly selected most parsimonious MRP trees were subjected to analysis of diversification shifts, with 10^6^ trees simulated under the equal rates model. The Δ_2_ statistic was used to infer diversification shifts following published recommendations^37^. We report both significant (*p* < 0.05) and substantial (0.05 < *p* < 0.1) shifts^13^. Due to the fact that each of the 10 MPTs would contain a different set of relationships, only those shifts present in all 10 trees are reported. A plot of mean Δ_2_ values through time illustrates temporal trends in magnitude and number of shifts.

SymmeTREE does not incorporate any temporal information into the analysis. However, the statistics employed assume that all descendants of the node under analysis have had equal time to diversify^13^. Ruta *et al.*^13^ suggested the use of time slicing: for each substage, the tree was pruned to include only those taxa present in the time slice, or with a ghost lineage extending into it. Each of these pruned trees was analysed in SymmeTREE separately. This method of time slicing was implemented using the *timeSliceTree* function of the paleotree^59^ package in R^60^. We used Gradstein *et al.*’s time scale^61^. International stages were split into two substages (early and late) with the temporal boundary placed at the mid-point of stage duration. It should be noted that an alternative method of time slicing has been suggested^62^. The impact of using this alternative, as well as justification for using the Ruta method, is discussed in the Supplementary Methods, where other sensitivity analyses are presented in order to ascertain the impact of uncertain taxon relationships.

### Diversity, origination rates and extinction rates

Amniote diversity (species richness) through time was estimated from the time-calibrated supertree. This phylogenetic diversity estimate (PDE) refines and augments taxic richness based solely on raw counts of taxa in each time bin. Such counts are likely to have been influenced by uneven sampling and rock availability, whilst PDE attempts to provide additions to observed diversity in the form of ghost lineages and range extensions, i.e. unsampled portions of the fossil record inferred from the tree^63^,^64^. The PDE was calculated for each of 100 randomly selected MPTs using the *phyloDiv* function in palaeotree, and a mean curve was calculated.

The supertree was also used to infer extinction and origination rates. Extinction rates were calculated by dividing the number of lineages terminating in a time bin by the total number of lineages (observed and inferred) present in that bin. Origination rates were calculated by dividing the total number of cladogenetic events in each time bin by the total number of lineages present in that bin. As rates are affected by sampling, it is difficult to establish if an origination event occurred in the time bin in which it was observed, or in an earlier time bin in which the taxon was not sampled. The same is true of extinction rates; the last appearance of a taxon in the fossil record is unlikely to represent the last individual to have lived^22^. Here, we provided a correction for bias using the proportion of ghost lineages relative to observed lineages as a measure of sampling in each time bin (see Supplementary Methods for full details). Again, a set of origination and extinction rates were calculated for each of the 100 MPTs and the mean curve was calculated.

Origination rates were used to evaluate the impact of evolutionary innovation on diversification. As case studies of possible instances of adaptive radiations, we examined two ecological traits, namely herbivory and a secondarily aquatic lifestyle. Two binary characters (one for each trait) were created, and taxa were coded for the absence (0) or presence (1) of the traits in question (see Supplementary Data 8). Both characters were optimised over the tree using maximum likelihood estimates of ancestral conditions of each trait for all tree nodes using the *ancestral.pml* function in the phangorn package^65^ in R. A set of per-lineage origination rates were assembled for all lineages descended from an herbivorous ancestor, and another for those descended from an aquatic ancestor.

Generalised least squares regression (GLS) was used to investigate the relationship between diversification rate shifts, on the one hand, and origination and extinction rates on the other. This method has an advantage over simple correlation tests, in that it allows multivariate models to be compared as well as single variables. Through GLS, we correlated the set of amniote Δ_2_ values through time to a null model (statistically random variation around a mean of 1), to origination and extinction rates, and to a multivariate model of both origination and extinction. They were also compared to a variable representing mass extinctions (those time bins in which a mass extinction is deemed to have occurred i.e., extinction rate is above 30%, receive a score of 1, others a score of 0), and to a multivariate model of mass extinction + origination, to account for the possibility of diversification rate shifts being driven primarily by increased origination rates, but exceptional extinction events providing different diversification dynamics.

Additionally, a Random Forest (RF) regression was used to compare the Δ_2_ curve to the three variables in order to measure absolute model fit. This approach establishes how much of the Δ_2_ variance may be explained by the four variables whilst correcting for overfitting of the models.

## Additional Information

**How to cite this article**: Brocklehurst, N. *et al.* Elevated Extinction Rates as a Trigger for Diversification Rate Shifts: Early Amniotes as a Case Study. *Sci. Rep.* **5**, 17104; doi: 10.1038/srep17104 (2015).

## Supplementary Material

Supplementary Information

Supplementary Data 7-10

## Figures and Tables

**Table 1:** Summary of time series regression models comparing origination and extinction rates to the Delta 2 curve.

Model	Generalised Least Squares Regression			Random Forest Regression(Variance explained: 52%)	
	LogLikelihood	AIC	AkaikeWeights	%MSE	Increase inNode Purity
Extinction	36.99345	−69.9869	0.662553746	38.88489	5566.1174
Origination	35.62917	−65.25833	0.062290971	−10.32131	6401.3968
Mass Extinction	34.98031	−63.96063	0.032556088	31.10235	774.6298
Origination + Extinction	35.69722	−63.39445	0.024529433	NA	NA
Origination + Mass Extinction	33.81104	−59.62208	0.003719877	NA	NA
Null	36.86496	−67.72992	0.214349884	NA	NA
