# Supplementary material for: Elevated Extinction Rates as a Trigger for Diversification Rate Shifts: Early Amniotes as a Case Study
Source: Sci Rep. 2015 Nov 23;5:17104. doi: 10.1038/srep17104 (PMC4655484; doi:10.1038/srep17104)
Supplement: Supplementary Information [file srep17104-s1.pdf]

Elevated Extinction Rates as a Trigger for Diversification Rate Shifts: Early Amniotes as a Case Study

Neil Brocklehurst[a], **Marcello Ruta**[c], **Johannes Müller**[a, b, d] **and Jörg Fröbisch**[a, b]

[a] *Museum für Naturkunde, Leibniz-Institut für Evolutions- und Biodiversitätsforschung, Invalidenstraße 43, D-10115 Berlin, Germany*

[b] *Institut für Biologie, Humboldt-Universität zu Berlin, Invalidenstraße 110, Berlin D-10115, Germany*

[c] *School of Life Sciences, University of Lincoln, Joseph Banks Laboratories, Green Lane, Lincoln LN6 7DL, UK*

[d] *Berlin-Brandenburg Institute of Advanced Biodiversity Research, Altensteinstrasse 6, Berlin D-14195, Germany*

Corresponding Author: Neil Brocklehurst, neil.brocklehurst@mfn-berlin.de, +493020938306

1. SUPPLEMENTARY METHODS

a) *Formation of the supertree*

A large number of methods exist for combining phylogenetic hypotheses into a supertree. “Matrix representation with parsimony” (MRP), developed independently by Baum[1] and Ragan[2] is the most frequently used and has been subjected to extensive analyses regarding its performance and biases[3-7]. This method treats each node in each source tree as a character. If a species is a descendant of a particular node in a particular source tree, it is scored as “1” for the character representing that node. If it is not a descendant of the node it is scored as “0”. Species not included in that source tree are scored as “?”. These “characters” are combined into a matrix which may be subjected to a parsimony analysis, rooted on an all-0 outgroup.

All published hypotheses of phylogenetic relationships created using computer algorithms (rather than manually generated) containing 3 or more amniote or diadectomorph taxa from the time period under study were considered as source trees for the analysis. In order that the supertree input data was “accountable” (a concern raised by Gatesy et al. (8)), published trees which did not include full details of their method e.g. did not include a character matrix, details of algorithms and outgroups used, were rejected.

In order to reduce instances of tree non-independence (another issue raised by Gatesy et al.[8]; including many trees based on the same character list would bias the supertree towards topologies based on those characters) the following procedure was followed (modified from[9]): (1) if one study uses a character list and a taxon list that is identical to, or a subset of, another analysis, then only the more inclusive study was included as a source tree; (2) if one study uses a character list which is identical to, or a subset of, another analysis, but the taxon lists are not identical nor is one a subset of the other, then a mini supertree (using the MRP method) was constructed of the two or more trees. This mini supertree was included as a source tree; (3) if a study uses different methods to analyse the same dataset e.g. analysing the

dataset using both parsimony and Bayesian methods, a mini supertree was constructed from the results of each analysis, and this mini supertree was included as a source tree.

After pruning the list of published phylogenetic analyses in this way, 177 source trees remained (see below). These trees then needed to be standardised with respect to the taxonomic level: different phylogenetic analyses study phylogeny at different levels: the family, genus or species level. This is a problem when combining trees, as the greatest resolution is wanted, but not all studies are undertaken at the species level. The following procedures were carried out to standardise the level of the source trees: (1) If a taxon is not studied at species level in any included analysis, then it is included at the genus or family level in all source trees e.g., *Sphenacodon* spp., Mesosauridae (2) If the paper specifies that their coding for a higher level taxon above the level of species is based primarily on a particular species, then that species is used to replace the higher-level taxon in the source tree. (3) If a taxon is included at the genus level or higher in one or more studies, and one or more different studies use more than one different species of that taxon, then all species included in a phylogenetic analysis are included as a polytomy replacing the higher-level taxon e.g. if Ophiacodontidae is used as a terminal taxon in a source tree, it is replaced in this analysis with a polytomy containing *Ophiacodon mirus*, *O.retroversus*, *Archaeothyris florensis Varanosaurus acutirostris* and *Stereophallodon ciscoesnsis*. As well as standardising the taxonomic level of the source trees, the taxonomy was updated; taxa now considered nomina dubia were removed.

Having been modified in this way, all source trees were incorporated into an MRP matrix using the program Supertree085b[10] (see Supplementary Data 1 for the data supplied to this program, including all source trees in phylip format). The Baum and Regan method was used, with all nodes and trees given equal weight. The MRP matrix (Supplementary Data 2) was subjected to parsimony analysis using the Willi Hennig Society edition of TNT[11], using the sectorial search, parsimony ratchet and drift algorithms. The minimum length was

searched for 100 times. However the MRP matrix of all 177 trees could not be analysed due to the poor overlap between many of the trees; more trees were produced in a single round of searches than could be stored in the memory of TNT. In order to deal with this problem, the list of source trees was divided into 8 categories, based on universal agreement of monophyly: Synapsida, Parareptilia, Archosauromorpha, Lepidosauriformes, Sauropterygia, Ichthyopterygia, and "Basal". The source trees were divided between these categories based on which clade they were representing the relationships of. Those in the "Basal" category include studies examining the relationships of multiple clades relative to each other and those including basal amniotes and basal eureptiles. An MRP matrix was produced for each category, and a supertree created for each clade, using the programs described above. Because of the uncertainty surrounding the position of turtles (either within parareptiles or lepidosauromophs), the categories Parareptilia, Sauropterygia and Basal were combined, and a single supertree of the taxa in the categories produced in order to test which of these relationships was best supported. The supertrees produced in each of these separate analyses were combined, again with MRP.

The final supertree, after collapsing all nodes containing no descendant taxa from the time interval under study and removing *post hoc* several taxa whose position could not be resolved, contained 686 species (Supplementary Data 3). It should be noted that the lack of resolution of the position of those taxa which needed to be removed was sometimes due to controversy surrounding their relationships, but it could also be due simply to the fact that a species had not been tested against a wide enough sample of taxa for the MRP method to resolve its position e.g. the assignment to *Nothosaurus* of *N. haasi*, *N. jagisteus*, *N. edingerae*, *N. marchicus*, *N. winterswijkensis*, *N. youngi*, *N. juvenilis*, *N. tchernovi*, *N. winkelhorsti*, *N. yangiuanensis* is not currently controversial and was supported in a recent study[12]. However, since this study employed few outgroup taxa and no other study has included any

Nothosaurus species other than the type and *N. giganteus*, the MRP methods could not resolve the position of all these species relative to other sauropterygians.

It should be noted that molecular analyses have produced an alternative position of turtles not identified by morphological studies: as the sister to Archosauria[13, 14]. Since molecular studies are not included in this analysis (which only includes tree analysing taxa present in the Palaeozoic-early Mesozoic), this potential position of turtles is not tested. While this could potentially affect an analysis of diversification patterns in amniotes later in the Mesozoic or Cenozoic, it is unlikely to affect this analysis; only four turtle taxa in the supertree are identified as present and only three have ghost lineages extending into the Triassic. Their presence in the Lepidosauromorpha in the supertree has not driven any diversification rate shifts in this clade (only two lepidosauromorph shifts have been identified, one at the base of Sauropterygia, one within Ichthyopterygia). If they were placed as the sister to Archosauria, as suggested by the molecular analyses, one of two outcomes could be observed: 1) the diversification rate shift identified at the base of the Archosauria would move, instead occurring in the clade containing Archosauria+Chelonia; 2) the shift would be identified in the same location, and Archosaurs would be shown to have experienced a higher diversification rate than Chelonia during the Triassic.

b) ***List of source trees used in the formation of the supertree***

For references in which more than one dataset was analysed, the tree used in the study is identified by the figure in which the results were presented.

Archosauromorpha

Andres, B., Clark, J. M. & Xing, X. A new rhamphorhynchid pterosaur from the Upper Jurassic of Xingjiang, China, and the phylogenetic relationships of basal pterosaurs. *J.Vert. Paleontol.* **30**, 163-187 (2010)

Bennett, C. S. The phylogenetic position of the Pterosauria within the Archosauromorpha re-examined. *Historical Biol.* 25 1-19 (2012)

Figs. 2 and 3

Benton, M. J. *Scleromochlus taylori* and the origin of dinosaurus and pterosaurs. *Philos. T. Roy. Soc. B.* **354**, 1423-1446 (1999)

Benton, M. J. & Allen, J. L. *Boreopricea* from the Lower Triassic of Russia, and the relationships of the prolacertiform reptiles. *Palaeontology* **40**, 931-953 (1997)

Benton, M. J. & Walker, A. D. *Erpetosuchus*, a crocodile-like basal archosaur from the Late Triassic of Elgin, Scotland. *Zool. J. Linn. Soc.* **136**, 25-47 (2002)

Butler, R. J., Brusatte, S. L., Reich, M., Nesbitt, S. J., Schoch, R. R. & Homung, J. J. The sail-backed reptile *Ctenosauriscus* from the latest Early Triassic of Germany and the timing and biogeography of the early archosaur radiation. *PlosOne* **6**, e25693 (2011)

Figs. 14 and 15

Clark, J. M. & Sues, H.-D. Two new basal crocodylomorph archosaurs from the Lower Jurassic and the monophyly of the Sphenosuchia. *Zool. J. Linn. Soc.* **136**, 77-95 (2002)

Clark, J. M., Xu, X., Forster, C. A. & Wang, Y. A Middle Jurassic sphenosuchian from China and the origin of the crocodilian skull. *Nature* **430**, 1021-2024 (2004)

Desojo, J. B., Ezcurra M. D., Schultz, C. L. An unusual new archosauriform from the Middle-Late Triassic of southern Brazil and the monophyly of Doswelliidae. *Zool. J. Linn. Soc.* **161**, 839-871 (2011)

Dilkes, D. W. The rhynchosaur *Howesia browni* from the Lower Triassic of South Africa. *Palaeontology* **38**, 665-685 (1995)

Dilkes, D. W. The Early Triassic rhynchosaur *Mesosuchus browni* and the interrelationships of basal archosauromorph reptiles. *Philos. T. Roy. Soc. B.* **353**, 501-541 (1998)

Dilkes, D. W. & Arcucci, A. *Proterochampsa barrionuevoi* (Archosauriformes: Proterochampsia) from the Late Triassic (Carnian) of Argentina and a phylogenetic analysis of Proterochampsia. *Palaeontology* **55**, 853-885 (2012)

Ezcurra, M. D. A review of the systematic position of the dinosauriform archosaur *Eucoelophysis baldwini* Sullivan & Lucas, 1999 from the Upper Triassic of New Mexico, USA. *Geodiversitas* **28**, 649-684 (2006)

Ezcurra, M. D. A new early dinosaur (Saurischia: Sauropodomorpha) from the Late Triassic of Argentina: a reassessment of dinosaur origin and phylogeny. *J. Syst. Palaeontol.* **8**, 371-425 (2010)

Ezcurra, M. D. & Novas, F. E. Phylogenetic relationships of the Triassic theropod *Zupaysaurus rougieri* from NW Argentina. *Historical Biol.* **19**, 35-72 (2007)

Ezcurra, M. D., Lecuoina, A. & Martinelli, A. A new basal archosauriform diapsid from the Lower Triassic of Argentina. *J.Vert. Paleontol.* **30**, 1433-1450 (2010)

Franca, M. A. G., Ferigolo, J., Langer, M. C. Associated skeletons of a new Middle Triassic “Rauisuchia” from Brazil. *Naturwissenschaften* **98**, 389-395 (2011)

Gower, D. J. & Sennikov, A. G. *Sarmatosuchus* and the early history of the Archosauria. *J.Vert. Paleontol.* **17**, 60-73 (1997)

Harris, S. R., Gower, D. J. & Wilkinson, M. Intraorganismal homology, character construction, and the phylogeny of aetosaurian archosaurs (Reptilia, Diapsida). *Syst. Biol.* **52**, 239-252 (2003)

Hone, D. W. & Benton, M. J. A new genus of rhynchosaur from the Middle Triassic of South-West England. *Palaeontology* **51**, 95-115 (2008)

Jalil, N. E. A new prolacertiform diapsid from the Triassic of North Africa and the interrelationships of the Prolacertiformes. *J.Vert. Paleontol.* **17**, 506-525 (1997)

Langer, M. C. & Schultz, C. L. A new species of the Late Triassic rhynchosaur *Hyperodapedon* from the Santa Maria formation of South Brazil. *Palaeontology* **43**, 633-652 (2000)

Li, C., Wi, X.-C., Zhao, L.-J., Sato, T. & Wang, L.-T. A new archosaur (Diapsida, Archosauriformes) from the marine Triassic of China. *J.Vert. Paleontol.* **32**, 1064-1081 (2012)

Lü, J. & Ji, Q. Preliminary results of a phylogenetic analysis of the pterosaurs from western Liaoning and surrounding areas. *J. Paleontol. Soc. Korea* **22**, 239-261 (2006)

Martinez, R. N. & Alcobar, O. A. A basal sauropodomorph (Dinosauria: Saurischia) from the Ischigualasto Formation (Triassic, Carnian) and the Early Evolution of Sauropodomorpha. *PlosOne* **4**, e4397 (2009)

Martinez, R. N. et al. A basal dinosaur from the dawn of the dinosaur era in southwestern Pangaea. *Science* **331**, 206-210 (2011)

Modesto, S. P. & Sues H.-D. The skull of the Early Triassic archosauromorph reptile *Prolacerta broomi* and its phylogenetic significance. *Zool. J. Linn. Soc.* **140**, 335-351 (2004)

Montefeltro, F. C., Bittencourt, J. S., Langer, M. C. & Schultz, C. L. Postcranial anatomy of the hyperodapedontine rhynchosaur *Teyumbaita sulcognathus* (Azevedo and Schulty, 1987) from the Late Triassic of Southern Brazil. *J. Vert. Paleontol.* **33**, 67-84 (2013)

Nesbitt, S. J. *Arizonasaurus* and its implications for archosaur divergence. *P. Roy Soc. B.* **270**, 234-237 (2003)

Nesbitt, S. J. The anatomy of *Effigia okeeffeae* (Archosauria, Suchia), theropod-like convergence, and the distribution of related taxa. *B. Am. Mus. Nat. Hist. 302*, 1-84 (2007)

Nesbitt, S. J., Irmis, R. B., Parker, W. G., Smith, N. D., Turner, A. H. & Rowe, T. Hindlimb osteology and distribution of basal dinosauromorphs from the Late Triassic of North America. *J.Vert. Paleontol.* **29**, 498-516 (2009a)

Nesbitt, S. J., Smith, N. D., Irmis, R. B., Turner, A. H., Downs, A. & Norell M. A. A complete skeleton of a Late Triassic saurischian and the early evolution of dinosaurs. *Science* **326**, 1530-1533 (2009b)

Olson, P. E., Sues, H.-D. & Norell, M. A. First record of *Erpetosuchus* (Reptilia: Archosauria) from the Late Triassic of North America. *J.Vert. Paleontol.* **20**, 633-636 (2000)

Parker, W. G. Reassessment of the aetosaur *Desmatosuchus chamaensis* with a reanalysis of the phylogeny of the Aetosauria (Archosauria: Pseudosuchia). *J. Syst. Palaeontol.* **5**, 41-68 (2007)

Parker, W. G. A new species of the Late Triassic phytosaur *Pseudopalatus* (Archosauria: Pseudosuchia) from Petrified Forest National Park, Arizona. In *A Century of Research at Petrified Forest National Park: Geology and Paleontology* (eds Parker, W.G., Ash, S.R., Irmis, R.B.) 126-143 (Museum of Northern Arizona Bulletin 62, 2006)

Parrish, J. M. Phylogeny of the Crocodylotarsi, with reference to archosaurian and crurotarsan monophyly. *J.Vert. Paleontol.* **13**, 287-308 (1993)

Renesto, S. & Binelli, G. *Vallesaurus cenensis* Wild, 1991, a drepanosaurid (Reptilia, Diapsida) from the Late Triassic of Northern Italy. *Riv. Ital. Paleontol. S.* **112**, 77-94 (2006)

Sereno, P. C. Basal archosaurs: phylogenetic relationships and functional implications. *J.Vert. Paleontol.* **11**, 1-53 (1991)

Sereno, P. C. The evolution of dinosaurs. *Science* **284**, 2137-2147 (1999)

Stocker, M. R. A new phytosaur (Archosauriformes, Phytosauria) from the Lot's Wife Beds (Sonslea Member) within the Chinle Formation (Upper Triassic) of Petrified Forest National Park, Arizona. *J.Vert. Paleontol.* **32**, 573-586 (2012)

Wang, X., Kellner, A. W. A., Zhou, Z. & de Almeida Campos, D. Pterosaur diversity and faunal turnover in Cretaceous terrestrial ecosystems in China. *Nature* **437**, 875-879 (2005)

Weinbaum, J. C. & Hungerbühler, A. A revision of *Poposaurus gracilis* (Archosauria: Suchia) based on two new specimens from the Late Triassic of the southwestern USA. *Palaeontol. Z.* **81**, 131-145 (2007)

Yates, A. M. A new species of the primitive dinosaur *Thecodontosaurus* (Saurischia: Sauropodomorpha) and its implications for the systematics of early dinosaurs. *J. Syst. Palaeontol.* **1**, 1-42 (2003)

Figures 22 and 26

Yates, A. M. & Kitching, J. W. The earliest known sauropod dinosaur and the first steps towards sauropod locomotion. *P. Roy Soc. B.* **270**, 1753-1758 (2003)

<u>Ichthyopterygia</u>

Jiang, D.-Y., Schmitz, L., Hao, W.-C. & Sun Y.-L. A new mixosaurid ichthyosaur from the Middle Triassic of China. *J.Vert. Paleontol.* **26**, 60-69 (2006)

Maisch, M. W. & Matzke, A. T. Observations on Triassic ichthyosaurs. Part X: The Lower Triassic *Merriamosaurus* from Spitzbergen – additional data on its anatomy and phylogenetic position. *Neues Jahrb. Geol. P-A.* **227**, 93–137 (2003a)

Maisch, M. W. & Matzke, A. T. Observations on Triassic ichthyosaurs. Part XII. A new Lower Triassic ichthyosaur genus from Spitzbergen. *Neues Jahrb. Geol. P-A.* **229**, 317–338 (2003b)

Maisch, M. W. & Matzke, A. T. Observations on Triassic ichthyosaurs. Part XIV: The Middle Triassic mixosaurid *Phalarodon major* (V. HUENE, 1916) from Switzerland and a reconsideration of mixosaurid phylogeny. *Neues Jahrb. Geol. P-M*, 597–613 (2005)

Motani, R. Phylogeny of the Ichthyopterygia. *J.Vert. Paleontol.* **19**, 473-496 (1999)

Sander, P. M. Ichthyosauria: their diversity, distribution and phylogeny. *Palaeontol. Z.* **74**, 1-35 (2000)

Thorne, P. M., Ruta, M. & Benton, M. J. Resetting the evolution of marine reptiles at the Triassic-Jurassic boundary. *Proc. Natl. Acad. Sci.* **108**, 8339-8344 (2011)

Mini-supertree 3 is formed from Motani (1999) and Thorne et al. (2011)

<u>Lepidosauriformes</u>

Apesteguia, S. & Novas, F. E. Large Cretaceous spehondontian from Patagonia provides insight into lepidosaur evolution in Gondwana. *Nature* **425**, 609-612 (2003)

Apesteguia, S., Gomez, R. O. & Rougier, G. W. A basal sphenodontian (Lepidosauria) from the Jurassic of Patagonia: new insights on the phylogeny and biogeography of Gondwanan rhynchocephalians. *Zool. J. Linn. Soc.* **166**, 342-360 (2012)

Dupret, V. The pleurosaurs: anatomy and phylogeny. Revue de Paléobiologie **9**, 61-80 (2004)

Evans, S. E. & Borsuk-Bialyncka, M. A small lepidosauromorph reptile from the Early Triassic of Poland. *Palaeontol. Pol.* **65**, 179-202 (2009)

Gauthier, J., Estes, R. & de Queiroz K A phylogenetic analysis of Lepidosauromorpha. In *Phylogenetic Relationships of the Lizard Families,* (eds Estes, R.J., Pregill, G.K) 16-98. (Stanford University Press 1988)

Rauhut, O. W. M., Heyng, A. M., López-Arbarello, A. & Hecker, A. A new rhynchocephalian from the Late Jurassic of Germany with a dentition that is unique amongst tetrapods. *PlosOne* **7**: e46739 (2012)

Reynoso, V. H. An unusual aquatic sphenodontian (Reptilia: Diapsida) from the Tlayua Formation (Albian), Central Mexico. *J. Paleontol.* **74**, 133-148 (2000)

Sues, H.-D., Shubin, N. H. & Olsen, P. E. A new sphenodontian (Lepidosauria: Rhynchocephalia) from the McCoy Brook formation (Lower Jurassic) of Nova Scotia, Canada. *J.Vert. Paleontol.* **14**, 327-340 (1994)

Parareptilia

Cisneros, J. C. et al. A procolophonoid reptile with temporal fenestration from the Middle Triassic of Brazil. *P. Roy Soc. B.* **271**, 1541-1546 (2004)

Jalil, N. E. & Janvier, P. Les pareiasaures (Amniota, Parareptilia) du permien supérieur du Bassin d'Argana, Maroc. *Geodiversitas* 27, 35-132 (2005)

Lee, M. Historical burden in systematics and the interrelationships of ‘Parareptiles’. *Biol. Rev.* **70**, 459-547 (1995)

Lee, M. Pareiasaur phylogeny and the origin of turtles. *Zool. J. Linn. Soc.* **120**, 197-280 (1997)

Lyson, T. R., Bever, G. S., Bhullar, B.-A. S., Joyce, W. G. & Gauthier, J. Transitional fossils and the origin of turtles. *Biol. Lett.* **6**: 830-833 (2010)

MacDougall, M. J. & Reisz, R. R. A new parareptile (Parareptilia, Lanthanosuchoidea) from the Early Permian of Oklahoma. *J.Vert. Paleontol.* **32**, 1018-1026 (2012)

Modesto, S. P. & Damiani, R. The procolophonoid reptile *Sauropareion anoplus* from the lowermost Triassic of South Africa. *J.Vert. Paleontol.* **27**, 337-349 (2007)
Figures 7 A,B, 8

Modesto, S. P. & Reisz, R. R. New material of *Colobomycter pholeter*, a small parareptile from the Lower Permian of Oklahoma. *J.Vert. Paleontol.* **28**, 677-684 (2009)

Modesto, S., Sues H.-D. & Damiani, R. A new Triassic procolophonid reptile and its implications for procolophonoid survivorship during the Permo-Triassic extinction event. *P. Roy Soc. B.* **268**, 2047-2052 (2001)

Modesto, S., Damiani, R. J. & Sues H.-D. A reappraisal of *Coletta seca*, a basal procolophonoid reptile from the Lower Triassic of South Africa. *Palaeontology* **45**, 883-895 (2002)

Modesto, S. P., Damiani, R. J., Neveling, J. & Yates, A. M. A new Triassic owenettid parareptile of the Mother of Mass Extinctions. *J.Vert. Paleontol.* **23**, 715-719 (2003)

Müller J., Li J.-L. & Reisz R. R. A new bolosaurid parareptile, *Belebey chengi* sp. nov., from the Middle Permian of China and its paleogeographic significance. *Naturwissenschaften* 95, 1169-1174 (2009)

Reisz, R. R. & Scott, D. *Owenetta kitchingorum*, sp. nov. a small parareptile (Procolophonia: Owenettidae) from the Lower Triassic of South Africa. *J.Vert. Paleontol.* **22**, 244-256 (2002)

Reisz R. R., Müller, J., Tsuji, L. & Scott, D. The cranial osteology of *Belebey vegrandis* (Parareptilia: Bolosauridae) from the Middle Permian of Russia, and its bearing on reptilian evolution. *Zool. J. Linn. Soc.* **151**, 191-214 (2007)

Säilä, L. K. The osteology and affinities of *Anomoiodon liliensterni*, a procolophonid reptile from the Lower Triassic Bundsandstein of Germany. *J.Vert. Paleontol.* **28**, 1199-1205 (2008)

Sues, H.-D. & Reisz, R. R. Anatomy and phylogenetic relationships of *Sclerosaurus armatus* (Amniota: Parareptilia) from the Buntsandstein (Triassic) of Europe. *J.Vert. Paleontol.* **28**, 1031-1042 (2008)

Tsuji, L. A. Cranial anatomy and phylogenetic affinities of the Permian parareptile *Macroleter poezicus*. *J.Vert. Paleontol.* **26**, 849-865 (2006)

Tsuji, L. A. & Müller, J. A re-evaluation of *Parasaurus geinitzi*, the first named pareiasaur (Amniota, Parareptilia). *Can. J. Earth. Sci.* **45**, 1111-1121 (2008)

Mini-supertree 4 is formed from Jalil & Janvier 2005 and Tsuji & Müller 2008

Mini-supertree 5 is formed from Lyson et al 2010, MacDougal & Reisz 2012 and Modesto & Reisz 2009

<u>Sauropterygia</u>

Holmes, R., Cheng, Y.-N., Wu, X.-C. New information on the skull of *Keichousaurus hui* (Reptilia: Sauropterygia) with comments on sauropterygian interrelationships. *J.Vert. Paleontol.* **28**, 76-84 (2008)

Figure 7

Klein, N. & Albers P. C. H. A new species of the sauropsid reptile *Nothosaurus* from the Lower Muschelkalk of the western Germanic Basin, Winterswijk, The Netherlands. *Acta Palaeontol. Pol.* **54**, 589-598 (2009)

O'Keefe, F. R. Preliminary description and phylogenetic position of a new plesiosaur (Reptilia: Sauropterygia) from the Toacian of Holzmaden, Germany. *J. Paleontol.* **78**, 973-988 (2004)

Rieppel, O. *Paraplacodus* and the phylogeny of Placodontia (Reptilia:Sauropterygia). *Zool. J. Linn. Soc.* **130**, 635-659 (2000a)

Rieppel, O. The cranial anatomy of Plancochelys placodonta Jaekel, 1902, and a review of the Cyamodontoidea (Reptilia, Placodonta). *Fieldiana* **45** 1-104 (2000b)

Rieppel, O., Zannon, R. T. The interrelationships of Placodontia. *Historical Biol.* **12**, 211-227(1997)

Rieppel, O., Jinling, L. & Jun, L. *Lariosaurus xingyiensis* (Reptilia: Sauropterygia) from the Triassic of China. *Can. J. Earth. Sci.* **40**, 621-634 (2003)

Sato, T., Cheng, Y.-N., Wu, X.-C. & Li, C. Osteology of *Yunguisaurus* (Reptilia; Sauropterygia), a Triassic pistosauroid from China. *Paleontol. Res.* **14**, 179-195 (2010)

Storrs, G. W. The systematic position of *Silvestrosaurus* and a classification of Triassic sauropterygians (Neodiapsida). *Palaeontol. Z.* **67**, 178-191(1993)

Synapsida

Abdala, F. Redescription of *Platycraniellus elegans* (Therapsida, Cynodontia) from the lower Triassic of South Africa, and the cladistic relationships of eutheriodonts. *Palaeontology* **59**, 591-618 (2007)

Abdala, F., Neveling, J. & Welman, J. A new trirachodontid cynodont from the lower levels of the Burgersdorp Fromation (Lower Triassic) of the Beaufort Group, South Africa and the cladistics relationships of Gondwanan gomphodonts. *Zool. J. Linn. Soc.* **147**, 383-413 (2006)

Amson, E. & Laurin, M. On the affinityies of *Tetraceatops insignis*, an Early Permian synapsid. *Acta Palaeontol. Pol.* **56**, 301-312 (2011)

Angielczyk, K. D. Phylogenetic evidence for and the implications of a dual origin of propaliny in anomodont therapsids (Synapsida). *Paleobiology* **30**, 268-296 (2004)

Angielczyk, K. D. & Rubidge, B. S. Skeletal morphology, phylogenetic relationships and stratigraphic range of *Eosimops newtoni* Broom, 1921, a pylaecephalid dicynodont (Therapsida, Anomodontia) from the Middle Permian of South Africa. *J. Syst. Palaeontol.* **11**, 191-231 (2012)

Benson, R. B. J. The global interrelationships of basal synapsids: cranial and postcranial morphology partitions suggest different topologies. *J. Syst. Palaeontol.* **10**, 601-624 (2012)

Berman, D. S., Reisz, R. R., Bolt, J. R. & Scott, D. The cranial anatomy and relationships of the synapsid *Varanosaurus* (Eupelycosauria: Ophiacodontidae) from the Early Permian of Texas and Oklahoma. *Ann. Carnegie Mus.* **64**, 99-133 (1995)

Bonaparte, J.F., Martinelli, A. G. & Schultz, C. L. New information on *Brasilodon* and *Brasilitherium* (Cnodontia, Probainognathia) from the Late Triassic of southern Brazil. *Rev. Bras. Palaeontol.* **8**, 25-46 (2005)

Botha J., Abdala, F. & Smith, R. The oldest cynodont: new clues on the origin and early diversification of the Cynodontia. *Zool. J. Linn. Soc.* **149**, 477-492 (2007)

Botha-Brink, J. & Modesto, S. P. Anatomy and relationships of the Middle Permian varanopid *Heleosaurus scholtzi* based on a social aggregation from the Karoo Basin of South Africa. *J.Vert. Paleontol.* **29**, 389-400 (2009)

Campione, N. E. & Reisz, R. R. *Varanops brevirostris* (Eupelycosauria: Varanopidae) from the Lower Permian of Texas, with discussion of varanopid morphology and interrelationships. *J.Vert. Paleontol.* **30**, 724-746 (2010)

Cisneros, J. C., Abdala, F., Rubidge, B. S., Dentizien-Dias, P. C. & de Oliveira Bueno, A. Dental occlusion in a 260 million year old therapsid with saber canines from the Permian of Brazil. *Science* **331**, 1603-1605 (2011)

Cisneros, J. C., Abdala, F., Atayman-Güven, S., Rubidge, B. S., Celâl Şengör, A. M. & Schultz CL Carnivorous dinocephalian from the Middle Permian of Brazil and tetrapod dispersal in Pangaea. *Proc. Natl. Acad. Sci.* **109**, 1584-1588 (2012)

Damiani, R., Vasconcelos, C., Renaut, A., Hancox, J. & Yates, A. *Dolichuranus primaevus* (Therapsida: Anomodontia) from the Middle Triassic of Namibia and its phylogenetic relationships. *Palaeontology* **50**, 1531-1546 (2007)

Fröbisch, J. & Reisz, R.R. The postcranial anatomy of *Suminia getmanovi* (Synapsida: Anomodontia), the earliest known arboreal tetrapod. *Zool. J. Linn. Soc.* **162**, 661-698 (2011)

Fröbisch, J., Angielczyk, K. D. & Sidor, C. A. The Triassic dicynodont *Kombuisia* (Synapsida, Anomodontia) from Antarctica, a refuge from the terrestrial Permian-Triassic mass extinction. *Naturwissenschaften* **97**, 187-196 (2010)

Fröbisch, J., Schoch, R. R., Müller, J., Schindler, T., Schweiss, D. A new basal sphenacodontid synapsid from the Late Carboniferous of the Saar-Nahe Basin, Germany. *Acta Palaeontol. Pol.* **56**, 113-120 (2011)

Gebauer, E. V. I. *Phylogeny and Evolution of the Gorgonopsia with a Special reference to the Skull and Skeleton of GPIT/RE/7113 (*'Aelurognathus*?'* parringtoni*)* (Eberhard-Karls Universität 2007)

Govender, R. & Yates, A. Dicynodont postcrania from the Triassic of Namibia and their implication for the systematics of kannemeyeriiform dicynodonts. *Paleontol. Afr.* **44**, 41-57 (2009)

Hopson, J. A. & Kitching, J. W. A probainognathian cynodont from South Africa and the phylogeny of nonmammalian cynodonts. *Bull. Mus. Comp. Zool.* **156**, 5-35 (2001)

Kamerer, C. F. Systematics of the Anteosauria (Therapsida: Dinocephalia). *J. Syst. Palaeontol.* **9**, 261-304 (2011)

Kammerer, C. F., Angielczyk, K. D. & Fröbisch, J. A comprehensive taxonomic revision of *Dicynodon* (Therapsida, Anomodontia) and its implications for dicynodont phylogeny, biogeography, and biostratigraphy. *J.Vert. Paleontol.* **31** (S1), 1-158 (2011)

Kissel, R. A. & Reisz, R. R. Synapsid fauna of the upper Pennsylvanian Rock Lake Shale near Garnett, Kansas and the diversity pattern of early amniotes. In *Recent Advances in the Origin and Early Radiation of Vertebrates*, (eds Arratia, G., Wilson, M.V.H.), pp 409-428. (Verlag Dr. Friedrich Pfeil 2004)

Liu, J. & Li, J.-L. A new material of kannemeyeriid from Xinjiang and the restudy of *Parakannemeyeria brevirostris*. *Vertebrat. PalAsiatic.* **41**, 147–156 (2003)

Liu, J. & Olsen, P. The phylogenetic relationships of Eucynodontia (Amniota: Synapsida). *J. Mamm. Evol.* **17**, 151-176 (2010)

Liu, J., Li, J.-L. & Cheng, Z. The *Lystrosaurus* fossils from Xinjiang and their bearing on the terrestrial Permo-Triassic boundary. *Vertebrat. PalAsiatic.* **40**, 267–275 (2002)

Liu, J., Rubidge, B. S. & Li, J. A new specimen of *Biseridens qilianicus* indicates its phylogenetic position as the most basal anomodont. *P. Roy Soc. B.* **277**, 285-292 (2010)

Lucas, S. G. & Luo, Z. *Adelobasileus* from the Upper Triassic of West Texas: the oldest mammal. *J.Vert. Paleontol.* **13**, 209-334 (1993)

Luo, Z.-X. Sister taxon relationships of mammals and the transformations of the diagnostic mammalian characters. In *In the Shadow of the Dinosaurs – Early Mesozoic Tetrapods,* (eds Fraser, N.C. & Sues, H.-D.) 98-128. (Cambridge University Press 1994)

Luo, Z.-X., Crompton, A. W. & Sun A.-L. A new mammaliaform from the Early Jurassic of China and evolution of mammalian characteristics. *Science* **292**, 1535-1540 (2001)

Maddin, H. C., Evans, D. C. & Reisz, R. R. An Early Permian varanodontine varanopid (Synapsida: Eupelycosauria) from the Richards Spur Locality, Oklahoma. *J.Vert. Paleontol*. **26**, 957-966 (2006)

Maddin, H. C., Sidor, C. A. & Reisz, R. R. Cranial anatomy of *Ennatosaurus tecton* (Synapsida: Caseidae) from the Middle Permian of Russia and the evolutionary relationships of Caseidae. *J.Vert. Paleontol.* 28, 160-180 (2008)

Maisch, M. W. Observations on Karoo and Gondwana vertebrates. Part 2: A new skull-reconstruction of *Stahleckeria potens* von Huene, 1935 (Dicynodontia, Middle Triassic) and a reconsideration of kannemeyeriiform phylogeny. *Neues Jahrb. Geol. P-A.* **220**, 127-152 (2001)

Maisch, M. W. & Gebauer E. V. I. Reappraisal of *Geikia locusticeps* (Therapsida: Dicynodontia) from the Upper Permian of Tanzania. *Palaeontology* **48**, 309-324 (2005)

Martinez, R. N., May, C. L. & Forster, C. A. A new carnivorous cynodont from the Ischigualasto Formation (Late Triassic, Argentina) with comments on eucynodont phylogeny. *J.Vert. Paleontol.* **16**, 271-284 (1996)

Mazierski, D. M., Reisz, R. R. Description of a new specimen of *Ianthasaurus hardestiorum* (Eupelycosauria: Edaphosauridae) and a re-evaluation of edaphosaurid phylogeny. *Can. J. Earth. Sci.* **47**, 901-912 (2010)

Modesto, S. P. The Lower Permian synapsid *Glaucosaurus* from Texas. *Palaeontology* **37**: 51-60 (1994)

Modesto, S. P. & Rybczynski, N. The amniote faunas of the Russian Permian: implications for Late Permian terrestrial vertebrate biogeography. In *The Age of the Dinosaurs in Russia and Mongolia*, (eds Benton, M. J., Shishkin, M.A., Unwin, D.M. & Kurochkin E.N.) pp. 17-35. (Cambridge Univeristy Press 2000)

Modesto, S. P., Rubidge, B. S. & Welman, J. The most basal anomodont therapsid and the primacy of Gondwana in the evolution of anomodonts. *P. Roy Soc. B.* **266**, 331-337 (1999)

Modesto, S. P., Rubidge, B. S., Visser, I. & Welman, J. A new basal dicynodont from the Upper Permian of South Africa. *Palaeontology* **46**, 211-223 (2003)

Oliveira, E. V. Reevaluation of *Therioherpeton cargnini* Bonaparte & Barberena, 1975 (Probainognathia, Therioherpetidae) from the Upper Triassic of Brazil. *Geodiversitas* **28**, 447-465 (2006)

Oliveira, E. V., Soares, M. B. & Schultz, C. L. *Trucidocynodon riograndensis* gen. nov et sp. noc. (Eucynodontia), a new cynodont from the Brazilian Upper Triassic (Santa Maria Formation). *Zootaxa* **2382**, 1-71 (2010)

Ray, S. Functional and Evolutionary aspects of the postcranial anatomy of dicynodonts (Synapsida, Therapsida). *Palaeontology* **49**, 1263-1286 (2006)

Reisz, R. R., Berman, D. S. & Scott, D. The cranial anatomy and relationships of *Secodontosaurus*, an unusual mammal-like reptile (Synapsida: Sphenacodontidae) from the Early Permian of Texas. *Zool. J. Linn. Soc.* **104**, 127-184 (1992)

Reisz, R. R., Godfrey, S. J. & Scott, D. *Eothyris* and *Oedaleops*: do these Early Permian synapsids from Texas and New Mexico form a clade? *J.Vert. Paleontol.* **29**: 39-48 (2009)

Reisz, R. R., Laurin, M. & Marjanović, D. *Apsisaurus witteri* from the Lower Permian of Texas: yet another small varanopid synapsid, not a diapsid. *J.Vert. Paleontol.* **30**, 1628-1631 (2010)

Rowe, T. Phylogenetic systematics and the early history of mammals. In *Mammal phylogeny* 129-145 (Springer: 1993)

Rubidge, B. S. & van den Heever, J. A. Morphology and systematic position of the dinocephalian *Styracocephalus platyrhynchus*. *Lethaia* **30**, 157-168 (1997)

Rubidge, B. S. & Kitching, J. W. A new burnetiamorph (Therapsida: Biarmosuchia) from the lower Beaufort Group of South Africa. *Palaeontology* **46**, 199-210 (2003)

Rubidge, B. S., Sidor, C. A. & Modesto S. P. A new burnetiamorph (Therapsida: Biarmosuchia) from the Middle Permian of South Africa. *J. Paleontol.* **80**: 740-749 (2006)

Rybczynski, N. Cranial anatomy and phylogenetic position of *Suminia getmanovi*, a basal anomodont (Amniota: Therapsida) from the Late Permian of eastern Europe. *Zool. J. Linn. Soc.* **130**, 329-373 (2000)

Sidor, C. A. & Hancox, J. A. *Elliotherium kersteni*, a new tritheledontid from the lower Elliot Formation (Upper Triassic of South Africa). *J. Paleontol.* **80**: 333-342 (2006)

Sidor, C. A. & Hopson, J.A. Ghost lineages and "mammalness": assessing the temporal pattern of character acquisition in the Synapsida. *Paleobiology* **24**, 254-273 (1998)

Sidor, C. A. & Rubidge, B. S. *Herpetoskylax hopsoni*, a new biarmosuchian (Therapsida: Biarmosuchia) from the Beaufort Group of South Africa). In *Amniote Paleobiology* (eds Carrano, M.T., Gaudin, T.J., Blob, R.W. & Wible, J.R.) pp. 76-113 (University of Chicago 2006)

Sidor, C. A. & Smith, R. M. H. A new galesaurid (Therapsida: Cynodontia) from the Lower Triassic of South Africa. *Palaeontology* **47**, 535-556 (2004)

Sidor, C. A. & Smith, R. M. H. A second burnetiamorph therapsid from the Permian Teekloof Formation of South Africa and its associated fauna. *J.Vert. Paleontol.* **27**, 420-430 (2007)

Sidor, C. A. & Welman, J. A second specimen of *Lemurosaurus pricei* (Therapsida: Burnetiamorpha). *J.Vert. Paleontol.* **23**, 631-642 (2003)

Sigurdsen, T., Huttenlocker, A. K., Modesto, S. P., Rowe, T. B. & Damiani, R. Reassessment of the morphology and paleobiology of therocephalian *Tetracynodon darti* (Therapsida), and the phylogenetic relationships of the Baurioidea. *J.Vert. Paleontol.* **32**, 1112-1134 (2012)

Smith, R. M. H., Rubidge, B. S. & Sidor, C. A. A new burnetiid (Therapsida: Biarmosuchia) from the Upper Permian of South Africa and its biogeographic implications. *J.Vert. Paleontol.* **26**, 331-343 (2006)

Sues H.-D. & Hopson, J. A. Anatomy and phylogenetic relationships of *Boreogomphodon jeffersoni* (Cynodontia: Gomphodontia) from the Upper Triassic of Virginia. *J.Vert. Paleontol.* **30**, 1202-1220 (2010)

Surkov, M. V., Kalandadze, N. N. & Benton, M. J. *Lystrosaurus georgi*, a dicynodont from the Lower Triassic of Russia. *J.Vert. Paleontol*, **25**: 402-413 (2005)

Vega-Dias, C., Maisch, M. W. & Schultz, C. L. A new phylogenetic analysis of Triassic dicynodonts (Therapsida) and the systematic position of *Jachaleria candelariensis* from the Upper Triassic of Brazil. *Neues Jahrb. Geol. P-A.* **231**, 145–166 (2004)

Wible J. R. Origin of Mammalia: the craniodental evidence re-examined. *J.Vert. Paleontol.* **11**, 1-28 (1991)

Mini-supertree 6 is formed from Angielczyk 2004, Modesto et al 1999, Modesto & Rybzynski 2000 and Rybzynski 2000

Mini-supertree 7 is formed from Botha Brink & Modesto 2009, Campione & Reisz 2010 and Maddin et al 2006

Mini-supertree 8 is formed from Cisneros et al. 2012 and Kammerer 2011

Mini-supertree 9 is formed from Sidor & Smith 2007 and Smith et al. 2006

<u>Chelonia</u>

Anquetin, J. Reassessment of the phyloegentic interrelationships of basal turtles (Testudinata). *J. Syst. Palaeontol.* **10**, 3-45 (2012)

Anquetin, J., Barrett, P. M., Jones, M. H., Moore-Fay, S. & Evans, S. E. A new stem turtle from the Middle Jurassic of Scotland: new insights into the evolution and palaeoecology of basal turtles. *Proc. R. Soc. B.* **276**, 879-886 (2009).

Gaffney E. S., Rich, T. H., Vickers-Rich, P., Constantine, A., Vacca, R. & Kool, L. *Chubutemys,* a new eucryptodiran turtle from the Early Cretaceous of Argentina and the relationships of the Meiolaniidae. *Am. Mus. Novit.* **3599**, 1-35 (2007)

Li, C., Wu, X.-C., Rieppel, O., Wang, L.-T. & Zhao, L.-J. An ancestral turtle from the Late Triassic of southwestern China. *Nature* **456**: 497-501 (2008)

Rougier, G. W., de la Fuente, M. S. & Arcucci, A. B. Late Triassic turtles from South America. *Science* **268**, 855-858 (1995)

Sterli, J. Phylogenetic relationships among extinct and extant turtles: the position of Pleurodira and the effects of the fossils on rooting crown-group turtles. *Contrib. Zool.* **79**, 93-106 (2010)

Mini-supertree 10 is formed from Li et al. 2008 and Sterli 2010

<u>Basal</u>

Bickelmann, C., Müller, J., Reisz, R. R. The enigmatic diapsid *Acerosodontosaurus piveteaui* (Reptilia: Neodiapsida) from the Upper Permian of Madagascar and the paraphyly of “younginiform” reptiles. *Can. J. Earth. Sci*. **46**, 651-661 (2009)

DeBraga, M. & Reisz, R. R. A new diapsid reptile from the uppermost Carboniferous (Stephanian) of Kansas. *Palaeontology* **38**, 199-212 (1995)

Evans, S. E. An early kuehneosaurid reptile from the Early Triassic of Poland. *Palaeontol. Pol.* **65**, 145-178 (2009)

Hill, R. V. Integration of morphological data sets for phylogenetic analysis of Amniota: the importance of integumentary characters and increased taxonomic sampling. *Syst. Biol.* **54**, 530-547 (2005)

Jiang, D.-Y., Maisch, M.-W., Sun, Y.-L., Matzke, A. T. & Hao W.-C. A new species of *Xinpusaurus* (Thalattosauria) from the Upper Triassic of China. *J.Vert. Paleontol*. **24**, 80-88 (2004)

Kissel, R. *Morphology, Phylogeny and Evolution of Diadectidae (Cotylosauria: Diadectomorpha)* (University of Toronto 2010).

Laurin, M. & Reisz, R. R. A new perspective on tetrapod phylogeny. In *Amniote Origins: Completing the Transition to Land*, (eds Sumida, S. & Martin, K.L.M.) pp. 9 (San Diego Academic Press 1997)

Lee, M. Molecules, morphology, and the monophyly of diapsid reptiles. *Contrib. Zool.* **70**: 1-22 (2001)

Li, C., Wu, X.-C., Rieppel, O., Wang, L.-T. & Zhao, L.-J. An ancestral turtle from the Late Triassic of southwestern China. *Nature* **456**, 497-501 (2008)

Liu, J. & Rieppel, O. Restudy of *Anshunsaurus huangguoshuensis* (Reptilia: Thalattosauria) from the Middle Triassic of Guizhou, China. *Am. Mus. Novit.* **3488**, 1-34 (2005)

Modesto, S. P. & Reisz, R. R. An enigmatic new diapsid reptile from the upper Permian of Eastern Europe. *J.Vert. Paleontol.* **22**, 851-855 (2002)

Müller, J. Early loss and multiple return of the lower temporal arcade in diapsid reptiles. *Naturwissenschaften* **90**: 473-476 (2003)

Müller, J. The anatomy of *Askeptosaurus italicus* from the Middle Triassic of Monte San Giorgio and the interrelationships of thalattosaurs (Reptilia, Diapsida). *Can. J. Earth. Sci.* **42**, 1347-1367 (2005)

Müller, J. & Reisz, R. R. The phylogeny of early eureptiles: comparing parsimony and Bayesian approaches in the investigation of a basal fossil clade. *Syst. Biol.* **55**, 503-511 (2006)

Reisz, R. R., Liu, J., Li, J. L. & Müller, J. A new captorhinid reptile, *Gansurhinus qingtozshanensis*, gen. et sp. nov., from the Permian of China. *Naturwissenschaften* **98**: 435-441 (2011)

Reisz, R. R., Modesto, S. P. & Scott, D. M. A new Early Permian reptile and its significance in early diapsid evolution. *P. Roy Soc. B.* **278**, 3731-3737 (2011)

Rieppel, O. & DeBraga, M. Turtles as diapsid reptiles. *Nature* 384: 453-455

Sumida, S. S., Dodick, J., Metcalf, A. & Albright, G. *Reiszorhinus olsoni*, a new single-tooth-rowed captorhinid reptile from the Lower Permian of Texas. *J.Vert. Paleontol.* **30**, 704-714 (2010)

Wu, X. C., Cheng, Y. N., Sato, T. & Shan, H. Y. *Miodentosaurus brevis* Cheng et al., 2007 (Diapsida: Thalattosauria): its postcranial skeleton and phylogenetic relationships. Vertebrat. *PalAsiatic.* **47**, 1-20 (2009)

Mini-supertree 1 formed from Bickelmann et al. 2009, Evans 2009 and Reisz et al. 2011

Mini-supertree 2 formed from Hill 2005 and Li et al. 2009

c) *List of taxa removed*

Basal

Anthracodromeus longiceps

Protorothyris archeri

Cephalerpeton ventriarmatum

Hylonomus lyelli

Hovasaurus boulei

Thadeosaurus colcanapi

Galesphyrus capensis

Kenyasaurus mariakaniensis

Synapsids

Traversodon stahleckeri

Propelanomodon devilliersi

Dicynodon trigonocephalus

Uralokannemeyeria vjuschkovi

Xiyukannemeyeria brevirostris

Zambiasaurus submersus

Prorubidgea spp.

Lystrosaurus youngi

Cteniosaurus platyceps

Mirotenthes digitipes

Ictidostoma hemburyi

Hofmeyria atavus

Euchambersia mirabilis

Parareptilia

Bunostegos akokanensis

Koiloskiosaurus cogburgensis

Nanoparia luckhoffi

Sauropterygia

ELASMOSAURIDAE

Neusticosaurus peyeri

Neusticosaurus.toeplitschi

Neusticosaurus edwardsi

Microcleidus homalospondylus

Saurosphargis volzi

Hanosaurus hupehensis

Wumengosaurus delicatomandibularis

Qianxisaurus chajiangsensis

Dianopachysaurus dingi

Diandongosaurus acutidentatus

Chicenia sungi

Kwangsisaurus orientalis

Sanchiaosaurus dengi

Sinosaurosphargis yunguiensis

Nothosaurus haasi

Nothosaurus giganteus

Nothosaurus jagisteus

Nothosaurus edingerae

Nothosaurus marchicus

Nothosaurus winterswijkensis

Nothosaurus youngi

Nothosaurus juvenilis

Nothosaurus tchernovi

Nothosaurus winkelhorsti

Nothosaurus yangiuanensis

Ceresiosaurus spp.

<u>Lepidosauromorpha</u>

Polysphenodon muelleri

Clevosaurus wangi

<u>Archosauromorpha</u>

Nicrosaurus kapffi

Nicrosaurus meyeri

Eocursor parvus

Mystriosuchus planirostris

Ebrachosuchus neukami

Boreopricea funerea

Jesairosaurus lehmani

Malerisaurus robinsonae

Pseudohesperosuchus jachaleri

Turfanosuchus dabensis

Yonghesuchus sangbiensis

Procompsognathus triassicus

Gojirasaurus quayi

Segisaurus halli

Saltoposuchus connectens

Preondactylus buffarinii

Chilenosuchus forttae

Jingshanosaurus xinwaensis

Yunnanosaurus spp.

Ichthyopterygia

Thaisaurus chonglakmanii

Guizhouichthyosaurus tangae

d. Time calibration

Time calibration of a phylogeny can be a complex issue due to zero-length branches, these result when taxa appear in the fossil record in a different order to that suggested by the branching shown in the phylogeny. These imply instantaneous speciation and character evolution, both unrealistic.

A variety of methods exist to eliminate these zero-length branches. The majority involve selecting an arbitrary length of time, and adding this to all branches[15] or to zero-length branches only[16], or extending the length of branches until all reach an arbitrary minimum length[17]. Two less arbitrary methods have been proposed. Both involve extending zero-length branches back in time along the non-zero-length branch immediately ancestral to them, sharing this ancestral branch's length among the zero-length branches descended from

it. The zero-length branches may be shared equally among the ancestral branch[18] or, if a morphological character matrix exists, adjusting branch lengths to represent the proportion of morphological change occurring along each branch[19]. In this case the former method is used due to the lack of character data.

100 of the MPTs found in the MRP analysis were randomly selected for the analysis. While SymmeTREE does include the option to randomly resolve polytomies, some random resolutions will be suboptimal (less parsimonious)[20]. In order to deal with the uncertainty surrounding the ages of certain taxa, 100 sets of first and last appearences were drawn for each taxon from a uniform proability distribution covering the full possible range of ages for each taxon[21].

e. Sensitivity analyses

Two possible sources of error were investigated: poorly supported relationships and missing taxa. Ideally, a supertree analysis should represent a summary of researchers' analyses into clade relationships. Although in theory an MRP supertree should not contain any relationships that have never before been suggested, unsupported relationships can appear, albeit rarely[5]. For these reasons, it is necessary to provide a support measure indicating to what extent the source trees support the relationships shown in the supertree. In this study, the V measure was used[22]. Each node is assigned a value between -1 and 1, representing the proportion of source trees supporting the node in question relative to the source trees that do not support it (see Supplementary Data 9 for the support values assigned to each node). In order to ensure that poorly supported relationships do not unduly affect the inferences, the analyses with SymmeTREE were repeated after all nodes with negative support were collapsed into a polytomy, which were then randomly resolved 100 times.

The second potential source of error is the impact of missing taxa. While this supertree is the most comprehensive representation of early amniote relationships available, there are

taxa which have not yet been included in any phylogenetic analyses and higher taxa which have not been included in analyses below genus or family level e.g. *Sphenacodon*, Mesosauridae. One could address this by replacing all higher taxa in the supertree with their constituant species in a polytomy, and then randomly resolving those polytomies in the analysis. However, in the case of early amniotes, this presents a problem; a great many clades have not had their species level taxonomy examined in a cladistic context; in fact a great many of the species level taxonomies have not been questioned since the 1960s or even earlier[23], a time when classification was often based on stratigraphy, location or body size. In order to assess the impact of the use of higher taxa, an expanded supertree was subjected to the same set of analyses in SymmeTREE. Terminal taxa above species level in the supertree were replaced with a polytomy containing all species currently considered valid, provided this species level taxonomy had been confirmed in recent study (more recently than 25 years). Higher taxa whose species-level taxonomy had not been examined since 1990 were kept as higher taxa. These polytomies were randomly resolved 100 times. The expanded supertree contained 742 taxa and is presented in Supplementary Data 4.

We found that neither uncertainty surrounding taxon age nor poorly supported relationships substantially affect the results. When nodes with negative support are collapsed into a polytomy, the timing and location of substantial and significant diversification shifts identified by SymmeTree are unchanged from those found using the original supertree, with one exception; a diversification rate shift within the Kannemeyeriiformes is not identified when poorly supported nodes are collapsded (Supplementary Data 7). When missing taxa are included, three extra diversification rate shifts are identified. Two of these occur within the therapsid clade Gorgonopsia (at the base of Rubidginae and at the base of Gorgonopsidae), due to the fact that many speciose gorgonopsian genera are only included at the genus level in the basic supertree. The third new diversification rate shift was identified at the base of the

ichthyosaur clade Thunnosauria, again due to the large number of speciose taxa only included at the genus level in the original supertree.

The impact of using different methods of time slicing was investigated. Ruta et al.[19] argued for pruning the tree to include only those taxa present in a particular time slice, since Symmetree is designed for use on ultrametric trees. Tarver & Donoghue[24] suggested an alternative in which the tree was grown through time, with new taxa added in each substage, and retaining taxa from preceding time slices. The rationale behind this method is that each speciation event is retained in the tree, allowing the identification of uneven rates of origination or extinction as the driver behind a particular shift[24]. We carried out the shift analyses using both time-slicing methods, hereafter referred to as the Ruta and the Tarver methods.

The use of different time-slicing methods has a greater effect on shift distributions than age (Supplementary Data 7). The Ruta method identified 22 clades experiencing substantial diversification shifts, while the Tarver method identified 28. Of these clades, only 14 were identified by both methods simultaneously. The timing of these 14 shifts differed in most cases; in only five of these 14 clades was the timing of the diversification shift found to be the same when both methods of time slicing are used. Eight clades show shifts only when the Ruta method was used, and 14 clades show shifts only when the Tarver method was used.

The results presented in the main text are those using the ages that take into account uncertainties in dating, as well as the Ruta method of time-slicing. The Tarver method is rejected as it reintroduces the problem that Ruta et al.[19] were trying to solve: the inclusion of extinct taxa means not all lineages will have had equal time to diversify and the assumptions of the Yule model are violated. It has been argued that the Ruta method of time slicing is misleading as the time sliced trees do not include all cladogenetic events. However we would argue that this is not a serious problem when investigating shifts in the rate of diversification. If less cladogenetic events are observed in one lineage than in its sister, this could imply

increased cladogenesis in the sister, or it could imply increased extinction in the lineage having “removed” the speciation events from the observed tree. However, both of these possibilities imply a diversification rate shift. It is true that under the Ruta method one cannot distinguish between these two possibilities, but one can investigate which is more likely by comparing the number and magnitude of shifts to origination and extinction rates, as is done in this paper.

f. Inferring Origination and Extinction Rates from Phylogenies

The inference of origination and extinction rates is central to the analyses presented here and is key to many other macroevolutionary investigations. However, as in all fossil-based evolutionary studies, such analyses have to contend with the issue of sampling biases in the fossil record. First documented occurrences of species in the fossil record are unlikely to be synchronous with the time of origination of those species. It follows that quality and intensity of sampling in any time interval will dictate whether a speciation event occurred in the same time interval where a specimen has been observed, or in an earlier interval. Thus, the better the quality of the fossil record in the time bin immediately preceding the time of first appearance of any given taxon, the less likely the chance that the taxon in question was actually present in earlier time bins than that where it is observed. The same reasoning can be extended to estimates of extinction rates; the last documented occurrence of a lineage in the fossil record is unlikely to represent its very last individual, and the likelihood that the lineage actually went extinct in the same time bin as its observed disappearance will depend on the quality of sampling in later time bins.

A wide range of methods have been devised to incorporate estimates of sampling into origination and extinction rate estimates[25-28]. However, the vast majority of such methods may not be appropriate for early amniotes, certainly due to the fact that many of the proposed methods rely on a random and extensive sample of multiple individuals (different invertebrate

groups offer notable examples) for which dating is relatively unproblematic. Most available methods rely on observed distributions of taxa within and across adjacent time intervals, including whether such taxa cross the bottom and/or top boundaries of an interval, and whether they occur in adjacent intervals. Moreover, such methods do not include singleton taxa (i.e., taxa found exclusively within an interval) [25, 26], which are extensively represented among early amniotes.

Here, a new method is proposed that seeks to correct for sampling in origination and extinction rates, using a time-calibrated phylogeny as a starting point. The per-lineage origination and extinction rates are calculated from the tree, the former by counting the number of cladogenetic events in each time bin, the latter by counting the number of lineages terminating in that time bin.

The origination rate for a given time bin is then multiplied by an estimate of sampling probability from the previous time bin. If the sampling probability in the previous time bin is low, then we can be less sure that the taxa observed in the time bin of interest actually originated in that bin, and therefore our origination rate needs to be lowered. Multiplying the observed extinction rate by the sampling probability (a value between 0 and 1) means that the lower the sampling probability, the more the origination rate will be lowered, representing the fact that one can be less sure that the origination events observed in a particular time bin actually occurred in that bin. A similar correction applies in the case for extinction rates; thus, the estimated extinction rate of the time bin of interest is multiplied by an index that gives a measure of sampling from the following time bin.

The shape of the phylogeny offers a way to estimate sampling probability in each time bin. For each time bin, the number of observed lineages (those represented by fossil occurrences) is divided by the total number of lineages (observed plus ghost lineages) in that time bin. As ghost lineages represent portions of the fossil record that may be inferred to be present but have not been sampled, the proportion of observed lineages relative to combined

observed and ghost lineages provides an operational estimate (albeit one that can be constantly refined by novel taxon discoveries) of sampling in that bin (R code for calculating the origination and extinction rates using these methods are supplied in Supplementary Data 5 and 6).

In order to ensure that the proposed method provides reliable estimates of origination and extinction, simulations were carried out. The simulations were undertaken using the *simFossilTaxa* function in the R paleotree package[29]. This function produces a stochastic birth-death model which allows anagenetic, cladogenetic, and budding speciation based upon user-defined probabilities (for the analysis presented here, the probability of each mode of speciation was set to be equal). A second paleotree function, *taxa2cladogram,* was used to produce a cladogram using information from the output of the birth-death model. This cladogram takes into account resolutions of ancestor-descendant relationships based upon current phylogenetic methods (i.e., ancestors are resolved either as sister taxa to, or in a polytomy with, descendants, depending on the model of speciation).

These two functions together produce a list of taxa, their temporal ranges, and a cladogram summarising patterns of relationships based upon initial parameters (e.g. origination and extinction rates). The simulated time period was spilt into equal-length bins, and for each taxon in each bin we assigned a sampling probability (the simulations were run with sampling probabilities from 0.1 to 0.9, with equal increments of 0.1). Once the sampling filter had been applied, the tree (with unsampled taxa pruned) was time calibrated using the new age ranges (potentially ranges might be reduced by sampling). The per-lineage origination and extinction rates were calculated using two methods: 1) a raw count, which consists in dividing the observed number of cladogenetic events in each time bin by the observed number of lineages in that bin in order to get the origination rate (for extinction rates, we divided the observed number of lineage terminations in each time bin by the observed number of lineages in that bin); and 2) an inferred count (the method described

above), whereby we correct for sampling using the proportion of ghost lineages in each time bin. Both of these methods were compared to the 'true' origination and extinction rate curves obtained from the simulated data before applying the sampling filter, using the Spearman's rank correlation test. These simulations were repeated 1000 times at each sampling probability.

The results of the simulations (Supplementary Table 1) suggest that, at low sampling levels, the new method proposed here provides a close correspondence to the true origination and extinction rates, and outperforms the raw count method. Unsurprisingly, both methods perform worse at low levels of sampling, but the drop in performance of the new method is considerably less pronounced; even at a sampling probability of 0.2, the mean Spearman's rho value (comparing the rates produced by the new method to the 'true' rates generated by simulations) is greater than 0.6, but only about 0.3 for the raw count.

Interestingly, at high levels of sampling (probability of sampling greater than 0.6), the raw count method outperforms the new method. A possible explanation for this finding can be found in the treatment of ancestors in the phylogeny. As discussed above, our current phylogenetic methods resolve ancestors either as the sister to, or in a polytomy with, their descendants. This means that a ghost lineage is inferred where one may not exist. At higher sampling levels, it is considerably more likely that both an ancestor and its descendant will be sampled in the same time bin, a false ghost lineage will be inferred and the sampling estimate will be less reliable. This shows that our new method for estimating origination and extinction rates may be inappropriate for very well sampled clades. However, the new method offers much promise, particularly in studies of Palaeozoic terrestrial vertebrates, for which the quality of the fossil record is often regarded as poor[23,30-31].

REFERENCES

1. Baum, B. R. Combining trees as a way of combining datasets for phylogenetic inference, and the desirability for combining gene trees. *Taxon* 41, 1-10 (1992).

2. Ragan, M. A. Phylogenetic inference based on matrix representation of trees. *Mol. Phylogenet. Evol.* 1: 53-58 (1992).

3. Beninda-Emonds, O, R & Bryant, N. H. Properties of matrix representation with parsimony analyses. *Syst. Biol.* **47**, 497-508 (1998)

4. Beninda-Emonds, O. R., Gittleman, J. L. & Steel, M. A. The (super) Tree of Life: procedures, problems and prospects. *Annu. Rev. Ecol. Syst.* **33**, 265-289 (2002).

5. Beninda-Emonds, O. R. Novel versus unsupported clades: assessing the qualitative support for clades in MRP supertrees. *Syst. Biol.* **52**: 839-848 (2003).

6. Wilkinson, M., Thorley, J. L., Littlewood, D. T. J., & Bray, R. A. Towards a supertree for the Platyhelminthes? In *Interrelationships of the Platyhelminthes* (eds. Littlewood D. T. J., Bray, R. A.) (Chapman-Hall 2001).

7. Wilkinson, M., et al. The shape of supertrees to come: tree shape related properties of fourteen supertree methods. *Syst. Biol.* **54**, 419-431 (2005).

8. Gatesy, J., Matthee, C., DeSalle, R. & Hayashi, C. Resolution of a supertree/supermatrix paradox. *Syst. Biol.* **51**, 652-664 (2002).

9. Beninda-Emonds, O. R., Jones, K. E., Price, S. A., Cardillo, M., Greyner, R. & Purvis, A. Garbage in, garbage out. Data issues in supertree construction. In *Phylogenetic Supertrees: Combining Information to Reveal the Tree of Life* (ed. Beninda-Emonds O.R.) (Kluwer Academic Publishers 2004)

10. Salamin, N., Hodkinson, T. R. & Savolainen, V. Building supertrees: an empirical assessment using the grass family (Poaceae). *Syst. Biol.* **51**, 134-150 (2002).

11. Goloboff, P. A., Farris, J. S. & Nixon, K. C. TNT, a free program for phylogenetic analysis. *Cladistics* **24**, 774-778 (2008).

12. Klein, N. & Albers, P. C. H. A new species of the sauropsid reptile *Nothosaurus* from the Lower Muschelkalk of the western Germanic Basin, Winterswijk, The Netherlands. *Acta Palaeontol. Pol.* **54**, 589-598 (2009).

13. Lu, B., Yang, W. & Fu, J. Using genes as characters and a parsimony analysis to explore the phylogenetic position of turtles. *PlosOne* **8**, e79348 (2013)

14. Crawford, N.G., Faircloth, B.C., McCormack, J.E., Brumfield, R.T., Winker, K., Glen, T.C. More than 1000 ultraconserved elements provide evidence that turtles are the sister group of archosaurs. *Biol. Lett.* **8**, 783-786

15. Ruta, M., Cisneros, J. C., Liebrecht, T., Tsuji, L. A. & Müller, J. Amniotes through major biological crises: faunal turnover among parareptiles and the end-Permian mass extinction. *Palaeontology* **54**, 1117-1137 (2011)

16. Hunt, G. & Carrano, M. T. Models and methods for analysing phenotypic evolution in lineages and clades. In *Short Course on Quantitative Methods in Paleobiology* (eds. Alroy, J., Hunt, G.) (Paleontological Society)

17. Laurin, M. The evolution of body size, Cope's Rule and the origin of amniotes. *Syst. Biol.* **4**, 594-622 (2004)

18. Brusatte, S. L., Benton, M. J., Ruta, M., & Llody, G. T. Superiority, competition and opportunism in the evolutionary radiation of dinosaurs. *Science* **321**, 1485-1488 (2008)

19. Ruta, M., Pisani, D., Lloyd, G. T. & Benton, M. J. A supertree of Temnospondyli: cladogenetic patterns in the most species-rich group pf early tetrapods. *Proc. R. Soc. B* **274**, 3087-3095 (2007).

20. Bell, M. A. & Lloyd, G. T. strap, an R package for plotting phylogenies against stratigraohy and assessing their stratigraphic congruence. *Palaontology* **58**, 379-389 (2014)

21. Pol, D. & Norell, M.A. Uncertainty in the age of fossils and the stratigraphic fit to phylogenies. *Syst Biol* **55**, 512-521.

22. Wilkinson, M., Pisani, D., Cotton, J. A. & Corfe, I. Measuring support and finding unsupported relationships in supertrees. *Syst Biol* **54**, 823-831 (2005).

23. Brocklehurst, N. & Fröbisch, J. Current and historical perspectives on the completeness of the fossil record of pelycosaurian-grade synapsids. *Palaeogeogr. Palaeocl.* **399**, 114-126 (2014).

24. Tarver, J. & Donoghue, P. The trouble with topology: Phylogenies without fossils provide a revisionist perspective of evolutionary history in topological analyses of diversity. *Syst. Biol.* **60**, 700-712 (2011)

25. Foote, M. Temporal variation in extinction risk and temporal scaling of extinction metrics. *Paleobiology* **20**, 424-444 (1994).

26. Foote, M. Origination and extinction components of taxonomic diversity: general problems. In *Deep time: Paleobiology's perspective.* (eds Erwin D. H. & Wing S. L.) (Paleobiology suppl 26, 2000)

27. Alroy, J. Dynamics of origination and extinction in the marine fossil record. *Proc Nat Acad Sci* **56**, 536-542 (2008).

28. Alroy, J. Accurate and precise estimates of origination and extinction rates. *Paleobiology* **40**, 374-397 (2014).

29. Bapst D. W. Paleotree: an R package for paleontological and phylogenetic analyses of evolution. *Methods. Ecol. Evol.* **3**, 803-803 (2012).

30. Benson R. B. J. & Upchurch, P. Diversity trends in the establishment of terrestrial vertebrate ecosystems: interactions between spatial and temporal sampling biases. *Geology* **41**, 43-46 (2012).

31. Brocklehurst, N., Kammerer, C. F. & Fröbisch, J. The early evolution of synapsids, and the influence of sampling on their fossil record. *Paleobiology* **39**, 470-490 (2013).

Supplementary Table 1

Results of the simulation study testing the accuracy of the origination and extinction rates deduced by the method described in the supplmentary text. Sampling level: 1-probability that each taxon may be deleted from a time bin in which it is present. Results represent the mean Spearman's rho value of 1000 comparisons between rate estimates from simulated data

Sampling level	Origination rate calculated using new method of sampling correction described in Supplementary Method	Origination rate calculated from the tree: no sampling correction	Extinction rate calculated using new method of sampling correction described in Supplementary Method	Extinction rate calculated from the tree: no sampling correction
0.1	0.534189	0.3045735	0.589352829	0.3145735
0.2	0.600342	0.3457919	0.655505829	0.3757919
0.3	0.629017	0.4070103	0.684180829	0.4370103
0.4	0.648123	0.4882287	0.703286829	0.5382287
0.5	0.674219	0.5394471	0.729382829	0.6294471
0.6	0.6981095	0.6506655	0.733273329	0.7006655
0.7	0.7221832	0.7418839	0.754546658	0.7618839
0.8	0.7462569	0.8031023	0.777347029	0.8631023
0.9	0.7703306	0.8943207	0.801420729	0.9443207

Supplementary Figure 1

Results of the simulation study testing the accuracy of the origination and extinction rates deduced by the method described in the supplmentary text. Sampling level: 1-probability that each taxon may be deleted from a time bin in which it is present. Results represent the mean Spearman's rho value of 1000 comparisons between rate estimates from simulated data

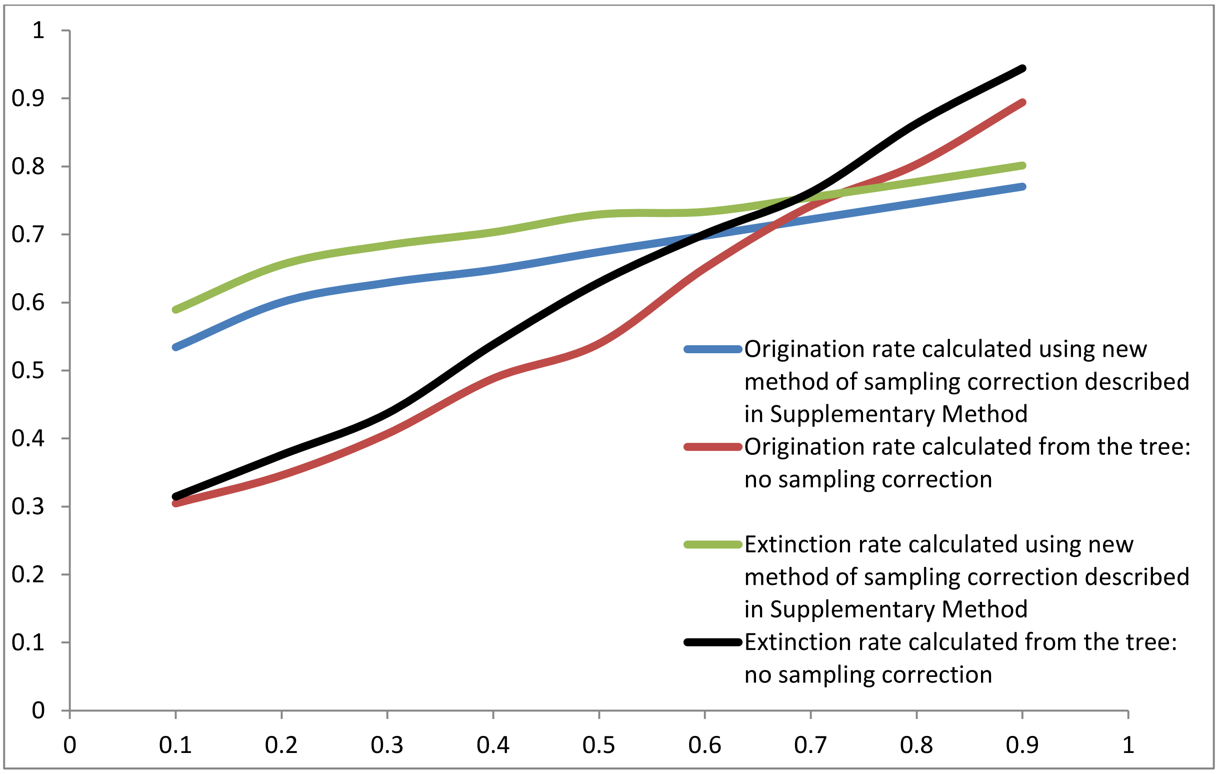

Supplementary Figure 2-15

Strict concensus phylogenies time sliced using the Ruta method, illustrating substantial (grey) and significant (black) diversification rate shifts. The shifts are marked at the node where substantial imbalance is identified using SymmeTREE; therefore the relative diversification increase is deemed to have occurred in the more diverse of the two descendant lineages of that node.

Supplementary Figure 2
Late Artinskian

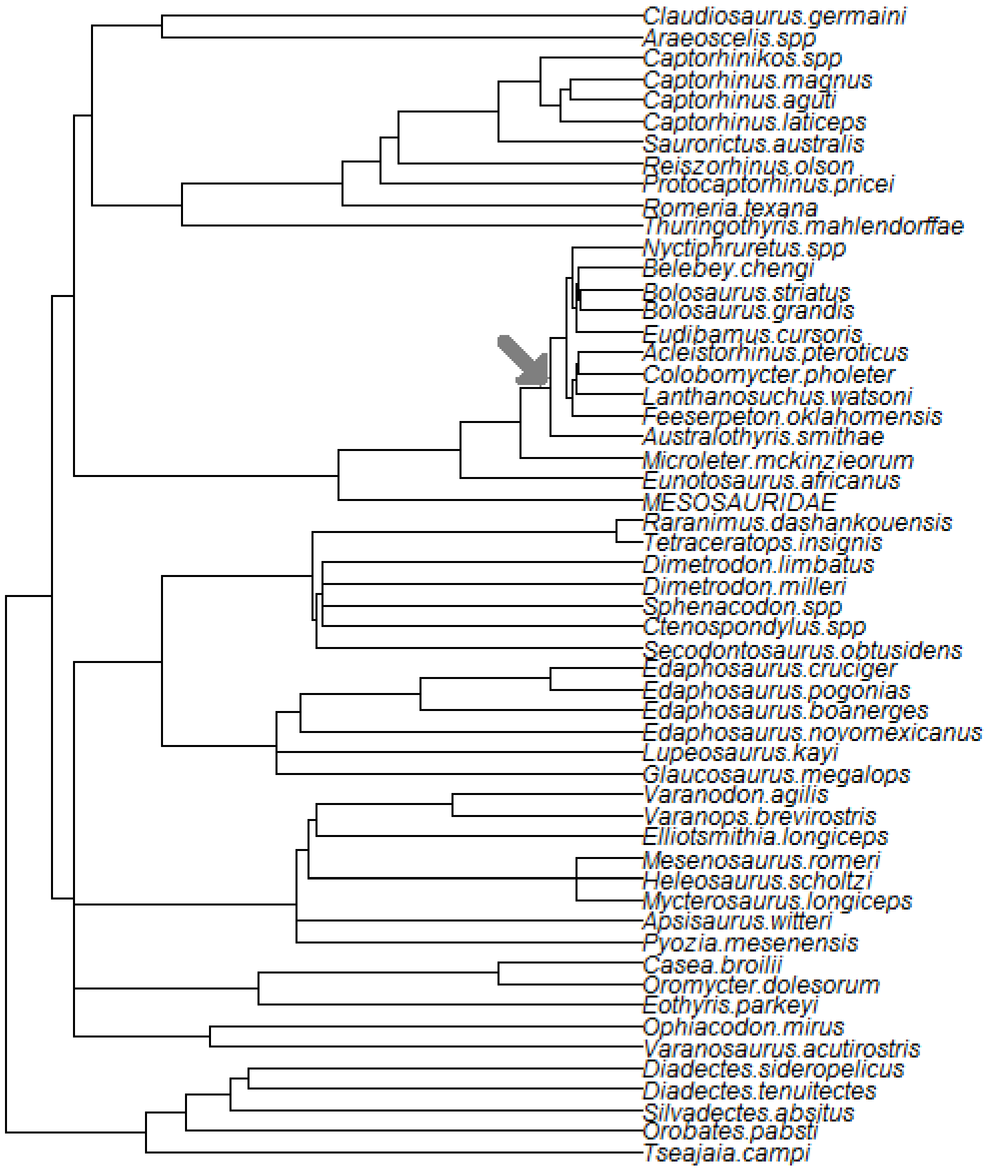

Figure 3
Late Kungurian

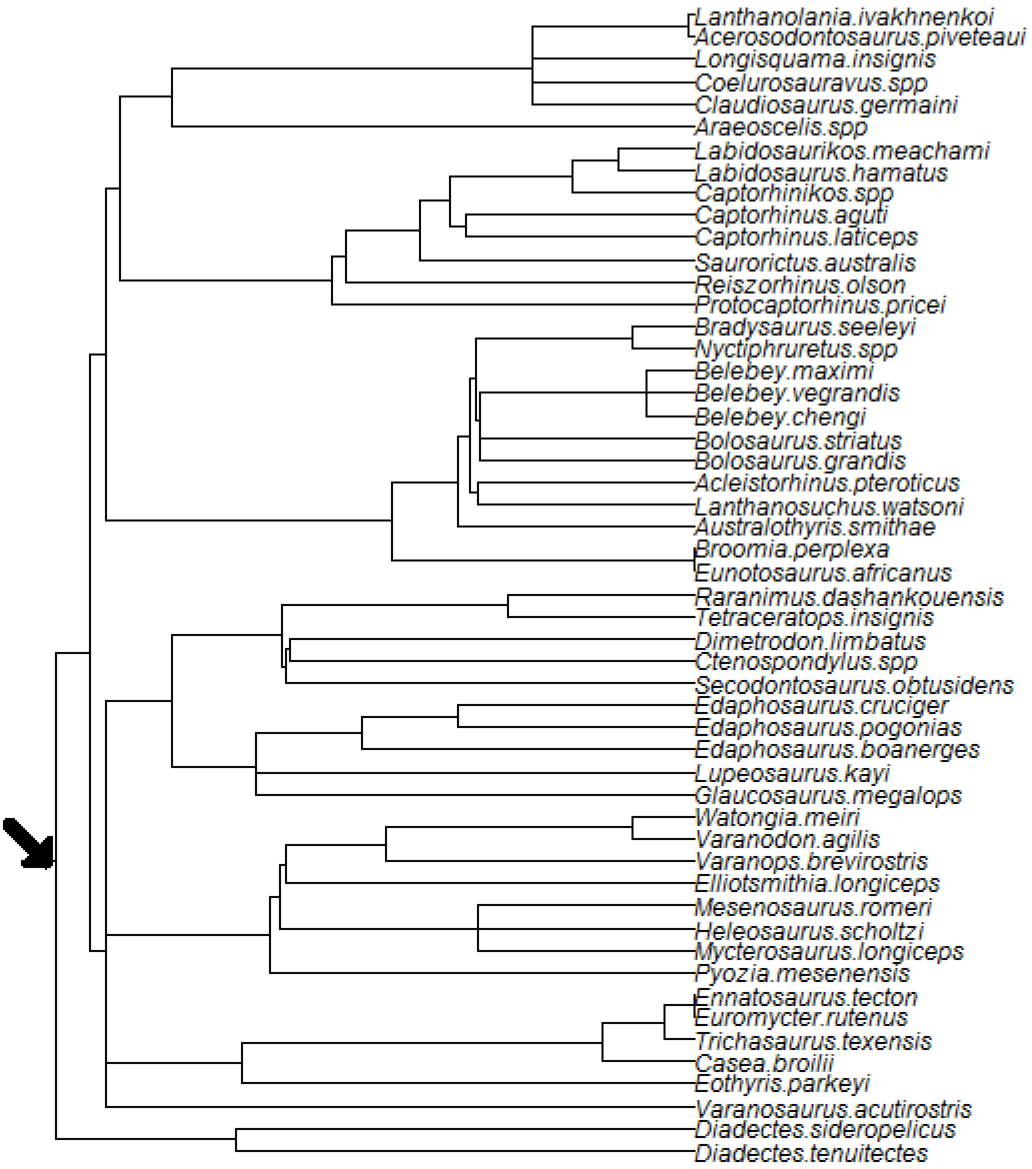

Supplementary Figure 4

Early Wordian

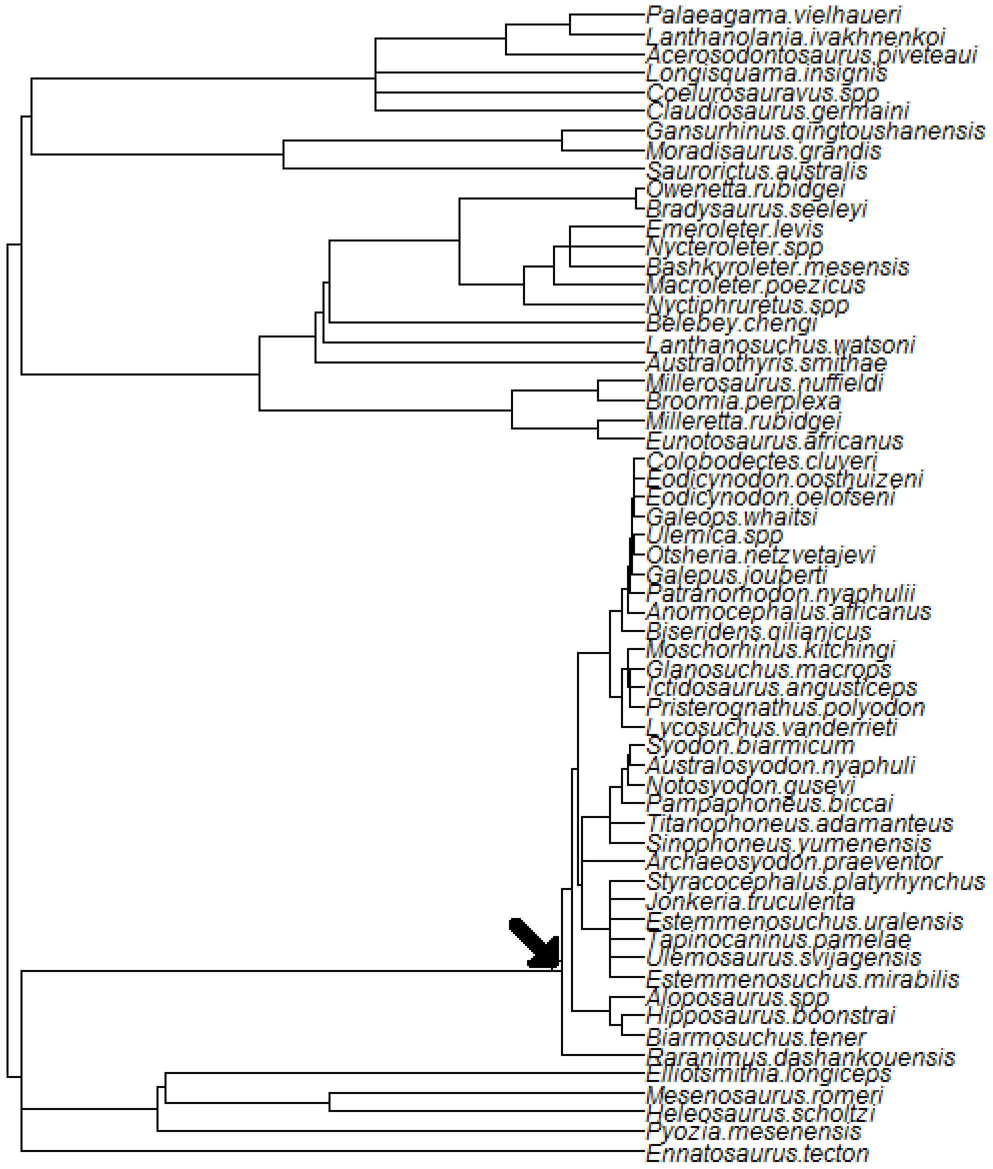

Supplementary Figure 5

Early Capitanian

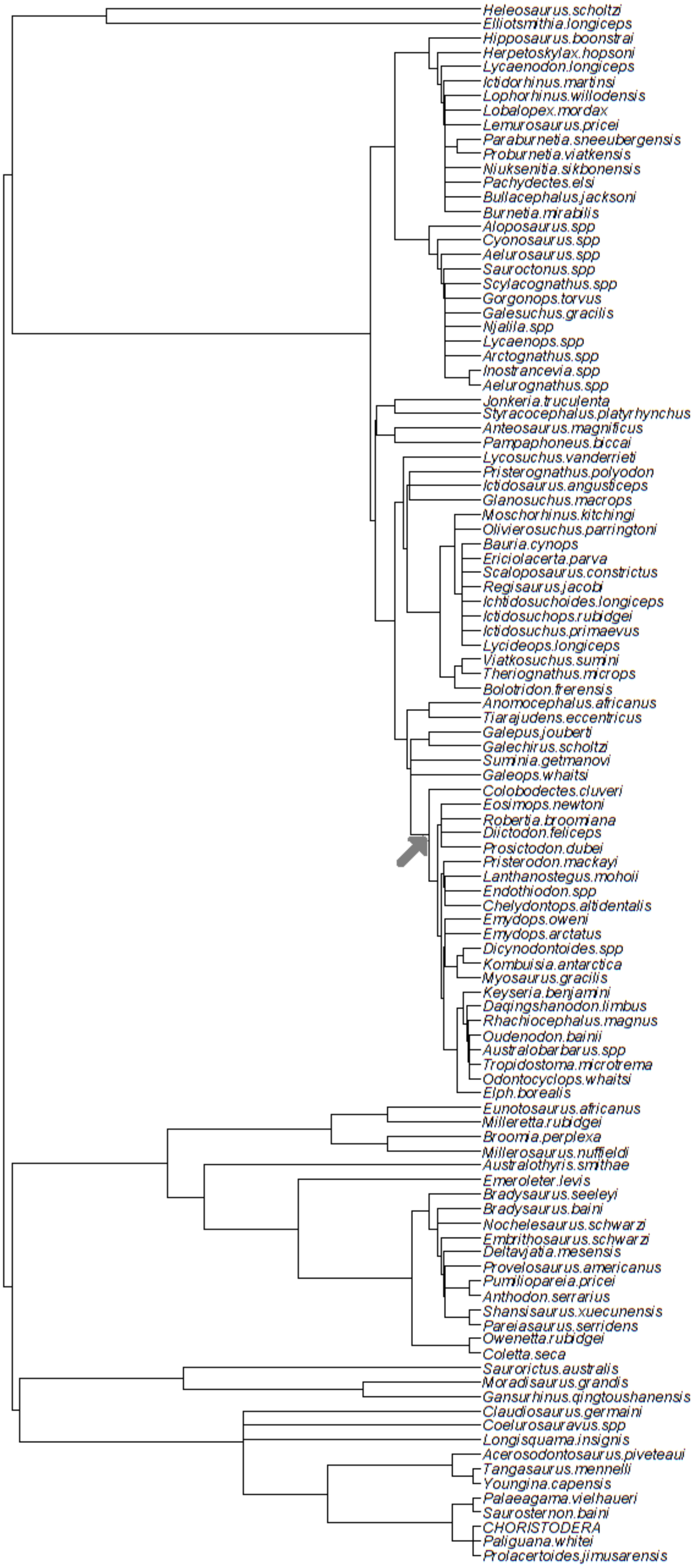

Supplementary Figure 6
Early Wuchiapingian

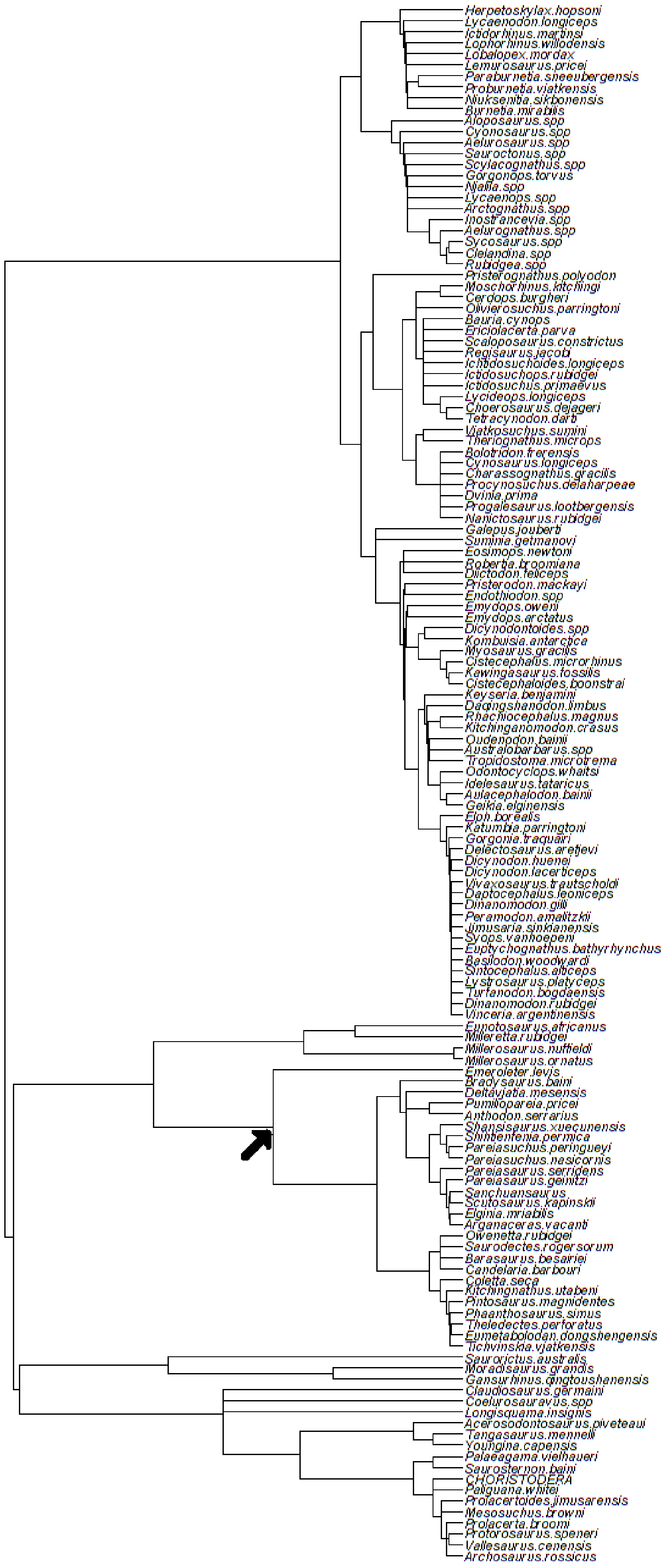

Supplementary Figure 7

Early Induan

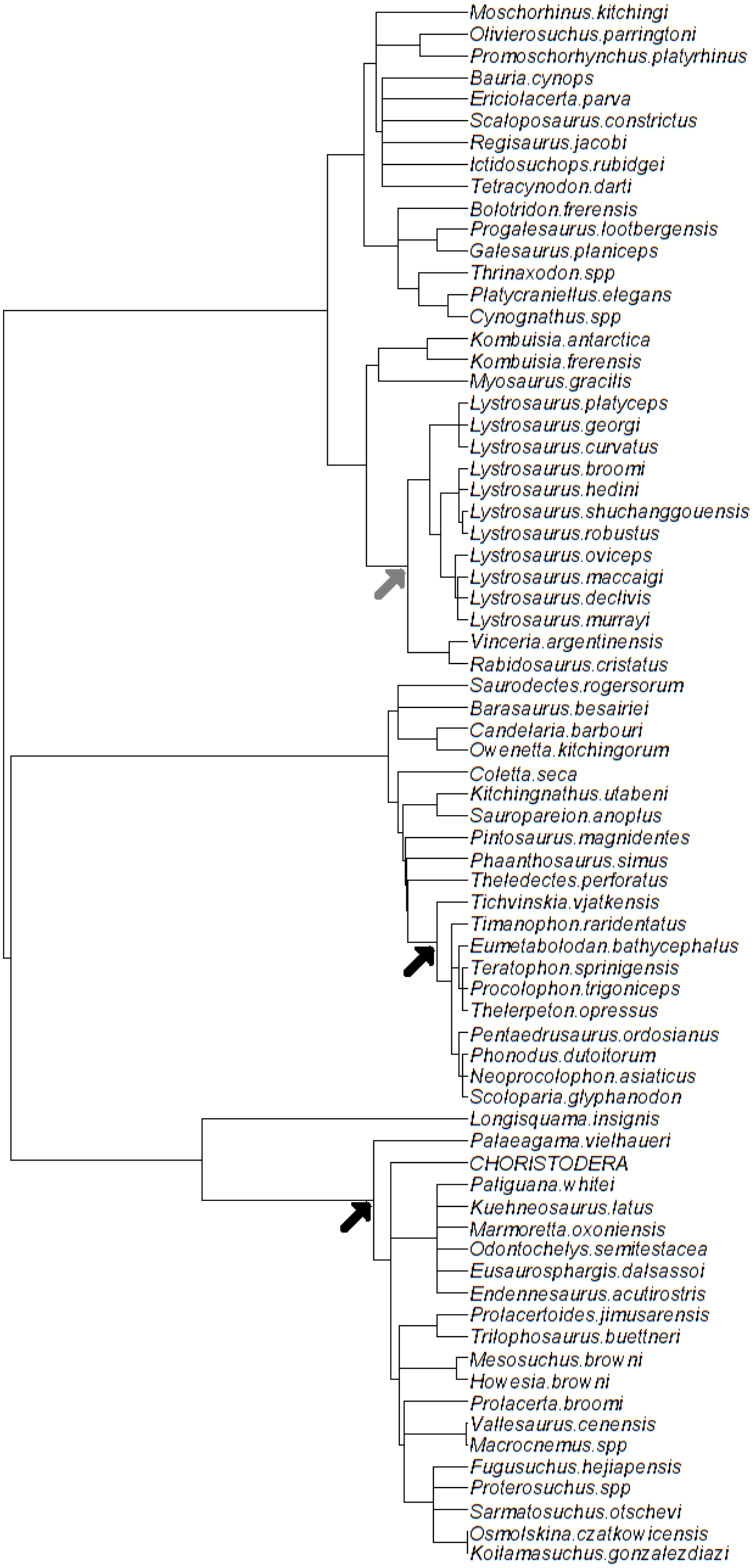

Supplementary Figure 8

Early Olenekian

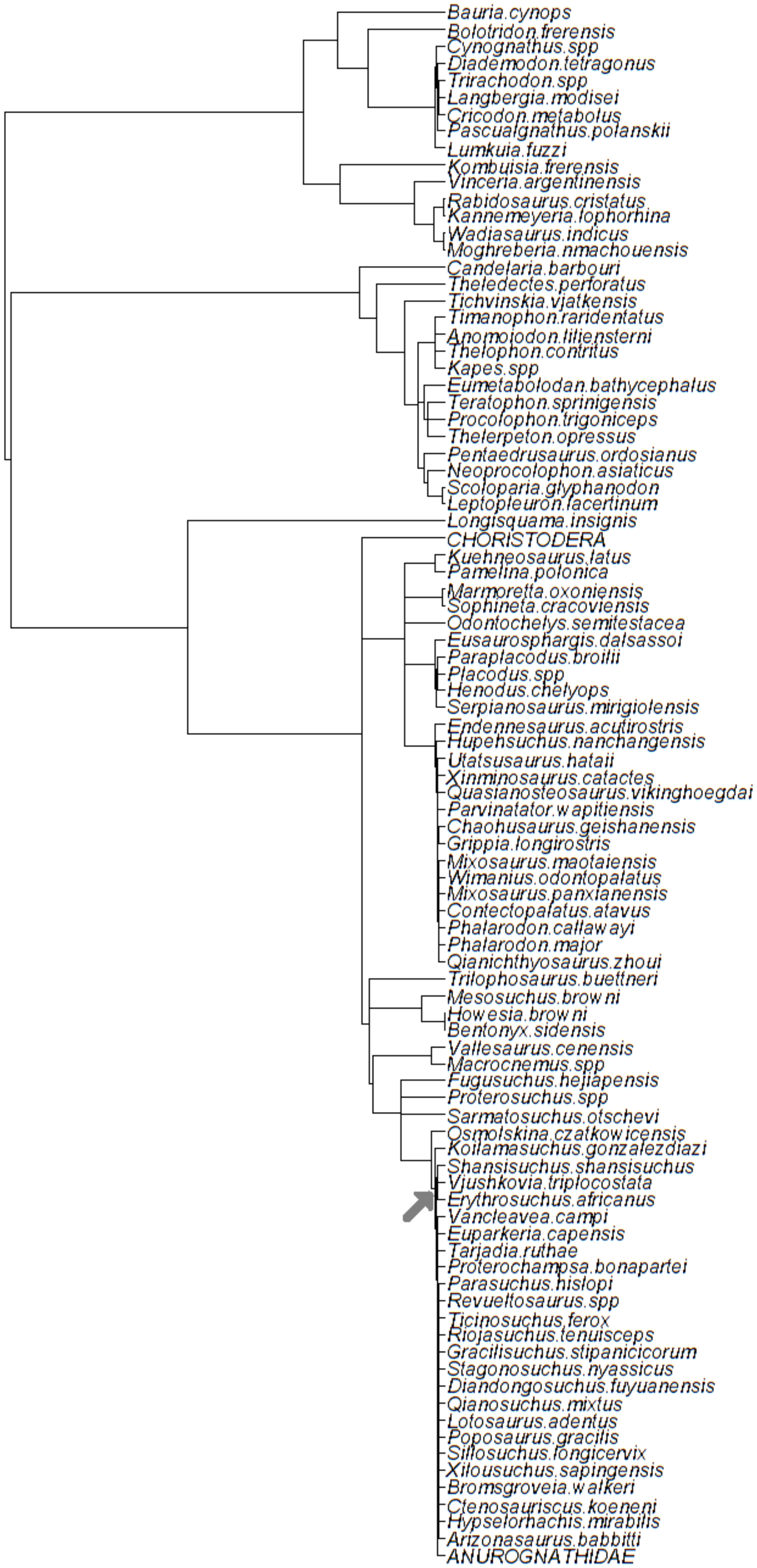

Supplementary Figure 9

Late Olenekian

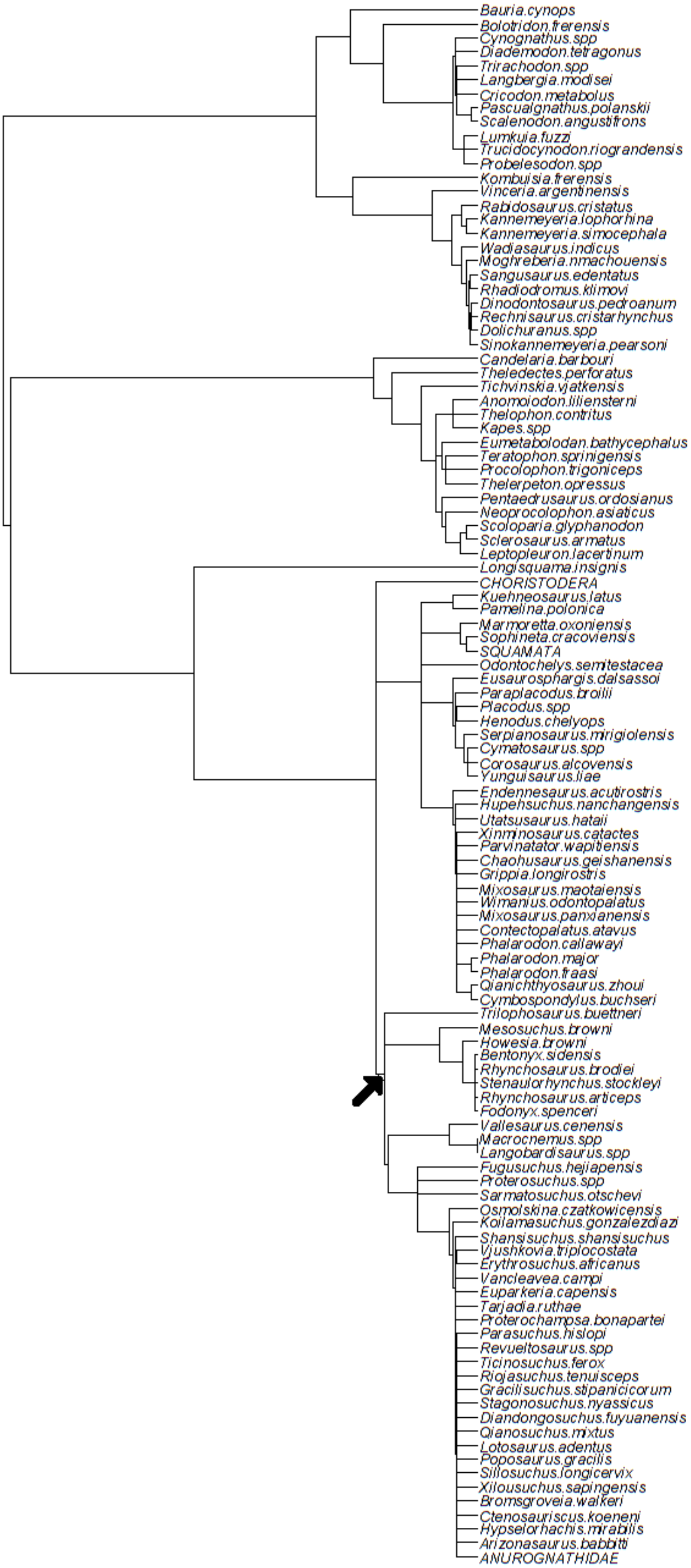

<u>Supplementary Figure 10</u>
Early Anisian

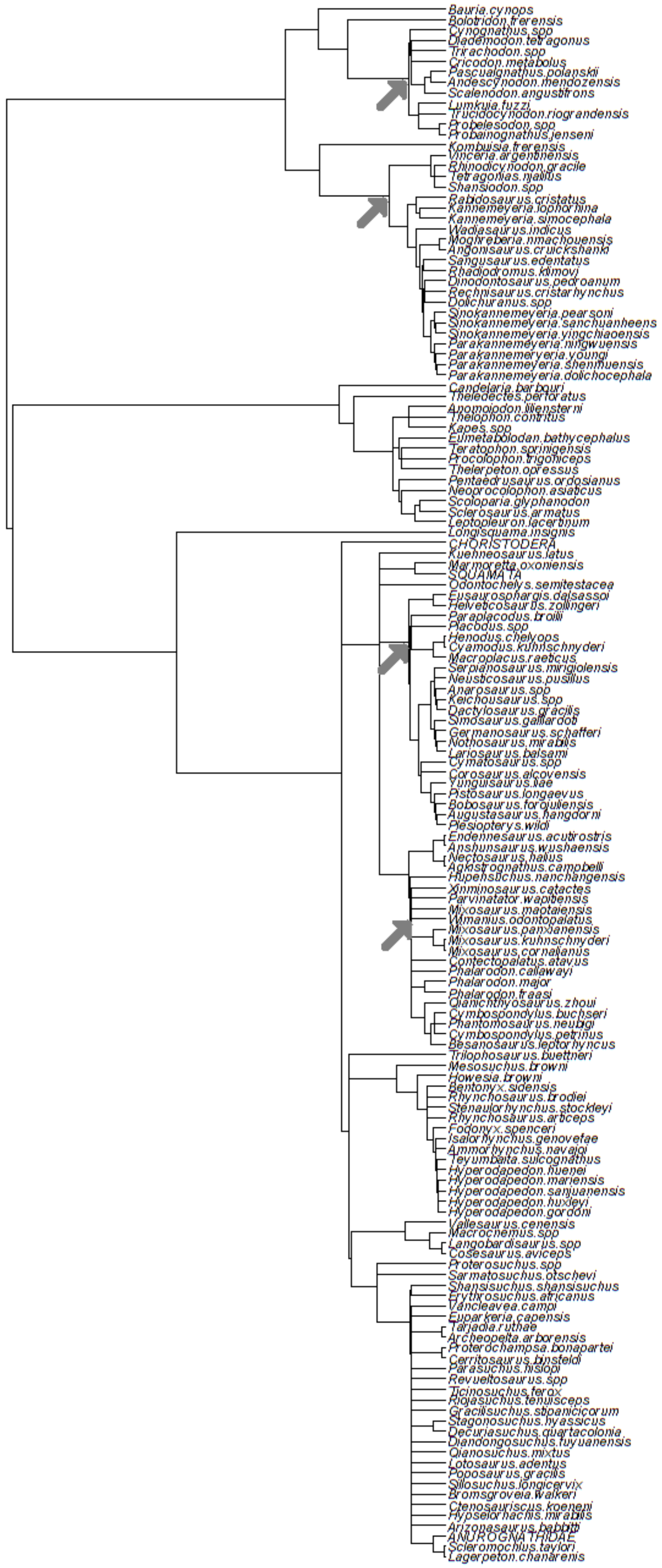

Supplementary Figure 11

Late Anisian

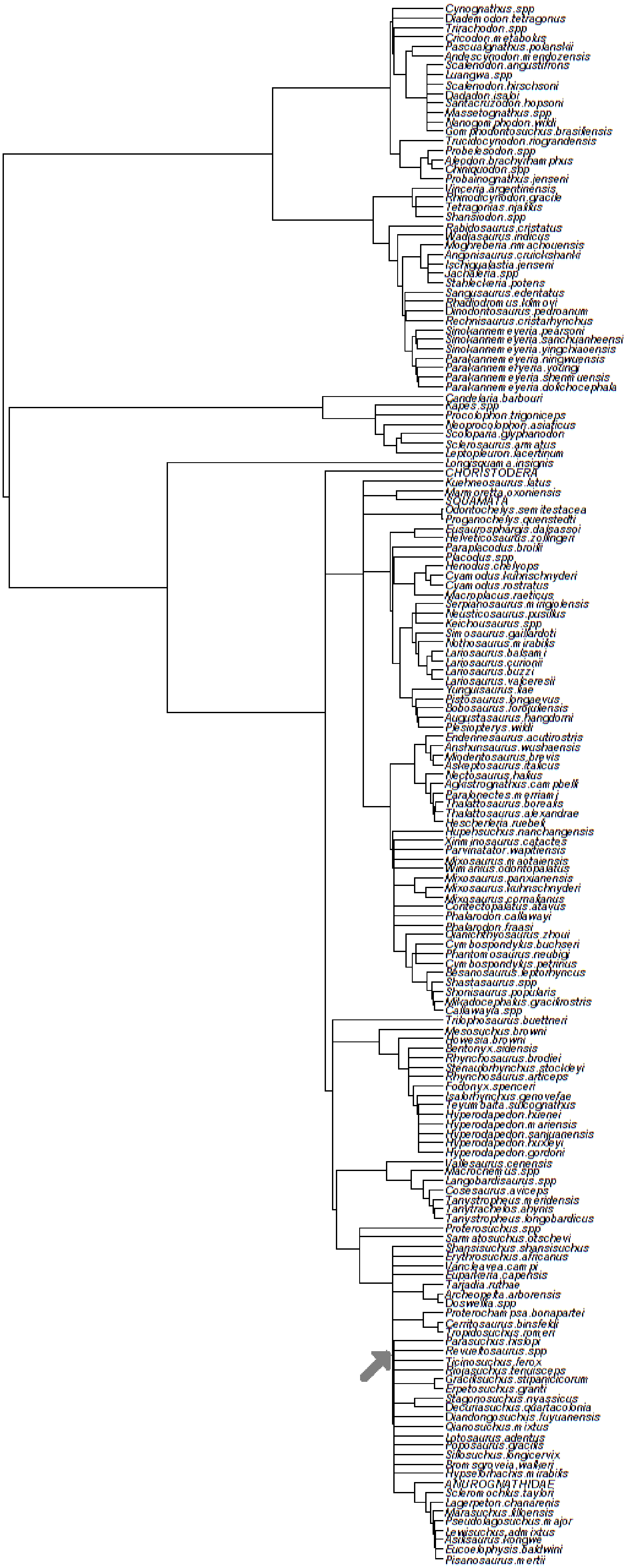

Supplementary Figure 12

Late Landinian

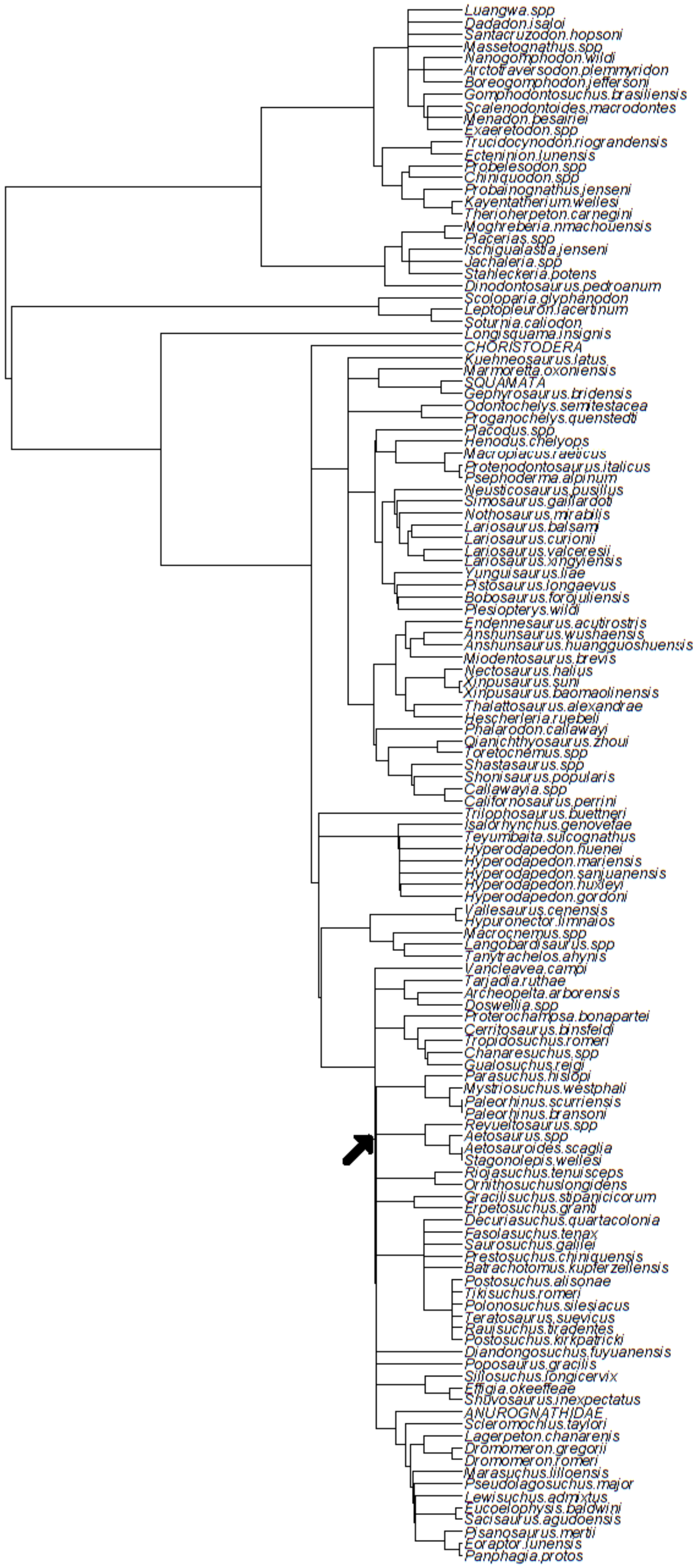

<u>Supplementary Figure 13</u>
Early Carnian

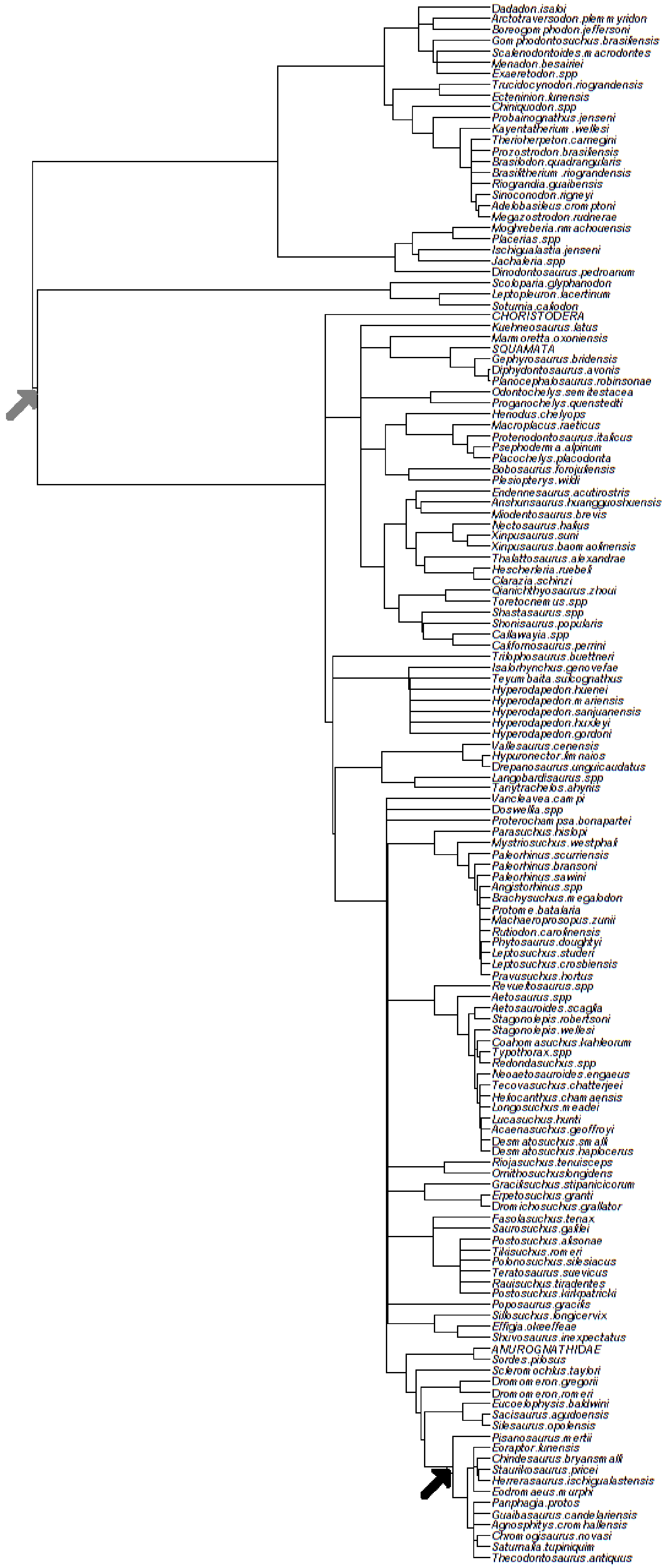

<u>Supplementary Figure 14</u>

Late Carnian

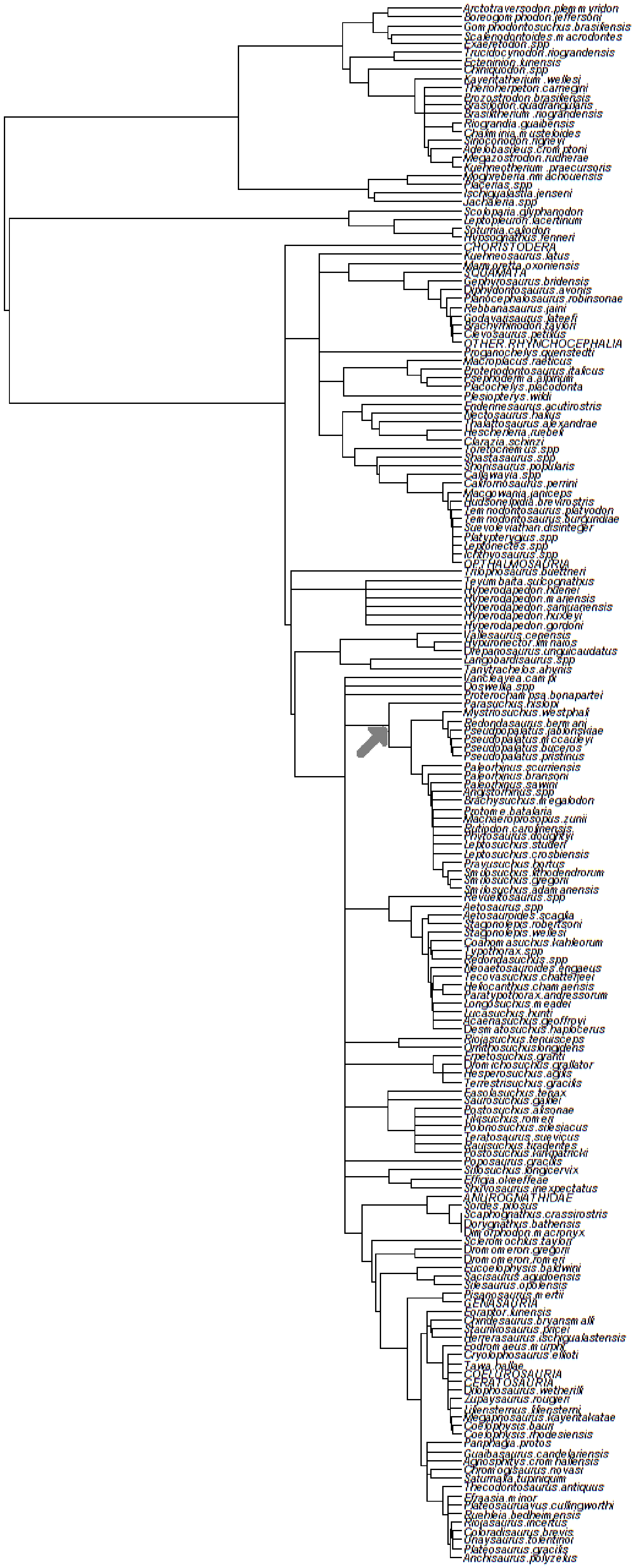

Supplementary Figure 15

Early Norian

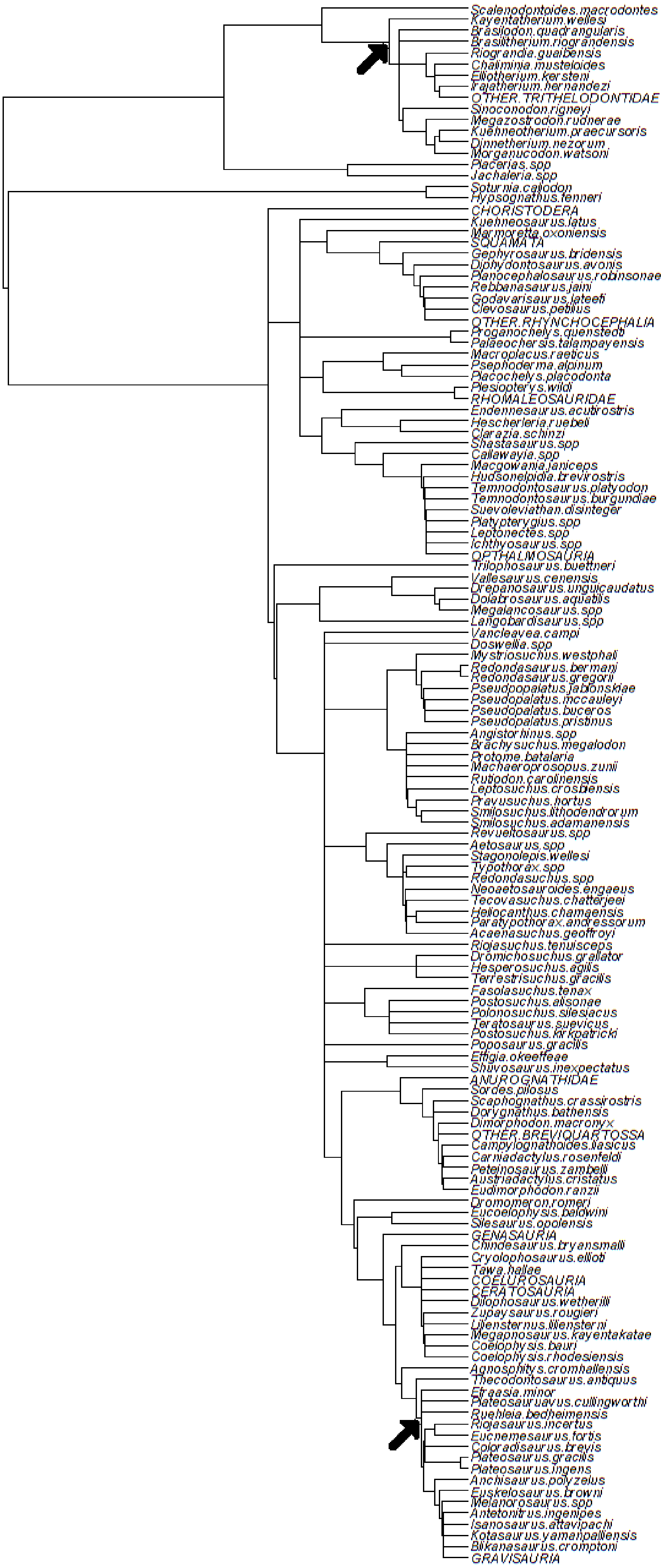

Supplementary Data 1
Tree files uploaded to SuperTree0.85b, including all source trees in phylip format:

Archosauromorpha:
tree Benton&Walker2002 =
(Stagonolepis.robertsoni,((Ornithosuchus,(Saurosuchus,(Batrachotomus,Prestosuchus))),(Gracilisuchus,(Postosuchus.kirkpatricki,(Erpetosuchus,(Sphenosuchus,CROCODYLIFORMES)))))));
tree Cark&Sues2002 =
((Dibothrosuchus,Sphenosuchus),Saltoposuchus,Hesperosuchus.agilis,Pseudohesperosuchus,Litargosuchus,Kayentasuchus,Terrestrisuchus,CROCODYLIFORMES);
tree Harrisetal2003 =
(Aetosaurus,((Aetosauroides,Stagonolepis.robertsoni),((Stagonolepis.wellesi,(Coahomasuchus,(Redondasuchus,Typothorax))),(Neoaetosauroides,(Paratypothorax,(Longosuchus,(Lucasuchus,(Desmatosuchus.haplocerus,Acaenasuchus))))))));
tree Nesbitt2003 =
(Marasuchus,(Aetosaurus,(CROCODYLIFORMES,(Saurosuchus,(Postosuchus.kirkpatricki,(Poposaurus.gracilis,Arizonasaurus,Shuvosaurus))))));
tree Olsonetal2000 =
(Gracilisuchus,(Postosuchus.kirkpatricki,(Erpetosuchus,(Sphenosuchus,CROCODYLIFORMES))));
tree Parker2007 =
(Revueltosaurus,(((Coahomasuchus,Neoaetosauroides,(Aetosaurus,Stagonolepis.robertsoni,Stagonolepis.wellesi,Aetosauroides)),((Tecovasuchus,(Heliocanthis,Paratypothorax)),(Typothorax,Redondasuchus))),(Longosuchus,(Lucasuchus,(Acaenasuchus,(Desmatosuchus.haplocerus,Desmatosuchus.smalli))))));
tree Parker&Irmis2006 =
(Ebrachosuchus.neukami,((Nicrosaurus.kapffi,Nicrosaurus.meyeri),(((Pseudopalatus.jablonskiae,(Pseudopalatus.mccauleyi,(Pseudopalatus.pristinus,Pseudopalatus.buceros))),(Redondasaurus.gregorii,Redondasaurus.bermani)),(Mystriosuchus.planirostris,Mystriosuchus.westphali))));
tree Parrish1993 =
(Erythrosuchus,(Euparkeria,(Proterochampsa,(Marasuchus,(Ornithosuchus,(Phytosaurus.doughtyi,((Saurosuchus,(Ticinosuchus,Prestosuchus)),(Turfanosuchus.dabanensis,Lewisuchus,(Aetosaurus,((Gracilisuchus,(Poposaurus.gracilis,CROCODYLIFORMES)),(Lotosaurus,(Fasolasuchus,(Rauisuchus,Batrachotomus)))))))))))));
tree Stocker2012 =
(Paleorhinus.scurriensis,(Paleorhinus.bransoni,(Paleorhinus.sawini,(Brachysuchus.megalodon,Angistorhinus,(Rutiodon.carolinensis,Machaeroprosopus.zunii,Protome.batalaria,(Phytosaurus.doughtyi,(Leptosuchus.crosbiensis,Leptosuchus.studeri),(((Smilosuchus.adamanensis,Smilosuchus.gregorii),Smilosuchus.lithodendrorum),(Pravusuchus.hortus(Pseudopalatus.mccauleyi,(Mystriosuchus.westphali,Pseudopalatus.pristinus))))))))));
tree Suesetal2003 =
(Stagonolepis.robertsoni,(Postosuchus.kirkpatricki,(Erpetosuchus,(Dromichosuchus,Saltoposuchus,Kayentasuchus,Pseudohesperosuchus,Hesperosuchus.agilis,Terrestrisuchus,Litargosuchus,(Sphenosuchus,(Dibothrosuchus,CROCODYLIFORMES))))));
tree Weinbaum&Hungurbuller2007 =
(Aetosaurus,((Arizonasaurus,Poposaurus.gracilis,(Shuvosaurus,Sillosuchus)),(Batrachotomus,(Saurosuchus,(CROCODYLIFORMES,(Teratosaurus,Postosuchus.kirkpatricki))))));
tree Andresetal2010 =
(Scleromochlus,(Preondactylus.buffarinii,((Eudimorphodon.ranzii,Austriadactylus.cristatus),(

Peteinosaurus.zambelli,(Dimorphodon.macronyx,(Campylognathoides.liasicus,(((OTHER.RHAMPHORHYNCHINAE,Dorygnathus.bathensis),Scaphognathus.crassirostris),(Sordes.pilosus,(ANUROGNATHIDAE,PTERODACTYLOIDEA)))))))));
tree Ezcurra2006 =
(Marasuchus,(Pseudolagosuchus,(Silesaurus,(Eucoelophysis.baldwini,((Lesothosaurus,(Scutellosaurus,Heterodontosaurus)),((Herrerasaurus,Staurikosaurus.pricei),((Saturnalia,(Thecodontosaurus.antiquus,Plateosaurus.engelhardti)),(Eoraptor.lunensis,((Dilophosaurus.wetherilli,(Liliensternus.liliensterni,(Megapnosaurus.kayentakatae,(Coelophysis.rhodesiensis,Coelophysis.bauri)))),(CERATOSAURIA,COELUROSAURIA))))))))));
tree Ezcurra2010 =
(CROCODYLIFORMES,(Marasuchus,(Silesaurus,(Heterodontosaurus,((Staurikosaurus.pricei,Herrerasaurus),(Chindesaurus,(Eoraptor.lunensis,COELUROSAURIA),((Angosphitys,Guaibasaurus,Panphagia,(Saturnalia,Chromogisaurus)),(Pantydraco,Thecodontosaurus.antiquus,(Plateosauruavus,(Ruehleia,((Unaysaurus,(Plateosaurus.gracilis,(Plateosaurus.engelhardti,Plateosaurus.ingens))),((Eucenemesaurus,Riojasaurus),((Massospondylus,(Coloradisaurus,(Lufengosaurus,Glacialisaurus))),(Jingshanosaurus,(Anchisaurus,(Yunnanosaurus,(Melanorosaurus,EUSAUROPODA)))))))))))))))))));
tree Ezcurra&Novas2007 =
((Lesothosaurus,(Scutellosaurus,Heterodontosaurus)),((Saturnalia,(Thecodontosaurus.antiquus,Plateosaurus.engelhardti)),(Eoraptor.lunensis,(Herrerasaurus,((Dilophosaurus.wetherilli,(Liliensternus.liliensterni,(Zupaysaurus,Coelophysis.bauri,Coelophysis.rhodesiensis,Megapnosaurus.kayentakatae,Gojirasaurus,Segisaurus,Procompsognathus))),(CERATOSAURIA,COELUROSAURIA))))));
tree Lu&Ji2006 =
(ANUROGNATHIDAE,((Preondactylus.buffarinii,Dimorphodon.macronyx),(Sordes.pilosus,Dorygnathus.bathensis,Scaphognathus.crassirostris,((Campylognathoides.liasicus,Carniadactylus.rosenfeldi),(OTHER.RHAMPHORHYNCHINAE,PTERODACTYLOIDEA)))));
tree Martinez&Alcober2008 =
(Silesaurus,(Pisanosaurus,Heterodontosaurus),((Herrerasaurus,Staurikosaurus.pricei),(Eoraptor.lunensis,(Guaibasaurus,COELUROSAURIA,(Panphagia,(Saturnalia,EUSAUROPODA))))))
;
tree Martinezetal2011 =
(Marasuchus,(Silesaurus,((Lesothosaurus,(OTHER.ORNITHOPODA,Heterodontosaurus)),((EUSAUROPODA,(Eoraptor.lunensis,Panphagia,Saturnalia)),((Herrerasaurus,Staurikosaurus.pricei),(Eodromaeus,(CERATOSAURIA,(Tawa,(Dilophosaurus.wetherilli,(Megapnosaurus.kayentakatae,(Coelophysis.rhodesiensis,Coelophysis.bauri)))))))))));
tree Nesbittetal2009 =
((Leptosuchus.crosbiensis,(Aetosaurus,(Riojasuchus,(Effigia,(Postosuchus.kirkpatricki,(Hesperosuchus.agilis,CROCODYLIFORMES)))))),((Dimorphodon.macronyx,Eudimorphodon.ranzii),((Lagerpeton,(Dromomeron.romeri,Dromomeron.gregorii)),(Marasuchus,((Silesaurus,Eucoelophysis.baldwini),(((Heterodontosaurus,Lesothosaurus,Scutellosaurus),Pisanosaurus),((Herrerasaurus,Chindesaurus),((Saturnalia,(Plateosaurus.engelhardti,Efraasia)),(Coelophysis.bauri,COELUROSAURIA)))))))));
tree Nesbittetal2009a =
(Heterodontosaurus,(EUSAUROPODA,((Staurikosaurus.pricei,(Herrerasaurus,Chindesaurus)),(Tawa,((Coelophysis.bauri,Megapnosaurus.kayentakatae),(Liliensternus.liliensterni,(Zupaysaurus,(Crylophosaurus,(Dilophosaurus.wetherilli,COELUROSAURIA)))))))));
tree Sereno1999 =
((Pisanosaurus,(Lesothosaurus,((Scutellosaurus,OTHER.THYREOPHORA),(MARGINOCEPHALIA,(OTHER.ORNITHOPODA,Heterodontosaurus))))),(((Riojasaurus,((Yunnanosaurus,Massospondylus),(Lufengosaurus,(Plateosaurus.gracilis,Plateosaurus.engelhardti)))),(Vulcan

odon,EUSAUROPODA)),(Eoraptor.lunensis,(Herrerasaurus,((CERATOSAURIA,(Dilophosaurus.wetherilli,(Liliensternus.liliensterni,Coelophysis.bauri))),COELUROSAURIA)))));
tree Wangetal2005 =
(ANUROGNATHIDAE,(Sordes.pilosus,(Preondactylus.buffarinii,Scaphognathus.crassirostris,(Dorygnathus.bathensis,(Dimorphodon.macronyx,(Peteinosaurus.zambelli,Carniadactylus.rosenfeldi),((Campylognathoides.liasicus,Eudimorphodon.ranzii),(OTHER.RHAMPHORHYNCHINAE,PTERODACTYLOIDEA)))))));
tree Yates2003 =
(Heterodontosaurus,(Herrerasaurus,(COELUROSAURIA,(Saturnalia,((Thecodontosaurus.antiquus,Pantydraco.caducus),(Efraasia,(Plateosaurus.engelhardti,(Riojasaurus,Anchisaurus,(Coloradisaurus,Lufengosaurus,(Massospondylus,Yunnanosaurus),(Euskelosaurus,(Melanorosaurus,(Blikanasaurus,(Vulcanodon,EUSAUROPODA)))))))))))));
tree Yates2003Bentonetal =
(Herrerasaurus,(EUSAUROPODA,Coloradisaurus,Thecodontosaurus.antiquus,Plateosaurus.gracilis,Massospondylus,Plateosaurus.engelhardti,Anchisaurus,Melanorosaurus,Euskelosaurus,Vulcanodon));
tree Yates&Kitching2003 =
(Saturnalia,(Thecodontosaurus.antiquus,(Efraasia,((Riojasaurus,(Plateosaurus.engelhardti,(Coloradisaurus,(Massospondylus,Lufengosaurus)))),(Anchisaurus,(Melanorosaurus,(Antetonitrus,(Isanosaurus,(Kotosaurus,(Vulcanodon,EUSAUROPODA))))))))));
tree Benton1999 =
(Proterochampsa,((Phytosaurus.doughtyi,(Stagonolepis.robertsoni,(CROCODYLIFORMES,(Ornithosuchus,(Prestosuchus,Postosuchus.kirkpatricki))))),(Scleromochlus,(Dimorphodon.macronyx,(Lagerpeton,(Marasuchus,(Heterodontosaurus,(Plateosaurus.engelhardti,(Herrerasaurus,COELUROSAURIA)))))))));
tree Benton&Allen1997 =
((Trilophosaurus,Prolacertoides),((Boreopricea,Prolacerta),(Protorosaurus,(Malerisaurus.langstoni,(Langobardisaurus,(Macrocnemus,(Cosesaurus,(Tanystropheus.meridensis,(Tanystropheus.longobardicus,Tanytrachelos)))))))));
tree Bennett2012revised =
(Rhynchosaurus.articeps,(Prolacerta,(Proterosuchus,(Erythrosuchus,(Proterochampsa,(Euparkeria,(Phytosaurus.doughtyi,CROCODYLIFORMES,(Ornithosuchus,(Scleromochlus,(Herrerasaurus,Marasuchus)))))),PTERODACTYLOIDEA))));
tree Bennett2012updated =
(Rhynchosaurus.articeps,(Prolacerta,(Tanystropheus.longobardicus,(Megalancosaurus,Vallesaurus),(Proterosuchus,((ANUROGNATHIDAE,(Peteinosaurus.zambelli,Eudimorphodon.ranzii)),(Erythrosuchus,(Proterochampsa,(Euparkeria,(Phytosaurus.doughtyi,CROCODYLIFORMES,(Ornithosuchus,(Scleromochlus,(Marasuchus,Herrerasaurus)))))))))))));
tree Butleretal2011BrusatteB =
(Euparkeria,(Proterochampsa,(((Scleromochlus,Dimorphodon.macronyx),((Lagerpeton,Dromomeron.romeri),(Marasuchus,(Pseudolagosuchus,((Lewisuchus,(Silesaurus,Eucoelophysis.baldwini,Sacisaurus)),(Heterodontosaurus,COELUROSAURIA)))))),(Revueltosaurus,Phytosaurus.doughtyi,Aetosaurus,(Gracilisuchus,(Erpetosuchus,CROCODYLIFORMES)),((Ticinosuchus,Qianosuchus,(Stagonosuchus,((Prestosuchus,Batrachotomus),(Fasolasuchus,Saurosuchus,(Tikisuchus,Rauisuchus,(Polonosuchus.silesiacus,Postosuchus.kirkpatricki)))))),(Ornithosuchus,(Lotosaurus,((Sillosuchus,Poposaurus.gracilis,(Shuvosaurus,Effigia)),(Arizonasaurus,Hypselorhachis,Ctenosauriscus,Bromsgroveia)))))))));
tree Dilkes1995 =
(Mesosuchus,(Howesia.browni,(Rhynchosaurus.articeps,(Stenaulorhynchus.stockleyi,(Hyperodapedon.sanjuanensis,Hyperodapedon.gordoni)))));

tree Dilkes&Arcucci2012 =
(Erythrosuchus,((Doswellia,Vancleavea),((Proterochampsa,(Cerritosaurus,(Tropidosuchus,(Gualosuchus,Chanaresuchus)))),(Euparkeria,(Parasuchus,((Riojasuchus,Aetosaurus),(Silesaurus,Herrerasaurus)))))));
tree Desojoetal2011 =
(Prolacerta,(Proterosuchus,(Vancleavea,(Erythrosuchus,(Euparkeria,(Chanaresuchus,(Turfanosuchus.dabanensis,(Tarjadia,(Doswellia,Archeopelta)),(Yonghesuchus,((Scleromochlus,Marasuchus),(Qianosuchus,Gracilisuchus,(Parasuchus,(Chilenosuchus,Stagonolepis.robertsoni))))))))))));
tree Ezcurraetal2010 =
(Prolacerta,(Proterosuchus,(Sarmatosuchus,Fugusuchus,(Osmolskina,(Koilamasuchus,((Shansisuchus,(Vjushkovia.triplocostata,Erythrosuchus)),(Euparkeria,(Chanaresuchus,((Vancleavea,Doswellia),((Turfanosuchus.dabanensis,(Qianosuchus,(Parasuchus,(Stagonolepis.robertsoni,(Saurosuchus,(Terrestrisuchus,Gracilisuchus)))))),((Dimorphodon.macronyx,Scleromochlus),(Lagerpeton,(Marasuchus,(Silesaurus,(Eoraptor.lunensis,Herrerasaurus)))))))))))))));
tree Francaetal2012 =
(Euparkeria,(Proterochampsa,(((Scleromochlus,Dimorphodon.macronyx),((Lagerpeton,Dromomeron.romeri),(Marasuchus,(Pseudolagosuchus,((Lewisuchus,(Sacisaurus,Eucoelophysis.baldwini,Silesaurus)),(Heterodontosaurus,COELUROSAURIA)))))),((Paleorhinus.bransoni,(Mystriosuchus.planirostris,Rutiodon.carolinensis)),(((Aetosaurus,(Stagonolepis.robertsoni,Desmatosuchus.haplocerus)),(Gracilisuchus,(Erpetosuchus,(Postosuchus.kirkpatricki,Sphenosuchus,Terrestrisuchus)))),(Ticinosuchus,Qianosuchus,(Riojasuchus,Ornithosuchus),(Sillosuchus,Poposaurus.gracilis,Lotosaurus,Bromsgroveia,Arizonasaurus,(Shuvosaurus,Effigia)),(Tikisuchus,Saurosuchus,Rauisuchus,(Teratosaurus,Postosuchus.kirkpatricki),(Decuriasuchus,(Prestosuchus,Batrachotomus)))))))));
tree Gower&Sennikov1997 =
((Proterosuchus,(Fugusuchus,Sarmatosuchus)),((Vjushkovia.triplocostata,Erythrosuchus),(Euparkeria,Sphenosuchus)));
tree Hone&Benton2008 =
(Trilophosaurus,(Prolacerta,(Mesosuchus,(Howesia.browni,(Stenaulorhynchus.stockleyi,(Rhynchosaurus.articeps,Rhynchosaurus.brodiei,(Fodonyx.spenceri,(Ammorhucnhus.navajoi,(Teyumbaita.sulcognathus,Hyperodapedon.gordoni,Hyperodapedon.sanjuanensis,Hyperodapedon.mariensis,Hyperodapedon.gordoni,Hyperodapedon.huxleyi)))))))));
tree Jalil1997 =
(Youngina,((Gephyrosaurus,SQUAMATA),(CHORISTODERA,(Rhynchosaurus.articeps,(Trilophosaurus,(Proterosuchus,(Prolacerta,((Macrocnemus,(Langobardisaurus,(Boreopricea,(Cosesaurus,(Tanystropheus.longobardicus,Tanytrachelos))))),(Malerisaurus.langstoni,Jesairosaurus)))))))));
tree Langer&Schultz2000 =
(Fodonyx.spenceri,(Teyumbaita.sulcognathus,(Hyperodapedon.huenei,(Hyperodapedon.mariensis,(Hyperodapedon.gordoni,Hyperodapedon.huxleyi),Hyperodapedon.sanjuanensis))));
tree Montefeltroetal2013 =
(Howesia.browni,(Rhynchosaurus.articeps,(Stenaulorhynchus.stockleyi,Bentonyx.sidensis,(Fodonyx.spenceri,(Isalorhynchus.genovefae,(Teyumbaita.sulcognathus,Hyperodapedon.gordoni))))));
tree Sereno1991 =
(Erythrosuchus,(Euparkeria,(Proterochampsa,((Phytosaurus.doughtyi,((Riojasuchus,Ornithosuchus),CROCODYLIFORMES)),(PTERODACTYLOIDEA,Herrerasaurus,Scleromochlus)))));
tree Renesto&Binelli2006 =
(Petrolacosaurus,((Coelursauravus,Longisquama),(Youngina,(Gephyrosaurus,(Mesosuchus,((

Prolacerta,(Macrocnemus,Langobardisaurus)),((Proterosuchus,(Euparkeria,(Ornithosuchus,COELUROSAURIA))),(Eudimorphodon.ranzii,(Vallesaurus,(Hypuronector,(Drepanosaurus,(Dolabrosaurus,Megalancosaurus)))))))))));
tree minitree1 =
(Prolacerta,((Archosaurus,Proterosuchus),(Erythrosuchus,(Vancleavea,((Chanaresuchus,Tropidosuchus),(Euparkeria,((Parasuchus,(Pseudopalatus.pristinus,Smilosuchus.gregorii)),(((Dimorphodon.macronyx,Eudimorphodon.ranzii),((Lagerpeton,(Dromomeron.romeri,Dromomeron.gregorii)),(Marasuchus,((Lewisuchus,(Asilisaurus,(Eucoelophysis.baldwini,(Silesaurus,Sacisaurus)))),((Eocursor,Lesothosaurus,Scutellosaurus,(Heterodontosaurus,Pisanosaurus)),((Saturnalia,(Plateosaurus.engelhardti,Efraasia)),(Herrerasaurus,(Staurikosaurus.pricei,(Eoraptor.lunensis,(Tawa,(Coelophysis.bauri,(COELUROSAURIA,Dilophosaurus.wetherilli))))))))))))),((Ornithosuchus,Riojasuchus),(Turfanosuchus.dabanensis,Gracilisuchus,(Revueltosaurus,(Aetosaurus,Longosuchus,Stagonolepis.robertsoni)),(Ticinosuchus,(Diandongosuchus,(Qianosuchus,((Ctenosauriscus,Hypselorhachis,Xilosuchus,Arizonasaurus),(Poposaurus.gracilis,(Lotosaurus,(Sillosuchus,(Effigia,Shuvosaurus))))))),(Prestosuchus,(Saurosuchus,(Batrachotomus,(Fasolasuchus,((Rauisuchus,(Postosuchus.alisonae,Postosuchus.kirkpatricki,Polonosuchus.silesiacus)),(Dromichosuchus,Hesperosuchus.agilis,(Sphenosuchus,(Terrestrisuchus,Dibothrosuchus,(Litargosuchus,(CROCODYLIFORMES,Kayentasuchus))))))))))))))))))))));
tree minitree2 =
(Youngina,(CHORISTODERA,((SQUAMATA,Gephyrosaurus),((Protorosaurus,((Drepanosaurus,Megalancosaurus),(Macrocnemus,(Langobardisaurus,Tanystropheus.longobardicus)))),(Trilophosaurus,((Prolacerta,(Euparkeria,Proterosuchus)),(Mesosuchus,(Howesia.browni,(Rhynchosaurus.articeps,(Stenaulorhynchus.stockleyi,(Hyperodapedon.gordoni,Hyperodapedon.sanjuanensis)))))))))));

<u>Basal + Sauropterygia + Parareptilia</u>
tree Reiszetal2011 =
(Paleothyris,(Thuringothyris,(Concordia,(Rhiodenticulatus,((Romeria.texana,Romeria.prima),(Protocaptorhinus,(Saurorictus,((Captorhinus.laticeps,(Captorhinus.magnus,Captorhinus.aguti)),(Captorhinikos,(Labidosaurus,(Labidosaurikos,(Moradisaurus,(Rothianiscus,Gansurhinus))))))))))))));
tree Sumidaetal2010 =
(Thuringothyris,(Concordia,(Romeria.texana,(Protocaptorhinus,(Reiszorhinus,(Rhiodenticulatus,(Saurorictus,((Captorhinus.laticeps,Captorhinus.aguti),(Labidosaurus,Labidosaurikos)))))))));
tree minisupertree7 =
(Casea.broilii,(MESOSAURIDAE,(Procolophon.trigoniceps,Milleretta.rubidgei),(Coelostegus,((Brouffia,(Paleothyris,(Hylonomus,((Araeoscelis,Petrolacosaurus),(Cephalerpeton,(Anthracodromeus,Protorothyris.archeri)))))),(Thuringothyris,(Concordia,(Romeria.texana,(Protocaptorhinus,Rhiodenticulatus,(Saurorictus,((Labidosaurikos,Labidosaurus),(Captorhinus.aguti,Captorhinus.laticeps)))))))))));
tree minisupertree8 =
(MESOSAURIDAE,((Ophiacodon.retroversus,(Casea.broilii,(Ophiacodon.mirus,(Edaphosaurus.cruciger,(Sphenacodon,(Biarmosuchus.tener,(Anteosaurus.magnificus,(Gorgonops.torvus,(Dicynodon.lacerticeps,(Lychosuchus.vanderrieti,(Procynosuchus.delaharpeae,(Thrinaxodon,(Diademodon,(Exaeretodon,(Kayentatherium,(Morganucodon,PLACENTA)))))))))))))))),((Milleretta.rubidgei,(Macroleter.poezicus,(Lanthanosuchus,Acleistorhinus),((Owenetta.rubidgei,Procolophon.trigoniceps),(Bradysaurus.baini,Bradysaurus.seeleyi,(Anthodon.serrarius,(Pareiasaurus.serridens,Scutosaurus.kapinskii)))))),(Captorhinus.aguti,(Paleothyris,(Araeoscelis,(Claudiosaurus,(Youngina,((KUEHNEOSAURIDAE,(((Planocephalosaurus,SQUAMATA),(Odontochelys.semitestacea,OTHER.CHELONIA,Proganochelys.quenstedti)),(Simosaurus,(Cyamodu

s.rostratus,Placodus)))),(CHORISTODERA,(Hyperodapedon,Trilophosaurus.buettneri),Prolacerta,(Protorosaurus,(Proterosuchus,(Erythrosuchus,(Proterochampsa,(DINOSAURIA,(CROCODYLIA,(Protosuchus.richardsoni,(Paratypothorax,((Rutiodon,Pseudopalatus),(Desmatosuchus.haplocercus,(Coahomasuchus.kahleorum,Typothorax.coccinarum))))))))))))))))))));
tree DeBraga&Reisz1995 =
(Paleothyris,((Spinoaequalis,(Petrolacosaurus,Araeoscelis)),(Apsisaurus,(Hovasaurus,(Acerosodontosaurus,Youngina)))));
tree Laurin&Reisz1997 = (Casea.broilii,(Captorhinus.aguti,Procolophon.trigoniceps));
tree Lee2001 =
((Casea.broilii,(Ophiacodon.mirus,(Edaphosaurus.cruciger,(Sphenacodon,(Gorgonops.torvus,Procynosuchus.delaharpeae))))),((Milleretta.rubidgei,((Lanthanosuchus,Acleistorhinus),(Nycteroleter,(Procolophon.trigoniceps,(Pareiasaurus.serridens,Proganochelys.quenstedti))))),(Captorhinus.aguti,(Protorothyris.archeri,(Araeoscelis,(Claudiosaurus,(Youngina,(((Placodus,Simosaurus),(KUEHNEOSAURIDAE,(Planocephalosaurus,SQUAMATA))),(CHORISTODERA,(Prolacerta,(DINOSAURIA,(Trilophosaurus.buettneri,Hyperodapedon)))))))))))));
tree Modesto&Reisz2002 =
(Paleothyris,((Petrolacosaurus,Araeoscelis),(Coelurosauravus,(Apsisaurus,((Acerosodontosaurus,Youngina),(Lanthanolania,Planocephalosaurus,SQUAMATA,(Prolacerta,(Trilophosaurus.buettneri,Hyperodapedon))))))));
tree Muller2003 =
(PLACENTA,(Procolophon.trigoniceps,(Captorhinus.aguti,(Araeoscelis,(Apsisaurus,(Youngina,(Claudiosaurus,(KUEHNEOSAURIDAE,(Coelurosauravus,((Thalattosaurus.alexandrae,ICHTHYOPTERYGIA),((Tanystropheus,(Trilophosaurus.buettneri,(Prolacerta,(Hyperodapedon,DINOSAURIA)))),(CHORISTODERA,((Simosaurus,Placodus),(Proganochelys.quenstedti,(SQUAMATA,Planocephalosaurus)))))))))))))));
tree Rieppel&DeBraga =
(PLACENTA,(Procolophon.trigoniceps,(Captorhinus.aguti,(Paleothyris,(Araeoscelis,(Claudiosaurus,(Youngina,(DINOSAURIA,(SQUAMATA,(Proganochelys.quenstedti,(Placodus,Simosaurus)))))))))));
tree minisupertree5 =
(Bunostegos.akokanensis,Lanthanosuchus,Sclerosaurus.armatus,Procolophon.trigoniceps,Barasaurus,Owenetta.rubidgei,(Bradysaurus.seeleyi,(Nochelesaurus.alexanderi,Bradysaurus.baini,(Embrithosaurus.schwarzi,(Deltavjatia.mesensis,((Nanoparia.luckhoffi,(Provelosaurus.americanus,(Anthodon.serrarius,(Proganochelys.quenstedti,Pumiliopareia.pricei)))),((Shansisaurus.xuecunensis,(Shihtienfenia.permica,(Pareiasuchus.nasicornis,Pareiasuchus.peringueyi))),(Pareiasaurus.serridens,(Parasaurus.geinitzi,((Arganaceras.vacanti,Elginia.mirabilis),(Scutosaurus.kapinskii,Sanchuansaurus)))))))))));
tree minisupertree6 =
(PLACENTA,((Captorhinus.aguti,(Paleothyris,(Youngina,Araeoscelis))),(MESOSAURIDAE,(Milleretta.rubidgei,(Eunotosaurus,(Proganochelys.quenstedti,Odontochelys.semitestacea)),(Microleter,(Australothyris,((Feeserpeton,(Lanthanosuchus,(Colobomycter,Acleistorhinus))),(Nyctiphruretus,((Eudibamus.cursoris,Belebey.vegrandis),(Procolophon.trigoniceps,(Owenetta.rubidgei,Barasaurus)),((Bradysaurus.baini,(Scutosaurus.kapinskii,Pareiasuchus.peringueyi)),((Tokosaurus,Macroleter.poezicus),(Rhipaeosaurus,Bashkyroleter.bashkyricus,Nycteroleter,Emeroleter,Bashkyroleter.mesensis))))))))))));
tree Cisnerosetal2004 =
((Barasaurus,(Owenetta.rubidgei,(Saurodectes,(Owenetta.kitchingorum,Candelaria)))),(Coletta.seca,(Sauropareion.anoplus,Procolophon.trigoniceps)));
tree Cisnerosetal2008 =
(MESOSAURIDAE,((Nyctiphruretus,Macroleter.poezicus),((Eunotosaurus,Milleretta.rubidgei),(Broomia.perplexa,(Millerosaurus.nuffieldi,(Milleropsis.pricei,Millerosaurus.ornatus))))));

tree Lee1995 =
((Nyctiphruretus,Nycteroleter),(((Owenetta.rubidgei,Barasaurus),Procolophon.trigoniceps),(Lanthanosuchus,(Sclerosaurus.armatus,Pareiasaurus.serridens))));
tree Lee1997 =
((Nycteroleter,Nyctiphruretus),((Procolophon.trigoniceps,(Owenetta.rubidgei,Barasaurus)),(Lanthanosuchus,(Sclerosaurus.armatus,(Bradysaurus.seeleyi,(Bradysaurus.baini,Nochelesaurus.alexanderi,(Embrithosaurus.schwarzi,(Deltavjatia.mesensis,(((Shansisaurus.xuecunensis,(Shihtienfenia.permica,(Pareiasuchus.peringueyi,Pareiasuchus.nasicornis))),(Pareiasaurus.serridens,((Sanchuansaurus,Scutosaurus.kapinskii),Elginia.mirabilis))),(Provelosaurus.americanus,(Anthodon.serrarius,Pumiliopareia.pricei)))))))))));
tree MacDougall&Modesto2010 =
(Owenetta.rubidgei,(Coletta.seca,Pintosaurus.magnidentes,Sauropareion.anoplus,Kitchingnathus.utabeni,(Phaantosaurus,((Eumetabolodan.dongshengensis,Theledectes.perforatus),(Tichvinskia.vjatkensis,(((Timanophon.raridentatus,(Kapes,Thelophon.contritus)),(Eumetabolodan.bathycephalus,(Procolophon.trigoniceps,Thelerpeton.oppressus,Teratophon.spinigenis))),(Pentaedrusaurus.ordosianus,(Neoprocolophon.asiaticus,Phonodus.dutoitorum,((Sclerosaurus.armatus,Scoloparia.glyphanodon),(Leptopleuron.lacertinum,(Hypsognathus.fenneri,Soturnia.caliodon)))))))))));
tree Modesto&Damiani2007 =
(((Owenetta.rubidgei,Barasaurus),(Owenetta.kitchingorum,Candelaria)),(Coletta.seca,(Sauropareion.anoplus,(Phaantosaurus,(Procolophon.trigoniceps,Thelerpeton.oppressus,(Tichvinskia.vjatkensis,(Scoloparia.glyphanodon,(Leptopleuron.lacertinum,Hypsognathus.fenneri))))))));
tree Modesto&DamianiDeBraga =
(Lanthanosuchus,(Macroleter.poezicus,(Pareiasaurus.serridens,((Owenetta.rubidgei,Barasaurus),Sauropareion.anoplus,(Procolophon.trigoniceps,Sclerosaurus.armatus,Koiloskiosaurus,Tichvinskia.vjatkensis,(Thelegnathus,(Leptopleuron.lacertinum,Hypsognathus.fenneri)))))));
tree Modesto&DamianiPineiro =
(Owenetta.kitchingorum,(Owenetta.rubidgei,(Pintosaurus.magnidentes,Coletta.seca,Sauropareion.anoplus,Phaantosaurus,Tichvinskia.vjatkensis,(Procolophon.trigoniceps,Thelegnathus,(Scoloparia.glyphanodon,(Leptopleuron.lacertinum,Hypsognathus.fenneri))))));
tree Modestoetal2001 =
((Barasaurus,Owenetta.rubidgei,Owenetta.kitchingorum),(Coletta.seca,(Sauropareion.anoplus,(Phaantosaurus,(Tichvinskia.vjatkensis,(Procolophon.trigoniceps,Hypsognathus.fenneri))))));
tree Modestoetal2002 =
(Coletta.seca,(Phaantosaurus,(Tichvinskia.vjatkensis,(Procolophon.trigoniceps,Leptopleuron.lacertinum))));
tree Modestoetal2003 =
(((Barasaurus,Owenetta.rubidgei,Saurodectes),Owenetta.kitchingorum),(Coletta.seca,(Sauropareion.anoplus,Procolophon.trigoniceps)));
tree Mulleretal2008 =
(Acleistorhinus,Owenetta.rubidgei,(Eudibamus.cursoris,((Bolosaurus.striatus,Bolosaurus.grandis),(Belebey.chengi,Belebey.vegrandis,Belebey.maximi))));
tree Reisz&Scott2002 =
(Petrolacosaurus,(MESOSAURIDAE,(Milleretta.rubidgei,((Macroleter.poezicus,(Pareiasaurus.serridens,(Procolophon.trigoniceps,(Owenetta.rubidgei,Barasaurus)))),(Acleistorhinus,Lanthanosuchus)))));
tree Saila2008 =
(Nyctiphruretus,((Barasaurus,Owenetta.kitchingorum),(Coletta.seca,(Sauropareion.anoplus,((((Procolophon.trigoniceps,Thelerpeton.oppressus,Teratophon.spinigenis),(((Hypsognathus.fenneri,Leptopleuron.lacertinum),Scoloparia.glyphanodon),(Kapes,Anomoiodon))),Tichvinskia.vjatkensis),Phaantosaurus)))));

tree Sues&Reisz2008 =
(Lanthanosuchus,(Macroleter.poezicus,(Owenetta.kitchingorum,(Sauropareion.anoplus,(Procolophon.trigoniceps,(Tichvinskia.vjatkensis,(Sclerosaurus.armatus,(Hypsognathus.fenneri,Leptopleuron.lacertinum),Scoloparia.glyphanodon)))))));
tree Tsuji2006 =
(MESOSAURIDAE,((Eunotosaurus,Milleretta.rubidgei),((Acleistorhinus,Lanthanosuchus),((Belebey.vegrandis,Eudibamus.cursoris),((Procolophon.trigoniceps,(Owenetta.rubidgei,Barasaurus)),(Nyctiphruretus,(Macroleter.poezicus,(Bradysaurus.baini,Scutosaurus.kapinskii))))))));
tree minisupertree10 =
(Captorhinus.aguti,(Araeoscelis,(Claudiosaurus,(Youngina,((Trilophosaurus.buettneri,(Prolacerta,(CHORISTODERA,(DINOSAURIA,Hyperodapedon)))),((KUEHNEOSAURIDAE,(SQUAMATA,Planocephalosaurus)),((Odontochelys.semitestacea,Proganochelys.quenstedti),(Thalattosaurus.alexandrae,((Cyamodus.rostratus,Placodus),((Corosaurus,(Cymatosaurus,(Yunguisaurus,(Pistosaurus,(Bobosaurus,(Augustasaurus,(Thalassiodracon.hawkinsi,Plesiosaurus.dolichodeirus))))))),(Hanosaurus,(Wumengosaurus,(Qianxisaurus,((Dactylosaurus,Neusticosaurus.pusillus,Anarosaurus),(Dianopachysaurus,(Keichousaurus,Diandongosaurus,(Chinchenia,(Sanchiaosaurus,Kwangsisaurus)),(Simosaurus,(Germanosaurus,(Nothosaurus.mirabilis,((Lariosaurus.curionii,Lariosaurus.balsami),(Lariosaurus.buzzi,(Lariosaurus.valceresii,(Lariosaurus.xingyiensis,Lariosaurus.calcagnii)))))))))))))))))))))))));
tree Druckmiller&Russell2008 =
(Cymatosaurus,(Augustasaurus,(((CRYPTOCLIDIA,Plesiosaurus.dolichodeirus),(Thalassiodracon.hawkinsi,Pleisiopterys.wildi)),(Rhomalesaurus.victor,(Rhomalesaurus.megacephalus,(Macroplata.tenuiceps,(PLIOSAURIDAE,(Leptocleidus,(Umoonasaurus.demoscyllus,(Polycotylus.latipinnis,(Dolichorhynchops.osborni,Edgarosaurus.muddi)))))))))));
tree Holmesetal2008 =
(Anarosaurus,Dactylosaurus,Neusticosaurus.pusillus,Neusticosaurus.peyeri,Neusticosaurus.edwardsi,Neusticosaurus.toeplitschi,Keichousaurus,Serpianosaurus,Hanosaurus,(Cymatosaurus,(Lariosaurus.balsami,Nothosaurus.mirabilis),Pistosaurus,Simosaurus,Placodus));
tree Klein&Albers2009 =
(Simosaurus,(Germanosaurus,(Nothosaurus.juvenilis,Nothosaurus.winkelhorsti,(Nothosaurus.yangiuanensis,Nothosaurus.youngi,Nothosaurus.edingerae,Nothosaurus.marchicus,Nothosaurus.winterswijkensis,(Nothosaurus.giganteus,Nothosaurus.mirabilis,Nothosaurus.jagisteus,(Nothosaurus.haasi,Nothosaurus.tchernovi))))));
tree Lietal2011 =
((Odontochelys.semitestacea,(Proganochelys.quenstedti,OTHER.CHELONIA)),((Helveticosaurus,Eusaurosphargis),((Thalattosaurus.alexandrae,(Saurosphargis,Sinosaurosphargis)),Simosaurus)));
tree OKeefe2004 =
(Cymatosaurus,(Pistosaurus,((Pleisiopterys.wildi,(Plesiosaurus.dolichodeirus,(Microcleidus,ELASMOSAURIDAE,(CRYPTOCLIDIA,(Tricleidus,(Kimmerosaurus,Morturneria),((Edgarosaurus.muddi,Polycotylus.latipinnis),(Dolichorhynchops.osborni,Trinacromerum))))))),(Thalassiodracon.hawkinsi,(Eurycleidus,(Attenborosaurus,(PLIOSAURIDAE,((Rhomalesaurus.victor,Rhomalesaurus.megacephalus),(Macroplata.tenuiceps,(Rhomalesaurus.zetlandicus,(Simolestes,Leptocleidus)))))))))));
tree Rieppel2000a =
((Simosaurus,Nothosaurus.mirabilis),(Paraplacodus,(Placodus,((Henodus,Cyamodus.rostratus),(Macroplacus,(Protenodontosaurus,(Placochelys,Psephoderma)))))));
tree Rieppel2000b =
(Paraplacodus,(Placodus,((Henodus,(Cyamodus.rostratus,Cyamodus.kuhnschnyderi)),(Macroplacus,(Protenodontosaurus,(Placochelys,Psephoderma))))));

tree Rieppel&Zannon1997 =
(Youngina,((Simosaurus,Neusticosaurus.pusillus),((Placodus,Paraplacodus),Henodus,Cyamodus.rostratus,Psephoderma,Placochelys)));
tree Storrs1993 =
(Captorhinus.aguti,(Petrolacosaurus,(Youngina,(Claudiosaurus,((Neusticosaurus.pusillus,Serpianosaurus,(Anarosaurus,(Dactylosaurus,Keichousaurus))),(Placodus,((Corosaurus,(Simosaurus,(Cymatosaurus,(Paranothosaurus,Nothosaurus.mirabilis,(Silvestorsaurus,(Lariosaurus.balsami,Ceresiosaurus)))))),(Pistosaurus,(Plesiosaurus.dolichodeirus,PLIOSAURIDAE)))))))));
tree Muller2005 =
(Youngina,(((Askeptosaurus.italicus,Anshuansaurus.huangguoshuensis),Endennasaurus.acutirostris),((((Thalattosaurus.alexandrae,(Clarazia.schinzi,Hescheleria.ruebeli)),Paralonectes.merriami),Agkistrognathus.campbelli),(Xinpusaurus.baomaolinensis,Nectosaurus.halius))));
tree Liu&Rieppel2005 =
(((Askeptosaurus.italicus,Anshuansaurus.huangguoshuensis),Endennasaurus.acutirostris),((((Thalattosaurus.alexandrae,Thalattosaurus.borealis),(Clarazia.schinzi,Hescheleria.ruebeli,Paralonectes.merriami,Nectosaurus.halius)),(Xinpusaurus.suni,Xinpusaurus.baomaolinensis)),Agkistrognathus.campbelli));
tree Jiangetal2003 =
(Askeptosaurus.italicus,(Anshuansaurus.huangguoshuensis,(Clarazia.schinzi,Hescheleria.ruebeli,(Thalattosaurus.alexandrae,(Xinpusaurus.baomaolinensis,Nectosaurus.halius)))));
tree Wuetal2009 =
((((Askeptosaurus.italicus,Miodentosaurus),(Anshuansaurus.wushaensis,Anshuansaurus.huangguoshuensis)),Endennasaurus.acutirostris),((((Thalattosaurus.alexandrae,(Clarazia.schinzi,Hescheleria.ruebeli)),Paralonectes.merriami),Agkistrognathus.campbelli),(Xinpusaurus.baomaolinensis,Nectosaurus.halius)));
tree minisupertree7 =
(PLACENTA,(Procolophon.trigoniceps,(Captorhinus.aguti,(Araeoscelis,(Orovenator,Apsisaurus,Youngina,Galesphyrus,Thadeosaurus,Lanthanolania,Kenyasaurus,Tangasaurus,Acerosodontosaurus,Hovasaurus,(Claudiosaurus,(Coelurosauravus,(Saurosternon,Palaeagama),(Protorosaurus,CHORISTODERA,((Hupehsuchus,ICHTHYOPTERYGIA),(Askeptosaurus.italicus,(Thalattosaurus.alexandrae,Clarazia.schinzi))),(Proganochelys.quenstedti,(Helveticosaurus,(Placodus,(Serpianosaurus,(Simosaurus,Pistosaurus))))),(Tanystropheus,(Macrocnemus,(Prolacerta,(Trilophosaurus.buettneri,(Hyperodapedon,DINOSAURIA))))),((Drepanosaurus,(Pamelina,KUEHNEOSAURIDAE)),(Marmoretta,(Sophineta,(Planocephalosaurus,SQUAMATA)))))))))));

Ichthyopterygia
tree Jiangetal2006 =
((Mixosaurus.panxianensis,(Mixosaurus.cornalianus,Mixosaurus.kuhnschnyderi)),((Phalarodon.fraasi,Phalarodon.callawayi),Contectopalatus.atavus));
tree Maisch&Matzke2003a =
(Thaisaurus,(Utatsusaurus,((Grippia.longirostris,Chaohusaurus),(Parvinatator,((Wimanius,(Mixosaurus.cornalianus,(Phalarodon.fraasi,Contectopalatus.atavus))),((Toretocnemus,Qianichthyosaurus),((Cymbospondylus.petrinus,Phantomosaurus),(Merriamosaurus,(Besanosaurus,(Shastasaurus,(Shonisaurus,(Mikadocephalus.gracilirostris,(Californosaurus,(Callawayia,(Macgowania,Hudsonelpidia,(Temnodontosaurus.platyodon,((Leptonectes,Eurhinosaurus),(Suevoleviathan,(Ichthyosaurus,(Stenopterygius,OPTHALMOSAURIA)))))))))))))))))))));
tree Maisch&Matzke2003b =
(Thaisaurus,(Utatsusaurus,((Grippia.longirostris,Chaohusaurus),(Parvinatator,(Quasianosteosaurus,((Wimanius,(Mixosaurus.cornalianus,(Phalarodon.fraasi,Contectopalatus.atavus))),((Toretocnemus,Qianichthyosaurus),((Cymbospondylus.petrinus,Phantomosaurus),(Merriamosauru

s,(Besanosaurus,(Shastasaurus,(Shonisaurus,(Mikadocephalus.gracilirostris,Californosaurus,(Callawayia,(Macgowania,Hudsonelpidia,(Temnodontosaurus.platyodon,((Leptonectes,Eurhinosaurus),(Suevoleviathan,(Ichthyosaurus,(Stenopterygius,OPTHALMOSAURIA))))))))))))))))));
tree Maisch&Matzke2005 =
(Grippia.longirostris,((Cymbospondylus.petrinus,Mikadocephalus.gracilirostris),(Mixosaurus.maotaiensis,((Mixosaurus.cornalianus,Mixosaurus.kuhnschnyderi),(Contectopalatus.atavus,(Phalarodon.callawayi,(Phalarodon.major,Phalarodon.fraasi)))))));
tree Sander2000 =
((Petrolacosaurus,Araeoscelis),(Grippia.longirostris,(Utatsusaurus,((Mixosaurus.cornalianus,Phalarodon.fraasi),((Shonisaurus,(Besanosaurus,Cymbospondylus.petrinus)),(Temnodontosaurus.platyodon,(Temnodontosaurus.burgundiae,(Suevoleviathan,(Platypterygius,(Eurhinosaurus,(Ichthyosaurus,(Leptonectes,(Stenopterygius,OPTHALMOSAURIA)))))))))))));
tree minitree =
(Claudiosaurus,((Hovasaurus,Thadeosaurus),(Hupesuchus,(Utatsusaurus,Parvinatator,(Xinminosaurus,(Chaohusaurus,Grippia.longirostris,((Cymbospondylus.buchseri,Cymbospondylus.petrinus),((Phalarodon.fraasi,Contectopalatus.atavus,Mixosaurus.cornalianus),((Besanosaurus,(Guizhouichthyosaurus,(Shonisaurus,(Callawayia,Shastasaurus)))),((Qianichthyosaurus,Toretocnemus),(Californosaurus,(Macgowania,(Hudsonelpidia,(Suevoleviathan,(Temnodontosaurus.platyodon,((Stenopterygius,(OPTHALMOSAURIA,Ichthyosaurus)),(Leptonectes,(Eurhinosaurus,Excalibosaurus))))))))))))))))));

Lepidosauromorpha
tree Apesteguia&Novas2003 =
(Diphydontosaurus,(Homeosaurus.maximilliani,((Brachyrhinodon.taylori,Clevosaurus.hudsoni),(Palaeopleurosaurus.posidoniae,((Kallimodon,Sapheosaurus.thiolleri),(Pamizinsaurus,((Zapatadon,(Sphenodon.punctatus,Cynosphenodon.huizachalensis)),(Opisthias,(Toxolophosaurus,(Eilenodon,Priosphenodon))))))))));
tree Apesteguiaetal2012 =
(Diphydontosaurus,(Planocephalosaurus.robinsonae,(Rebbanasaurus,((Sphenocondor,Godavarisaurus),((Clevosaurus.hudsoni,Brachyrhinodon.taylori),(Palaeopleurosaurus.posidoniae,((Sapheosaurus.thiolleri,Kallimodon),((Theretairus,Sphenovipera),(Homeosaurus.maximilliani,Zapatadon,Cynosphenodon.huizachalensis,Sphenodon.punctatus,Ankylosphenodon,(Kawasphenodon,Opisthias,(Kaikaifilusaurus,Eilenodon,Toxolophosaurus)))))))))));
tree Dupret2004 =
(Youngina,SQUAMATA,(Gephyrosaurus.bridensis,(Diphydontosaurus,Planocephalosaurus.robinsonae,(Polysphenodon.muelleri,Homeosaurus.maximilliani,((Cynosphenodon.huizachalensis,Sphenodon.punctatus),(((Pleurosaurus.goldfussi,Pleurosaurus.ginsburgi),Palaeopleurosaurus.posidoniae),Sapheosaurus.thiolleri)),Brachyrhinodon.taylori,Clevosaurus.hudsoni))));
tree Evans&BorsulBialynicka2009 =
(Youngina,Coelurosauravus,Saurosternon,(Prolacerta,((Kuehneosaurus,Paliguana),(Marmoretta,(Sophineta,(Gephyrosaurus.bridensis,SQUAMATA))))));
tree Gauthieretal1988 =
(((Tangasaurus,Youngina),Acerosodontosaurus),Palaeagama,(Paliguana,Saurosternon,(Kuehneosaurus,((Gephyrosaurus.bridensis,(Clevosaurus.hudsoni,(Homeosaurus.maximilliani,Sapheosaurus.thiolleri,Sphenodon.punctatus))),SQUAMATA))));
tree Rauhautetal2012 =
(SQUAMATA,(Gephyrosaurus.bridensis,(Diphydontosaurus,(Planocephalosaurus.robinsonae,((Homeosaurus.maximilliani,(Brachyrhinodon.taylori,Clevosaurus.hudsoni,Clevosaurus.wangi)),((Palaeopleurosaurus.posidoniae,Pleurosaurus.goldfussi),((Kallimodon,Sapheosaurus.thio

lleri),((Sphenodon.punctatus,(Oenosaurus,(Cynosphenodon.huizachalensis,Zapatadon))),(Opisthias,(Toxolophosaurus,(Eilenodon,Priosphenodon))))))))));
tree Reynoso2000 =
(Diphydontosaurus,Gephyrosaurus.bridensis,(Planocephalosaurus.robinsonae,(Palaeopleurosaurus.posidoniae,Clevosaurus.hudsoni,(Brachyrhinodon.taylori,Polysphenodon.muelleri),Pamizinsaurus,Homeosaurus.maximilliani,Sapheosaurus.thiolleri,Kallimodon,(Sphenodon.punctatus,Cynosphenodon.huizachalensis),Zapatadon,(Toxolophosaurus,Eilenodon,Ankylosphenodon))));
tree Suesetal1994 =
(Gephyrosaurus.bridensis,(Diphydontosaurus,(Planocephalosaurus.robinsonae,(Polysphenodon.muelleri,(Brachyrhinodon.taylori,((Clevosaurus.hudsoni,Clevosaurus.petilius),(Clevosaurus.bairdi,Clevosaurus.mcgilli))),Homeosaurus.maximilliani,Kallimodon,Palaeopleurosaurus.posidoniae,Sphenodon.punctatus))));

<u>Synapsida</u>
tree Amson&Laurin2011 =
(Dimetrodon.limbatus,(Tetraceratops,(Raranimus.dashankouensis,((((Biarmosuchus.tener,(Hipposaurus,(Herpetoskylax,(Lycaenodon,(Lemurosaurus,(Proburnetia,Burnetia)))))),(Gorgonops.torvus,(Cyonosaurus,Lycaenops))),(Biseridens.qilianicus,(Patranomodon,Suminia))),(((Syodon.biarmicum,Titanophoneus.potens),Sinophoneus.yumenensis),((Styracocephalus,Jonkeria),Estemmenosuchus.uralensis))))));
tree Angielczyk&Rubidge2012 =
(Eodicynodon.oosthuizeni,(Colobodectes,(Lanthanostegus,((Eosimops,(Robertia,(Prosictodon,Diictodon))),((Chelydontops,Endothiodon),(Pristerodon,((Emydops.arctatus,((Myosaurus,(Dicynodontoides,Kombuisia.frerensis)),(Cistecephalus,(Cistecephaloides,Kawingasaurus)))),((Rhachiocephalus.magnus,((Oudenodon.bainii,(Tropidostoma,Australobarbarus)),(Odontocyclops.dubius,(Idelesaurus,(Aulacephalodon.bainii,(Pelanomodon.tuberosus,Geikia.locusticeps)))))),Interpresosaurus,Elph,(Katumbia.parringtoni,(Delectosaurus,(Dicynodon.lacerticeps,(Lystrosaurus.murrayi,Dicynodon.trautscholdi,Dicynodon.amalitzkii,Kannemeyeria.simocephala)))))))))));
tree Benson2012 =
((Captorhinus.aguti,Protorothyris.archeri),(((Archaeothyris.florensis,(Varanosaurus.acutirostris,(Ophiacodon.mirus,Stereophallodon.ciscoensis))),(Archaeovenator.hamiltonensis,(Pyozia.mesenensis,((Mycterosaurus.longiceps,(Mesenosaurus.romeri,Heleosaurus.scholtzi)),((Varanops.brevirostris,(Watongia.meieri,Varanodon.agilis)),(Ruthiromia.elcobriensis,(Aerosaurus.wellesi,Aerosaurus.greenleeorum))))))),(((Eothyris.parkeyi,Oedaleops.campi),(Oromycter.dolesorum,(Casea.broilii,(Trichasaurus.texensis,(Euromycter.rutena,(Ennatosaurus.tecton,(Cotylorhynchus.romeri,Angelosaurus.romeri,Angelosaurus.dolani,(Cotylorhynchus.hancocki,Cotylorhynchus.bransoni)))))))),(Ianthodon.schultzei,(Ianthasaurus.hardestii,(Lupeosaurus.kayi,Glaucosaurus.megalops,(Edaphosaurus.boanerges,Edaphosaurus.novomexicanus))),(Haptodus.garnettensis,(Pantelosaurus.saxonicus,((Raranimus.dashankouensis,(Biarmosuchus.tener,(Titanophoneus.potens,Biseridens.qilianicus))),(Cutleria.wilmarthi,(Secodontosaurus.obtusidens,(Cryptovenator.hirschbergeri,(Dimetrodon.milleri,Sphenacodon)))))))))));
tree Bermanetal1995 =
(Mycterosaurus.longiceps,((Ianthasaurus.hardestii,(Haptodus.garnettensis,Sphenacodon)),(Archaeothyris.florensis,(Ophiacodon.mirus,Varanosaurus.acutirostris))));
tree Cisnerosetal2011 =
(Tetraceratops,(Raranimus.dashankouensis,(Lycosuchus.vanderrieti,((Biarmosuchus.tener,(Gorgonops.torvus,Herpetoskylax)),((Estemmenosuchus.uralensis,(Tapinocaninus.pamelae,(Titanophoneus.potens,Syodon.biarmicum))),(Biseridens.qilianicus,((Tiarajudens,Anomocephalus)

,((Galechirus,(Otsheria,(Suminia,Ulemica))),(Patranomodon,(Galeops,(Eodicynodon.oosthuizeni,(Diictodon,Robertia)))))))))));
tree Damianietal2007 =
(((((((((((((Ischigualastia,Stahleckeria),Dolichuranus),Kannemeyeria.simocephala),Shansiodon),Vinceria),Tetragonias),Dinanomodon.rubidgei),Lystrosaurus.murrayi),((((Pelanomodon.tuberosus,Oudenodon.bainii),Aulacephalodon.bainii),Dicynodon.lacerticeps),Daptocephalus.leoniceps)),((Cistecephalus,Myosaurus),Rhachiocephalus.magnus),Dicynodontoides),Endothiodon),((Diictodon,Robertia),Pristerodon)),Eodicynodon.oosthuizeni);
tree Frobisch&Reisz2011 =
(Titanophoneus.potens,Gorgonops.torvus,(Lycosuchus.vanderrieti,(Biseridens.qilianicus,(Anomocephalus,((Otsheria,(Ulemica,Suminia)),(Galechirus,Galepus,Patranomodon,(Galeops,(Eodicynodon.oosthuizeni,(Colobodectes,((Diictodon,Robertia),((Pristerodon,(Lanthanostegus,(Chelydontops,Endothiodon))),(((Emydops.arctatus,Emydops.oweni),((Dicynodontoides,(Kombuisia.frerensis,Kombuisia.antarctica)),(Myosaurus,(Cistecephalus,Cistecephaloides,Kawingasaurus)))),((Rhachiocephalus.magnus,((Oudenodon.bainii,Tropidostoma,Australobarbarus),(Idelesaurus,(Aulacephalodon.bainii,(Pelanomodon.tuberosus,(Geikia.elginensis,Geikia.locusticeps)))))),((Elph,Interpresosaurus),(Delectosaurus,(Dicynodon.lacerticeps,((Lystrosaurus.murrayi,Kannemeyeria.simocephala),(Dicynodon.amalitzkii,Dicynodon.trautscholdi)))))))))))))))))));
tree Frobischetal2010 =
(Ophiacodon.mirus,(Edaphosaurus.cruciger,(Haptodus.garnettensis,(Palaeohatteria,Pantelosaurus.saxonicus,Ianthodon.schultzei,(Cutleria.wilmarthi,((Secodontosaurus.obtusidens,(Cryptovenator.hirschbergeri,(Sphenacodon,Ctenospondylus,Dimetrodon.limbatus))),(Biarmosuchus.tener,Titanophoneus.potens)))))));
tree Frobischetal2010b =
(Gorgonops.torvus,(Lycosuchus.vanderrieti,(Anomocephalus,(Patranomodon,((Galeops,(Otsheria,(Ulemica,Suminia))),(Eodicynodon.oosthuizeni,(Colobodectes,((Diictodon,Robertia),((Pristerodon,(Lanthanostegus,(Chelydontops,Endothiodon))),(((Emydops.arctatus,Emydops.oweni),((Dicynodontoides,(Kombuisia.frerensis,Kombuisia.antarctica)),(Myosaurus,(Cistecephalus,Cistecephaloides,Kawingasaurus)))),((Rhachiocephalus.magnus,((Oudenodon.bainii,Tropidostoma,Australobarbarus),(Idelesaurus,(Aulacephalodon.bainii,(Pelanomodon.tuberosus,(Geikia.elginensis,Geikia.locusticeps)))))),((Elph,Interpresosaurus),(Delectosaurus,(Dicynodon.lacerticeps,((Lystrosaurus.murrayi,Kannemeyeria.simocephala),(Dicynodon.amalitzkii,Dicynodon.trautscholdi))))))))))))))));
tree Sigurdsenetal2012 =
(Gorgonops.torvus,(Galepus,((Charassognathus.gracilis,(Dvinia.prima,Procynosuchus.delaharpeae)),(Lycosuchus.vanderrieti,(Glanosuchus.macrops,Ictidosaurus.angusticeps,Pristerognathus.polyodon),((Euchambersia.mirabilis,(Promoschorhynchus.platyrhinus,Olivierosuchus.parringtoni),(Cerdops.burgheri,Moschorhinus.kitchingi)),(Ictidostoma.hemburyi,Hofmeyria.atavus,Mirotenthes.digitipes,(Viatkosuchus.sumini,(Moschowhaitsia.vjuschkovi,Theriognathus.microps)),(Ictidosuchus.primaevus,Ictidosuchops.rubidgei,Ichtidosuchoides.longiceps,Regisaurus.jacobi,Scaloposaurus.constrictus,Ericiolacerta.parva,Bauria,(Lycideops.longiceps,(Choerosaurus.dejageri,(Tetracynodon.tenuis,Tetracynodon.darti))))))))));
tree Kammereretal2011 =
(Anomocephalus,(Patranomodon,((Suminia,(Otsheria,Ulemica)),((Galepus,Galechirus),(Galeops,(Eodicynodon.oelofseni,((Eodicynodon.oosthuizeni,Colobodectes),(Lanthanostegus,(Chelydontops,(Endothiodon,(Pristerodon,(((Diictodon,(Eosimops,(Prosictodon,Robertia))),(Emydops.arctatus,((Dicynodontoides,Kombuisia.frerensis),(Myosaurus,(Cistecephalus,(Cistecephaloides,Kawingasaurus)))))),((Keyseria.benjamini,(Daqingshanodon.limbus,((Oudenodon.bainii,(Tropidostoma,Australobarbarus)),((Odontocyclops.dubius,Idelesaurus),((Rhachiocephalus.magnus,Kitchinganomodon),((Aulacephalodon.bainii,Pelanomodon.tuberosus),(Geikia.locusticeps,Geikia.elginensis))))))),(Interpresosaurus,Elph,Katumbia.parringtoni,(Gordonia.traquairi,

(Delectosaurus,(Dicynodon.trautscholdi,((Dicynodon.lacerticeps,Dicynodon.huenei),((Daptoc
ephalus.leoniceps,(Dinanomodon.gilli,Dicynodon.amalitzkii)),((Jimusaria.sinkianensis,(Syops
.vanhoepeni,((Euptychognathus.bathyrhynchus,(Sintocephalus.alticeps,Basilodon.woodwardi)
),(Lystrosaurus.curvatus,((Lystrosaurus.declivis,Lystrosaurus.murrayi),(Lystrosaurus.maccaig
i,(Lystrosaurus.hedini,Kwazulusaurus.shakai))))))),(Turfanodon.bogdaensis,(((Dinodontosaur
us,(Dolichuranus,Rechnisaurus)),((Tetragonias,Vinceria),(Shansiodon,Rhinodicynodon))),(A
ngonisaurus,(Xiyukannemeyeria.brevirostris,((Uralokannemeyeria,(Parakannemeyeria.dolich
ocephala,(Rabidosaurus,(Kannemeyeria.simocephala,Kannemeyeria.lophorhina)))),(Sinokann
emeyeria.pearsoni,((Placerias,Moghreberia),((Rhadiodromus,Wadiasaurus),((Stahleckeria,San
gusaurus),(Jachaleria,Ischigualastia))))))))))))))))))))))))))))))));
tree Kissel&Reisz2004 =
(Ophiacodon.mirus,(Edaphosaurus.cruciger,(Haptodus.garnettensis,(Palaeohatteria,(Ianthodo
n.schultzei,(Pantelosaurus.saxonicus,(Cutleria.wilmarthi,((Sphenacodon,Dimetrodon.limbatus
),(Biarmosuchus.tener,Titanophoneus.potens)))))))));
tree Liuetal2009b =
((Gorgonops.torvus,(Biarmosuchus.tener,Herpetoskylax)),((Biseridens.qilianicus,(Anomocep
halus,((Otsheria,(Ulemica,Suminia)),(Patranomodon,(Galeops,Eodicynodon.oosthuizeni))))),(
Estemmenosuchus.uralensis,(Titanophoneus.potens,Syodon.biarmicum))));
tree Maddinetal2008 =
((Varanops.brevirostris,Mycterosaurus.longiceps),(Eothyris.parkeyi,(Oromycter.dolesorum,(C
asea.broilii,(Euromycter.rutena,(Ennatosaurus.tecton,(Cotylorhynchus.romeri,Angelosaurus.d
olani)))))));
tree Mayierski&Reisz2010 =
(Ophiacodon.mirus,(Haptodus.garnettensis,(Ianthasaurus.hardestii,(Glaucosaurus.megalops,L
upeosaurus.kayi,(Edaphosaurus.novomexicanus,(Edaphosaurus.colohistion,(Edaphosaurus.bo
anerges,(Edaphosaurus.cruciger,Edaphosaurus.pogonias))))))));
tree Modesto1994 =
(Captorhinus.aguti,((Cotylorhynchus.romeri,Eothyris.parkeyi),(Mycterosaurus.longiceps,(Hap
todus.garnettensis,(Ianthasaurus.hardestii,(Glaucosaurus.megalops,Edaphosaurus.cruciger)))))
);
tree Modestoetal2003 =
(Patranomodon,(Galeops,(Eodicynodon.oelofseni,(Eodicynodon.oosthuizeni,(Colobodectes,((
Diictodon,Robertia),(Emydops.arctatus,(Pristerodon,Chelydontops))))))));
tree Ray2006 =
(Robertia,((Diictodon,Pristerodon),(Cistecephalus,(Kingoria,(Dicynodon.lacerticeps,(Lystrosa
urus.murrayi,(Kannemeyeria.simocephala,(Wadiasaurus,(Stahleckeria,Placerias)))))))));
tree Reiszetal1992 =
(Archaeothyris.florensis,(Ianthasaurus.hardestii,(Haptodus.garnettensis,((Sphenacodon,(Cteno
spondylus,(Dimetrodon.limbatus,Secodontosaurus.obtusidens))),Titanophoneus.potens))));
tree Reiszetal2009 =
(Petrolacosaurus,((((Cotylorhynchus.romeri,Euromycter.rutena),Casea.broilii),(Eothyris.parke
yi,Oedaleops.campi)),((Archaeothyris.florensis,Ophiacodon.mirus),(((Mesenosaurus.romeri,
Mycterosaurus.longiceps),Varanops.brevirostris),Archaeovenator.hamiltonensis))));
tree Rieszetal2010 =
(((Casea.broilii,Eothyris.parkeyi),((Archaeothyris.florensis,Ophiacodon.mirus),(Archaeovenat
or.hamiltonensis,(Apsisaurus,(Mycterosaurus.longiceps,Mesenosaurus.romeri,(Elliotsmithia,(
Aerosaurus.greenleeorum,(Varanops.brevirostris,Varanodon.agilis)))))))),(Captorhinus.aguti,(
Paleothyris,((Petrolacosaurus,Araeoscelis),(Coelurosauravus,((Acerosodontosaurus,Youngina
),(((Trilophosaurus.buettneri,Prolacerta),Hyperodapedon),(Planocephalosaurus,SQUAMATA
))))))));

tree Rubidge&Kitching2003 =
((Hipposaurus,(Bullacephalus,(Proburnetia,Burnetia))),(Australosyodon.nyaphuli,(Gorgonops.torvus,Ictidosuchus.primaevus)));
tree Rubidge&vandenHeever1997 =
(Anteosaurus.magnificus,(Estemmenosuchus.uralensis,(Styracocephalus,(Jonkeria,Tapinocaninus.pamelae))));
tree Rubidgeetal2006 =
(((Titanophoneus.potens,Syodon.biarmicum),(Jonkeria,Estemmenosuchus.uralensis)),(Gorgonops.torvus,(Biarmosuchus.tener,(Hipposaurus,(Lycaenodon,Herpetoskylax,(Lemurosaurus,(Lobalopex,(Proburnetia,(Burnetia,(Pachydectes,Bullacephalus))))))))));
tree Sidor&Hopson1998 =
(Edaphosaurus.cruciger,(Haptodus.garnettensis,(Sphenacodon,(Biarmosuchus.tener,((Anteosaurus.magnificus,Estemmenosuchus.uralensis),(Dicynodon.lacerticeps,(Gorgonops.torvus,(Ictidosuchus.primaevus,(Dvinia.prima,(Procynosuchus.delaharpeae,(Galesaurus,(Thrinaxodon,(Cynognathus,(Probelesodon,(Probainognathus,(Pachygenelus,(Sinoconodon,Morganucodon)))))))))))))))));
tree Sidor&Rubidge2006 =
(Dimetrodon.limbatus,(((Syodon.biarmicum,Titanophoneus.potens),(Jonkeria,Estemmenosuchus.uralensis)),(Patranomodon,Suminia),Biarmosuchus.tener,(Hipposaurus,(Herpetoskylax,Lycaenodon,(Ictidorhinus,(Lobalopex,Lemurosaurus))))));
tree Sidor&Welman2003 =
(Dimetrodon.limbatus,(Anteosaurus.magnificus,(Biarmosuchus.tener,(Hipposaurus,(Lycaenodon,Herpetoskylax,(Ictidorhinus,(Lemurosaurus,(Proburnetia,(Bullacephalus,Burnetia)))))))));
tree Surkovetal2005 =
(((((((((((((Dinodontosaurus,Stahleckeria,Ischigualastia),Wadiasaurus,Placerias),Kannemeyeria.simocephala,Zambiasaurus),Rhinodicynodon,Tetragonias,Angonisaurus),((Lystrosaurus.georgi,Lystrosaurus.curvatus),Lystrosaurus.murrayi,Lystrosaurus.maccaigi)),Dicynodon.trigonocephalus,Oudenodon.bainii),Aulacephalodon.bainii),Pelanomodon.tuberosus),Tropidostoma),Dicynodontoides),Pristerodon),Robertia),Diictodon),(Cistecephalus,Kawingasaurus));
tree minisupertree1 =
(Casea.broilii,Eothyris.parkeyi,Cotylorhynchus.romeri,((Ophiacodon.mirus,Archaeothyris.florensis),(Pyozia.mesenensis,Archaeovenator.hamiltonensis,((Heleosaurus.scholtzi,(Mesenosaurus.romeri,Mycterosaurus.longiceps)),(Elliotsmithia,(Aerosaurus.greenleeorum,(Varanops.brevirostris,(Varanodon.agilis,Watongia.meieri))))))));
tree minisupertree2 =
((Estemmenosuchus.uralensis,Ulemosaurus.svijagensis,Estemmenosuchus.mirabilis,Tapinocaninus.pamelae),(Microsyodon,Archaeosyodon.praeventor,((Pampaphoneus.biccai,(Notosyodon.gusevi,(Australosyodon.nyaphuli,Syodon.biarmicum))),(Sinophoneus.yumenensis,(Titanophoneus.adamanteus,(Titanophoneus.potens,Anteosaurus.magnificus))))));
tree minisupertree3 =
(Herpetoskylax,Lycaenodon,(Ictidorhinus,(Lophorhinus,Lemurosaurus,Lobalopex,((Proburnetia,Paraburnetia),(Pachydectes,Bullacephalus,Niuksenitia,Burnetia)))));
tree minisupertree4 =
(Gorgonops.torvus,(Lycosuchus.vanderrieti,Pristerognathus.polyodon,Ictidosuchus.primaevus,Anomocephalus,(Patranomodon,((Otsheria,(Suminia,Ulemica)),(Galeops,(Eodicynodon.oosthuizeni,(Dicynodon.lacerticeps,Pristerodon,Diictodon)))))));
tree Abdala2007 =
(Prorubidgea,(Lycosuchus.vanderrieti,(Glanosuchus.macrops,(Hofmeyria.atavus,(Ictidosuchops.rubidgei,Moschorhinus.kitchingi,Bauria,(Theriognathus.microps,((Procynosuchus.delaharpeae,Dvinia.prima),(Cynosaurus,(Galesaurus,Progalesaurus),(Thrinaxodon,(Platycraniellus,((Ecteninion,(Cynognathus,(Diademodon,(Trirachodon,(Massetognathus,Exaeretodon))))),(Lum

kuia,(Chiniquodon,(Probainognathus,(Brasilodon,((Pachygenelus,(Oligokyphus,Kayentatherium)),(Brasilitherium,(Morganucodon,Sinoconodon))))))))))))))));
tree Abdalaetal2006 =
(Diademodon,(((Cricodon,Langbergia),Trirachodon),(Luangwa,Scalenodon.angustifrons,(Scalenodon.hirschsoni,Pascualgnathus,Traversodon,(Andescynodon,(Dadadon,(Santacruzodon,(Massetognathus,(Gomphodontosuchus,(Scalenodontoides,(Exaeretodon,Menadon))))))))))));
tree Bonaparteetal2005 =
((Cynognathus,(Pascualgnathus,(Exaeretodon,Oligokyphus))),(Lumkuia,(Probainognathus,(Probelesodon,(Chiniquodon,((Riograndia,Pachygenelus),((Prozostrodon,Therioherpeton),((Brasilitherium,Brasilodon),(Adelobasileus,(Megazostrodon,((Morganucodon,Hadrocodium),(Dinnetherium,Kuehneotherium)))))))))))));
tree Bothaetal2007 =
(Lycosuchus.vanderrieti,((Moschorhinus.kitchingi,(Ictidosuchops.rubidgei,Olivierosuchus.parringtoni,(Regisaurus.jacobi,Bauria))),(Theriognathus.microps,(Charassognathus.gracilis,(Procynosuchus.delaharpeae,Dvinia.prima),(Cynosaurus,((Galesaurus,Progalesaurus),((Nanictosaurus,Thrinaxodon),(Cynognathus,Lumkuia))))))));
tree Hopson&Kitching2001 =
(Lycosuchus.vanderrieti,(Dvinia.prima,Procynosuchus.delaharpeae,(Galesaurus,(Thrinaxodon,((Lumkuia,(Ecteninion,((Probelesodon,(Chiniquodon,Aleodon)),(Probainognathus,(Pachygenelus,Morganucodon))))),(Cynognathus,(Diademodon,(Trirachodon,(Pascualgnathus,(Scalenodon.angustifrons,(Luangwa,(Massetognathus,((Scalenodon.hirschsoni,Oligokyphus),(Gomphodontosuchus,Exaeretodon))))))))))))));
tree Liu&Olson2010 =
(Cynognathus,(Lumkuia,(Exaeretodon,Chiniquodon,(Probainognathus,(Prozostrodon,(Therioherpeton,((Sinoconodon,Morganucodon),(Riograndia,Pachygenelus))))))));
tree Lucas&Luo1993 =
(Thrinaxodon,Probainognathus,(Pachygenelus,(Oligokyphus,(Sinoconodon,(Adelobasileus,Haldanodon,Megazostrodon,Morganucodon,MONOTREMATA,MULTITUBERCULATA,PLACENTA)))));
tree Luo1994 =
(Probainognathus,(Diademodon,(Traversodon,(Oligokyphus,(Pachygenelus,(Sinoconodon,(Adelobasileus,Haldanodon,Kuehneotherium,Triconodon,Dinnetherium,(Morganucodon,Megazostrodon))))))));
tree Luoetal2001 =
(Probainognathus,Oligokyphus,Pachygenelus,(Adelobasileus,Sinoconodon,(Morganucodon,(Haldanodon,(Hadrocodium,(Triconodon,Ornithorhynchus,MULTITUBERCULATA,(Zhangheotheirum,(Vincelestes,(MARSUPIALIA,PLACENTA)))))))));
tree Martinezetal1996 =
(Cynognathus,(Massetognathus,((Chiniquodon,Probelesodon),(Ecteninion,Probainognathus,(Pachygenelus,(Oligokyphus,Morganucodon))))));
tree Oliveiraetal2010 =
(Lycosuchus.vanderrieti,Bauria,(Procynosuchus.delaharpeae,(Galesaurus,(Thrinaxodon,(Cynognathus,Diademodon,Chiniquodon,Lumkuia,Probainognathus,(Ecteninion,Trucidocynodon),(Massetognathus,Exaeretodon),(Brasilodon,((Brasilitherium,Morganucodon),(Pachygenelus,(Oligokyphus,Kayentatherium)))))))));
tree Oliveira2006 =
(Lumkuia,((Probelesodon,Chiniquodon),(Probainognathus,(Exaeretodon,(Prozostrodon,(Sinoconodon,Morganucodon),((Brasilodon,Brasilitherium),((Therioherpeton,Riograndia),(Chaliminia,(Irajatherium,Pachygenelus,Diarthrognathus,Elliotherium)))))))));
tree Rowe1988 =
(Scalenodontoides,Exaeretodon,Scalenodon.angustifrons,Luangwa,(Oligokyphus,HARAMIY

IDAE,Pachygenelus,(Morganucodon,Sinoconodon,Dinnetherium,(Kuehneotherium,(MONOTREMATA,DRYOLESTIDA,Ambolotheirum,Amphitheirum,(Phascolotherium,Triconodon,Peramus,Trioracodon,Spalacotherium,(MULTITUBERCULATA,(MARSUPIALIA,PLACENTA))))))));
tree Sidor&Hancox2006 =
(Lumkuia,(Cynognathus,Exaeretodon),(Probainognathus,((Probelesodon,Chiniquodon),(Therioherpeton,((Prozostrodon,(Sinoconodon,Morganucodon)),(Riograndia,((Irajatherium,(Pachygenelus,Diarthrognathus)),(Chaliminia,Elliotherium))))))));
tree Sidor&Smith2004 =
(Lycosuchus.vanderrieti,(Dvinia.prima,(Procynosuchus.delaharpeae,((Cynosaurus,(Progalesaurus,Galesaurus)),Bolotridon,(Platycraniellus,Thrinaxodon,(Lumkuia,Trirachodon))))));
tree Sues&Hopson2010 =
(Thrinaxodon,(Cynognathus,(Diademodon,(Trirachodon,((Pascualgnathus,Andescynodon),(Scalenodon.hirschsoni,Massetognathus,Traversodon,Dadadon,Santacruzodon,(Scalenodon.angustifrons,Luangwa),(Boreogomphodon,Arctotraversodon,Nanogomphodon),(Gomphodontosuchus,(Menadon,(Exaeretodon,Scalenodontoides)))))))));
tree Wible1991 =
(Cynognathus,Diademodon,Exaeretodon,(Probainognathus,(Pachygenelus,(Oligokyphus,(Sinoconodon,(Morganucodon,Dinnetherium,Kuehneotherium,((MULTITUBERCULATA,HARAMIYIDAE),(MONOTREMATA,Vincelestes,(MARSUPIALIA,PLACENTA)))))))));
tree Liu&Li2003 =
(Xiyukannemeyeria.brevirostris,((Sinokannemeyeria.pearsoni,(Sinokannemeyeria.yingchiaoensis,Sinokannemeyeria.sanchuanheensis)),(Parakannemeyeria.ningwuensis,(Parakannemeyeria.youngi,(Parakannemeyeria.dolichocephala,Parakannemeyeria.shenmuensis)))));
tree Liuetal2002 =
(Kwazulusaurus.shakai,Lystrosaurus.youngi,(Lystrosaurus.curvatus,Lystrosaurus.platyceps),((Lystrosaurus.oviceps,(Lystrosaurus.declivis,(Lystrosaurus.murrayi,Lystrosaurus.maccaigi))),(Lystrosaurus.hedini,Lystrosaurus.broomi,(Lystrosaurus.robustus,Lystrosaurus.shichanggouensis))));
tree Maisch2001 =
(Dicynodon.lacerticeps,((Vinceria,(Rhinodicynodon,(Shansiodon,Tetragonias))),((Kannemeyeria.simocephala,Wadiasaurus),(((Stahleckeria,Angonisaurus),(Placerias,Ischigualastia)),(Dinodontosaurus,((Parakannemeyeria.dolichocephala,Sinokannemeyeria.pearsoni),(Rhadiodromus,(Rechnisaurus,Sangusaurus))))))));
tree Maisch2002 =
((Cteniosaurus.platyceps,(Oudenodon.bainii,(Rhachiocephalus.magnus,Rhachiocephalus.crassus))),(Propelanomodon.devilliersi,((Aulacephalodon.bainii,Pelanomodon.tuberosus),(Odontocyclops.dubius,((Lystrosaurus.murrayi,Kwazulusaurus.shakai),(Dicynodon.lacerticeps,(Kannemeyeria.simocephala,Dinanomodon.rubidgei)))))));
tree Maisch&Gebauer2005 =
(Oudenodon.bainii,(Aulacephalodon.bainii,(Pelanomodon.tuberosus,(Geikia.locusticeps,Geikia.elginensis))));
tree Vegadiasetal2004 =
(Kannemeyeria.simocephala,Wadiasaurus,(Shansiodon,Tetragonias),(Parakannemeyeria.dolichocephala,Sinokannemeyeria.pearsoni),(Dinodontosaurus,(Placerias,((Angonisaurus,Stahleckeria),(Ischigualastia,Jachaleria)))));
tree Govender&Yates2009 =
(Dicynodon.lacerticeps,(Lystrosaurus.murrayi,(Dicynodon.trautscholdi,(((Shansiodon,Tetragonias),Vinceria),(Wadiasaurus,(Kannemeyeria.simocephala,(((((Sinokannemeyeria.pearsoni,Parakannemeyeria.dolichocephala),Dolichuranus),Rechnisaurus),Dinodontosaurus),(Placerias,(Ischigualastia,(Jachaleria,(Stahleckeria,Angonisaurus))))))))));

Testudines

tree minisupertree9 =
(Odontochelys.semitestacea,Simosaurus,Anthodon.serrarius,Owenetta.kitchingorum,(Proganochelys.quenstedti,(Australochelys.africanus,Palaeochersis.talampayensis,(OTHER.CHELONIA,Proterochersis.robusta))));
tree Anquetin2012 =
(Odontochelys.semitestacea,(Proterochersis.robusta,Proganochelys.quenstedti,Palaeochersis.talampayensis,Australochelys.africanus,OTHER.CHELONIA));
tree Anquetinetal2009 =
(Proganochelys.quenstedti,(Palaeochersis.talampayensis,Australochelys.africanus,Proterochersis.robusta,OTHER.CHELONIA));
tree Gaffneyetal2007 =
(Proganochelys.quenstedti,(Australochelys.africanus,(Palaeochersis.talampayensis,((Proterochersis.robusta,MEGAPLEURODIRA),OTHER.CHELONIA))));
tree Rougieretal1995 =
(Proganochelys.quenstedti,((Palaeochersis.talampayensis,Australochelys.africanus),(Proterochersis.robusta,OTHER.CHELONIA)));

Supplementary Data 2
MRP matrices produced by SuperTree0.85b for each category and used in the formation of the supertree.

Archosauromorpha
#NEXUS

begin data;
dimensions ntax=229 nchar=616;
format datatype=standard missing=?;
matrix
Batrachotomus
111000011???00001111111111111??
?????????????????00000111??
???
???
?????????????????????000000000000100011100000011111??????????????????????????????
????????????????????????????0000000000000000000000011111111??????????????????????
???00000000000000000000000000000000000
00000000000011111111111111???????????????????
Prestosuchus
111000011???11000000001111111??
???
???
???111110000000011?????????????
??????????????????????000000000000100011100000011111?????????????????????????????
?????????????????????????????0000000000000000000000111111111?????????????????????
??0000000000000000000000000000000000
000000000000000111111111111???????????????????
Saurosuchus
011000011??????????????001111???????????????????????????01000000011111111?
??????????????????00001111??
???
???
??????????????????????000000000000000111100000011111?????????????????????????????
????00001111100000011111111110000000000000000000000000111111??????????????????
???00000000000000000000000000000000
00000000000000001111111111111???????????????????
Ornithosuchus
001000011???00000000000111111??
???
???
???011110000000011??????????00
11111110000011111111110000000000000000000000000111111?????????????????????????
?????????????????????????????????000000000000000000010000001111 1?????????????????
????????????????????????111011110001110000011111110000000000000000000000000001
000000000000000000000000111111111???????????????????
Sphenosuchus
00011111111??????????????????1111???
??????????011111??
???

??
??
???????????????????????00000000000000111100000000011111000111?????????????????????
???000000000000000000000000000000000000000
0000111111111111111111???????????????????
CROCODYLIFORMES
0001111101???????????0001111111???????????????????????00110001111111111
?????????????1111100011111???????????????????????????????0000000000000000000000
000001???111110000000000000000001???????
??00111000000011???
????????00011111110000000111111111000000000110000000000000001111???????????????
??
??????????????????????????????0110111??????????????????000000000000000000000000
000000000000000011111111111111111111???????????????????
Erpetosuchus
000011111????????????????????0111???
??????????000111???
??
??
????????????????????000000000110000000000000001111???????????????????????????????
???????????????????????????0000000000000000111000000011111???????????????????????
??
???????????????????????????????????????
Postosuchus.kirkpatricki
000001111??????????????011110011???
??????????00001100111111???
??011111000000000000000001??????????????
???11111000000011??????????
??????????????????????????000000000000011111100000011111?????????????????????????
?????????????????????????????????000000000000000111000100111111??????????????????
??0000000000000000000000000000000
000000011000001111111111111111???????????????????
Gracilisuchus
000000111????????????????????0001???????????????????????00010001111111111?
??
??
??
?????????????????????000000000001000000000000001111??????????????????0000011111
1111100011111000000111111111000000000000000001100000000011111????????????????
???000000000000000000000000000000
0000000000000000000001111111111???????????????????
Stagonolepis.robertsoni
000000001??100000000011??????????1100001000011????????????????????????????
????????????000001??
??
???00011000000011????????????
??00011111111
1111000001111000000111111111100000000000001100010000000011111?????????????????
??0000000000000000000000000000110
000000000000000000001111111111???????????????????

Dibothrosuchus
?????????11???
????????111111??
???
???
???
???
??????????????????????????????????0000000000000000000000000000000000000000000111
1111111111111111???????????????????
Saltoposuchus
?????????01???
????????001111??
???
???
???
???
???
?????????????????????????????
Hesperosuchus.agilis
?????????01???
????????001111??
???1111100000000000000000001????????????????
???
???
???
?????????????????????????????????????0000000000000000000000000000000000000000000
0011111111111111111???????????????????
Pseudohesperosuchus
?????????01???
????????001111??
???
???
???
???
???
?????????????????????????????
Litargosuchus
?????????01???
????????001111??
???
???
???
???
??????????????????????????????????0000000000000000000000000000000000000000001111
1111111111111111???????????????????
Kayentasuchus
?????????01???
????????001111??
???
???
???

???
??????????????????????????????????000000000000000000000000000000000000000011111
1111111111111111???????????????????
Terrestrisuchus
??????????01??
????????001111??
???
???
???000
11111100000011111111110000000000000011110000000011111?????????????????????????
??00000000000000000000000000000000000000
00001111111111111111111???????????????????
Aetosauroides
???????????100000000011??????????1100001000011?????????????????????????????
???
???
???
???
???
???
???
???????????????????????????????
Redondasuchus
???????????011100000111??????????0000111000011?????????????????????????????
???
???
???
???
???
???
???
???????????????????????????????
Typothorax
???????????011100000111??????????0000111000011?????????????????????????????
???
???
???
???
???
???
???
???????????????????????????????
Coahomasuchus
???????????001100000111??????????0100001000011?????????????????????????????
???
???
???
???
???
???
???
???????????????????????????????
Stagonolepis.wellesi
???????????000100000111??????????1100001000011?????????????????????????????
???

???
???
???
???
???
???????????????????????????????
Desmatosuchus.haplocerus
???????????000011111111??????????0000000111111?????????????????????????????
???
???
???
???
???????????????????????00000000000011000100000000011111??????????????????????????
???
???????????????????????????????????
Acaenasuchus
???????????000011111111??????????0000000011111?????????????????????????????
???
???
???
???
???
???
???????????????????????????????
Lucasuchus
???????????000001111111??????????0000000001111?????????????????????????????
???
???
???
???
???
???
???????????????????????????????
Longosuchus
???????????000000111111??????????0000000000111?????????????????????????????
???
???
???
???
???
????????????????????????????????????0000000000000000000000000000110000000000000
000000001111111111???????????????????
Paratypothorax
???????????000000011111??????????0011011000011?????????????????????????????
???
???
???
???
???
???
???????????????????????????????

Neoaetosauroides
???????????000000001111??????????0100001000011?????????????????????????????
???
???
???
???
???
???
???????????????????????????????
Aetosaurus
???????????0000000000001000011????1100001000011?????????0000000011111111
1???????????????????00000001??
???000011000000000000000001???????????
???
???????????????????????????000000000000000000000000001111?????0000010111111????
????????????????????????????????????0000000000000100010000000011111??????????????
???0000000000000000000000000000
110000000000000000000001111111111???????????????????
Poposaurus.gracilis
???????????????????????111111???????????????????????????00110001111111111??
?????????????????01000011???
???
???
?????????????????????000000000000000000001011111111??????????????????????????????
????????????????????????????0000000000000000000010000111111??????????????????????
???00000000000000000000000000000000001
11100000000000011111111111???????????????????
Arizonasaurus
???????????????????????111111??
???????????????01000011???
???
???
???????????????????000000000000000000000111111111????????????????????????????????
??????????????????????????0000000000000000000010000111111????????????????????????
???0000000000000000000000000000001000011
100000000000011111111111???????????????????
Shuvosaurus
???????????????????????111111??
???????????????11000011???
???
???
???????????????????000000000000000000110111111111????????????????????????????????
??????????????????????????0000000000000000000110000111111????????????????????????
???0000000000000000000000000000000111111
100000000000011111111111???????????????????
Marasuchus
???????????????????????000001???????????????????????????00000000000001111??
?????????????????????????????????????000000000000000000100000000000000000000000
00011????????????????????????000000000000000100000000000000000000001111??????
???0000000011111??
?????????11111111110001111111111100000001110000000000000000001111??????????????

????00100011111111100000000000011111111111110000000111000000000000000000111?
??0000000000000000
000000001110000000000000000000000000011111111???????????????????
Heliocanthis
?????????????????????????????????0011011000011??????????????????????????????
???
???
???
???
???
???
??????????????????????????????
Tecovasuchus
?????????????????????????????????0001011000011??????????????????????????????
???
???
???
???
???
???
??????????????????????????????
Desmatosuchus.smalli
?????????????????????????????????0000000111111??????????????????????????????
???
???
???
???
???
???
??????????????????????????????
Revueltosaurus
?????????????????????????????????0000000000001??????????????????????????????
???
???
???
???????????????????000000000000000000000000001111????????????????????????????????
???
???????????????????????????????????????0000000000000000000000000000010000000000
000000000000111111111???????????????????
Nicrosaurus.kapffi
??1000000011?????????????????????
???
???
???
???
???
???
?????????????????????????????
Nicrosaurus.meyeri
??1000000011?????????????????????
???

??
??
??
??
??
?????????????????????????????
Pseudopalatus.pristinus
???0111010111?????????????????000
1100000000??
??
??
??
??
?????????????????????????????????????0011000000000000000000000000000000000000000
00000000000001111111???????????????????
Pseudopalatus.buceros
???0111010111?????????????????????
??
??
??
??
??
??
?????????????????????????????
Pseudopalatus.mccauleyi
???0011010111?????????????????000
0100000000??
??
??
??
??
??
????????????????????????????????
Pseudopalatus.jablonskiae
???0001010111?????????????????????
??
??
??
??
??
??
?????????????????????????????
Redondasaurus.gregorii
???0000110111?????????????????????
??
??
??
??
??
??
?????????????????????????????

Redondasaurus.bermani
??0000110111?????????????????????
???
???
???
???
???
???
?????????????????????????????
Mystriosuchus.planirostris
??0000001111?????????????????????
???
???
???
???
?????????????????????0000000001100000000000000001111?????????????????????????????
???
?????????????????????????????????
Mystriosuchus.westphali
??0000001111?????????????????000
1100000000???
???
???
???
???
???
????????????????????????????????
Ebrachosuchus.neukami
??0000000001?????????????????????
???
???
???
???
???
???
?????????????????????????????
Ticinosuchus
??11000000001111111???
???
???
???
???????????????????000000000000000000100000011111????????????????????????????????
??????????????????????????0000000000000000000000000111111????????????????????????
???0000000000000000000000000000000000000
000000000000011111111111???????????????????
Rauisuchus
??00001111111111111???
???
???
???
???????????????????000000000000001111100000011111????????????????????????????????

??????????????????????????000000000000000000000000001111111????????????????????????
???0000000000000000000000000000000000000
001000001111111111111111???????????????????
Fasolasuchus
??000001111111111111???
???
???
???
???????????????????000000000000000111100000011111????????????????????????????????
???
???????????????????????????????????????0000000000000000000000000000000000000000
000000111111111111111???????????????????
Lotosaurus
??00000011111111111???
???
???
???
???????????????????000000000000000000000001111111????????????????????????????????
??????????????????????????0000000000000000000001000011111????????????????????????
???0000000000000000000000000000000001111
100000000000001111111111???????????????????
Turfanosuchus.dabanensis
??00000000111111111???
???
???
???
???0000000011111110
000000010000001111111111??
??0000000000000000000000000000000000000000
000000000001111111111???????????????????
Lewisuchus
??00000000111111111???
???
???
???
???????????????????000101111100000000000000000111????????????????????????????????
??????????????????????????0001011111000000000000000000111????????????????????????
???0000000000100000000000011100000000000
000000000000000011111111???????????????????
Phytosaurus.doughtyi
??0000000000001111100
00000111111??
???
???00001000000011???????????0
00111111100000011111111100000000000000000000000000001111????????????????????????
???
?????????????????????00101111???
???
Proterochampsa
??00000000000000111???
???

???
???00000000000001????????????000
0011111000000011111110000000000000000000000000000011?????000100000111??????
??????????????????????????????????000000000000000000000000000011????????????????
??????????????????????????00000111??
???
Euparkeria
??000000000000000011???
???
???
??00001
111110000000111111100000000000000000000000000000001?????0000000001111000000000
0011111000000000000000000111111000000000000000000000000000000001000111???????
?????????????????????????????00000011000011000001111111000000000000000000000000
0000000000000000000000000000000001111110000001100000111111
Erythrosuchus
??000000000000000001???
???
???
??00000
0111110000000000111111?????????????????????????????????0000000000001000000000000
1111110000000000000000000111111??????????????????????????????????001011??????????????
???????????????????????00000001?????????????????????000000000000000000000000000000000
000000000000000000000000000000111???????????????????
Leptosuchus.crosbiensis
??1000
000111111???
??00000100000000000000000001????????????????
???
???
???
???
??????????????????????????????????????
Leptosuchus.studeri
??1000
000111111???
???
???
???
???
???
???????????????????????????????????
Smilosuchus.adamanensis
??0110
001111111???
???
???
???
???
???
???????????????????????????????????

Smilosuchus.gregorii
??0110
001111111???
???
???
???
???
???????????????????????????????????00110000000000000000000000000000000000000000
00000000001111111???????????????????
Smilosuchus.lithodendrorum
??0010
001111111???
???
???
???
???
???
??????????????????????????????
Pravusuchus.hortus#78
??0000
011111111???
???
???
???
???
???
??????????????????????????????
Rutiodon.carolinensis
??0000
000011111???
???
???
???
??????????????????????0000000000110000000000000001111????????????????????????????
???
??????????????????????????????????
Machaeroprosopus.zunii
??0000
000011111???
???
???
???
???
???
??????????????????????????????
Protome.batalaria
??0000
000011111???
???
???
???

??
??
??????????????????????????????
Brachysuchus.megalodon
??0000
000001111???
??
??
??
??
??
??????????????????????????????
Angistorhinus
??0000
000001111???
??
??
??
??
??
??????????????????????????????
Paleorhinus.sawini
??0000
000000111???
??
??
??
??
??
??????????????????????????????
Paleorhinus.bransoni
??0000
000000011???
??
??
??
??????????????????????0000000000010000000000000001111????????????????????????????
??
??????????????????????????????????
Paleorhinus.scurriensis
??0000
000000001???
??
??
??
??
??
??????????????????????????????
Dromichosuchus
??
????????001111??

???
???
???
???
??????????????????????????????????000000000000000000000000000000000000000000001
1111111111111111???????????????????
Sillosuchus
??
??????????????1100011???
???
???
??????????????????00000000000000000000101111111?????????????????????????????????
?????????????????????????0000000000000000000010000111111?????????????????????????
??00000000000000000000000000000000111111
00000000000011111111111???????????????????
Teratosaurus
??
??????????????0011111???
???
???
???
?????????????????????0000000000000000000010011111111?????????????????????????????
???
?????????????????????????????????
Eudimorphodon.ranzii
??
??????????????????????100000000111???
??000001000000000000000011???????????????????
?????????????????010111111??
????????01100000000011111??
???
???????????????????????0000000001111111000010000000000000000000000010000000000
000000000000000011111111???????????????????
Austriadactylus.cristatus
??
??????????????????????100000000111???
???
???
???
???
???
??????????????????????????????
OTHER.RHAMPHORHYNCHINAE
??
??????????????????????011001111111???
??????????0011111???
???????????????001111111??
???
???
???
????????????????????????????????

Dorygnathus.bathensis
??
??????????????????????011001111111???
???????????0000111??
???????????????000001111??
???
???
???
????????????????????????????????
Scaphognathus.crassirostris
??
??????????????????????001001111111???
???????????0000111??
???????????????000000111??
???
???
???
????????????????????????????????
ANUROGNATHIDAE
??
??????????????????????000111111111???
???????????0000001??
???????????????000000001??
??????001000000011111??
???
???
??????????????????????????????????
PTERODACTYLOIDEA
??
??????????????????????000111111111???
???????????0011111??
???????????????001111111??000
0001111???
???
?????????????00011111???
???????????????????????????????????
Sordes.pilosus
??
??????????????????????000011111111???
???????????0000111??
???????????????000000011??
???
???
???
????????????????????????????????
Campylognathoides.liasicus
??
??????????????????????000000111111???
???????????0101111??
???????????????010111111??
???

???
???
?????????????????????????????????
Dimorphodon.macronyx
??
??????????????????????000000011111???
???????????1000011?????????????????????????0000001000000000000000011???????????????
??????????????????000011111????????????????????????????????00000000001111???????????
??????????????????????????1000000010000000000000000000111??????????????????????????
????????000000000100001111111111110000000010000000000000000000111???????????????
??000100000000000000000000000010
0000000000000000000000000011111111???????????????????
Peteinosaurus.zambelli
??
??????????????????????000000001111???
???
??????????????100011111???
?????011000000011111???
???
???
??????????????????????????????????
Preondactylus.buffarinii
??
??????????????????????000000000011???
???????????1000011??
???????????????000000111??
???
???
???
?????????????????????????????????
Scleromochlus
??
??????????????????????000000000001???
???
???000000000000111???????????011
11111110000111111111111000000001000000000000000000111???????????????????0010001
11111110000000001000011111111111100000000100000000000000000000111??????????????
?????????????????????????????00011111???
???
Scutellosaurus
??
???????????????????????????????????1100000000000011111?????????????????????????????1
100000000001????????????????????????????????0000000000110000000111111????????????1
001110000000000000001??
???
???
???0000000000000100000000011111000000000
00000000000000000011111111???????????????????
Heterodontosaurus
??
???????????????????????????????????1100000000000011111000000000000000000000000011

111100000000001???????10000001110000000000011100000000001100000111111000000
00000101111100000000000000001?????????000000000000001???????????????00000001111
111??????????????????????????????????????0000111110000000000000000000111???????????
??00001111100000000000000000000111???
??0000000000001100000
000011111000000000000000000000000000011111111???????????????????
Lesothosaurus
??
???????????????????????????????????010000000000001111????????????????????????????0
100000000001???????????????01000000000001110000000000110000001111111???????????
?00000110000000000000001???
??
??
??0000000000001000000000111110000000
00000000000000000011111111????????????????????
Herrerasaurus
??
???????????????????????????????????001000000000011111110000000000000000000000000111
110000000001111???????01000011000010000001111110000000000001000011111111110000
00011100000000000000000001111?????????00000000000001101???????????????00000111111
111????????????111111111100011111111111????????????????????????????????????000000
1111111???????????????00000000001111111111111???????????????????????????????????????
??00011111???????????????????0000000000000000
0001111111100000000000000000000000000000011111111????????????????????
Staurikosaurus.pricei
??
???????????????????????????????????001000000000011111110000000000000000000000000111
11?????????????????????010000110000100000011111??????????????????????????0100000001
11??
??
??
???0000000000000000000011111111000000000
000000000000000000011111111????????????????????
Thecodontosaurus.antiquus
??
???????????????????????????????????00011000000011111110000000000000000000000111111
110011000000011??
??????????????????????????????100000000011111110000000000000011??????????????????????
??
??
??
??
Plateosaurus.engelhardti
??
???????????????????????????????????00011000000011111110000111000000000001111111111
110011000000011??????????????????????????????????0000000000000110111111111???????????
??00000001111010000011?????????0000000011111111100110000001111000000111111
1??
??
???000000000000110000001111110
000000000000000000000000011111111????????????????????

Saturnalia
??
??????????????????????????????????000010000000111111100110000000000000000011111
110001000000011???????00111111001100000000111100000000000000101111111???????
???????????????????????????????????00000000000111??0000000000000001???????????????
???
???
???000000000000001000000111111000
000000000000000000000001111111???????????????????
Coelophysis.rhodesiensis
??
??????????????????????????????????0000011101111111111????????????????????????????0
000111011111???
???
???
???
???
?????????????????????????????????
Coelophysis.bauri
??
??????????????????????????????????0000011101111111111????????????????????????????0
000111011111???????????????00000111111111110000000000000001111111110010000111
11000000000000011111111???
???
???
???000000000000000011111111111000000
0000000000000000000011111111???????????????????
Megapnosaurus.kayentakatae
??
??????????????????????????????????0000001110111111111????????????????????????????0
000111011111???????????????0000001111111111????????????????????????001000011111?
???
???
???
???
????????????????????????????????????
Liliensternus.liliensterni
??
??????????????????????????????????0000000110111111111????????????????????????????0
000011011111???00000011111100
0000000000011111111???
???
???
???
??????????????????????????????????????
Dilophosaurus.wetherilli
??
??????????????????????????????????0000000010111111111????????????????????????????0
000001011111???????????????0000000111111111????????????????????????000111111111
000000000000001111111???
???

??
???0000000000000000111111111100000000
0000000000000000011111111???????????????????
CERATOSAURIA
??
????????????????????????????????????00000000111111111??????????????????????????????0
0000001111111????????????????000000000111111??????????????????????????????????????00
0000000000000111111???
??
??
??
???
COELUROSAURIA
??
????????????????????????????????????0000000011111111101000000000000000000001111
110000000111111???????00001111????????????????000000000000000111111111100011111
111100000000000000000011111?????????00000000000111????????????????0000011111111
1???????????????????????????????????????000011111100000000000000000111??????????????
??0000111111000000000000000000000111??????
???00011100000111111000000000000000001111
1111111100000000000000000000000000011111111????????????????????
Eoraptor.lunensis
??
????????????????????????????????????00000000000111111101000000000000000000000001111
110000000000111???????0000011100110000000001111????????????????????????????????????
??0000000000000000000111???
??
?????????0000000000111111111111111??
??00000000000000000001111111111000
00000000000000000000000111111111????????????????????
Eucoelophysis.baldwini
??
????????????????????????????????????00000000000000001111???????????????????????????????
??000000000100000000011111???????????????
??
????????????????????????0011011111000000000000000000111?????????????????????????????
????????????????????????????????00110111110000000000000000000111????????????????????
???0000000011100000000000001111000000
0000000000000000000111111111????????????????????
Silesaurus
??
????????????????????????????????????00000000000000000111000000000000000000000000001
11????????????????????00000001000000000000000110000000001000000000011111????????
??
????????????????????????????????0011011111000000000000000000000111?????000000111111?
??????????????0000000000111111111111111001101111100000000000000000000111????????
??0000000111100000000000
1111000000000000000000000000011111111????????????????????
Pseudolagosuchus
??
????????????????????????????????????000000000000000000011?????????????????????????????????

???
???
????????????????????000000111100000000000000000111???????????????????????????????
???????????????????????????00000011110000000000000000000111??????????????????????
???
???????????????????????????????????????
Chromogisaurus
??
???0011000000000000000001111111
???
???
???
???
???
????????????????????????????????
Angosphitys
??
???0001000000000000000001111111
???
???
???
???
???
????????????????????????????????
Guaibasaurus
??
???0001000000000000000001111111
????????????????????00001111??
???
???
???
???
?????????????????????????????????
Panphagia
??
???0001000000000000000001111111
????????????????????000111110011000000001111??????????????????????????????????????
???
???
???
???
???????????????????????????????????
Plateosaurus.ingens
??
???0000111000000000011111111111
???
???
???
???
???
????????????????????????????????

Plateosaurus.gracilis
??
???0000011000000000011111111111
??0000
00011110100000011????????????????????????11???????????????????????????????????????
???
???
???
????????????????????????????????????
Unaysaurus
??
???0000001000000000011111111111
???
???
???
???
???
????????????????????????????????
Eucenemesaurus
??
???0000000100000000111111111111
???
???
???
???
???
????????????????????????????????
Riojasaurus
??
???0000000100000000111111111111
??0000
00000010100000011?????????00000001111111111??00010000001111????????????????????
???
???
???
???????????????????????????????????????
Lufengosaurus
??
???0000000011100001111111111111
??0000
00001110100000011?????????00000011111111111??11110000001111????????????????????
???
???
???
???????????????????????????????????????
Glacialisaurus
??
???0000000011100001111111111111
???
???
???

???
???
????????????????????????????????
Coloradisaurus
??
???0000000001100001111111111111
???
?????????????????????????0000001111111111011100000001111?????????????????????????
???
???
???
????????????????????????????????????
Massospondylus
??
???0000000001000011111111111111
??0000
00100110100000011?????????0100001111111111111110000001111??????????????????????
???
???
???
???????????????????????????????????????
Melanorosaurus
??
???0000000000011111111111111111
???
?????????????????????????0000111111111111100000000111111?????????????????????????
???
???
???
????????????????????????????????????
EUSAUROPODA
??
???0000000000011111111111111111
?????????????????????001111110001000000001111???????????????????????000000000011
000000000001100000011?????????001111111111111110000111111111?????????????????
???
???
???
??
Yunnanosaurus
??
???0000000000001111111111111111
??0000
00100110100000011?????????010000111111111??
???
???
???
?????????????????????????????????????
Anchisaurus
??
???0000000000000111111111111111

???
?????????????????????????0000000111111111100000000011111?????????????????????????
???
???
???
????????????????????????????????????

Jingshanosaurus

??
???0000000000000011111111111111
???
???
???
???
???
????????????????????????????????

Ruehleia

??
???0000000000000000001111111111
???
???
???
???
???
????????????????????????????????

Plateosauruavus

??
???0000000000000000000111111111
???
???
???
???
???
????????????????????????????????

Pantydraco

??
???0000000000000000000011111111
???
???
???
???
???
????????????????????????????????

Chindesaurus

??
???0000000000000000000000111111
??000000000000100001111111110000000111
???
???
???
???
?????????????????????????????????????

Zupaysaurus
??
???000
0111011111??000001111111?????
???
???
???
???
?????????????????????????????????
Gojirasaurus
??
???000
0111011111???
???
???
???
???
????????????????????????????????
Segisaurus
??
???000
0111011111???
???
???
???
???
????????????????????????????????
Procompsognathus
??
???000
0111011111???
???
???
???
???
????????????????????????????????
Carniadactylus.rosenfeldi
??
???
?????????0101111??
?????????????100011111??
???
???
???
???????????????????????????????
Pisanosaurus
??
???
????????????????10000001?????????????????0000000000010000000111111????????????0000
01000000000000001??
???

???
???00000000000110000000001111100000000000
000000000000001111111???????????????????
OTHER.ORNITHOPODA

??
???
????????????????????????110000000000111????????????????????????????????????011111
00000000000001??
???
???
???
?????????????????????????????????

Megapnosaurus.rhodesiensis

??
???
????????????????????????0000011111111111???
???
???
???
???
??????????????????????????????

Tawa

??
???
????????????????????????000000001111111????????????????????????000000001111?????
???
???
???
?????????????????????????????????????0000000000000000111111111000000000000000
000000000011111111???????????????????

Eodromaeus

??
???
????????????????????????0000000000111111???
???
???
???
???
??????????????????????????????

Effigia

??
???
??00111000000000000000001??????????????????
???
????????????????????00000000000000000011011111111???????????????????????????????
???????????????????????????000000000000000000011000011111???????????????????????
??0000000000000000000000000000011111
110000000000001111111111???????????????????

Riojasuchus

??
???

??000111000000000000000001??????????????????
???
???0000010111111???????????????
?????????????????????????0000000000000000001000000111111?????????????????????????
????????????????11101111??????????????????0000000000000000000000000001000000000
000000000000000111111111???????????????????
Dromomeron.romeri
???
???
??000000011000000000000111??????????????????
???
????????????????????010000001100000000000000000111???????????????????????????????
???????????????????????????0100000011000000000000000000111???????????????????????
??000001100000000000000000110000000000
0000000000000000111111111???????????????????
Dromomeron.gregorii
???
???
??000000011000000000000111??????????????????
???
???
???
????????????????????????????????????0000011000000000000000011000000000000000000
000000000011111111???????????????????
Lagerpeton
???
???
??000000001000000000000111??????????????????
???00000000011111??????????????
??????????????????????010000001100000000000000000111?????????????????????????????
????000000000000011111111111010000001100000000000000000000111??????????????????
???0000001000000000000000000011000
0
000000000000000000000111111111???????????????????
Efraasia
???
???
??000000000000110111111111??????????????????
????????????????????????000000000111111??00000000000111??????????????????????????
???
???
??0000000000000110000001111110000000000000
000000000000011111111???????????????????
Crylophosaurus
???
???
??000011111111??????
???
???
???
???
??????????????????????????????

OTHER.THYREOPHORA

???
??
???10011100
0000000000001???
??
??
??
???????????????????????????????

MARGINOCEPHALIA

???
??
???00111100
0000000000001???
??
??
??
???????????????????????????????

Vulcanodon

???
??
???00000000
0001100000011?????????0011111111111111100001111111111???????????????????????????
??
??
??
???????????????????????????????????

Thecodontosaurus.caducus

???
??
??
?????????????????????100000000011111???
??
??
??
??????????????????????????????

Blikanasaurus

???
??
??
?????????????????????000111111111111???
??
??
??
??????????????????????????????

Euskelosaurus

???
??
??
?????????????????????00000111111111111??
??

???
???
???????????????????????????????
Kotosaurus
??
???
???
??????????????????????????????????????00000111111111???????????????????????????????
???
???
???
??????????????????????????????
Isanosaurus
??
???
???
??????????????????????????????????????00000011111111???????????????????????????????
???
???
???
??????????????????????????????
Antetonitrus
??
???
???
??????????????????????????????????????00000001111111???????????????????????????????
???
???
???
??????????????????????????????
Trilophosaurus
??
???
???
??10000000001??????
???
???00000000100000000001111
1??
??????????????0000000000000011111
Prolacertoides
??
???
???
??10000000001??????
???
???
???
??????????????????????????????
Boreopricea
??
???

??
??0100000011??????
??
???000111011111111
??
??????????????????????????????
Prolacerta
???
??
??
???0100000001100000
00011000000000000011???000000000000
0010000000000000000000000001????????????????????????????????00000001100000
0001111111???????????????????00100000000011111100000000000000000000000000
00000000000000000000000000010000000100000111111
Tanystropheus.longobardicus
???
??
??
??0011111111??????
????00000000000111??
???0111110111111
11???
???????????????001111000000001111
Tanytrachelos
???
??
??
??0011111111??????
??
???011110111111111
??
??????????????????????????????
Tanystropheus.meridensis
???
??
??
??0001111111??????
??
??
??
????????????????????????????
Cosesaurus
???
??
??
??0001111111??????
??
???001111011111111
??
??????????????????????????????

Macrocnemus
??
???
???
??00000111111??????
???
??000001011111111
???????????????????011000000000111111???
????????????????0001110000000001111
Langobardisaurus
??
???
???
??00000011111??????
???
??000011011111111
???????????????????011000000000111111???
????????????????0011110000000001111
Malerisaurus.langstoni
??
???
???
??00000001111??????
???
??000000111111111
???
????????????????????????????????
Protorosaurus
??
???
???
??00000000111??????
???
???
???
???????????0000010000000001111
Proterosuchus
??
???
???
???0000000
111000000000001111???00000000000001
10000000000000000000000011???????????????????????????????010001?????????00000000
0111111???????????????????000001000001111111100000000000000000000000000000000
000000000000000000000000001100000001100000111111
Rhynchosaurus.articeps
??
???
???
???0000000
001000000000000001??????????????????????????????00111?????????????????????????????

??000111111000000000001
111?????000011???
?????????????????0000000000111111111
Megalancosaurus
???
??
??
??
??100000000000111???
??
?????????????????000000111111111111??
??????????????0100110000000001111
Vallesaurus
???
??
??
??
??100000000000111???
??
?????????????????000000000111111111??
????????????????????????????????
Sacisaurus
???
??
??
??
?????????????????0011011111000000000000000111???????????????????????????????????
????????????????????????00110111110000000000000000111?????????????????????????
???0000000111100000000000001111000000000
0000000000000011111111??????????????????
Polonosuchus.silesiacus
???
??
??
??
?????????????????0000000000001111110000011111???????????????????????????????????
??
?????????????????????????????????????000000000000000000000000000000000001100
0001111111111111111??????????????????
Tikisuchus
???
??
??
??
?????????????????0000000000001111110000011111???????????????????????????????????
????????????????????????0000000000000000000000001111111?????????????????????????
??
???????????????????????????????????
Stagonosuchus
???
??

??
??
?????????????????000000000000000000110000001111 1??????????????????????????????????
??
??
????????????????????????????????
Qianosuchus
??
??
??
??
?????????????????0000000000000000001000000 11111??????????????????0000011111111
1000000011000000111111111000000000000000000000000000111111????????????????????
???0000000000000000000000000000000
0001100000000000011111111111??????????????????
Hypselorhachis
??
??
??
??
?????????????????000000000000000000001111111111?????????????????????????????????????
??
?????????????????????????????????????00000000000000000000000000000000100001110000
000000001111111111??????????????????
Ctenosauriscus
??
??
??
??
?????????????????000000000000000000001111111111?????????????????????????????????????
??
?????????????????????????????????????00000000000000000000000000000000100001110000
000000001111111111??????????????????
Bromsgroveia
??
??
??
??
?????????????????000000000000000000001111111111?????????????????????????????????????
????????????????????????00000000000000000000100001111111??????????????????????????
??
????????????????????????????????????
Hyperodapedon.sanjuanensis
??
??
??
??
???11111???????????????????????????????
??111111111???????????????0
1111??
?????????????00000000111111111111

Hyperodapedon.gordoni
??
???
???
???
???11111????????????????????????????????
???111111111??????????????1
1111111111???
??????????????0000000011111111111
Stenaulorhynchus.stockleyi
??
???
???
???
???01111????????????????????????????????
???000011111????????????????
???000111???
????????????0000000001111111111
Howesia.browni
??
???
???
???
???00011????????????????????????????????
???000001111????????????????
???000001???
????????????0000000000011111111
Mesosuchus
??
???
???
???
???00001????????????????????????????????
???000000111????????????????
?????????????????000000000000011111???
??????????????0000000000001111111
Doswellia
??
???
???
???
??10000000000111100000011111110
010000000000000111111111??
???
???????????????????????????????????
Vancleavea
??
???
???
???
??10000000000110000000000001110

010000000000000111111111??
??0000000000000000000000000000000000000000
000000000000000011111??????????????????
Gualosuchus
??
???
???
???
??011110000111??????????????????
???
???
??????????????????????????????
Chanaresuchus
??
???
???
???
??01111000001110000000001111110
000000000000000011111111??
??0100000000000000000000000000000000000000
000000000000000011111??????????????????
Tropidosuchus
??
???
???
???
??001110000111??????????????????
???
???????????????????????????????????01000000000000000000000000000000000000000000
00000000000011111???????????????????
Cerritosaurus
??
???
???
???
??000110000111??????????????????
???
???
??????????????????????????????
Parasuchus
??
???
???
???
??00000000111110000111111111110
000001110000001111111111??
??0001000000000000000000000000000000000000
000000000000001111111??????????????????
Archeopelta
??
???

???
???
???110000001111111???
???
???
??????????????????????????????
Tarjadia
??
???
???
???
???010000001111111???
???
???
??????????????????????????????
Chilenosuchus
??
???
???
???
???000111111111111???
???
???
??????????????????????????????
Yonghesuchus
??
???
???
???
???000000011111111???
???
???
??????????????????????????????
Vjushkovia.triplocostata
??
???
???
???
??1100
000000000000000111111???????????????????????????????001011???????????????????????
???
?????????????????????????????????
Shansisuchus
??
???
???
???
??0100
000000000000000111111???
???
????????????????????????????????

Koilamasuchus
???
???
???
???
??0000
000000000000000011111??
???
???????????????????????????????????
Osmolskina
???
???
???
???
??0000
000000000000000001111??
???
???????????????????????????????????
Sarmatosuchus
???
???
???
???
??0000
000000000000000000111??????????????????????????????????110001?????????????????????????
???
????????????????????????????????????
Fugusuchus
???
???
???
???
??0000
000000000000000000111??????????????????????????????????110001?????????????????????????
???
????????????????????????????????????
Decuriasuchus
???
???
???
???
???
????????????????????00000000000000000000000011111111?????????????????????????????????
???
???????????????????????????????????
Teyumbaita.sulcognathus
???
???
???
???
???

???111111111???????????????00
011111111???
???????????????????????????????
Hyperodapedon.mariensis
??
???
???
???
???
???111111111???????????????01
111???
???????????????????????????????
Hyperodapedon.huxleyi
??
???
???
???
???
???111111111???????????????11
111???
???????????????????????????????
Ammorhucnhus.navajoi
??
???
???
???
???
???011111111??????????????????
???
?????????????????????????????
Fodonyx.spenceri
??
???
???
???
???
???001111111???????????????00
001001111???
???????????????????????????????
Rhynchosaurus.brodiei
??
???
???
???
???
???000111111??????????????????
???
?????????????????????????????
Gephyrosaurus
??
???

??
??
??
???1000000000000011??
?????????????????00000000000000001111???
??????????????1000000000000000111

SQUAMATA

??
??
??
??
??
???1000000000000011??
??
????????????1000000000000000111

Jesairosaurus

??
??
??
??
??
???0000001111111111??
??
?????????????????????????????????

CHORISTODERA

??
??
??
??
??
???0000000000000111??
??
????????????0000000000000000011

Youngina

??
??
??
??
??
???0000000000000001??
?????????????????000000000000000111???
??????????????0000000000000000001

Hyperodapedon.huenei

??
??
??
??
??
??001
11???
?????????????????????????????????

Isalorhynchus.genovefae
??
???
???
???
???
???
?011111???
?????????????????????????????
Bentonyx.sidensis
??
???
???
???
???
???
?000111???
?????????????????????????????
Coelursauravus
??
???
???
???
???
???
???????????????100000000000000011???
???????????????????????????????
Longisquama
??
???
???
???
???
???
???????????????100000000000000011???
???????????????????????????????
Dolabrosaurus
??
???
???
???
???
???
???????????????000000111111111111???
???????????????????????????????
Drepanosaurus
??
???
???
???
???

??
???????????????000000011111111111??
????????????0100110000000001111
Hypuronector
???
??
??
??
??
??
???????????????000000001111111111??
???????????????????????????????
Petrolacosaurus
???
??
??
??
??
??
???????????????000000000000000001??
???????????????????????????????
Archosaurus
???
??
??
??
??
??
?????????????????????????????????10000000000000000000000000000000000000000000000
000000000000011???????????????????
Asilisaurus
???
??
??
??
??
??
?????????????????????????????????00000000011000000000000111100000000000000000000
000000011111111???????????????????
Eocursor
???
??
??
??
??
??
?????????????????????????????????00000000000010000000001111100000000000000000000
000000011111111???????????????????
Xilosuchus
???
??

```
?????????????????????????????????????????????????????????????????????????????????????
?????????????????????????????????????????????????????????????????????????????????????
?????????????????????????????????????????????????????????????????????????????????????
?????????????????????????????????????????????????????????????????????????????????????
?????????????????????????????????0000000000000000000000000000001000011100000000
000011111111111???????????????????
Diandongosuchus
	??????????????????????????????????????????????????????????????????????????????
?????????????????????????????????????????????????????????????????????????????????????
?????????????????????????????????????????????????????????????????????????????????????
?????????????????????????????????????????????????????????????????????????????????????
?????????????????????????????????????????????????????????????????????????????????????
?????????????????????????????????????????????????????????????????????????????????????
?????????????????????????????????0000000000000000000000000000000000000100000000
000011111111111???????????????????
Postosuchus.alisonae
	??????????????????????????????????????????????????????????????????????????????
?????????????????????????????????????????????????????????????????????????????????????
?????????????????????????????????????????????????????????????????????????????????????
?????????????????????????????????????????????????????????????????????????????????????
?????????????????????????????????????????????????????????????????????????????????????
?????????????????????????????????????????????????????????????????????????????????????
?????????????????????????????????0000000000000000000000000000000000000011000001
111111111111111???????????????????
outgroup
	000000000000000000000000000000000000000000000000000000000000000000000
000000000000000000000000000000000000000000000000000000000000000000000000000
000000000000000000000000000000000000000000000000000000000000000000000000000
000000000000000000000000000000000000000000000000000000000000000000000000000
000000000000000000000000000000000000000000000000000000000000000000000000000
000000000000000000000000000000000000000000000000000000000000000000000000000
000000000000000000000000000000000000000000000000000000000000000000000000000
000000000000000000000000000000000000000000000000000000000000000000000000000
0000000000000000000000
;
end;

begin assumptions;
	options deftype=irrev;
end;

begin sets;
	charset Benton&Walker2002 = 1-9;
	charset Cark&Sues2002 = 10-11;
	charset Harrisetal2003 = 12-23;
	charset Nesbitt2003 = 24-29;
	charset Olsonetal2000 = 30-33;
	charset Parker2007 = 34-46;
	charset Parker&Irmis2006 = 47-56;
	charset Parrish1993 = 57-73;
	charset Stocker2012 = 74-86;
```

charset Suesetal2003 = 87-92;
charset Weinbaum&Hungurbuller2007 = 93-100;
charset Andresetal2010 = 101-112;
charset Ezcurra2006 = 113-131;
charset Ezcurra2010 = 132-159;
charset Ezcurra&Novas2007 = 160-172;
charset Lu&Ji2006 = 173-179;
charset Martinez&Alcober2008 = 180-187;
charset Martinezetal2011 = 188-203;
charset Nesbittetal2009 = 204-227;
charset Nesbittetal2009a = 228-239;
charset Sereno1999 = 240-260;
charset Wangetal2005 = 261-269;
charset Yates2003 = 270-284;
charset Yates2003Bentonetal = 285-286;
charset Yates&Kitching2003 = 287-300;
charset Benton1999 = 301-314;
charset Benton&Allen1997 = 315-325;
charset Bennett2012revised = 326-335;
charset Bennett2012updated = 336-350;
charset Butleretal2011BrusatteB = 351-380;
charset Dilkes1995 = 381-385;
charset Dilkes&Arcucci2012 = 386-398;
charset Desojoetal2011 = 399-413;
charset Ezcurraetal2010 = 414-438;
charset Francaetal2012 = 439-469;
charset Gower&Sennikov1997 = 470-475;
charset Hone&Benton2008 = 476-484;
charset Jalil1997 = 485-499;
charset Langer&Schultz2000 = 500-504;
charset Montefeltroetal2013 = 505-510;
charset Sereno1991 = 511-518;
charset Renesto&Binelli2006 = 519-536;
charset minitree1 = 537-597;
charset minitree2 = 598-616;
end;

Basal + Parareptilia + Sauropterygia
#NEXUS

begin data;
dimensions ntax=263 nchar=571;
format datatype=standard missing=?;
matrix
Romeria.texana
1000000000011111000000011100000000000001111111????????????????????????????
??
??
??
??

???
??
Romeria.prima
10000000000011111???
???
???
???
???
???
??
Captorhinus.magnus
0110000011111111???
???
???
???
???
???
??
Captorhinus.aguti
0110000011111111011111111000000000111111111100000000000000000000000
000000000000000000000000001111???????11000000000000000000000000111??????????
00000000000000000011100000000111??????????????????00100000000000000000000011
???
???00000000000000000
0000000000000000000000001??
??????????????????000000000000000001???????????????????????????????????0000000000
0000000000000000111
Captorhinus.laticeps
00100000111111110111111110000000001111111111??????????????????????????
???
???
???
???
???
??
Rothianiscus
0001111111111111???
???
???
???
???
???
??
Gansurhinus
0001111111111111???
???
???
???
???
???
??

Moradisaurus
0000111111111111??
???
???
???
???
???
??
Labidosaurikos
0000011111111111011111111100000000101111111111??????????????????????????
???
???
???
???
???
??
Labidosaurus
0000001111111111011111111100000000101111111111??????????????????????????
???
???
???
???
???
??
Captorhinikos
0000000111111111??
???
???
???
???
???
??
Saurorictus
0000000001111111000111111100000000000011111111??????????????????????????
???
???
???
???
???
??
Protocaptorhinus
0000000000111111000000111100000000000011111111??????????????????????????
???
???
???
???
???
??
Rhiodenticulatus
0000000000001111000011111100000000000011111111??????????????????????????
???

??
??
??
??
???
Concordia
000000000000011100000000110000000000000111111???????????????????????????
??
??
??
??
??
??
???
Thuringothyris
0000000000000011000000000100000000000000011111??????????????????????????
??
??
??
??
??
??
???
Paleothyris
0000000000000001??????????00000011000000001111000000000000000000000000
0000000000000000000000000111110000001?????????????????????????????000000001???
??????????????????0000001111??????????????????0110000000000000000000011??????
??
??
??
??
??
Reiszorhinus
????????????????0000011111???
??
??
??
??
??
???
Procolophon.trigoniceps
??????????????????????????100000000000000000011000000000000000000100011100
000000000000000000000000111???????1100000001111000000000000000011??????????000
000000000000000011000000000110000000000000000000100000000001000000111111110
000111????????00100110010000000000000000011000111000000111111000000111110001 1
11100111101111111111100111?????0111011110100001111111000111110000100011111???
??
??000
000000000000000000000000011
Milleretta.rubidgei
??????????????????????????1000000000000000000110000000000000000000000000100
000000000000000000000000111?????????000000000100000000000000011??????????????
???0000000000000000000001111???????0

1000111??
???????????????000000111?????????????????????1000000000011???????????????????????
??
??
????
Araeoscelis
??????????????????????????0100111100000000111100000000000000000000000000
00000000000000000000001111111100011??0000000000000000000000111111000000011 0
00000000000000001111000000111111??????????????????11100000000000000000000000011?
??
???000000000000000000
0000000000000000000000011???
??00000000000000
000000000001111
Petrolacosaurus
??????????????????????????01001111000000001111?????????????????????????????
?????????????????????????1100011????????????????????????????1000000011???????????
??
??
???000000001???
???000000
000000000011???
Anthracodromeus
??????????????????????????00111111000000001111?????????????????????????????
??
??
??
??
??
??
Protorothyris.archeri
??????????????????????????00111111000000001111?????????????????????????????
?????????????????????????????????000000000000000000000001111????????????????????
??
??
??
??
??
Cephalerpeton
??????????????????????????00011111000000001111?????????????????????????????
??
??
??
??
??
??
Hylonomus
??????????????????????????00000111000000001111?????????????????????????????
??
??
??

??
??
??
Brouffia
??????????????????????????0000000100000001111?????????????????????????????
??
??
??
??
??
??
??
Coelostegus
??????????????????????????0000000000000000111?????????????????????????????
??
??
??
??
??
??
??
MESOSAURIDAE
??????????????????????????000000000000000001100000000000000000000000000
0000000000000000000000000001??
???0000000000000000000000111???????0000
0001???
????????????000000011????????????????????0000000000001???????????????????????????
??
??
?
Casea.broilii
??????????????????????????000000000000000000010000000000000110000000000
0000000000000000000000000011???????0100001000000000000000000001?????????????
??
??
??
??
???
Morganucodon
???1111111111111111000000000000
00000000000000000000000011??
??
??
??
??
???
PLACENTA
???1111111111111111000000000000
00000000000000000000000011???00000000
000000000000100000000001??????????????????0000000000000000000000001?????????
??
??
??

??00000000000000000000000000
001
Kayentatherium
??0111111111111111000000000000
0000000000000000000000011???
??
??
??
??
???
Exaeretodon
??0011111111111111000000000000
0000000000000000000000011???
??
??
??
??
???
Diademodon
??0001111111111111000000000000
0000000000000000000000011???
??
??
??
??
???
Thrinaxodon
??0000111111111111000000000000
0000000000000000000000011???
??
??
??
??
???
Procynosuchus.delaharpeae
??0000011111111111000000000000
0000000000000000000000011?????????1111100000000000000000001???????????????
??
??
??
??
??
Lychosuchus.vanderrieti
??0000001111111111000000000000
0000000000000000000000011???
??
??
??
??
???

Dicynodon.lacerticeps
??0000000111111111000000000000
00000000000000000000000011???
???
???
???
???
??
Gorgonops.torvus
??0000000011111111000000000000
00000000000000000000000011?????????111110000000000000000000001????????????????
???
???
???
???
???
Anteosaurus.magnificus
??0000000001111111000000000000
00000000000000000000000011???
???
???
???
???
??
Biarmosuchus.tener
??0000000000111111000000000000
00000000000000000000000011???
???
???
???
???
??
Sphenacodon
??0000000000011111000000000000
00000000000000000000000011?????????011110000000000000000000001????????????????
???
???
???
???
???
Edaphosaurus.cruciger
??0000000000001111000000000000
00000000000000000000000011?????????001110000000000000000000001????????????????
???
???
???
???
???
Ophiacodon.mirus
??0000000000000111000000000000
00000000000000000000000011?????????000110000000000000000000001????????????????

??
??
??
??
??
Ophiacodon.retroversus
???0000000000000000100000000000
00000000000000000000000011??
??
??
??
??
???
Lanthanosuchus
???0000000000000000100000110000
00000000000000000000000111?????????00001000110000000000000011????????????????
??????????????????????????00000000000000000100000011000000000001111111????????
???????0000111000000000000000000111????????????????????????????00000001????????
???????????????????000011111?????????????000000010100000000111???????????????????
??
??
?????????
Acleistorhinus
???0000000000000000100000110000
00000000000000000000000111?????????00001000110000000000000011????????????????
??00000111000000000001111111??????????
??
???????00001000011111?????????????????????0100000000111??????????????????????????
??
??
??
Owenetta.rubidgei
???0000000000000000010001110000
00000000000000000000000111??
???????????????????????000000000000000010000000001100000001111111110011001???
?????0110011011000000000000000011000000000000000110100000000110001111000011
1000001????1100100001111101111????????????????????000110001111????????????????
??
??
????????????
Pareiasaurus.serridens
???0000000000000000001111110000
00000000000000000000000111?????????0000001111100000000000000011????????????????
??????????????????????????000000000001111111?????????????????????????????????????
????0001111000000011001111111111????????????????????????????00000111???????????
????????????????001101111??
??
??
???
Scutosaurus.kapinskii
???0000000000000000001111110000

00000000000000000000000111???
???????????????????????00000000111111111100000000000110001111111111???????????
???????????000000111100111111111???
??0000011111111??????????????????????????
???
???
??
Anthodon.serrarius
??0000000000000000000111110000
00000000000000000000000111???
???????????????????????0111000000000111111???????????????????????????????????????
????????000000000011111111111??
???
???
??
Bradysaurus.baini
??0000000000000000000011110000
00000000000000000000000111???
???????????????????????0000000000000000111000000000000010001111111111???????????
???????????000000000000000111111??
??0000011111111??????????????????????????
???
???
??
Bradysaurus.seeleyi
??0000000000000000000011110000
00000000000000000000000111???
???????????????????????0000000000000000011??
????????000000000000000011111??
???
???
??
Macroleter.poezicus
??0000000000000000000000110000
00000000000000000000000111???
??000000000000101111111111???????100000
11???00000011????????????????
???????????000101111?????????????000000110000001111111???????????????????????????
???
???
?
Planocephalosaurus
??0000000000000000000000001010
01100000000000000111111111?????????00000000000011100001111111000011111110000
001111111111111111???
???
??0000110000000000000000000
000000000000111111??
??00000000000000000111
111111111

SQUAMATA
??0000000000000000000000001010
0110000000000000011111111?????????0000000000001110000111111110000111110000
001111111111111100111111111???
???
???000011000000000000000000
000000000000111111??
???0000000000000000011
1111111111
Odontochelys.semitestacea
??0000000000000000000000000110
0110000000000000011111111???
???0001100000000000000001111???????????????
???
???0000001000000000000000000000
0000001111111????????????????????????0100001??????????????????????????????????????
???
??
OTHER.CHELONIA
??0000000000000000000000000110
0110000000000000011111111???
???
???
???
????????????????????????????1100001???
???
Proganochelys.quenstedti
??0000000000000000000000000110
0110000000000000011111111?????????000000111110000000000000011?????????00000
0011111111111111011111111111110000000001111110001100000000000000001111????
???
???000000100000000000000
000000000000001111111??????????????????????1100001?????????????????????????????
???0000000010000000
0000011111111
Cyamodus.rostratus
??0000000000000000000000000001
1110000000000000011111111???
???
???
??00000001000000000000000000000000
0111111111??0100011111100011100
111???
?
Placodus
??0000000000000000000000000001
1110000000000000011111111?????????00000000001001000011111111?????????00000
100111111111111111111111111??
???
??0000000100000000000000000
00000000111111111??????????????011???????????????????????????????????000000111000

00011011110000000000000111111???????????????????????????????????0000000111000000
0000011111111
Simosaurus
??0000000000000000000000000000
11100000000000000011111111?????????000000000010010000111111111??????????00000
1001111111111111111111111111??
???
??0000000000000000000000000
11111111111111111??????????????01100000100000111?????????????????????100000001?
???????10011000000011001111111???????????????????????????????????000001111100000
00000011111111
KUEHNEOSAURIDAE
??0000000000000000000000000000
00100000000000000111111111?????????000000000000011000011111111??????????00000
0000000011111111???
???
??000001000000000000000000000
000000000111111??
???00000000000000011000111
111111
Hyperodapedon
??0000000000000000000000000000
00010000000000001111111111?????????00000000000000011111111111001111111110111
10000011111111111???
???
??1110000000000000000000000
00000000000011111???
??00000000001111100000
011111111
Trilophosaurus.buettneri
??0000000000000000000000000000
00010000000000001111111111?????????00000000000000011111111111001111111110001
10000011111111111???
???
??0001000000000000000000000
00000000000011111???
??00000000000111000000
011111111
Rutiodon
??0000000000000000000000000000
00001001111111111111111111???
???
???
???
???
??
Pseudopalatus
??0000000000000000000000000000
0000100111111111111111111111???
???
???

???
???
??
Coahomasuchus.kahleorum
??0000000000000000000000000000
00000111111111111111111111???
???
???
???
???
??
Typothorax.coccinarum
??0000000000000000000000000000
00000111111111111111111111???
???
???
???
???
??
Desmatosuchus.haplocercus
??0000000000000000000000000000
00000011111111111111111111???
???
???
???
???
??
Paratypothorax
??0000000000000000000000000000
00000000111111111111111111???
???
???
???
???
??
Protosuchus.richardsoni
??0000000000000000000000000000
00000000011111111111111111???
???
???
???
???
??
CROCODYLIA
??0000000000000000000000000000
00000000001111111111111111???
???
???
???
???
??

DINOSAURIA
???0000000000000000000000000000
0000000000011111111111111?????????0000000000000000111111111?????????01111
00000111111111111000111111111??
???
??1111000000000000000000000
0000000000011111??
??0000000000111110000
011111111
Proterochampsa
???0000000000000000000000000000
00000000000111111111111??
???
???
???
???
???
Erythrosuchus
???0000000000000000000000000000
000000000000111111111111???
???
???
???
???
???
Proterosuchus
???0000000000000000000000000000
0000000000000111111111111??
???
???
???
???
???
Protorosaurus
???0000000000000000000000000000
00000000000000111111111111???
???
???
???
???
???000000000000000000000011111111
CHORISTODERA
???0000000000000000000000000000
00000000000000001111111111?????????0000000000000000011111111??????????00000
00001111111111111??
???
??0111000000000000000000000
0000000000011111??
???0000000000000000000011
111111

Prolacerta
??0000000000000000000000000000
00000000000000001111111111????????000000000000000000111111111100011111110011
1000001111111111??
??
???0011000000000000000000000
0000000000011111??
???0000000000011100000
011111111
Youngina
??0000000000000000000000000000
0000000000000000011111110011111??0000000000000000000011111101000111110000
000000000001111110000111111????????????????????111000000000000000000000011????
??
??0000000000000000000000
00000000000000000001111??
???????0000100000000000000011100000000001???????????????????????0000000000000
0000000000011111
Claudiosaurus
??0000000000000000000000000000
0000000000000000001111111????????00000000000000000000111111?????????00000
0000000011111100000111111??
??
???00000000000000000000000
000000000000000111??
?????????00000000000001111???????????????????????????????????000000000000000000
00000111111
Spinoaequalis
??
??????????????????????0100011???
??
??
??
??
???
Acerosodontosaurus
??
??????????????????????0011111??????????????????????????????0100011111??????????????
??
??
??
??
???000000000000000000000000011111
Hovasaurus
??
??????????????????????0001111???
??
??
??
??
???0000000000000000000000000011111

Apsisaurus

???
??????????????????????0000111?????????????????????????????00000011110000000000000
00011111???
??
??
??
??00000000000000000000000011111

Nycteroleter

???
???????????????????????????????000000001110000000000000011??????????????????????
??????????????????????????????????????0000000000000011111111111???????????????10
00001100000000000000000001??
??
??
???

Lanthanolania

???
???0000111111????????????????
??
??
??
??
???00000000000000000000000011111

Coelurosauravus

???
??000000011100000000000011
1111111??
??
??
??
???00000000000000000000001111111

Thalattosaurus.alexandrae

???
??100000000001111
111111???
??
???0000000000000000000000000000000000111
11111???????????????????????00001111??
??????????????000111011100101011101111000001110110011100000000000000001111111
1

ICHTHYOPTERYGIA

???
??100000000001111
111111???
??
??
??
??010010000000000000000011111111

Tanystropheus

???

??0000100000011111
111111???
??
??
??
??00000000000000100000011111111
Pumiliopareia.pricei
???
??
???????????????1111000000000111111???
000000000011111111111??
??
??
???
Provelosaurus.americanus
???
??
???????????????0011000000000111111???
000000000001111111111??
??
??
???
Nanoparia.luckhoffi
???
??
???????????????0001000000000111111???
??
??
??
???
Pareiasuchus.nasicornis
???
??
???????????????0000111000001111111???
000111000100111111111??
??
??
???
Pareiasuchus.peringueyi
???
??
???????????????000011100000111111100000000001100011111111111?????????????????????
???000111000100111111111??
??
??
??
Shihtienfenia.permica
???
??
???????????????0000011000001111111???
000011000100111111111??

??
??
???
Shansisaurus.xuecunensis
???
??
???????????????0000001000001111111???
000001000100111111111??
??
??
???
Arganaceras.vacanti
???
??
???????????????0000000101111111111???
??
??
??
??
Elginia.mirabilis
???
??
???????????????0000000101111111111???
000000011100111111111??
??
??
???
Sanchuansaurus
???
??
???????????????0000000011111111111???
000000111100111111111??
??
??
???
Parasaurus.geinitzi
???
??
???????????????0000000000111111111???
??
??
??
??
Deltavjatia.mesensis
???
??
???????????????0000000000000011111???
000000000000011111111??
??
??
???

Embrithosaurus.schwarzi
??
???
???????????????0000000000000001111??
000000000000001111111???
???
???
??
Nochelesaurus.alexanderi
??
???
???????????????0000000000000000111??
000000000000000111111???
???
???
??
Bunostegos.akokanensis
??
???
???????????????0000000000000000001??
???
???
???
???
Sclerosaurus.armatus
??
???
???????????????0000000000000000001???000111
1000000000000000001111000000100111111111???????????00011111????????????????????
?????????????????????????????01111111???
???
???
Barasaurus
??
???
???????????????000000000000000000100000000011000000111111110001001????????011
0011011000000000000000011??????????????????1010000000110001111??????1000001???
?11001?????1111011111000000000011????????0001100011111??????????????????????????
???
???
??
Eunotosaurus
??
???
??????????????????????????????????00010000000000000000001111???????01000111?????
???
????????????????????????????????1000000000011??????????????????????????????????????
???
???
Colobomycter
??

??
????????????????????????????????????00000111000000000001111111??????????????????????
??
??
??
???
Feeserpeton
???
??
????????????????????????????????????00000001000000000001111111??????????????????????
??
??
??
???
Eudibamus.cursoris
???
??
????????????????????????????????????00000000100000000111111111??????????????????????
???000
11????????????????????????????????001000001111??
??
??
Belebey.vegrandis
???
??
????????????????????????????????????00000000100000000111111111??????????????????????
???011
11????????????????????????????????001000001111??
??
??
Tokosaurus
???
??
????????????????????????????????????00000000000001011111111111??????????????????????
??
??
??
???
Rhipaeosaurus
???
??
????????????????????????????????????00000000000000111111111111??????????????????????
??
??
??
???
Bashkyroleter.bashkyricus
???
??
????????????????????????????????????00000000000000111111111111??????????????????????
??

???
???
??
Emeroleter
??
???
??????????????????????????????????00000000000000111111111111?????????????????????
???
???
???
??
Bashkyroleter.mesensis
??
???
??????????????????????????????????00000000000000111111111111?????????????????????
???
???
???
??
Nyctiphruretus
??
???
??????????????????????????????????00000000000000000011111111???????1000001110000
01100000000000000000001???
??????????????0000000000001????????0000000111111?????????????????????????????????
???
??
Australothyris
??
???
??????????????????????????????????00000000000000000000111111?????????????????????
???
???
???
??
Microleter
??
???
??????????????????????????????????00000000000000000000011111?????????????????????
???
???
???
??
Owenetta.kitchingorum
??
???
??1111001?????????????????
?????????????????????????????????????01100000001????????0000011000001????01001???
???????????100000000001100000111??
???
??

Candelaria
???
???
???1111001???????????????????
??0110000001???
???
???
???
Saurodectes
???
???
???0111001???????????????????
??11001??????
???
???
??
Sauropareion.anoplus
???
???
???0000111???????????????????
??????????????????0000000000000001100000000111000011110001110000111????00111
??????????????0000000001111000011111??
???
??
Coletta.seca
???
???
???0000011???????????????????
???????????????????00000000000000011000000000011????????0001110000011000100011
??????????????000000000111???
???
???
Milleropsis.pricei
???
???
???00111111?????????
???
???
???
??
Millerosaurus.ornatus
???
???
???00111111?????????
???
???
???
??
Millerosaurus.nuffieldi
???
???

???00011111?????????
???
???
???
??
Broomia.perplexa
???
???
???00001111?????????
???
???
???
??
Eumetabolodan.dongshengensis
???
???
???
??????????????????100000000000001111??
???
???
??
Theledectes.perforatus
???
???
???
??????????????????100000000000001111??
???
???
??
Kapes
???
???
???
??????????????????011001000000111111??
?????????0000111111111???
???
??
Thelophon.contritus
???
???
???
??????????????????011001000000111111??
???
???
??
Timanophon.raridentatus
???
???
???
??????????????????001001000000111111??
???

??
???
Thelerpeton.oppressus
???
??
??
??????????????????00011100000011111100000011111?????????????????????????????????
??????????0100001111111???
??
???
Teratophon.spinigenis
???
??
??
??????????????????000111000000111111???
?????????0100001111111??
??
??
Eumetabolodan.bathycephalus
???
??
??
??????????????????000011000000111111???
??
??
??
Scoloparia.glyphanodon
???
??
??
??????????????????00000010011111111100001111111????????011111??????????????????
???????????000101111111101111111???
??
???
Hypsognathus.fenneri
???
??
??
??????????????????00000001111111111100011111110111111111111110111111??????????
?????????????001101111111111111111???
??
???
Soturnia.caliodon
???
??
??
??????????????????000000011111111111???
??
??
??

Leptopleuron.lacertinum
??
???
???
??????????????????0000000011111111100011111110111111111111111???????1111???????
????????????0011011111111111111111??
???
???
Neoprocolophon.asiaticus
??
???
???
??????????????????000000000011111111???
???
???
???
Phonodus.dutoitorum
??
???
???
??????????????????000000000011111111???
???
???
???
Pentaedrusaurus.ordosianus
??
???
???
??????????????????000000000011111111???
???
???
???
Tichvinskia.vjatkensis
??
???
???
??????????????????0000000000000111110000011111100011111000111001111110111??????
?????????????0000000111111001111111???
???
??
Phaantosaurus
??
???
???
??????????????????000000000000000111000000001111????????000111000111110011???????
????????????0000000011111??
???
??
Pintosaurus.magnidentes
??
???

???
??????????????????000000000000000011???????????????????000111?????????????????????
???
???
???
Kitchingnathus.utabeni
??
???
???
??????????????????000000000000000011??
???
???
???
Thelegnathus
??
???
???
???00111111001111??????????????????????
???
???
??
Koiloskiosaurus
??
???
???
???00011111?????????????????????????????
???
???
???
Bolosaurus.striatus
??
???
???
???10111??
???
???
???
Bolosaurus.grandis
??
???
???
???10111??
???
???
???
Belebey.chengi
??
???
???
???01111??
???

???
???
Belebey.maximi
??
???
???
???01111??
???
???
???
Anomoiodon
??
???
???
???
??????0000111111111??
???
??
Thalassiodracon.hawkinsi
??
???
???
???
??0000000011111110000000000000000011111111
1101100000000111?????????????????000000000000000001111??????????????????????????
??
Plesiosaurus.dolichodeirus
??
???
???
???
??0000000011111110000000000000000011111111
1110100000000111?????????????????000000011000000000111??????????????????????000
000000111111111??
Augustasaurus
??
???
???
???
??0000000000111110000000000000000011111111
1100000000000011???
???
Bobosaurus
??
???
???
???
??0000000000111110000000000000000011111111
11??
??

Pistosaurus

??
???
???
???
???00000000000111100000000000000000011111111
11??????????????011??????????????000000000000000000011??????????????????????00000
0000011111111????????????????????????????????????00000111110000000000011111111

Yunguisaurus

??
???
???
???
???00000000000011100000000000000000011111111
11??
??

Cymatosaurus

??
???
???
???
???00000000000001100000000000000000011111111
1100000000000001011??????????????00000000000000000001??????????????????????000
0001110011111111???

Corosaurus

??
???
???
???
???00000000000000100000000000000000011111111
11???0000000
01001111111???

Dactylosaurus

??
???
???
???
???00000000000000010000000000001111111111111
11??????????????001???1110000
00000011111???

Neusticosaurus.pusillus

??
???
???
???
???00000000000000010000000000001111111111111
11??????????????001??10011001000
000000011111???

Anarosaurus

??
???

???
???
??0000000000000001000000000000111111111111
11??????????????001???0110000
00000011111??
Sanchiaosaurus
??
???
???
???
??0000000000000000110000000011111111111111
11??
??
Kwangsisaurus
??
???
???
???
??0000000000000000110000000011111111111111
11??
??
Chinchenia
??
???
???
???
??0000000000000000010000000011111111111111
11??
??
Lariosaurus.curionii
??
???
???
???
??0000000000000000001000111111111111111111
11??
??
Lariosaurus.balsami
??
???
???
???
??0000000000000000001000111111111111111111
11??????????????111???0001111
11001111111??
Lariosaurus.xingyiensis
??
???
???
???
??0000000000000000000111111111111111111111

11???
??
Lariosaurus.calcagnii
??
??
??
??
??0000000000000000000111111111111111111111
11???
??
Lariosaurus.valceresii
??
??
??
??
??0000000000000000000111111111111111111111
11???
??
Lariosaurus.buzzi
??
??
??
??
??0000000000000000000011111111111111111111
11???
??
Nothosaurus.mirabilis
??
??
??
??
??0000000000000000000000011111111111111111
11??????????????111011111????????????????????????????????10000001?????????????00000
1111001111111??
Germanosaurus
??
??
??
??
??0000000000000000000000001111111111111111
11?????????????????000011???
??
Keichousaurus
??
??
??
??
??0000000000000000000000000111111111111111
11??????????????001???1110000
00000011111??

Diandongosaurus

??
???
???
???
??0000000000000000000000000011111111111111
11??
??

Dianopachysaurus

??
???
???
???
??0000000000000000000000000001111111111111
11??
??

Qianxisaurus

??
???
???
???
??0000000000000000000000000000011111111111
11??
??

Wumengosaurus

??
???
???
???
??0000000000000000000000000000001111111111
11??
??

Hanosaurus

??
???
???
???
??0000000000000000000000000000000111111111
11??????????????001??
??

CRYPTOCLIDIA

??
???
???
???
??10
100000000111?????????????????000001111000000000111??????????????????????????????
??

Pleisiopterys.wildi

??
???

???
???
??01
100000000111?????????????????000000001000000000111??????????????????????????????
??
Dolichorhynchops.osborni
??
???
???
???
??00
011111111111?????????????????001111111000000000111??????????????????????????????
??
Edgarosaurus.muddi
??
???
???
???
??00
011111111111?????????????????010111111000000000111??????????????????????????????
??
Polycotylus.latipinnis
??
???
???
???
??00
001111111111?????????????????010111111000000000111??????????????????????????????
??
Umoonasaurus.demoscyllus
??
???
???
???
??00
000111111111???
???
Leptocleidus
??
???
???
???
??00
000011111111?????????????????000000000011111111111??????????????????????????????
??
PLIOSAURIDAE
??
???
???
???
??00

000001111111?????????????????0000000000000001111111??????????????????????0000000
00111111111???
Macroplata.tenuiceps
???
??
??
??
???00
000000111111?????????????????000000000000011111111??????????????????????????????
???
Rhomalesaurus.megacephalus
???
??
??
??
???00
000000011111?????????????????000000000100011111111??????????????????????????????
???
Rhomalesaurus.victor
???
??
??
??
???00
000000001111?????????????????000000000100011111111??????????????????????????????
???
Neusticosaurus.peyeri
???
??
??
??
??
???????????001???
??
Neusticosaurus.edwardsi
???
??
??
??
??
???????????001???
??
Neusticosaurus.toeplitschi
???
??
??
??
??
???????????001???
??

Serpianosaurus
??
???
???
???
???
???????????001???001000000000
011111???????????????????????????????????00000011110000000000011111111
Nothosaurus.haasi
??
???
???
???
???
??????????????111111??
???
Nothosaurus.tchernovi
??
???
???
???
???
??????????????111111??
???
Nothosaurus.giganteus
??
???
???
???
???
??????????????011111??
???
Nothosaurus.jagisteus
??
???
???
???
???
??????????????011111??
???
Nothosaurus.yangiuanensis
??
???
???
???
???
??????????????001111??
???
Nothosaurus.youngi
??
???

??
??
??
??????????????001111???
??
Nothosaurus.edingerae
???
??
??
??
??
??????????????001111???
??
Nothosaurus.marchicus
???
??
??
??
??
??????????????001111???
??
Nothosaurus.winterswijkensis
???
??
??
??
??
??????????????001111???
??
Nothosaurus.juvenilis
???
??
??
??
??
??????????????000111???
??
Nothosaurus.winkelhorsti
???
??
??
??
??
??????????????000111???
??
Helveticosaurus
???
??
??
??
??

????????????????????00100011??
??00000000110000000000011111111
Eusaurosphargis
??
???
???
???
???
????????????????????00100011??
???
Saurosphargis
??
???
???
???
???
????????????????????00011111??
???
Sinosaurosphargis
??
???
???
???
???
????????????????????00011111??
???
Kimmerosaurus
??
???
???
???
???
????????????????????????????100011111000000000111?????????????????????????????????
???
Morturneria
??
???
???
???
???
????????????????????????????100011111000000000111?????????????????????????????????
???
Trinacromerum
??
???
???
???
???
????????????????????????????001111111000000000111?????????????????????????????????
???

Tricleidus
???
??
??
??
??
????????????????????????????000011111000000000111?????????????????????????????????
??
Microcleidus
???
??
??
??
??
????????????????????????????000000111000000000111?????????????????????????????????
??
ELASMOSAURIDAE
???
??
??
??
??
????????????????????????????000000111000000000111?????????????????????????????????
??
Simolestes
???
??
??
??
??
????????????????????????????000000000011111111111?????????????????????????????????
??
Rhomalesaurus.zetlandicus
???
??
??
??
??
????????????????????????????000000000001111111111?????????????????????????????????
??
Attenborosaurus
???
??
??
??
??
????????????????????????????000000000000000111111?????????????????????????????????
??
Eurycleidus
???
??

???
???
???
????????????????????????????000000000000000011111?????????????????????????????????
???
Henodus
??
???
???
???
???
???0100011110100011100111???????????
???
Placochelys
??
???
???
???
???
???0011111110011111100111???????????
???
Psephoderma
??
???
???
???
???
???0011111110011111100111???????????
???
Protenodontosaurus
??
???
???
???
???
???00011111100011111????????????????
???
Macroplacus
??
???
???
???
???
???00001111100001111????????????????
???
Paraplacodus
??
???
???
???
???

??0000000110000000101111???????????????
??
Cyamodus.kuhnschnyderi
??
??
??
??
??
???11000111????????????????????
??
Ceresiosaurus
??
??
??
??
??
???000111111001
111111???
Silvestorsaurus
??
??
??
??
??
???000011111001
111111???
Paranothosaurus
??
??
??
??
??
???000001111001
111111???
Askeptosaurus.italicus
??
??
??
??
??
??
????11000000111100000001000011011000000010001100000000000000000011111111
Anshuansaurus.huangguoshuensis
??
??
??
??
??
??
????1100000011110000000100011011100000001???????????????????????????????

Endennasaurus.acutirostris
??
???
???
???
???
???
????0100000011010000001?????00010000001?????????????????????????????
Clarazia.schinzi
??
???
???
???
???
???
????0011110111000110111001110000111101100111000000000000000011111111
Hescheleria.ruebeli
??
???
???
???
???
???
????00111101110001101110011100001111011?????????????????????????????
Paralonectes.merriami
??
???
???
???
???
???
????0000110111000110111?????00000011011?????????????????????????????
Agkistrognathus.campbelli
??
???
???
???
???
???
????0000010111000000011?????00000001011?????????????????????????????
Xinpusaurus.baomaolinensis
??
???
???
???
???
???
????00000011110000011111111100000000111?????????????????????????????
Nectosaurus.halius
??
???

???
???
???
???
????0000001111000110111111110000000111??????????????????????????????
Thalattosaurus.borealis
??
???
???
???
???
???
??????????????001010111??
Xinpusaurus.suni
??
???
???
???
???
???
??????????????000001111??
Miodentosaurus
??
???
???
???
???
???
?????????????????????????????10110000001??????????????????????????????
Anshuansaurus.wushaensis
??
???
???
???
???
???
?????????????????????????????01110000001??????????????????????????????
Saurosternon
??
???
???
???
???
???
??1000000000000000000001111111
Palaeagama
??
???
???
???
???

???
???????????????????????????????????????10000000000000000000001111111
Hupehsuchus
??
???
???
???
???
???
???????????????????????????????????????010010000000000000000011111111
Macrocnemus
??
???
???
???
???
???
???????????????????????????????????????00000000000001100000011111111
Pamelina
??
???
???
???
???
???
???????????????????????????????????????00000000000000011000111111111
Drepanosaurus
??
???
???
???
???
???
???????????????????????????????????????00000000000000001000111111111
Sophineta
??
???
???
???
???
???
???????????????????????????????????????00000000000000000011111111111
Marmoretta
??
???
???
???
???
???
???????????????????????????????????????00000000000000000001111111111

Orovenator
??
???
???
???
???
???
???????????????????????????????????????00000000000000000000000011111
Galesphyrus
??
???
???
???
???
???
???????????????????????????????????????00000000000000000000000011111
Thadeosaurus
??
???
???
???
???
???
???????????????????????????????????????00000000000000000000000011111
Kenyasaurus
??
???
???
???
???
???
???????????????????????????????????????00000000000000000000000011111
Tangasaurus
??
???
???
???
???
???
???????????????????????????????????????00000000000000000000000011111
outgroup
000000000000000000000000000000000000000000000000000000000000000000000
000000000000000000000000000000000000000000000000000000000000000000000000000
000000000000000000000000000000000000000000000000000000000000000000000000000
000000000000000000000000000000000000000000000000000000000000000000000000000
000000000000000000000000000000000000000000000000000000000000000000000000000
000000000000000000000000000000000000000000000000000000000000000000000000000
000000000000000000000000000000000000000000000000000000000000000000000000000
0000000000000000000000000000000000000000000000000000
;
end;

```
begin assumptions;
	options deftype=irrev;
end;

begin sets;
	charset Reiszetal2011 = 1-16;
	charset Sumidaetal2010 = 17-26;
	charset minisupertree7 = 27-46;
	charset minisupertree8 = 47-100;
	charset DeBraga&Reisz1995 = 101-107;
	charset Laurin&Reisz1997 = 108-109;
	charset Lee2001 = 110-136;
	charset Modesto&Reisz2002 = 137-146;
	charset Muller2003 = 147-167;
	charset Rieppel&DeBraga = 168-178;
	charset minisupertree5 = 179-197;
	charset minisupertree6 = 198-223;
	charset Cisnerosetal2004 = 224-230;
	charset Cisnerosetal2008 = 231-238;
	charset Lee1995 = 239-245;
	charset Lee1997 = 246-266;
	charset MacDougall&Modesto2010 = 267-284;
	charset Modesto&Damiani2007 = 285-295;
	charset Modesto&DamianiDeBraga = 296-303;
	charset Modesto&DamianiPineiro = 304-309;
	charset Modestoetal2001 = 310-316;
	charset Modestoetal2002 = 317-320;
	charset Modestoetal2003 = 321-325;
	charset Mulleretal2008 = 326-330;
	charset Reisz&Scott2002 = 331-339;
	charset Saila2008 = 340-352;
	charset Sues&Reisz2008 = 353-360;
	charset Tsuji2006 = 361-373;
	charset minisupertree10 = 374-415;
	charset Druckmiller&Russell2008 = 416-429;
	charset Holmesetal2008 = 430-432;
	charset Klein&Albers2009 = 433-438;
	charset Lietal2011 = 439-446;
	charset OKeefe2004 = 447-467;
	charset Rieppel2000a = 468-476;
	charset Rieppel2000b = 477-484;
	charset Rieppel&Zannon1997 = 485-489;
	charset Storrs1993 = 490-507;
	charset Muller2005 = 508-517;
	charset Liu&Rieppel2005 = 518-526;
	charset Jiangetal2003 = 527-531;
	charset Wuetal2009 = 532-542;
	charset minisupertree7 = 543-571;
end;
```

<u>Ichthyopterygia</u>
#NEXUS

begin data;
dimensions ntax=48 nchar=113;
format datatype=standard missing=?;
matrix
Mixosaurus.cornalianus
11001001100000000000000000011111001100000000000000000011111101000111101000000000000111110010000000000000000011111111
Mixosaurus.kuhnschnyderi
11001???01000111???
Mixosaurus.panxianensis
01001??
Phalarodon.fraasi
00111011100000000000000000011111011100000000000000000011111100111111101000000000000111110010000000000000000011111111
Phalarodon.callawayi
00111???00011111???
Contectopalatus.atavus
0001101110000000000000000001111101110000000000000000001111110000111111?????????????????0010000000000000000011111111
Grippia.longirostris
?????100000000000000000000000111100000000000000000000000000111000000001000000000000000011000000000000000000000000111111
Chaohusaurus
?????10000000000000000000000011110000000000000000000000000111?????????????????????????0000000000000000000000111111
Wimanius
?????0001000000000000000000011111000100000000000000000001111111???
Toretocnemus
?????00001000000000000000001111110000100000000000000000111111??????????????????????????0000000100000000000111111111
Qianichthyosaurus
?????00001000000000000000001111110000100000000000000000111111??????????????????????????0000000100000000000111111111
Cymbospondylus.petrinus
?????000001000000000000000111111100000100000000000000111111111000000110011000000000111110100000000000000000000001111111
Phantomosaurus
?????0000010000000000000001111111000001000000000000000111111111???
Leptonectes
?????0000001000111111111111111110000001000111111111111111111?????????0000011111111111110000000000011111111111111111

Eurhinosaurus
?????000000100011111111111111111000000100011111111111111111?????????000
000011111111111000000000001111111111111111
Stenopterygius
?????000000011111111111111111111000000011111111111111111111?????????000
011111111111110000000001001111111111111111
OPTHALMOSAURIA
?????000000011111111111111111111000000011111111111111111111?????????000
011111111111110000000011001111111111111111
Ichthyosaurus
?????000000001111111111111111111000000001111111111111111111?????????000
000111111111110000000011001111111111111111
Suevoleviathan
?????000000000111111111111111111000000000111111111111111111?????????000
0000001111111100000000000000111111111111111
Temnodontosaurus.platyodon
?????000000000001111111111111111000000000001111111111111111?????????000
000000001111110000000000000111111111111111
Macgowania
?????000000000000111111111111111000000000000111111111111111????????????
??????????????0000000000000000111111111111
Hudsonelpidia
?????000000000000111111111111111000000000000111111111111111????????????
??????????????0000000000000000111111111111
Callawayia
?????000000000000011111111111111000000000000011111111111111????????????
??????????????0001111000000000000111111111
Californosaurus
?????000000000000001111111111111000000000000001111111111111????????????
??????????????0000000000000000011111111111
Mikadocephalus.gracilirostris
?????000000000000001111111111111000000000000001111111111111100000011??
???
Shonisaurus
?????000000000000000011111111111000000000000000011111111111?????????000
100000000111110000111000000000000111111111
Shastasaurus
?????000000000000000001111111111000000000000000001111111111????????????
??????????????0001111000000000000111111111
Besanosaurus
?????000000000000000000111111111000000000000000000111111111?????????001
100000000111110000001000000000000111111111
Merriamosaurus
?????000000000000000000011111111000000000000000000011111111????????????
??
Parvinatator
?????000000000000000000000001111000000000000000000000001111????????????
??????????????0000000000000000000000001111
Utatsusaurus
?????000000000000000000000000011000000000000000000000000011?????????000
000000000001110000000000000000000000001111

Thaisaurus
?????00000000000000000000000000001000000000000000000000000000001????????????
??
Quasianosteosaurus
??????????????????????????????????0000000000000000000000011111?????????????????
??
Phalarodon.major
??00111111???????????
??????????????????????????????????????
Mixosaurus.maotaiensis
??00000111???????????
??????????????????????????????????????
Petrolacosaurus
??100000000
00000001??????????????????????????????
Araeoscelis
??100000000
00000001??????????????????????????????
Platypterygius
??000000001
11111111??????????????????????????????
Temnodontosaurus.burgundiae
??000000000
01111111??????????????????????????????
Hovasaurus
??
???????100000000000000000000000000011
Thadeosaurus
??
???????100000000000000000000000000011
Cymbospondylus.buchseri
??
???????010000000000000000000001111111
Guizhouichthyosaurus
??
???????000001100000000000000111111111
Excalibosaurus
??
???????000000000001111111111111111111
Xinminosaurus
??
???????000000000000000000000000011111
Hupesuchus
??
???????000000000000000000000000000111
Claudiosaurus
??
???????000000000000000000000000000001
outgroup
0000000000000000000000000000000000000000000000000000000000000000000000000
0000000000000000000000000000000000000000000000

;
end;

begin assumptions;
options deftype=irrev;
end;

begin sets;
charset Jiangetal2006 = 1-5;
charset Maisch&Matzke2003a = 6-32;
charset Maisch&Matzke2003b = 33-59;
charset Maisch&Matzke2005 = 60-68;
charset Sander2000 = 69-85;
charset minitree = 86-113;
end;

<u>Lepidosauromorpha</u>
#NEXUS

begin data;
dimensions ntax=46 nchar=86;
format datatype=standard missing=?;
matrix
Brachyrhinodon.taylori
100000000001110100000000011111000001111???????????????110000000000001111
110011100011111
Clevosaurus.hudsoni
100000000001110100000000011111000001111??????00011111111000000000000111
1100011110111111
Kallimodon
010000000111110010000001111111????????????????????????0001000000001111111
00011100001111
Sapheosaurus.thiolleri
010000000111110010000001111111000111111??????00111111100010000000111111
11000111????????
Sphenodon.punctatus
001100011111110000000111111111100011111??????00111111100000010001111111
1101011100001111
Cynosphenodon.huizachalensis
001100011111110000000111111111100011111???????????????00001110001111111
1010111????????
Zapatadon
0001000111111100000001111111111????????????????????????000011100011111111
000111????????
Eilenodon
0000111111111110000111111111111????????????????????????00000001111111111
001111????????
Priosphenodon
0000111111111111???00000001111111111??
????????????

Toxolophosaurus
00000111111111000011111111111?????????????????????????000000001111111111
001111????????
Opisthias
00000011111111000001111111111?????????????????????????000000000111111111
??????????????
Pamizinsaurus
00000000111111???0001
11????????
Palaeopleurosaurus.posidoniae
00000000001111000000000111111001111111???????????????0010000000011111
100011100001111
Homeosaurus.maximilliani
00000000000011000000111111111000001111???????001111110100000000000111
1100011100001111
Diphydontosaurus
00000000000010000000000000001000000111???????????????0000000000000011
100000100000011
Sphenocondor
??????????????100000000001111???
??????????
Godavarisaurus
??????????????100000000001111???
??????????
Theretairus
??????????????000100011111111???
??????????
Sphenovipera
??????????????000100011111111???
??????????
Kaikaifilusaurus
??????????????000011111111111???
??????????
Kawasphenodon
??????????????000001111111111???
??????????
Ankylosphenodon
??????????????000000111111111??0011
11????????
Rebbanasaurus
??????????????000000000000111???
??????????
Planocephalosaurus.robinsonae
??????????????000000000000011000000011????????????????0000000000000011110
0001100000111
Pleurosaurus.goldfussi
?????????????????????????????011111111????????????????0010000000011111???
???????????
Pleurosaurus.ginsburgi
?????????????????????????????011111111??
?????????

Polysphenodon.muelleri
?????????????????????????????000001111??????????????????????????????????1001
1100001111
Gephyrosaurus.bridensis
?????????????????????????????0000000110111111000011111000000000000000011
00000100000001
Youngina
?????????????????????????????0000000010000001110000001?????????????????????
???????????
SQUAMATA
?????????????????????????????0000000010111111000001111000000000000000001?
?????????????
Kuehneosaurus
??????????????????????????????????????1000111000000111??????????????????????
??????????
Paliguana
??????????????????????????????????????1000111000000011??????????????????????
??????????
Sophineta
??????????????????????????????????????0011111????????????????????????????????
?????????
Marmoretta
??????????????????????????????????????0001111????????????????????????????????
?????????
Prolacerta
??????????????????????????????????????0000011????????????????????????????????
?????????
Coelurosauravus
??????????????????????????????????????0000001????????????????????????????????
?????????
Saurosternon
??????????????????????????????????????0000001000000011??????????????????????
??????????
Tangasaurus
???110000001???????????????????????
?????????
Acerosodontosaurus
???010000001???????????????????????
?????????
Palaeagama
???000000001???????????????????????
?????????
Clevosaurus.wangi
??110000000000011111????
??????????
Oenosaurus
??000001100011111111????
??????????
Clevosaurus.petilius
??
10111111

Clevosaurus.bairdi
	??
01111111
Clevosaurus.mcgilli
	??
01111111
outgroup
	000000000000000000000000000000000000000000000000000000000000000000000
00000000000000000
;
end;

begin assumptions;
	options deftype=irrev;
end;

begin sets;
	charset Apesteguia&Novas2003 = 1-14;
	charset Apesteguiaetal2012 = 15-29;
	charset Dupret2004 = 30-38;
	charset Evans&BorsulBialynicka2009 = 39-45;
	charset Gauthieretal1988 = 46-54;
	charset Rauhautetal2012 = 55-72;
	charset Reynoso2000 = 73-78;
	charset Suesetal1994 = 79-86;
end;

<u>Synapsida</u>
#NEXUS

begin data;
	dimensions ntax=306 nchar=829;
	format datatype=standard missing=?;
matrix
Proburnetia
	111111001001000001111??
???
???
???
???
???????????????????111001????0000011111111????????????????????????????011111111??
????????????????????????????????????101111??
???
???
???
Burnetia
	111111001001000001111??
???
???
???

??
???????????????????111001????0000111111111???????????????????????????111111111??
????????????????????????????????????011111???
??
??
??
Lemurosaurus
011111001001000001111???
??
??
??
??
?????????????????????????????0000000111111?????????????????0000111111001111111??
????????????????????????????????????000111???
??
??
??
Lycaenodon
001111001001000001111???
??
??
??
??
?????????????????????????????0000000011111?????????????????0000001111000011111??
????????????????????????????????????000001???
??
??
??
Herpetoskylax
000111001001000001111???
???????????????????????110000000000000001111?????????????????????????????????????
??
??
????????????1100000000001???
??????????????????????????????????0000000011111?????????????????0000001111000011
111??????????????????????????????????????000001????????????????????????????????????
??
??
???
Hipposaurus
000011001001000001111???
??
??
??
??
???????????????????001001????0000000001111?????????????????0000000111000001111?
??
??
??
??

Biarmosuchus.tener
000001001001000001111??????????????????????????????0000000000000000000000
0000001100001111111??????010000000000000001111??????????????????????????????????
???????????????????????????0001111111???
??
????????01111111111100000000001???
???000000000111000000000000000111100000000
1100000111???
??
??
??
????????

Cyonosaurus
000000111001000001111??
??
??
??
??
??
??
??
??
??

Lycaenops
000000111001000001111??
??
??
??
??
??
??
??
??
??

Gorgonops.torvus
000000011001000001111??
???????????????????????110000000000000001111????????????????????000000000000000
0000000000000000000000001??????????00000000000000000000000000000000000000100
0000000000000001???
?????????????????????????010000000001??
?????????????????????????????????????000111????0000000000011000000000000111111??
???000000001??????????????
??
??
??
?????????????

Patranomodon
000000000111000001111??
???????????????????????000000000000111111111?????????????????????000000000000000
0000000000000000000111111??????????000000000000000000000000000000000001111???
???????????????00000000000000000000000000000000000000000000000000000000000000

00000000000000000011??????????000001110011??????????????????????000000001????
??
??0001000011???000000111????????
??
??
??
??????????????????
Suminia
000000000111000001111???
???????????????????????000000111000011111111????????????????????110000000000000
00000000000000000000011111??????????111000000000000000000000000000000000011111???
???????????????0100000000000000000000000000000000000000000000000000000000000000
00000000000000000111??????????001100111001??????????????????????????????????????
??
?0001000011??11000111??????????
??
??
??
??????????????????
Biseridens.qilianicus
00000000011000001111?????????????????????????????000000000000000000000
0000011100001111111??????00000000000000001111111????????????????????00000000000
000000000000000000000000111???
??
??????????????????????000000010011??
??
??
??
??
??
????
Syodon.biarmicum
000000000000110011111???
???????????????????????0011100000000000011111????????????????????????????????????
??
??
????????????0000000001111???
??????????????????????????????????1010000000001???????????????????1010000011???????
??????????????????????????????0111000111??
??
??
??
Titanophoneus.potens
000000000000110011111?????????????????????????????000000000000000000000
0000011100001111111??????0011100000000000011111????????????????????00000000000
0000000000000000000000000010001111111???
??
?????????????011111111100000000001111???000
1111??1010000000001??????????????????1010
000011??????????????????????????????????????0000111111????????????????????????????????
??

???
???
????????????
Sinophoneus.yumenensis
000000000000010011111???
???
???
???
???
???
??????????????????????0000001111???
???
???
???
Styracocephalus
000000000000001111111???
???
???
???
???
???????????????????????????0111???
???
???
???
??
Jonkeria
000000000000001111111???
???
???
???
???
??????????????????????????11110110000000001??????????????????0110000011???????????
???
???
???
???
Estemmenosuchus.uralensis
000000000000000111111???
????????????????????????0000100000000000011111???????????????????????????????????????
???
???
????????????00000000000111??
???????????????????????????????0011011000000000110000000000000111110110000011????
??????????????????????????????????1000000001??
???
???
???
??
Raranimus.dashankouensis
000000000000000000111???????????????????????????????00000000000000000000000
0000000100001111111??????0000000000000000000011????????????????????????????????????

??
??
??
??
??
??
??
??
Tetraceratops
000000000000000000011??
???????????????????????000000000000000000001??????????????????????????????????????
??
??
??
??
??
??
??
??
Dimetrodon.limbatus
000000000000000000001??
??
????????????????????1110111111???
??
?1011111111??1111111?????????
??00000000010000000
01???
??
??
??
Prosictodon
?????????????????????111000000000000000000000001111???????????????????????
??
??
???000011100000010000000000000000000000000000000000000000000000000000000011
1111111111???
??
??
??
??
??
??
Diictodon
?????????????????????111000000000000000000000001111???????????????????????
????????????????????????000000000111111111110000000000000000001111001000000000
0000000000000000001111111111?????????00010000000000000000000000000001111111
1??????????????????0000001000000100000000000000000000000000000000000000000000
0000000000001111111111111???1001111111
000000011???
?????????????????????????0000000000000101????????????????????????????001111111???
??

???
???
????????????????????????
Robertia
?????????????????????0110000000000000000000000001111???????????????????????
????????????????????????0000000001111111111100000000000000000111100100000000000
0000000000000000001111111111??????????0001000000000000000000000000000000111111
1??????????????????000011100000010000000000000000000000000000000000000000000
000000000000111111111111??1001111110
000000001???
?????????????????????????00000000000001101??
???
???
???
???????????????????????
Eosimops
?????????????????????00100000000000000000000001111???????????????????????
???
???
???000001100000010000000000000000000000000000000000000000000000000000000011
1111111111???
???
???
???
???
???
??
Chelydontops
?????????????????????000100000000000000000000011111???????????????????????
??0001110000000000
000000000000011111111111??????????000011100000000000000000000001111111111????
??????????????0000000000000000000000000000000000000000000000000000000000000000
0000000000111111111??011111111???????
???
???
???
???
???
?????????????
Endothiodon
?????????????????????000100000000000000000000011111???????????????????????
???00000000000000001001100011000000000
00000000000000011111111111??????????000011100000000000000000000000011111111?
?????????????????000000000000000000000000000000000000000000000000000000000000
0000000000001111111111??
???
???
???
???
???
???????????????

Dicynodontoides
??????????????????????0000110011000000000000000111111????????????????????????
???000000000000000110011000000000100110
0000000000000001111111111111??????????0000000001001100000000000000001111111111?
?????????????????000000010001110000000000000000000000000000000000000000000000000
00000000001111111111111??
???
?????????????????????000000000000111101???
???
???
???
???????????????????
Kombuisia.frerensis
??????????????????????0000110011000000000000000111111????????????????????????
???0000000110011000
0000000000000111111111111111??????????0000000110011000000000000011111111111????
??????????????000000010001110000000000000000000000000000000000000000000000000000
000000011111111111111??
???
???
???
???
???
????????????
Myosaurus
??????????????????????0000010011000000000000001111111????????????????????????
???0000000000001111001100000000000001110
0000000000000001111111111111??????????0000000000011100000000000000001111111111?
?????????????????000000000011110000000000000000000000000000000000000000000000000
00000000001111111111111??
???
???
???
???
???
????????????????
Cistecephaloides
??????????????????????0000001111000000000000001111111????????????????????????
???0000000001111000
0000000000000111111111111111??????????0000000001111000000000000011111111111????
??????????????000000011111100000000000000000000000000000000000000000000000000000
000000011111111111111??
???
???
???
???
???
????????????
Kawingasaurus
??????????????????????0000001111000000000000001111111????????????????????????
???0000000001111000

000000000000011111111111?????????000000000111100000000000000001111111111????
??????????????00000000111111000000000000000000000000000000000000000000000000
0000000111111111111??
??
?????????????????0000000000000011??
??
??
??
???????????????
Cistecephalus
??????????????????????0000000111000000000000001111111???????????????????????
???00000000000001111001100000000011110
000000000000001111111111111?????????000000000111100000000000000001111111111?
?????????????????00000000011111000000000000000000000000000000000000000000000000
00000000001111111111111??0000
000111??
??????????????????????00000000000000011???
??
??
??
????????????????????
Emydops.arctatus
??????????????????????00000000010000000000000001111111???????????????????????
???0000001000001000
000000000000011111111111111?????????000000010000010000000000000001111111111????
??????????????000000000000110000000000000000000000000000000000000000000000000
0000000111111111111??001111111???????
??
??
??
??
??
?????????????
Tropidostoma
??????????????????????00000000000110000110000111111111???????????????????????
???0000000000000100
0011000000001111111111111?????????00000000000000100001100000001111111111????
??????????????00000000000001100000001110000000000000000000000000000000000000
000000111111111111??
??
?????????????????00000000001111101??
??
??
??
???????????????
Australobarbarus
??????????????????????00000000000110000110000111111111???????????????????????
???0000000000000100
0011000000001111111111111?????????00000000000000100001100000001111111111????
??????????????00000000000001100000001110000000000000000000000000000000000000
000000111111111111??

???
???
???
???
???
????????????
Oudenodon.bainii
?????????????????????000000000001000011000011111111???????????????????????
???00000000111110011001100000000000001
00001100000001111111111111??????????000000000000000100001100000001111111111?
?????????????????000000000000000100000001110000000000000000000000000000000000
000000000111111111111??
???
????????????????????00000001111111101??
???
???
??011000000010001?????
????????????????????
Pelanomodon.tuberosus
?????????????????????000000000000111111000011111111???????????????????????
???00000000111110011001100000000000000
01111100000001111111111111??????????000000000000000011111000000011111111111?
?????????????????000000000000000101111110000000000000000000000000000000000000
000000000111111111111??
???
????????????????????00000000011111101??
???
???
??000100000110111?????
????????????????????
Geikia.locusticeps
?????????????????????000000000000111111000011111111???????????????????????
??0000000000000011
11110000000011111111111111??????????00000000000000011111100000001111111111????
??????????????000000000000000000111111000000000000000000000000000000000000
000000111111111111???
???
???
???
???
??1111????????????
?????????????
Aulacephalodon.bainii
?????????????????????000000000000111111000011111111???????????????????????
???00000000011110011001100000000000000
00111100000001111111111111??????????000000000000000001111000000011111111111?
?????????????????000000000000000101111110000000000000000000000000000000000000
000000000111111111111??
???
????????????????????0000000011111101??
???

??
???0001000001110011?????
????????????????????
Idelesaurus
?????????????????????000000000000000111100001111111???????????????????????
???0000000000000000
011100000001111111111111??????????0000000000000000000111000000011111111111????
??????????????0000000000000000010000011110000000000000000000000000000000000000
0000001111111111111???
??
??
??
??
??
????????????
Odontocyclops.dubius
?????????????????????000000000000000011100001111111???????????????????????
??
??
???0000000000000000100000111100000000000000000000000000000000000000000000000111
1111111111??
??
??
??
??
???000000001111?????????????????????????
????
Rhachiocephalus.magnus
?????????????????????000000000000000000100001111111???????????????????????
??00000000000000111001100000000000000
00000100000001111111111111?????????0000000000000000000010000000111111111111?
?????????????????00000000000000000100011110000000000000000000000000000000000000
0000000001111111111111??
??
??
??
??
??111000000001????????????
?????????????????
Lystrosaurus.murrayi
?????????????????????000000000000000000111111111111???????????????????????
??00000001000010011001100000000000000
00000001011111111111111111?????????0000000000000000000001011111111111111111?
?????????????????00000000000000000000000000000001001111100000000000000000000000
0011111111111111111111??0000
111111???
??????????????????????00000111111111101???
??
??
??01110011????????????????000010011111??????
??????00000000000000011

Dicynodon.trautscholdi
?????????????????????000000000000000000111111111111?????????????????????????
???000000000000000
0000001111111111111111111??????????00000000000000000000000111111111111111????
??????????????00000000000000000000000000000000000000000000000000000000000000
001111111111111111??
??
??
??
??
???0000
0000000000111
Dicynodon.amalitzkii
?????????????????????000000000000000000111111111111?????????????????????????
???000000000000000
0000001111111111111111111??????????00000000000000000000000111111111111111????
??????????????000000000000000000000000000110000000000000000000000000000000000
111111111111111111??
??
??
??
??
??
????????????
Kannemeyeria.simocephala
?????????????????????000000000000000000111111111111?????????????????????????
??001111110000100110011000000000000000
000000010111111111111111111??????????0000000000000000000000101111111111111111?
?????????????????00000000000000000000000000000000000000000000000111100000000111
111111111111111111111??0001
111111??
???????????????????????0011001111111101???
??
??
???0001000000000111000001111111????0
0000001000000000000111111
Dicynodon.lacerticeps
?????????????????????000000000000000000111111111111?????????????????????????
??0000000000111001100110000000000000000
0000000000111111111111111111??????????0000000000000000000000000111111111111111?
?????????????????000000000000000000000000001000000000000000000000000000000000000
0000111111111111111111???0000
011111??00000000000001
11111???001111111??????
??
??
???0000000000000001000000111111????
????????00000000000000001
Delectosaurus
?????????????????????000000000000000000111111111111?????????????????????????
???000000000000000

000000000111111111111111?????????000000000000000000000000000011111111111????
??????????????000000000000000000000000000000000000000000000000000000000000000
000111111111111111???
???
???
???
???
???
????????????
Katumbia.parringtoni
?????????????????????0000000000000000000011111111????????????????????????
???
???
???00000000000000000000000000000000000000000000000000000000000000000000001111
1111111111??
???
???
???
???
???
??
Interpresosaurus
?????????????????????000000000000000000000011111111????????????????????????
???0000000000000000
0000100000111111111111111?????????0000000000000000000001000001111111111111????
??????????????000000000000000000000000000000000000000000000000000000000000000
00000111111111111111???
???
???
???
???
???
????????????
Elph
?????????????????????000000000000000000000011111111????????????????????????
???0000000000000000
0000100000111111111111111?????????0000000000000000000001000001111111111111????
??????????????000000000000000000000000000000000000000000000000000000000000000
00000111111111111111???
???
???
???
???
???
????????????
Pristerodon
?????????????????????000000000000000000000000111111????????????????????????
???0000000000000000001110000010000000
000000000000000111111111111?????????0000001000000000000000000000000011111111?
????????????????0000000000000000000000000000000000000000000000000000000000000
00000000000111111111111???011111111100

0000011???
???????????????????????00000000000011101???????????????????????????001111111?????
???
???
???
??????????????????????
Lanthanostegus
?????????????????????000000000000000000000000000111???????????????????????
??0000110000000000
000000000000011111111111??????????000001100000000000000000000000011111111????
??????????????0000000000000000000000000000000000000000000000000000000000000000
000000000011111111??
???
???
???
???
???
????????????
Colobodectes
?????????????????????0000000000000000000000000000011???????????????????????
??000000000000000
0000000000000001111111111??????????000000000000000000000000000000001111111????
??????????????00010000000000000000000000000000000000000000000000000000000000000
0000000000001111111??000011111???????
???
???
???
???
???
?????????????
Eodicynodon.oosthuizeni
?????????????????????0000000000000000000000000000001???????????????????????
????????????????????????00000000001111111111100000000000000000000010000000000000
000000000000000000011111111??????????00000000000000000000000000000000011111
1??????????????????00010000000000000000000000000000000000000000000000000000000
000000000000000001111111??????????0000111110011??????????????????????000001111
???
???000111111??????
???
???
???
?????????????????????
Captorhinus.aguti
??1000000000000000000000000
00000000000000001??
???
???
???0000001?????????????????????????????????????
?000000000000000000011??
???
???

???
???
Protorothyris.archeri
???100000000000000000000000
00000000000000001???
???
???
???
???
???
???
???
???
Ophiacodon.mirus
???011100000000001000000000
00000000000000011001111???
???????????????????????0000000001??
???
????0000000001?????????????????????00000001????????????????????????????????000010
001110100000011000000000001??
???????????????????????10000000011???
???
???
???
???
Stereophallodon.ciscoensis
???011100000000001000000000
00000000000000011???
???
???
???
???
???
???
???
???
Varanosaurus.acutirostris
???001100000000001000000000
00000000000000011001111???
???
???
???
???
???
???
???
???
Archaeothyris.florensis
???000100000000001000000000
00000000000000011000111???
???

???
???0000001000010001
110100000011000000000001??
???????????????????10000000011??
???
???
??
Mesenosaurus.romeri
???000011000001111000000000
00000000000000011???
???
???
??000001111
1100000111110000000000001??
???????????????????01100001111???
???
???
??
Heleosaurus.scholtzi
???000011000001111000000000
00000000000000011???
???
???
???
???
??????????????00100001111??
???
???
???
Mycterosaurus.longiceps
???000001000001111000000000
00000000000000011000001???
???
???
?????????????????????????10000001????????0000111?????????????????????????00000111
1110000011111000000000001??
?????????????????????01100001111???
???
???
???
?
Watongia.meieri
???000000110011110000000000
00000000000000011???
???
???
???
???
??????????????00011111111??
???

??
??
Varanodon.agilis
??000000110011111000000000
0000000000000011???
??
??
??
0011111111000000000001??
?????????????????00011111111??
??
??
???
Varanops.brevirostris
??000000010011111000000000
0000000000000011???
??
??
?????????????????????????10000001???00000011
1110011111111000000000001??
?????????????????????00001111111???
??
??
??
?
Aerosaurus.wellesi
??000000001111110000000000
0000000000000011???
??
??
??
??
??
??
??
??
Aerosaurus.greenleeorum
??000000001111110000000000
0000000000000011???
??
??
??
0001111111000000000001??
?????????????????00000111111??
??
??
???
Ruthiromia.elcobriensis
??000000000111111000000000
0000000000000011???
??

???
???
???
???
???
???
???
Pyozia.mesenensis
???000000000000111000000000
00000000000000011???
???
???
???
???
??????????????00000000111??
???
???
???
???
Archaeovenator.hamiltonensis
???000000000000011000000000
00000000000000011???
???
???
??000000011
1100000001110000000000001???
???????????????????00000000111???
???
???
??
Eothyris.parkeyi
???000000000000000100000001
00000000000000111???
???
???
?????????????????????????00000011????????1000011??????????????????????????00110000
0111000000001000000000001??
?????????????????????00000000001???
???
???
???
?
Oedaleops.campi
???000000000000000100000001
00000000000000111???
???
???
??001100000
11??
???
???

??
???
Cotylorhynchus.hancocki
???0000000000000000011111111
000000000000000111???
??
??
??
??
??
??
??
??
Cotylorhynchus.bransoni
???0000000000000000011111111
000000000000000111???
??
??
??
??
??
??
??
??
Cotylorhynchus.romeri
???0000000000000000011111111
000000000000000111???
??
??
??????????????????????????01111111????????1000011???????????????????????????11010000
011??
???????????????????00000000001??
??
??
??
Angelosaurus.romeri
???0000000000000000011111111
000000000000000111???
??
??
??
??
??
??
??
??
Angelosaurus.dolani
???0000000000000000011111111
000000000000000111???
??
??

?????????????????????????01111111???
???
???
???
???
??
Ennatosaurus.tecton
???000000000000000000111111
00000000000000111???
???
???
?????????????????????????00111111???
???
???
???
???
??
Euromycter.rutena
???000000000000000000011111
00000000000000111???
???
???
?????????????????????????00011111???11010000
011???
???
???
???
???
Trichasaurus.texensis
???000000000000000000001111
00000000000000111???
???
???
???
???
???
???
???
???
Casea.broilii
???000000000000000000000111
00000000000000111???
???
???
?????????????????????????00001111???01010000
0111000000001000000000001??
?????????????????????00000000001???
???
???
???
?

Oromycter.dolesorum
???000000000000000000000011
00000000000000111???
???
???
?????????????????????????00000111???
???
???
???
???
??
Edaphosaurus.boanerges
???000000000000000000000000
11100000000001111???
???
???
?????????????????????????????????01111111???
???
???
???
???
??
Edaphosaurus.novomexicanus
???000000000000000000000000
11100000000001111???
???
???
?????????????????????????????????00011111???
???
???
???
???
??
Lupeosaurus.kayi
???000000000000000000000000
01100000000001111???
???
???
?????????????????????????????????00001111???
???
???
???
???
??
Glaucosaurus.megalops
???000000000000000000000000
01100000000001111???
???
???
?????????????????????????????????000011110111111???????????????????????????????????
???

??
??
??
??
Ianthasaurus.hardestii
??000000000000000000000000
00100000000001111010011??
??
??
?????????????????????????????????000001110011111???????????????????0000011????????
??
??
??
??
???
Dimetrodon.milleri
??000000000000000000000000
00000011111111111??
??
??
??
??
??
??
??
??
Sphenacodon
??000000000000000000000000
00000011111111111110011??
???????????????????????1110111111???
??
????1011111111??0011111??????
???000000000000000111??????????????
??
??
??
??
Cryptovenator.hirschbergeri
??000000000000000000000000
00000001111111111??
???????????????????????0110111111???
??
??
??
??
??
??
??
Secodontosaurus.obtusidens
??000000000000000000000000
00000000111111111??

???????????????????????0010111111??
??
???1111111????????
??
??
??
??
???
Cutleria.wilmarthi
??000000000000000000000000
00000000011111111???
???????????????????????0000011111??
??
????0001111111??
??
??
??
??
??
Pantelosaurus.saxonicus
??000000000000000000000000
00000000000111111???
???????????????????????0000001111??
??
????0000111111??
??
??
??
??
??
Haptodus.garnettensis
??000000000000000000000000
00000000000011111110011??
???????????????????????0000000111??
??
????0000000111?????????????????????000000110001111???????????????????0000111?????
??000000000000000011?????????????
??
??
??
??
?
Ianthodon.schultzei
??000000000000000000000000
000000000000001111???
???????????????????????0000001111??
??
????0000011111??
??
??
??

???
???
Tapinocaninus.pamelae
??
????????????????????000110000000000011111???
???
???
???
?????????????????????????1111???
???????????????????????1000000001??
???
???
???
Tiarajudens
??
????????????????????000001000000001111111???
???
???
???
???
???
???
???
??
Anomocephalus
??
????????????????????000001000000001111111?????????????????????000000000000000000
0000000000000000001111??????????000000000000000000000000000000000000111??????
????????????0000000000000000000000000000000000000000000000000000000000000000
000000000000000001??????????0000000110011??
???
???000000011??????????????
???
???
???
?????????????
Ulemica
??
????????????????????000000111000011111111?????????????????????110000000000000000
0000000000000000011111??????????111000000000000000000000000000000011111??????
????????????1100000000000000000000000000000000000000000000000000000000000000
000000000000000111??????????0011001110011??
???
???110001111??????????????
???
???
???
?????????????
Otsheria
??
????????????????????000000011000011111111?????????????????????010000000000000000

0000000000000000011111??????????0110000000000000000000000000000000011111??????
????????????110000000000000000000000000000000000000000000000000000000000000000
000000000000000111??????????0001001110011???????????????????????????????????????
??
???010001111?????????????
??
??
??
?????????????
Galechirus
???
????????????????????000000001000011111111?????????????????????00000000000000000
0000000000000000111111???
???????001000000000000000000000000000000000000000000000000000000000000000000
0000000001111??
??
??
??
??
??
?????
Galeops
???
????????????????????000000000001111111111?????????????????????00000000000000000
00000000000000001111111??????????001000000000000000000000000000000011111??????
????????????0000000000000000000000000000000000000000000000000000000000000000
000000000000011111??????????0000111110011??????????????????????000000011???????
??
???000011111?????????????
??
??
??
??????????????
Lycosuchus.vanderrieti
???
????????????????????000000000000000000111?????????????????????00000000000000000
0000000000000000000011??????????00000000000000000000000000000000000001100000
0000000001111??
??
??
??000000011000000000000000000000
000011????????????????????????????????000000000000010000000000000000000001?????
?????????????????????????????????000000000001??????????????????????????????????000
00001???
???????????????
Ischigualastia
???
???111111110000100110011????????????????????
??
??0000000000000000000000000000000000000000000000000000111111111111111111111
111111111??

??
?????1111001111111101???
??
??
???????????????????????????????0000011000001111????????????????0001111000000001
11111111
Stahleckeria
???
???111111110000100110011????????????????????
??
??0000000000000000000000000000000000000000000000000001011111111111111111111
111111111??011111111????????
??
???????1111001111111101???
??
??
?????????????????????????????????0000101000001111????????????????0010111100000011
111111111
Dolichuranus
???
???011111110000100110011????????????????????
??
??0000000000000000000000000000000000000011000100000000000000011111111111111
111111111??
??
??
??
??
???000111000011111
11
Shansiodon
???
???000111110000100110011????????????????????
??
??0000000000000000000000000000000000000011100000000000000000011111111111111
111111111??
??
??
??
??
????????????????????????????1110000000000011????????????????10000001110000000000
01111
Vinceria
???
???000011110000100110011????????????????????
??
??0000000000000000000000000000000000000010110000000000000000011111111111111
111111111??
??
??
??

??
????????????????????????????0010000000000011????????????????????????0100000000000
1111
Tetragonias
???
???000001110000100110011????????????????????
??
??000000000000000000000000000000000000000000101100000000000000111111111111
111111111??
??
?????00010011111111101???
??
??
???????????????????????????????111000000000011???????????????1000000111000000
00001111
Dinanomodon.rubidgei
???
???000000110000100110011????????????????????
??
??
??
??
??
??
??
?????????????????????????????????000001111111????????????????????????????
Daptocephalus.leoniceps
???
???000000000001100110011????????????????????
??
??000000000000000000000000000010000000000000000000000000000000000011111111111
111111111??
??
??
??
??
??
Emydops.oweni
???
???00000010000010000000
00000000111111111111??????????000000010000010000000000000011111111111????????
??
??
??
??
??
??
???
Kombuisia.antarctica
???
???00000001100110000000

00000000111111111111??????????0000000011001100000000000000001111111111????????
???
???
???
???
???
???
??
Geikia.elginensis
??
??00000000000000111111
00000001111111111111??????????0000000000000000011111000000001111111111????????
??????????00000000000000000000111111100000000000000000000000000000000000000000
00011111111111111??
???
???
???
???
???
??1111????????????????
?????????
Galepus
??
??00000000000000000000
00000000000000111111??000000000000
00001100100000000000000000000000000000000000000000000000000000000000000000000
0000000001111???
???
???
???
???
???
?????
Ctenospondylus
??
???
?????????????????1110111111???
???
??0111111??????????????
???
???
???
???
??
Palaeohatteria
??
???
?????????????????0000001111???
???0
000001111???
???
???

???
???
???
Edaphosaurus.cruciger
??
???
?????????????????0000000011??
???0
000000011?????????????????????11111110111111?????????????????????????????????????
???000000000000000001???????????????????
???
???
???
??
Dvinia.prima
??
???
??1100000000000001
11??
???
???000000001111111111?????????????????????
???100000000000000000001111111???
?????????????????????????????000100000011110000000000000000000011???????????????
??00000011??????
???
???
Procynosuchus.delaharpeae
??
???
??1100000000000001
11??
???
???000000001111111111?????????????????????
???100000000000000000001111111???
?????????????????????????????000100000011110000000000000000000011???????????????
???????????????????????00000000011???????????????????????????????????00000111????
???
?????
Charassognathus.gracilis
??
???
??0100000000000001
11??
???
???
???
??????????????????????00000000001111???
???
???
Glanosuchus.macrops
??

???
??0010000000000011
11???
???
???
??000000000000000000000000111??????
???
???
???
Ictidosaurus.angusticeps
??
???
??0010000000000011
11???
???
???
???
???
???
???
Pristerognathus.polyodon
??
???
??0010000000000011
11???
???
???
???????????????????????????????????????000000011????????????????????????????????????
???
???
??
Promoschorhynchus.platyrhinus
??
???
??0001010000000111
11???
???
???
???
???
???
???
Olivierosuchus.parringtoni
??
???
??0001010000000111
11???
???
???
???
??????????????????????01100000000011???

???
???
Cerdops.burgheri
??
???
??0000110000000111
11??
???
???
???
???
???
???
Moschorhinus.kitchingi
??
???
??0000110000000111
11??
???
???
??000000000000000000000011111??????
??????????????????????????00100000000011???
???
???
Euchambersia.mirabilis
??
???
??0000010000000111
11??
???
???
???
???
???
???
Moschowhaitsia.vjuschkovi
??
???
??0000001100001111
11??
???
???
???
???
???
???
Theriognathus.microps
??
???
??0000001100001111
11??

??
??
???000000000000000000000111111??????
??????????????????????????00000000000111??
??
??
Viatkosuchus.sumini
???
??
???0000000100001111
11???
??
??
??
??
??
??
Tetracynodon.tenuis
???
??
???0000000011111111
11???
??
??
??
??
??
??
Tetracynodon.darti
???
??
???0000000011111111
11???
??
??
??
??
??
??
Choerosaurus.dejageri
???
??
???0000000001111111
11???
??
??
??
??
??
??

Lycideops.longiceps

???
???
??0000000000111111
11??
???
???
???
???
???
??

Ictidosuchus.primaevus

???
???
??0000000000011111
11??
???
?????????????????????000111??????????????????000000000011111111???????????????????????
??000000011???????????????????????????????????
???
???
??

Ictidosuchops.rubidgei

???
???
??0000000000011111
11??
???
???
??0000000000000000000000011111??????
???????????????????????????011000000000011???
???
???

Ichtidosuchoides.longiceps

???
???
??0000000000011111
11??
???
???
???
???
???
??

Regisaurus.jacobi

???
???
??0000000000011111
11??
???
???

???
??????????????????????11100000000011???
???
???
Scaloposaurus.constrictus
??
???
??0000000000011111
11??
???
???
???
???
???
???
Ericiolacerta.parva
??
???
??0000000000011111
11??
???
???
???
???
???
???
Bauria
??
???
??0000000000011111
11??
???
???
??000000000000000000000011111??????
??????????????????????????11100000000011???
?????????????????000000000001??
???
Ictidostoma.hemburyi
??
???
??0000000000001111
11??
???
???
???
???
???
???
Hofmeyria.atavus
??
???

??0000000000001111
11??
???
???
??00000000000000000000000001111??????
???
???
???
Mirotenthes.digitipes
??
???
??0000000000001111
11??
???
???
???
???
???
???
Kitchinganomodon
??
???
??0
000000000000000000000010001111100000000000000000000000000000000000000001111111
111111??
???
???
???
???
???
??
Daqingshanodon.limbus
??
???
??0
000000000000000000000001100000000000000000000000000000000000000000000001111111
111111??
???
???
???
???
???
??
Keyseria.benjamini
??
???
??0
000000000000000000000000100000000000000000000000000000000000000000000000001111111
111111??
???
???
???

???
??
Dicynodon.huenei

??
???
??0
0000000000000000000000000100000000000000000000000000000000000000111111111111
111111??
???
???
???
???
??
Dinanomodon.gilli

??
???
??0
0000000000000000000000000110000000000000000000000000000000000001111111111111
111111??
???
???
???
???
??
Sintocephalus.alticeps

??
???
??0
0000000000000000000000000000110000011100000000000000000000000011111111111111
111111??
???
???
???
???
??
Basilodon.woodwardi

??
???
??0
0000000000000000000000000000110000011100000000000000000000000011111111111111
111111??
???
???
???
???
??
Euptychognathus.bathyrhynchus

??
???
??0
0000000000000000000000000000010000011100000000000000000000000011111111111111

111111???
??
??
??
??
???
Lystrosaurus.declivis
???
??
???0
0000000000000000000000000000010011111000000000000000000000001111111111111
111111???
??
??
??
??
????????????????00110011??
Lystrosaurus.hedini
???
??
???0
0000000000000000000000000000001111110000000000000000000000001111111111111
111111???
??
??
??
??
????????????????00000111??
Kwazulusaurus.shakai
???
??
???0
0000000000000000000000000000001111110000000000000000000000001111111111111
111111???
??
??
??
??
????????????????00000001???????????????000010011111???????????????????????????
Lystrosaurus.maccaigi
???
??
???0
0000000000000000000000000000001111110000000000000000000000001111111111111
111111???
??
??000001111111111101??
??
??
????????????????????01110011???
?

Lystrosaurus.curvatus
???
??
???0
00000000000000000000000000000000000111100000000000000000000000011111111111111
111111???
??
??00001111111111101???
??
??
?????????????????????10000001??
?
Syops.vanhoepeni
???
??
???0
00000000000000000000000000000000000001100000000000000000000000011111111111111
111111???
??
??
??
??
???
Jimusaria.sinkianensis
???
??
???0
00000000000000000000000000000000000000100000000000000000000000011111111111111
111111???
??
??
??
??
???
Rechnisaurus
???
??
???0
00000000000000000000000000000000000001100010000000000000000011111111111111111
111111???
??
??
??
??
??????????????????????????0000000011111111?????????????????????????0000110000111111
1
Dinodontosaurus
???
??
???0
00000000000000000000000000000000000000001000100000000000000001111111111111111

111111???
???
??11110011111111101???
???
???
??????????????????????????????000000000011111???????????????00000011000001000011
11111
Rhinodicynodon
??
???
??0
000000000000000000000000000000000000000011100000000000000111111111111111111
111111???
???
??00010011111111101???
???
???
????????????????????????????0110000000000011???????????????????????????????????????
??
Kannemeyeria.lophorhina
??
???
??0
000000000000000000000000000000000000000000011100000000011111111111111111111
111111???
???
???
???
???
??
Rabidosaurus
??
???
??0
000000000000000000000000000000000000000000011100000000011111111111111111111
111111???
???
???
???
???
??
Parakannemeyeria.dolichocephala
??
???
??0
000000000000000000000000000000000000000000001100000000011111111111111111111
111111???
???
???
???
???

?????????0011111????????0000000100111111????????????????0100000100111100001111
111
Uralokannemeyeria
???
???
??0
00000000000000000000000000000000000000000000000000000000010000000011111111111111111111
111111???
???
???
???
???
???
Placerias
???
???
??0
000000000000000000000000000000000000000000000000000000000010000011111111111111111111111
111111??011111111????????????
???
???01110011111111101???
???
???
??????????????????????????????0000011000001111????????????????000001110000000011
111111
Moghreberia
???
???
??0
000000000000000000000000000000000000000000000000000000000010000011111111111111111111111
111111???
???
???
???
???
???
Rhadiodromus
???
???
??0
000000000000000000000000000000000000000000000000000000000001000111111111111111111111111
111111???
???
???
???
???
???????????????????????????000000000111111???
Wadiasaurus
???
???
??0

00000000000000000000000000000000000000000000000000100011111111111111111111111
111111???0011111111????????????
???
???01110011111111101??
???
???
?????????????????????????????0001000000000111????????????????0000000100000000000
011111
Sangusaurus
??
???
??0
00000000000000000000000000000000000000000000000000101111111111111111111111111
111111??
???
???
???
???
?????????????????????????0000000011111111???
Jachaleria
??
???
??0
000000000000000000000000000000000000000000000000000111111111111111111111111
111111??
???
???
???
???
???000111110000000111111111
1
Sinokannemeyeria.pearsoni
??
???
??0
000000000000000000000000000000000000000000000000000000011111111111111111111
111111??
???
???
???
???
??????????0100011????????0000000100111111????????????????0100000100111100001111
111
Xiyukannemeyeria.brevirostris
??
???
??0
000000000000000000000000000000000000000000000000000000000111111111111111111
111111??
???
???

???
???
??????????0000001???
Angonisaurus
???
???
??0
0000000000000000000000000000000000000000000000000000000000000011111111111111111
111111???
???
??0001001111111101???
???
???
??????????????????????????????0000101000001111????????????????00101111000000111111
11111
Turfanodon.bogdaensis
???
???
??0
0000000000000000000000000000000000000000000000000000000000000000111111111111111
111111???
???
???
???
???
??
Gordonia.traquairi
???
???
??0
0000000000000000000000000000000000000000000000000000000000000000000000111111111
111111???
???
???
???
???
??
Eodicynodon.oelofseni
???
???
??0
0000000000000000000000000000000000000000000000000000000000000000000000000000000
111111???000000111???????????????????????
???
???
???
???
???
Edaphosaurus.pogonias
???
???

???
???
???????????????????????????11111111???
???
???
???
???
??
Edaphosaurus.colohistion
??
???
???
???
???????????????????????????00111111???
???
???
???
???
??
Kingoria
??
???
???
???
???0000001111??????????????????????
???
???
???
???
???
Petrolacosaurus
??
???
???
???
??000000000010000
000000100000001111??
???
???
???
??
Elliotsmithia
??
???
???
???
???00001
11111000000000001???
????????????00000011111??
???

???
???
Apsisaurus
??
???
???
???
???00000
01111000000000001???
???
???
???
???
Araeoscelis
??
???
???
???
???00000
00000100000001111???
???
???
???
???
Acerosodontosaurus
??
???
???
???
???00000
00000010000111111???
???
???
???
???
Youngina
??
???
???
???
???00000
00000010000111111???
???
???
???
???
Trilophosaurus.buettneri
??
???
???
???

??00000
0000000110111111???
??
??
??
???
Prolacerta
???
??
??
??
??00000
0000000110111111???
??
??
??
???
Hyperodapedon
???
??
??
??
??00000
0000000010111111???
??
??
??
???
Planocephalosaurus
???
??
??
??
??00000
0000000001111111???
??
??
??
???
SQUAMATA
???
??
??
??
??00000
0000000001111111???
??
??
??
???

Coelurosauravus
???
??
??
??
??00000
000000000000011111???
??
??
??
???
Paleothyris
???
??
??
??
??00000
000000000000000111???
??
??
??
???
Bullacephalus
???
??
??
??
??
????????????????011001????0001111111111????????????????????????????111111111?????
???????????????????????????????????011111???
??
??
??
Australosyodon.nyaphuli
???
??
??
??
??
????????????????000011??
????????????????????0111000111???
??
??
??
Anteosaurus.magnificus
???
??
??
??
??
??????????????????????0001?????????????1000000000000011111??????????000000011?????

???????????????????????0000111111???
??
??
??
Pachydectes
???
??
??
??
??
??????????????????????????0001111111111???
???????????????????????????????011111??
??
??
???
Lobalopex
???
??
??
??
??
??????????????????????????0000001111111?????????????????0000111111?????????????????
????????????????????????????????000111???
??
??
??
Sinoconodon
???
??
??
??
??
??011111111111111111?????????????????????????
??000000000111111111111111111???????
??101111111011110011111
1000000011???????????????????01000000111110000011100110000011111???????????????
?????0000011111???
????
Morganucodon
???
??
??
??
??
??011111111111111111?????????????????????????
??000000000111111111111111111???????
??????0000001011111111111??????????????00111110000000000111110111111111111111
111111000000111011111100100111111101000000111110000011100110000011111???????
?????????????0000111111??
????????????

Pachygenelus
???
??
??
??
??
???00111111111111111??????????????????????????
??000000010011111111111111111???????
??????0001000000000111111??????????????0011110000000000111101111111110001100
01111100000000010011111000011111111000011111111100000011000011011111111???????
?????????????0000000111??
????????????
Probainognathus
???
??
??
??
??
???00011111111111111??????????????????????????
??00000000000001111111111111???????
??????0000000000000000111??????????????0001111000000000111110000011110000100
00000100000000010001111000000011111000000000000111????????00000000000011???????
????????????0000000011??
???????????
Probelesodon
???
??
??
??
??
???00001111111111111??????????????????????????
??
??000000000000001111???????????????0100111000000000011111???????????????????????
?????????1000111????????????1000000000011????????0100000000111???????????????????
??
Cynognathus
???
??
??
??
??
???00000111111111111??????????????????????????
??00000110000000001111111111???????
??????00100000000000000010000001111111000000000000000011111100000001???????
????????????????0000010000000011111??????????????????????1000000000001????????000
0000000110000000001??
?????????
Thrinaxodon
???
??
??

???
???
???????????????????????????????????????000000111111111111?????????????????????????
???000000000000000000111111111???????
?????????????????????????00000101111111000000000000000000001111?????????00001????
????????????????????000000001111???????????????????????????????????00011111000000
000001??
????
Galesaurus
??
???
???
???
???
???????????????????????????????????????000000011111111111?????????????????????????
???010000000000000000011111111???????
?????????????????????????000010001111110000000000000000000111???????????????????
???????????????????000000000111???????????????????????????????????11001111????????
???
?
Ictidorhinus
??
???
???
???
???
???0000011111000111111??????
????????????????????????????????000011??
???
???
???
Zambiasaurus
??
???
???
???
???
??00110011
111111101???
???
???
???
Lystrosaurus.georgi
??
???
???
???
???
??00001111
111111101???
???

??
???
Dicynodon.trigonocephalus
??
??
??
??
??
???00000001
111111101??
??
??
???
Ulemosaurus.svijagensis
??
??
??
??
??
??
???????????????????1000000001???
??
??
???
Estemmenosuchus.mirabilis
??
??
??
??
??
??
???????????????????1000000001???
??
??
???
Notosyodon.gusevi
??
??
??
??
??
??
???????????????????0011000111???
??
??
???
Pampaphoneus.biccai
??
??
??
??

??
??
???????????????????0001000111???
??
??
???
Titanophoneus.adamanteus
???
??
??
??
??
??
???????????????????0000011111???
??
??
???
Microsyodon
???
??
??
??
??
??
???????????????????0000000011???
??
??
???
Archaeosyodon.praeventor
???
??
??
??
??
??
???????????????????0000000011???
??
??
???
Paraburnetia
???
??
??
??
??
??
?????????????????????????????101111??
??
??
??

Niuksenitia
??
???
???
???
???
???
?????????????????????????????011111???
???
???
??
Lophorhinus
??
???
???
???
???
???
?????????????????????????????000111???
???
???
??
Progalesaurus
??
???
???
???
???
???
??010000000000000000011111111??????????
??????????????????????00001000111111???
??11001111????????????????
??
Massetognathus
??
???
???
???
???
???
??001111100000000011111111111000001111
1111?????????????????????????????????0000000000111111111111??????????????????????
?????????0000011010000011111???0000001111
11??
Exaeretodon
??
???
???
???
???
???

??0011111000000000111111111110011111111
1111111000000000000000001??????????????000000001111111111111100000111??????????
????????????????????0100000111110000000001111000000001100000000000001????????0001
1111111110000000001???
????????
Trirachodon
??
??
??
??
??
??
??0001111000000000111111111101000000000
0011?????????????????????????????????0000000000000011111111????????????????????????
???0011111100000000111
1??
Diademodon
??
??
??
??
??
??
??00001110000000001111111111000000000
0001?????????????????????????????????0000000000000001111111??????????????0000001
1????????????????00000001111??0000000001
110000000001??
?
Ecteninion
??
??
??
??
??
??
??000000100000000011111111111??????????
???????????????????????????????????0000011000000000011111????????????????????????
???????0001111100000011111???
???
Oligokyphus
??
??
??
??
??
??
??00000001100111111111111111??????????
???1110000000000000000001??????????????000000101111111111111?????????00111000011
1100000000101111110001111111111?????????????00000011?????????????????????????????
?????0000001111??
????

Kayentatherium
???
??
??
??
??
??
???000000011001111111111111111??????????
??
???????????000111111111???
??
Brasilitherium
???
??
??
??
??
??
???000000000011111111111111111??????????
???0000010000011111111???
??????????????0010011111110010000111111???
???
Brasilodon
???
??
??
??
??
??
???000000000000111111111111111??????????
???0000010000011111111???
??????????????0000001111110010000111111???
???
Chiniquodon
???
??
??
??
??
??
???000000000000001111111111111??????????
???0000000000000011111??????????????1100111000000000011111000000111???????????
???????????10001110000000111111000000000011????????01000000000111?????????????
??
???
Lumkuia
???
??
??
??
??

???
???000000000000000111111111111??????????
???000000000000000000110000001111111000000100000000001111100000000011??????????
??????????????????????00000001111100000000000001????????0000000000000100111111?????
???
????
Platycraniellus
???
???
???
???
???
???
???0000000000000000111111111??????????
???
??00011111??????????????????
???
Cynosaurus
???
???
???
???
???
???
???0000000000000000000011111111??????????
??????????????????????????000000000011111???
??01001111??????????????????
???
Prorubidgea
???
???
???
???
???
???
???00000000000000000000000001??????????
???
???
??
Cricodon
???
???
???
???
???
???
???110000000001
1??
???
???

Langbergia
???
??
??
??
??
??
??110000000001
1???
??
??
Menadon
???
??
??
??
??
??
??001111111111
1???
???000011111111???????
??
Scalenodontoides
???
??
??
??
??
??
??000111111111
1???
??????????????????????????????????????00000001?????????????????????????000111111111??????
???
Gomphodontosuchus
???
??
??
??
??
??
??000011111111
1?????????????????????????????????????0000000011111111111111?????????????????????????????
??000001111111????
???
Santacruzodon
???
??
??
??
??
??

???000000111111
1??
??000000111111???????
???
Dadadon
??
???
???
???
???
???
???000000011111
1??
??000000111111???????
???
Andescynodon
??
???
???
???
???
???
???000000001111
1??
??100000011111???????
???
Scalenodon.hirschsoni
??
???
???
???
???
???
???000000000111
1?????????????????????????????????0000000101111111111111??????????????????????????
???000000111111????
??
Pascualgnathus
??
???
???
???
???
???
???000000000111
1011000000000000001?????????????000000000000111111111???????????????????????
??100000011111?
???
Traversodon
??
???

???
???
???
???
???000000000111
1???0000111???????
???000000111111??????
??
Luangwa
??
???
???
???
???
???
???000000000011
1?????????????????????????????????0000000000011111111111??????????????????????????
?????????????????????????????????????00000001??????????????????????010000111111???
???
Scalenodon.angustifrons
??
???
???
???
???
???
???000000000011
1?????????????????????????????????0000000000011111111111??????????????????????????
?????????????????????????????????????00000001??????????????????????010000111111???
???
Riograndia
??
???
???
???
???
???
??0
001000000000111111????????????????????????????????????011111111??????????????????
???????????????????????0001001111111????????000000001111111???????????????????????
???
Prozostrodon
??
???
???
???
???
???
??0
000100000001111111????????????????????????????????????000011111??????????????????

???????????????????????0000000011111????????00010000011111???????????????????????
???
Therioherpeton
??
???
???
???
???
???
??0
000100000001111111????????????????????????????????????000111111??????????????????
???????????????????????0001001111111????????00000000001111???????????????????????
???
Hadrocodium
??
???
???
???
???
???
??0
000001011111111111??000011
111???
???
Dinnetherium
??
???
???
???
???
???
??0
000000111111111111??01111111?????
????????????????????????????????????00000111??????????????????????????????????00001
11111??
Kuehneotherium
??
???
???
???
???
???
??0
000000111111111111??01111111?????
????????????????????????????????????00001111??????????????????????????????????00001
11111??
Megazostrodon
??
???
???
???

??
??
???0
000000001111111111??1111111111111?????
??
??
Adelobasileus
???
??
??
??
??
??
???0
000000000111111111??11111011111110000
00011??
??
Nanictosaurus
???
??
??
??
??
??
??
??
??????????????????00000101111111??
??
??
Aleodon
???
??
??
??
??
??
??
??
?????????????????????????????????1100111000000000011111?????????????????????????????
??
???
Haldanodon
???
??
??
??
??
??
??
??1111101111111000001
111??
??

MONOTREMATA
??
???
???
???
???
???
???
???11111????????????????
?????????????????????????????????00011111??????????????????????????????????00111111
11??
MULTITUBERCULATA
??
???
???
???
???
???
???
???11111????????0001111
11????????????????????????????????01111111??????????????????????????????????100111
1111??
PLACENTA
??
???
???
???
???
???
???
???11111????????1111111
11????????????????????????????????11111111??????????????????????????????????011111
1111??
Triconodon
??
???
???
???
???
???
???
??011111110001111
11????????????????????????????????00111111???
???
MARSUPIALIA
??
???
???
???
???
???

???
??11111111
1????????????????????????????????11111111??????????????????????????????????0111111
111??
Vincelestes
??
???
???
???
???
???
???
??01111111
1??00111111
11??
Zhangheotheirum
??
???
???
???
???
???
???
??00111111
1??
??
Ornithorhynchus
??
???
???
???
???
???
???
??00011111
1??
??
Trucidocynodon
??
???
???
???
???
???
???
???
???????100000011111??
???
Irajatherium
??
???

???
???
???
???
???
???
???????????????????0000111111111????????00000101111111???????????????????????????
???
Diarthrognathus
??
???
???
???
???
???
???
???
???
???????????????????0000111111111????????00001101111111???????????????????????????
???
Elliotherium
??
???
???
???
???
???
???
???
???
???????????????????0000111111111????????00000011111111???????????????????????????
???
Chaliminia
??
???
???
???
???
???
???
???
???
???????????????????0000011111111????????00000011111111???????????????????????????
???
Phascolotherium
??
???
???
???
???
???
???
???

????????????????????????????????00111111??
??
Peramus
??
???
???
???
???
???
???
???
????????????????????????????????00111111??
??
Trioracodon
??
???
???
???
???
???
???
???
????????????????????????????????00111111??
??
Spalacotherium
??
???
???
???
???
???
???
???
????????????????????????????????00111111??
??
DRYOLESTIDA
??
???
???
???
???
???
???
???
????????????????????????????????00011111??
??
Ambolotheirum
??
???
???
???

???
???
???
???
????????????????????????????????00011111??
??
Amphitheirum
??
???
???
???
???
???
???
???
????????????????????????????????00011111??
??
HARAMIYIDAE
??
???
???
???
???
???
???
???
????????????????????????????????00000011??????????????????????????????????10011111
11??
Bolotridon
??
???
???
???
???
???
???
???
??00001111??????????????????????
??
Boreogomphodon
??
???
???
???
???
???
???
???
??001000111111?????????
???

Arctotraversodon
??
???
???
???
???
???
???
???
??001000111111?????????
???
Nanogomphodon
??
???
???
???
???
???
???
???
??001000111111?????????
???
Sinokannemeyeria.yingchiaoensis
??
???
???
???
???
???
???
???
??1
100011???
Sinokannemeyeria.sanchuanheensis
??
???
???
???
???
???
???
???
??1
100011???
Parakannemeyeria.shenmuensis
??
???
???
???
???
???

??
??
???0
011111??
Parakannemeyeria.youngi
???
??
??
??
??
??
??
??
???0
001111??
Parakannemeyeria.ningwuensis
???
??
??
??
??
??
??
??
???0
000111??
Lystrosaurus.platyceps
???
??
??
??
??
??
??
??
??
??????10000001??
Lystrosaurus.oviceps
???
??
??
??
??
??
??
??
??
??????00010011??
Lystrosaurus.robustus
???
??

??
??
??
??
??
??
??
??????00001111??
Lystrosaurus.shichanggouensis
???
??
??
??
??
??
??
??
??
??????00001111??
Lystrosaurus.broomi
???
??
??
??
??
??
??
??
??
??????00000111??
Lystrosaurus.youngi
???
??
??
??
??
??
??
??
??
??????00000001??
Rhachiocephalus.crassus
???
??
??
??
??
??
??
??

???
??????????????????????????????11100000001??????????????????????????????
Cteniosaurus.platyceps
	???
???
???
???
???
???
???
???
???
??????????????????????????????00100000001??????????????????????????????
Propelanomodon.devilliersi
	???
???
???
???
???
???
???
???
???
??????????????????????????????00000000011??????????????????????????????
outgroup
	000000000000000000000000000000000000000000000000000000000000000000000
000000000000000000000000000000000000000000000000000000000000000000000000000
000000000000000000000000000000000000000000000000000000000000000000000000000
000000000000000000000000000000000000000000000000000000000000000000000000000
000000000000000000000000000000000000000000000000000000000000000000000000000
000000000000000000000000000000000000000000000000000000000000000000000000000
000000000000000000000000000000000000000000000000000000000000000000000000000
000000000000000000000000000000000000000000000000000000000000000000000000000
000000000000000000000000000000000000000000000000000000000000000000000000000
000000000000000000000000000000000000000000000000000000000000000000000000000
000000000000000000000000000000000000000000000000000000000000000000000000000
0000000000
;
end;

begin assumptions;
	options deftype=irrev;
end;

begin sets;
	charset Amson&Laurin2011 = 1-21;
	charset Angielczyk&Rubidge2012 = 22-51;
	charset Benson2012 = 52-92;
	charset Bermanetal1995 = 93-98;
	charset Cisnerosetal2011 = 99-119;
	charset Damianietal2007 = 120-140;

```
	charset Frobisch&Reisz2011 = 141-180;
	charset Frobischetal2010 = 181-190;
	charset Frobischetal2010b = 191-229;
	charset Sigurdsenetal2012 = 230-247;
	charset Kammereretal2011 = 248-329;
	charset Kissel&Reisz2004 = 330-339;
	charset Liuetal2009b = 340-352;
	charset Maddinetal2008 = 353-360;
	charset Mayierski&Reisz2010 = 361-368;
	charset Modesto1994 = 369-375;
	charset Modestoetal2003 = 376-384;
	charset Ray2006 = 385-394;
	charset Reiszetal1992 = 395-401;
	charset Reiszetal2009 = 402-412;
	charset Rieszetal2010 = 413-434;
	charset Rubidge&Kitching2003 = 435-440;
	charset Rubidge&vandenHeever1997 = 441-444;
	charset Rubidgeetal2006 = 445-457;
	charset Sidor&Hopson1998 = 458-475;
	charset Sidor&Rubidge2006 = 476-485;
	charset Sidor&Welman2003 = 486-494;
	charset Surkovetal2005 = 495-511;
	charset minisupertree1 = 512-522;
	charset minisupertree2 = 523-532;
	charset minisupertree3 = 533-538;
	charset minisupertree4 = 539-547;
	charset Abdala2007 = 548-574;
	charset Abdalaetal2006 = 575-587;
	charset Bonaparteetal2005 = 588-606;
	charset Bothaetal2007 = 607-620;
	charset Hopson&Kitching2001 = 621-642;
	charset Liu&Olson2010 = 643-651;
	charset Lucas&Luo1993 = 652-656;
	charset Luo1994 = 657-664;
	charset Luoetal2001 = 665-673;
	charset Martinezetal1996 = 674-680;
	charset Oliveiraetal2010 = 681-692;
	charset Oliveira2006 = 693-705;
	charset Rowe1988 = 706-713;
	charset Sidor&Hancox2006 = 714-727;
	charset Sidor&Smith2004 = 728-735;
	charset Sues&Hopson2010 = 736-747;
	charset Wible1991 = 748-757;
	charset Liu&Li2003 = 758-764;
	charset Liuetal2002 = 765-772;
	charset Maisch2001 = 773-788;
	charset Maisch2002 = 789-800;
	charset Maisch&Gebauer2005 = 801-804;
	charset Vegadiasetal2004 = 805-812;
	charset Govender&Yates2009 = 813-829;
end;
```

Testudines

```
#NEXUS

begin data;
	dimensions ntax=11 nchar=17;
	format datatype=standard missing=?;
matrix
OTHER.CHELONIA 11111111011110111
Proterochersis.robusta		11111111111110111
Australochelys.africanus	01111111000111011
Palaeochersis.talampayensis	01111111001111011
Proganochelys.quenstedti	00111101000010001
Odontochelys.semitestacea	000101???????????
Simosaurus	0001?????????????
Anthodon.serrarius	0001?????????????
Owenetta.kitchingorum		0001?????????????
MEGAPLEURODIRA		????????11111????
outgroup	00000000000000000
;
end;

begin assumptions;
	options deftype=irrev;
end;

begin sets;
	charset minisupertree9 = 1-4;
	charset Anquetin2012 = 5-6;
	charset Anquetinetal2009 = 7-8;
	charset Gaffneyetal2007 = 9-13;
	charset Rougieretal1995 = 14-17;
end;
```

Supplementary Data 3
Supertree in nexus format

#NEXUS
begin trees;
tree tree1 = [&r] ((Limnoscelis.paludis, (Tseajaia.campi, (Ambedus.pusillus, (Oradectes.sanmiguelensis, (Orobates.pabsti, (Sepeliodon.hesperis, (Silvadectes.absitus, (Diadectes.tenuitectes, Diasparactus.zenos, Diadectes.sideropelicus)))))))), (((Archaeothyris.florensis, (Varanosaurus.acutirostris, (Stereophallodon.ciscoensis, Ophiacodon.mirus))), ((Oedaleops.campi, Eothyris.parkeyi), (Oromycter.dolesorum, (Casea.broilii, (Trichasaurus.texensis, (Euromycter.rutenus, (Ennatosaurus.tecton, (Angelosaurus.dolani, Angelosaurus.romeri, Cotylorhynchus.romeri, (Cotylorhynchus.bransoni, Cotylrohynchus.hancocki)))))))), (Archaeovenator.hamiltonensis, (Pyozia.mesenensis, Apsisaurus.witteri, ((Mycterosaurus.longiceps, Heleosaurus.scholtzi, Mesenosaurus.romeri), (Elliotsmithia.longiceps, ((Ruthiromia.elcobriensis, (Aerosaurus.greenleeorum, Aerosaurus.wellesi)), (Varanops.brevirostris, (Varanodon.agilis, Watongia.meiri))))))), ((Ianthasaurus.hardestii, (Glaucosaurus.megalops, Lupeosaurus.kayi, (Edaphosaurus.novomexicanus, (Edaphosaurus.colohistion, (Edaphosaurus.boanerges, (Edaphosaurus.pogonias, Edaphosaurus.cruciger)))))), (Haptodus.garnettensis, (Palaeohatteria.longicaudata, (Ianthodon.schultzei, (Pantelosaurus.saxonicus, (Cutleria.wilmarthi, ((Secodontosaurus.obtusidens, (Cryptovenator.hirschbergeri, (Ctenospondylus.spp, Sphenacodon.spp, Dimetrodon.milleri, Dimetrodon.limbatus))), (Tetraceratops.insignis, (Raranimus.dashankouensis, (((Biarmosuchus.tener, (Hipposaurus.boonstrai, (Herpetoskylax.hopsoni, (Lycaenodon.longiceps, (Ictidorhinus.martinsi, (Lophorhinus.willodensis, Lobalopex.mordax, Lemurosaurus.pricei, ((Paraburnetia.sneeubergensis, Proburnetia.viatkensis), (Niuksenitia.sikbonensis, Pachydectes.elsi, Bullacephalus.jacksoni, Burnetia.mirabilis)))))))), (Aloposaurus.spp, (Cyonosaurus.spp, (Aelurosaurus.spp, (Sauroctonus.spp, (Scylacognathus.spp, (Gorgonops.torvus, (Galesuchus.gracilis, (Njalila.spp, (Lycaenops.spp, (Arctognathus.spp, (Inostrancevia.spp, (Aelurognathus.spp, ((Sycosaurus.spp, Clelandina.spp), Rubidgea.spp))))))))))))), (((Estemmenosuchus.mirabilis, Ulemosaurus.svijagensis, Tapinocaninus.pamelae, Estemmenosuchus.uralensis, Jonkeria.truculenta, Styracocephalus.platyrhynchus), (Archaeosyodon.praeventor, Microsyodon.orlovi, (Sinophoneus.yumenensis, (Titanophoneus.adamanteus, (Anteosaurus.magnificus, Titanophoneus.potens)), (Pampaphoneus.biccai, (Notosyodon.gusevi, (Australosyodon.nyaphuli, Syodon.biarmicum)))))), ((Lycosuchus.vanderrieti, ((Pristerognathus.polyodon, Ictidosaurus.angusticeps, Glanosuchus.macrops), (((Moschorhinus.kitchingi, Cerdops.burgheri), (Olivierosuchus.parringtoni, Promoschorhynchus.platyrhinus), (Bauria.cynops, Ericiolacerta.parva, Scaloposaurus.constrictus, Regisaurus.jacobi, Ichtidosuchoides.longiceps, Ictidosuchops.rubidgei, Ictidosuchus.primaevus, (Lycideops.longiceps, (Choerosaurus.dejageri, (Tetracynodon.darti, Tetracynodon.tenuis))))), ((Viatkosuchus.sumini, (Theriognathus.microps, Moschowhaitsia.vjuschkovi)), (Bolotridon.frerensis, Cynosaurus.longiceps, Charassognathus.gracilis, Procynosuchus.delaharpeae, Dvinia.prima, (Progalesaurus.lootbergensis, Galesaurus.planiceps), ((Nanictosaurus.rubidgei, Thrinaxodon.spp), (Platycraniellus.elegans, ((Cynognathus.spp, (Diademodon.tetragonus, ((Trirachodon.spp, (Langbergia.modisei, Cricodon.metabolus)), ((Pascualgnathus.polanskii, Andescynodon.mendozensis), (Scalenodon.angustifrons, Luangwa.spp, Scalenodon.hirschsoni, Dadadon.isaloi, Santacruzodon.hopsoni, Massetognathus.spp, (Nanogomphodon.wildi, Arctotraversodon.plemmyridon, Boreogomphodon.jeffersoni),

(Gomphodontosuchus.brasiliensis, (Scalenodontoides.macrodontes, Menadon.besairiei, Exaeretodon.spp))))))), (Lumkuia.fuzzi, (Trucidocynodon.riograndensis, Ecteninion.lunensis), ((Probelesodon.spp, (Aleodon.brachyrhamphus, Chiniquodon.spp)), (Probainognathus.jenseni, ((Kayentatherium.wellesi, Oligokyphus.spp), (Therioherpeton.carnegini, Prozostrodon.brasiliensis, Brasilodon.quadrangularis, Brasilitherium.riograndensis, (Riograndia.guaibensis, (Chaliminia.musteloides, Elliotherium.kersteni, (Irajatherium.hernandezi, OTHER.TRITHELODONTIDAE))), (Sinoconodon.rigneyi, (Adelobasileus.cromptoni, (Megazostrodon.rudnerae, ((Kuehneotherium.praecursoris, Dinnetherium.nezorum), (Morganucodon.watsoni, OTHER.MAMMALIA)))))))))))))))))), (Biseridens.qilianicus, ((Anomocephalus.africanus, Tiarajudens.eccentricus), (Patranomodon.nyaphulii, ((Galepus.jouberti, Galechirus.scholtzi), (Otsheria.netzvetajevi, (Ulemica.spp, Suminia.getmanovi)), (Galeops.whaitsi, (Eodicynodon.oelofseni, (Eodicynodon.oosthuizeni, (Colobodectes.cluveri, ((Eosimops.newtoni, Robertia.broomiana, Diictodon.feliceps, Prosictodon.dubei), ((Pristerodon.mackayi, (Lanthanostegus.mohoii, (Endothiodon.spp, Chelydontops.altidentalis))), (((Emydops.oweni, Emydops.arctatus), ((Dicynodontoides.spp, (Kombuisia.antarctica, Kombuisia.frerensis)), (Myosaurus.gracilis, (Cistecephalus.microrhinus, (Kawingasaurus.fossilis, Cistecephaloides.boonstrai))))), ((Keyseria.benjamini, (Daqingshanodon.limbus, ((Rhachiocephalus.magnus, Kitchinganomodon.crasus), ((Oudenodon.bainii, (Australobarbarus.spp, Tropidostoma.microtrema)), (Odontocyclops.whaitsi, Idelesaurus.tataricus, (Aulacephalodon.bainii, (Geikia.elginensis, Geikia.locusticeps))))))), ((Elph.borealis, Interpresosaurus.blomi), (Katumbia.parringtoni, (Gorgonia.traquairi, (Delectosaurus.arefjevi, ((Dicynodon.huenei, Dicynodon.lacerticeps), ((Vivaxosaurus.trautscholdi, (Daptocephalus.leoniceps, (Dinanomodon.gilli, Peramodon.amalitzkii))), ((Jimusaria.sinkianensis, (Syops.vanhoepeni, ((Euptychognathus.bathyrhynchus, (Basilodon.woodwardi, Sintocephalus.alticeps)), ((Lystrosaurus.platyceps, Lystrosaurus.georgi, Lystrosaurus.curvatus), (Kwazulusaurus.shakai, (Lystrosaurus.broomi, Lystrosaurus.hedini, (Lystrosaurus.shuchanggouensis, Lystrosaurus.robustus)), (Lystrosaurus.oviceps, (Lystrosaurus.maccaigi, Lystrosaurus.declivis, Lystrosaurus.murrayi))))))), (Turfanodon.bogdaensis, Dinanomodon.rubidgei, ((Vinceria.argentinensis, (Rhinodicynodon.gracile, Tetragonias.njalilus, Shansiodon.spp)), ((Rabidosaurus.cristatus, (Kannemeyeria.lophorhina, Kannemeyeria.simocephala)), (Wadiasaurus.indicus, (((Moghreberia.nmachouensis, Placerias.spp), (Angonisaurus.cruickshanki, Ischigualastia.jenseni, Jachaleria.spp, Stahleckeria.potens)), ((Sangusaurus.edentatus, Rhadiodromus.klimovi), ((Dinodontosaurus.pedroanum, (Rechnisaurus.cristarhynchus, Dolichuranus.spp)), ((Sinokannemeyeria.pearsoni, (Sinokannemeyeria.sanchuanheensi, Sinokannemeyeria.yingchiaoensis)), (Parakannemeyeria.ningwuensis, (Parakannemeryeria.youngi, (Parakannemeyeria.shenmuensis, Parakannemeyeria.dolichocephala)), ((MESOSAURIDAE, (((Eunotosaurus.africanus, Milleretta.rubidgei), (Broomia.perplexa, (Millerosaurus.nuffieldi, (Millerosaurus.ornatus, Milleropsis.pricei)))), (Microleter.mckinzieorum, (Australothyris.smithae, ((Feeserpeton.oklahomensis, (Lanthanosuchus.watsoni, (Colobomycter.pholeter, Acleistorhinus.pteroticus))), ((Eudibamus.cursoris, ((Bolosaurus.grandis, Bolosaurus.striatus), (Belebey.chengi, Belebey.vegrandis, Belebey.maximi))), ((Nyctiphruretus.spp, ((Tokosaurus.perforatus, Macroleter.poezicus), (Bashkyroleter.mesensis, Bashkyroleter.bashkyricus, Nycteroleter.spp, Emeroleter.levis, Rhipaeosaurus.spp))), ((Bradysaurus.seeleyi, (Bradysaurus.baini, Nochelesaurus.schwarzi, (Embrithosaurus.schwarzi, (Deltavjatia.mesensis, ((Provelosaurus.americanus, (Pumiliopareia.pricei, Anthodon.serrarius)), ((Shansisaurus.xuecunensis,

(Shihtienfenia.permica, (Pareiasuchus.peringueyi, Pareiasuchus.nasicornis))), (Pareiasaurus.serridens, (Pareiasaurus.geinitzi, ((Sanchuansaurus, Scutosaurus.kapinskii), (Elginia.mriabilis, Arganaceras.vacanti)))))))))), ((Owenetta.rubidgei, Saurodectes.rogersorum, Barasaurus.besairiei, (Candelaria.barbouri, Owenetta.kitchingorum)), (Coletta.seca, ((Kitchingnathus.utabeni, Sauropareion.anoplus), (Pintosaurus.magnidentes, (Phaanthosaurus.simus, ((Theledectes.perforatus, Eumetabolodan.dongshengensis), (Tichvinskia.vjatkensis, ((Timanophon.raridentatus, Anomoiodon.liliensterni, Thelophon.contritus, Kapes.spp), (Eumetabolodan.bathycephalus, (Teratophon.sprinigensis, Procolophon.trigoniceps, Thelerpeton.opressus)), (Pentaedrusaurus.ordosianus, (Phonodus.dutoitorum, Neoprocolophon.asiaticus, ((Scoloparia.glyphanodon, Sclerosaurus.armatus), (Leptopleuron.lacertinum, (Soturnia.caliodon, Hypsognathus.fenneri))))))))))))))))))))), (Coelostegus.prothales, ((Thuringothyris.mahlendorffae, (Concordia.cunninghami, ((Romeria.prima, Romeria.texana), (Protocaptorhinus.pricei, (Reiszorhinus.olson, (Rhiodenticulatus.heatoni, (Saurorictus.australis, ((Captorhinus.laticeps, (Captorhinus.aguti, Captorhinus.magnus)), (Captorhinikos.spp, (Labidosaurus.hamatus, (Labidosaurikos.meachami, (Moradisaurus.grandis, (Gansurhinus.qingtoushanensis, Rothianiscus.spp))))))))))))), (Brouffia.orientalis, (Paleothyris.acadiana, ((Spinoaequalis.schultzei, (Petrolacosaurus.kansensis, Araeoscelis.spp)), (Claudiosaurus.germaini, Coelurosauravus.spp, Longisquama.insignis, ((Acerosodontosaurus.piveteaui, (Tangasaurus.mennelli, Youngina.capensis)), (Lanthanolania.ivakhnenkoi, ((Palaeagama.vielhaueri, Saurosternon.baini), (CHORISTODERA, (Paliguana.whitei, (Kuehneosaurus.latus, Pamelina.polonica), (Marmoretta.oxoniensis, (Sophineta.cracoviensis, (SQUAMATA, (Gephyrosaurus.bridensis, (Diphydontosaurus.avonis, (Planocephalosaurus.robinsonae, (Rebbanasaurus.jaini, ((Godavarisaurus.lateefi, Sphenocondor.gracilis), ((Brachyrhinodon.taylori, ((Clevosaurus.petilius, Clevosaurus.hudsoni), OTHER.CLEVOSAURUS)), OTHER.RHYNCHOCEPHALIA))))))))), (Odontochelys.semitestacea, (Proganochelys.quenstedti, (Palaeochersis.talampayensis, Australochelys.africanus, (OTHER.CHELONIA, (MEGAPLEURODIRA, Proterochersis.robusta))))), ((Eusaurosphargis.dalsassoi, Helveticosaurus.zollingeri), ((Paraplacodus.broilii, (Placodus.spp, ((Henodus.chelyops, (Cyamodus.kuhnschnyderi, Cyamodus.rostratus)), (Macroplacus.raeticus, (Protenodontosaurus.italicus, (Psephoderma.alpinum, Placochelys.placodonta)))))), (((Serpianosaurus.mirigiolensis, Neusticosaurus.pusillus, (Anarosaurus.spp, (Keichousaurus.spp, Dactylosaurus.gracilis))), (Simosaurus.gaillardoti, (Germanosaurus.schafferi, (Nothosaurus.mirabilis, ((Lariosaurus.balsami, Lariosaurus.curionii), (Lariosaurus.buzzi, (Lariosaurus.valceresii, (Lariosaurus.calcagnii, Lariosaurus.xingyiensis)))))))), (Cymatosaurus.spp, Corosaurus.alcovensis, (Yunguisaurus.liae, (Pistosaurus.longaevus, (Bobosaurus.forojuliensis, (Augustasaurus.hangdorni, ((Plesiopterys.wildi, Thalassiodracon.hawkinsi, PLESIOSAUROIDEA), RHOMALEOSAURIDAE)))))))), (((Endennesaurus.acutirostris, ((Anshunsaurus.wushaensis, Anshunsaurus.huangguoshuensis), (Miodentosaurus.brevis, Askeptosaurus.italicus))), ((Nectosaurus.halius, (Xinpusaurus.suni, Xinpusaurus.baomaolinensis)), (Agkistrognathus.campbelli, (Paralonectes.merriami, ((Thalattosaurus.borealis, Thalattosaurus.alexandrae), (Hescherleria.ruebeli, Clarazia.schinzi)))))), (Hupehsuchus.nanchangensis, (Utatsusaurus.hataii, (Xinminosaurus.catactes, Quasianosteosaurus.vikinghoegdai, Parvinatator.wapitiensis, (Chaohusaurus.geishanensis, Grippia.longirostris), ((Mixosaurus.maotaiensis, Wimanius.odontopalatus, ((Mixosaurus.panxianensis, (Mixosaurus.kuhnschnyderi, Mixosaurus.cornalianus)), (Contectopalatus.atavus, (Phalarodon.callawayi, (Phalarodon.major, Phalarodon.fraasi))))), ((Qianichthyosaurus.zhoui, Toretocnemus.spp), ((Cymbospondylus.buchseri, Phantomosaurus.neubigi, Cymbospondylus.petrinus),

(Besanosaurus.leptorhyncus, (Shastasaurus.spp, (Shonisaurus.popularis, (Mikadocephalus.gracilirostris, (Callawayia.spp, (Californosaurus.perrini, (Macgowania.janiceps, (Hudsonelpidia.brevirostris, (Temnodontosaurus.platyodon, (Temnodontosaurus.burgundiae, (Suevoleviathan.disinteger, (Platypterygius.spp, ((Leptonectes.spp, OTHER.LEPTONECTIDAE), (Ichthyosaurus.spp, OPTHALMOSAURIA))))))))))))))))))))), ((Prolacertoides.jimusarensis, Trilophosaurus.buettneri), (Mesosuchus.browni, (Howesia.browni, (Bentonyx.sidensis, Rhynchosaurus.brodiei, Stenaulorhynchus.stockleyi, Rhynchosaurus.articeps, (Fodonyx.spenceri, (Isalorhynchus.genovefae, Ammorhynchus.navajoi, (Teyumbaita.sulcognathus, (Hyperodapedon.huenei, (Hyperodapedon.mariensis, Hyperodapedon.sanjuanensis, (Hyperodapedon.huxleyi, Hyperodapedon.gordoni))))))))), (Prolacerta.broomi, (Protorosaurus.speneri, ((Vallesaurus.cenensis, (Hypuronector.limnaios, (Drepanosaurus.unguicaudatus, (Dolabrosaurus.aquatilis, Megalancosaurus.spp)))), (Macrocnemus.spp, (Langobardisaurus.spp, (Cosesaurus.aviceps, (Tanystropheus.meridensis, (Tanytrachelos.ahynis, Tanystropheus.longobardicus))))))), (Archosaurus.rossicus, Fugusuchus.hejiapensis, Proterosuchus.spp, Sarmatosuchus.otschevi, (Osmolskina.czatkowicensis, (Koilamasuchus.gonzalezdiazi, ((Shansisuchus.shansisuchus, (Vjushkovia.triplocostata, Erythrosuchus.africanus)), (Vancleavea.campi, Euparkeria.capensis, (Tarjadia.ruthae, (Archeopelta.arborensis, Doswellia.spp)), (Proterochampsa.bonapartei, (Cerritosaurus.binsfeldi, (Tropidosuchus.romeri, (Chanaresuchus.spp, Gualosuchus.reigi)))), (((Parasuchus.hislopi, ((Mystriosuchus.westphali, ((Redondasaurus.bermani, Redondasaurus.gregorii), (Pseudpopalatus.jablonskiae, (Pseudopalatus.mccauleyi, (Pseudopalatus.buceros, Pseudopalatus.pristinus))))), (Paleorhinus.scurriensis, (Paleorhinus.bransoni, (Paleorhinus.sawini, (Angistorhinus.spp, Brachysuchus.megalodon, (Protome.batalaria, Machaeroprosopus.zunii, Rutiodon.carolinensis, (Phytosaurus.doughtyi, (Leptosuchus.studeri, Leptosuchus.crosbiensis), (Pravusuchus.hortus, (Smilosuchus.lithodendrorum, (Smilosuchus.gregorii, Smilosuchus.adamanensis))))))))))), ((Revueltosaurus.spp, (Aetosaurus.spp, ((Aetosauroides.scaglia, Stagonolepis.robertsoni), (Stagonolepis.wellesi, (Coahomasuchus.kahleorum, (Typothorax.spp, Redondasuchus.spp)), (Neoaetosauroides.engaeus, ((Tecovasuchus.chatterjeei, (Heliocanthus.chamaensis, Paratypothorax.andressorum)), (Longosuchus.meadei, (Lucasuchus.hunti, (Acaenasuchus.geoffroyi, (Desmatosuchus.smalli, Desmatosuchus.haplocerus))))))))), (Ticinosuchus.ferox, (Riojasuchus.tenuisceps, Ornithosuchuslongidens), (Gracilisuchus.stipanicicorum, (Erpetosuchus.granti, (Dromichosuchus.grallator, Hesperosuchus.agilis, (Terrestrisuchus.gracilis, SPHENOSUCHIDAE, OTHER.CROCODYLIMORPHA)))), (Stagonosuchus.nyassicus, (Decuriasuchus.quartacolonia, Fasolasuchus.tenax, Saurosuchus.galilei, Prestosuchus.chiniquensis, Batrachotomus.kupferzellensis, (Postosuchus.alisonae, Tikisuchus.romeri, Polonosuchus.silesiacus, Teratosaurus.suevicus, Rauisuchus.tiradentes, Postosuchus.kirkpatricki))), (Diandongosuchus.fuyuanensis, (Qianosuchus.mixtus, (Lotosaurus.adentus, Poposaurus.gracilis, (Sillosuchus.longicervix, (Effigia.okeeffeae, Shuvosaurus.inexpectatus)), (Xilousuchus.sapingensis, Bromsgroveia.walkeri, Ctenosauriscus.koeneni, Hypselorhachis.mirabilis, Arizonasaurus.babbitti))))))), ((ANUROGNATHIDAE, (Sordes.pilosus, (Scaphognathus.crassirostris, Dorygnathus.bathensis, (Dimorphodon.macronyx, OTHER.BREVIQUARTOSSA, (Campylognathoides.liasicus, (Carniadactylus.rosenfeldi, Peteinosaurus.zambelli), (Austriadactylus.cristatus, Eudimorphodon.ranzii)))))), (Scleromochlus.taylori, ((Lagerpeton.chanarenis, (Dromomeron.gregorii, Dromomeron.romeri)), (Marasuchus.lilloensis, (Pseudolagosuchus.major, ((Lewisuchus.admixtus, (Asilisaurus.kongwe, (Eucoelophysis.baldwini, (Sacisaurus.agudoensis,

Silesaurus.opolensis)))), ((Pisanosaurus.mertii, GENASAURIA), ((Eoraptor.lunensis, (Chindesaurus.bryansmalli, (Staurikosaurus.pricei, Herrerasaurus.ischigualastensis)), (Eodromaeus.murphi, (Cryolophosaurus.ellioti, Tawa.hallae, COELUROSAURIA, CERATOSAURIA, Dilophosaurus.wetherilli, (Zupaysaurus.rougieri, Liliensternus.liliensterni, (Megapnosaurus.kayentakatae, (Coelophysis.bauri, Coelophysis.rhodesiensis)))))), (Panphagia.protos, Guaibasaurus.candelariensis, Agnosphitys.cromhallensis, (Chromogisaurus.novasi, Saturnalia.tupiniquim), ((Thecodontosaurus.antiquus, Pantydraco.caducus), (Efraasia.minor, Plateosauruavus.cullingworthi, Ruehleia.bedheimensis, (((Riojasaurus.incertus, Eucnemesaurus.fortis), ((Coloradisaurus.brevis, Massospondylus.spp, LUFENGOSAURINAE), (Unaysaurus.tolentinoi, (Plateosaurus.gracilis, (Plateosaurus.ingens, Plateosaurus.engelhardti))))), (Anchisaurus.polyzelus, (Euskelosaurus.browni, (Melanorosaurus.spp, (Antetonitrus.ingenipes, Isanosaurus.attavipachi, Kotasaurus.yamanpalliensis, Blikanasaurus.cromptoni, GRAVISAURIA)))))))))))))))))))))))))))))))))))));
end;

Supplementary Data 4

Supertree expanded by the replacement of higher taxa with their constituent species, in nexus format

#NEXUS
begin trees;
tree tree1 = [&r] ((Limnoscelis.paludis, (Tseajaia.campi, (Ambedus.pusillus, (Oradectes.sanmiguelensis, (Orobates.pabsti, (Sepeliodon.hesperis, (Silvadectes.absitus, (Diadectes.tenuitectes, Diasparactus.zenos, Diadectes.sideropelicus)))))))), (((Archaeothyris.florensis, (Varanosaurus.acutirostris, (Stereophallodon.ciscoensis, Ophiacodon.mirus))), ((Oedaleops.campi, Eothyris.parkeyi), (Oromycter.dolesorum, (Casea.broilii, (Trichasaurus.texensis, (Euromycter.rutenus, (Ennatosaurus.tecton, (Angelosaurus.dolani, Angelosaurus.romeri, Cotylorhynchus.romeri, (Cotylorhynchus.bransoni, Cotylrohynchus.hancocki)))))))), (Archaeovenator.hamiltonensis, (Pyozia.mesenensis, Apsisaurus.witteri, ((Mycterosaurus.longiceps, Heleosaurus.scholtzi, Mesenosaurus.romeri), (Elliotsmithia.longiceps, ((Ruthiromia.elcobriensis, (Aerosaurus.greenleeorum, Aerosaurus.wellesi)), (Varanops.brevirostris, (Varanodon.agilis, Watongia.meiri))))))), ((Ianthasaurus.hardestii, (Glaucosaurus.megalops, Lupeosaurus.kayi, (Edaphosaurus.novomexicanus, (Edaphosaurus.colohistion, (Edaphosaurus.boanerges, (Edaphosaurus.pogonias, Edaphosaurus.cruciger)))))), (Haptodus.garnettensis, (Palaeohatteria.longicaudata, (Ianthodon.schultzei, (Pantelosaurus.saxonicus, (Cutleria.wilmarthi, ((Secodontosaurus.obtusidens, (Cryptovenator.hirschbergeri, ((Ctenospondylus.casei, Ctenospondylus.ninevehensis), (Sphenacodon.ferox, Sphenacodon.ferocior), Dimetrodon.milleri, Dimetrodon.limbatus))), (Tetraceratops.insignis, (Raranimus.dashankouensis, (((Biarmosuchus.tener, (Hipposaurus.boonstrai, (Herpetoskylax.hopsoni, (Lycaenodon.longiceps, (Ictidorhinus.martinsi, (Lophorhinus.willodensis, Lobalopex.mordax, Lemurosaurus.pricei, ((Paraburnetia.sneeubergensis, Proburnetia.viatkensis), (Niuksenitia.sikbonensis, Pachydectes.elsi, Bullacephalus.jacksoni, Burnetia.mirabilis)))))))), (Aloposaurus.tenuis, (Cyonosaurus.longiceps, ((Aelurosaurus.felinus, Aelurosaurus.polyodon, Aelurosaurus.whaitsi, Aelurosaurus.wilmani), ((Sauroctonus.parringtoni, Sauroctonus.progressus), (Scylacognathus.spp, (Gorgonops.torvus, (Galesuchus.gracilis, ((Njalila.nasuta, Njalila.insigna), ((Lycaenops.ornatus, Lycaenops.angusticeps, Lycaenops.microdon, Lycaenops.sollasi), (Arctognathus.curvimola, (Inostrancevia.spp, ((Aelurognathus.trigriceps, Aelurognathus.broodiei, Aelurognathus.ferox, Aelurognathus.alticeps, Aelurognathus.kingwilli, Aelurognathus.maccabei), (Rubidgea.atrox, (Clelandina.spp, (Sycosaurus.laticeps, Sycosaurus.kingoriensis))))))))))))))))), (((Estemmenosuchus.mirabilis, Ulemosaurus.svijagensis, Tapinocaninus.pamelae, Estemmenosuchus.uralensis, Jonkeria.truculenta, Styracocephalus.platyrhynchus), (Archaeosyodon.praeventor, Microsyodon.orlovi, (Sinophoneus.yumenensis, (Titanophoneus.adamanteus, (Anteosaurus.magnificus, Titanophoneus.potens)), (Pampaphoneus.biccai, (Notosyodon.gusevi, (Australosyodon.nyaphuli, Syodon.biarmicum)))))), ((Lycosuchus.vanderrieti, ((Pristerognathus.polyodon, Ictidosaurus.angusticeps, Glanosuchus.macrops), (((Moschorhinus.kitchingi, Cerdops.burgheri), (Olivierosuchus.parringtoni, Promoschorhynchus.platyrhinus), (Bauria.cynops, Ericiolacerta.parva, Scaloposaurus.constrictus, Regisaurus.jacobi, Ichtidosuchoides.longiceps, Ictidosuchops.rubidgei, Ictidosuchus.primaevus, (Lycideops.longiceps, (Choerosaurus.dejageri, (Tetracynodon.darti, Tetracynodon.tenuis))))), ((Viatkosuchus.sumini, (Theriognathus.microps, Moschowhaitsia.vjuschkovi)), (Bolotridon.frerensis, Cynosaurus.longiceps, Charassognathus.gracilis,

Procynosuchus.delaharpeae, Dvinia.prima, (Progalesaurus.lootbergensis, Galesaurus.planiceps), ((Nanictosaurus.rubidgei, Thrinaxodon.liorhinus), (Platycraniellus.elegans, ((Cynognathus.spp, (Diademodon.tetragonus, ((Trirachodon.spp, (Langbergia.modisei, Cricodon.metabolus)), ((Pascualgnathus.polanskii, Andescynodon.mendozensis), (Scalenodon.angustifrons, (Luangwa.drysdalli, Luangwa.sudamericana), Scalenodon.hirschsoni, Dadadon.isaloi, Santacruzodon.hopsoni, (Massetognathus.ochagaviae, Massetognathus.pascuali), (Nanogomphodon.wildi, Arctotraversodon.plemmyridon, Boreogomphodon.jeffersoni), (Gomphodontosuchus.brasiliensis, (Scalenodontoides.macrodontes, Menadon.besairiei, (Exaeretodon.frenguellii, Exaeretodon.argentinus, Exaeretodon.riograndensis)))))))), (Lumkuia.fuzzi, (Trucidocynodon.riograndensis, Ecteninion.lunensis), (((Probelesodon.lewisi, Probelesodon.sanjuanensis), (Aleodon.brachyrhamphus, Chiniquodon.spp)), (Probainognathus.jenseni, ((Kayentatherium.wellesi, (Oligokyphus.triserialis, Oligokyphus.major, Oligokyphus.lufengensis)), (Therioherpeton.carnegini, Prozostrodon.brasiliensis, Brasilodon.quadrangularis, Brasilitherium.riograndensis, (Riograndia.guaibensis, (Chaliminia.musteloides, Elliotherium.kersteni, (Irajatherium.hernandezi, OTHER.TRITHELODONTIDAE))), (Sinoconodon.rigneyi, (Adelobasileus.cromptoni, (Megazostrodon.rudnerae, ((Kuehneotherium.praecursoris, Dinnetherium.nezorum), (Morganucodon.watsoni, OTHER.MAMMALIA)))))))))))))))))), (Biseridens.qilianicus, ((Anomocephalus.africanus, Tiarajudens.eccentricus), (Patranomodon.nyaphulii, ((Galepus.jouberti, Galechirus.scholtzi), (Otsheria.netzvetajevi, ((Ulemica.invisa, Ulemica.efremovi), Suminia.getmanovi)), (Galeops.whaitsi, (Eodicynodon.oelofseni, (Eodicynodon.oosthuizeni, (Colobodectes.cluveri, ((Eosimops.newtoni, Robertia.broomiana, Diictodon.feliceps, Prosictodon.dubei), ((Pristerodon.mackayi, (Lanthanostegus.mohoii, (Endothiodon.spp, Chelydontops.altidentalis))), (((Emydops.oweni, Emydops.arctatus), ((Dicynodontoides.spp, (Kombuisia.antarctica, Kombuisia.frerensis)), (Myosaurus.gracilis, (Cistecephalus.microrhinus, (Kawingasaurus.fossilis, Cistecephaloides.boonstrai))))), ((Keyseria.benjamini, (Daqingshanodon.limbus, ((Rhachiocephalus.magnus, Kitchinganomodon.crasus), ((Oudenodon.bainii, ((Australobarbarus.kotelnitschi, Australobarbarus.platycephalus), Tropidostoma.microtrema)), (Odontocyclops.whaitsi, Idelesaurus.tataricus, (Aulacephalodon.bainii, (Geikia.elginensis, Geikia.locusticeps))))))), ((Elph.borealis, Interpresosaurus.blomi), (Katumbia.parringtoni, (Gorgonia.traquairi, (Delectosaurus.arefjevi, ((Dicynodon.huenei, Dicynodon.lacerticeps), ((Vivaxosaurus.trautscholdi, (Daptocephalus.leoniceps, (Dinanomodon.gilli, Peramodon.amalitzkii))), ((Jimusaria.sinkianensis, (Syops.vanhoepeni, ((Euptychognathus.bathyrhynchus, (Basilodon.woodwardi, Sintocephalus.alticeps)), ((Lystrosaurus.platyceps, Lystrosaurus.georgi, Lystrosaurus.curvatus), (Kwazulusaurus.shakai, (Lystrosaurus.broomi, Lystrosaurus.hedini, (Lystrosaurus.shuchanggouensis, Lystrosaurus.robustus)), (Lystrosaurus.oviceps, (Lystrosaurus.maccaigi, Lystrosaurus.declivis, Lystrosaurus.murrayi))))))), (Turfanodon.bogdaensis, Dinanomodon.rubidgei, ((Vinceria.argentinensis, (Rhinodicynodon.gracile, Tetragonias.njalilus, Shansiodon.spp)), ((Rabidosaurus.cristatus, (Kannemeyeria.lophorhina, Kannemeyeria.simocephala)), (Wadiasaurus.indicus, (((Moghreberia.nmachouensis, (Placerias.gigas, Placerias.hesternus)), (Angonisaurus.cruickshanki, Ischigualastia.jenseni, Jachaleria.spp, Stahleckeria.potens)), ((Sangusaurus.edentatus, Rhadiodromus.klimovi), ((Dinodontosaurus.pedroanum, (Rechnisaurus.cristarhynchus, Dolichuranus.spp)), ((Sinokannemeyeria.pearsoni, (Sinokannemeyeria.sanchuanheensi, Sinokannemeyeria.yingchiaoensis)), (Parakannemeyeria.ningwuensis, (Parakannemeryeria.youngi, (Parakannemeyeria.shenmuensis,

Parakannemeyeria.dolichocephala))),
(((Brazilosaurus.sanpauloensis, Stereosternum.temidum, Mesosaurus.pleurogaster),
(((Eunotosaurus.africanus, Milleretta.rubidgei), (Broomia.perplexa, (Millerosaurus.nuffieldi,
(Millerosaurus.ornatus, Milleropsis.pricei)))), (Microleter.mckinzieorum,
(Australothyris.smithae, ((Feeserpeton.oklahomensis, (Lanthanosuchus.watsoni,
(Colobomycter.pholeter, Acleistorhinus.pteroticus))), ((Eudibamus.cursoris,
((Bolosaurus.grandis, Bolosaurus.striatus), (Belebey.chengi, Belebey.vegrandis,
Belebey.maximi))), (((Nyctiphruretus.acudens, Nyctiphruretus.optabilis),
((Tokosaurus.perforatus, Macroleter.poezicus), (Bashkyroleter.mesensis,
Bashkyroleter.bashkyricus, Nycteroleter.spp, Emeroleter.levis, Rhipaeosaurus.spp))),
((Bradysaurus.seeleyi, (Bradysaurus.baini, Nochelesaurus.schwarzi,
(Embrithosaurus.schwarzi, (Deltavjatia.mesensis, ((Provelosaurus.americanus,
(Pumiliopareia.pricei, Anthodon.serrarius)), ((Shansisaurus.xuecunensis,
(Shihtienfenia.permica, (Pareiasuchus.peringueyi, Pareiasuchus.nasicornis))),
(Pareiasaurus.serridens, (Pareiasaurus.geinitzi, ((Sanchuansaurus, Scutosaurus.kapinskii),
(Elginia.mriabilis, Arganaceras.vacanti)))))))))), ((Owenetta.rubidgei,
Saurodectes.rogersorum, Barasaurus.besairiei, (Candelaria.barbouri,
Owenetta.kitchingorum)), (Coletta.seca, ((Kitchingnathus.utabeni, Sauropareion.anoplus),
(Pintosaurus.magnidentes, (Phaanthosaurus.simus, ((Theledectes.perforatus,
Eumetabolodan.dongshengensis), (Tichvinskia.vjatkensis, ((Timanophon.raridentatus,
Anomoiodon.liliensterni, Thelophon.contritus, (Kapes.amaenus, Kapes.komiensis,
Kapes.majmesculae, Kapes.bentoni)), (Eumetabolodan.bathycephalus,
(Teratophon.sprinigensis, Procolophon.trigoniceps, Thelerpeton.opressus)),
(Pentaedrusaurus.ordosianus, (Phonodus.dutoitorum, Neoprocolophon.asiaticus,
((Scoloparia.glyphanodon, Sclerosaurus.armatus), (Leptopleuron.lacertinum,
(Soturnia.caliodon, Hypsognathus.fenneri))))))))))))))))))))), (Coelostegus.prothales,
((Thuringothyris.mahlendorffae, (Concordia.cunninghami, ((Romeria.prima,
Romeria.texana), (Protocaptorhinus.pricei, (Reiszorhinus.olson, (Rhiodenticulatus.heatoni,
(Saurorictus.australis, ((Captorhinus.laticeps, (Captorhinus.aguti, Captorhinus.magnus)),
(Captorhinikos.spp, (Labidosaurus.hamatus, (Labidosaurikos.meachami,
(Moradisaurus.grandis, (Gansurhinus.qingtoushanensis, Rothianiscus.spp))))))))))))),
(Brouffia.orientalis, (Paleothyris.acadiana, ((Spinoaequalis.schultzei,
(Petrolacosaurus.kansensis, Araeoscelis.spp)), (Claudiosaurus.germaini, Coelurosauravus.spp,
Longisquama.insignis, ((Acerosodontosaurus.piveteaui, (Tangasaurus.mennelli,
Youngina.capensis)), (Lanthanolania.ivakhnenkoi, ((Palaeagama.vielhaueri,
Saurosternon.baini), (CHORISTODERA, (Paliguana.whitei, (Kuehneosaurus.latus,
Pamelina.polonica), (Marmoretta.oxoniensis, (Sophineta.cracoviensis, (SQUAMATA,
(Gephyrosaurus.bridensis, (Diphydontosaurus.avonis, (Planocephalosaurus.robinsonae,
(Rebbanasaurus.jaini, ((Godavarisaurus.lateefi, Sphenocondor.gracilis),
((Brachyrhinodon.taylori, ((Clevosaurus.petilius, Clevosaurus.hudsoni),
OTHER.CLEVOSAURUS)), OTHER.RHYNCHOCEPHALIA))))))))),
(Odontochelys.semitestacea, (Proganochelys.quenstedti, (Palaeochersis.talampayensis,
Australochelys.africanus, (OTHER.CHELONIA, (MEGAPLEURODIRA,
Proterochersis.robusta))))), ((Eusaurosphargis.dalsassoi, Helveticosaurus.zollingeri),
((Paraplacodus.broilii, ((Placodus.gigas, Placodus.inexpectatus), ((Henodus.chelyops,
(Cyamodus.kuhnschnyderi, Cyamodus.rostratus)), (Macroplacus.raeticus,
(Protenodontosaurus.italicus, (Psephoderma.alpinum, Placochelys.placodonta)))))),
(((Serpianosaurus.mirigiolensis, Neusticosaurus.pusillus, ((Anarosaurus.pumilio,
Anarosaurus.heterodontus), (Keichousaurus.spp, Dactylosaurus.gracilis))),
(Simosaurus.gaillardoti, (Germanosaurus.schafferi, (Nothosaurus.mirabilis,
((Lariosaurus.balsami, Lariosaurus.curionii), (Lariosaurus.buzzi, (Lariosaurus.valceresii,

(Lariosaurus.calcagnii, Lariosaurus.xingyiensis)))))))), ((Cymatosaurus.fridericianus, Cymatosaurus.minor, Cymatosaurus.erikae), Corosaurus.alcovensis, (Yunguisaurus.liae, (Pistosaurus.longaevus, (Bobosaurus.forojuliensis, (Augustasaurus.hangdorni, ((Plesiopterys.wildi, Thalassiodracon.hawkinsi, PLESIOSAUROIDEA), RHOMALEOSAURIDAE))))))))), (((Endennesaurus.acutirostris, ((Anshunsaurus.wushaensis, Anshunsaurus.huangguoshuensis), (Miodentosaurus.brevis, Askeptosaurus.italicus))), ((Nectosaurus.halius, (Xinpusaurus.suni, Xinpusaurus.baomaolinensis)), (Agkistrognathus.campbelli, (Paralonectes.merriami, ((Thalattosaurus.borealis, Thalattosaurus.alexandrae), (Hescherleria.ruebeli, Clarazia.schinzi)))))), (Hupehsuchus.nanchangensis, (Utatsusaurus.hataii, (Xinminosaurus.catactes, Quasianosteosaurus.vikinghoegdai, Parvinatator.wapitiensis, (Chaohusaurus.geishanensis, Grippia.longirostris), ((Mixosaurus.maotaiensis, Wimanius.odontopalatus, ((Mixosaurus.panxianensis, (Mixosaurus.kuhnschnyderi, Mixosaurus.cornalianus)), (Contectopalatus.atavus, (Phalarodon.callawayi, (Phalarodon.major, Phalarodon.fraasi))))), ((Qianichthyosaurus.zhoui, (Toretocnemus.zitelli, Toretocnemus.californicus)), ((Cymbospondylus.buchseri, Phantomosaurus.neubigi, Cymbospondylus.petrinus), (Besanosaurus.leptorhyncus, (Shastasaurus.spp, (Shonisaurus.popularis, (Mikadocephalus.gracilirostris, ((Callawayia.neoscapularis, Callawayia.altispinus), (Californosaurus.perrini, (Macgowania.janiceps, (Hudsonelpidia.brevirostris, (Temnodontosaurus.platyodon, (Temnodontosaurus.burgundiae, (Suevoleviathan.disinteger, ((Platypterygius.platypterygius, Platypterygius.scahicarum, Platypterygius.hercynicus, Platypterygius.ochevi), (((Leptonectes.moorei, Leptonectes.solei, Leptonectes.tenuirostris), OTHER.LEPTONECTIDAE), (Ichthyosaurus.spp, OPTHALMOSAURIA)))))))))))))))))))))), ((Prolacertoides.jimusarensis, Trilophosaurus.buettneri), (Mesosuchus.browni, (Howesia.browni, (Bentonyx.sidensis, Rhynchosaurus.brodiei, Stenaulorhynchus.stockleyi, Rhynchosaurus.articeps, (Fodonyx.spenceri, (Isalorhynchus.genovefae, Ammorhynchus.navajoi, (Teyumbaita.sulcognathus, (Hyperodapedon.huenei, (Hyperodapedon.mariensis, Hyperodapedon.sanjuanensis, (Hyperodapedon.huxleyi, Hyperodapedon.gordoni))))))))), (Prolacerta.broomi, (Protorosaurus.speneri, ((Vallesaurus.cenensis, (Hypuronector.limnaios, (Drepanosaurus.unguicaudatus, (Dolabrosaurus.aquatilis, (Megalancosaurus.preonensis, Megalancosaurus.endennae))))), ((Macrocnemus.basannii, Macrocnemus.fuyanensis), ((Langobardisaurus.rossii, Langobardisaurus.pandolfii, Langobarisaurus.tonelloi), (Cosesaurus.aviceps, (Tanystropheus.meridensis, (Tanytrachelos.ahynis, Tanystropheus.longobardicus))))))), (Archosaurus.rossicus, Fugusuchus.hejiapensis, Proterosuchus.fergusi, Sarmatosuchus.otschevi, (Osmolskina.czatkowicensis, (Koilamasuchus.gonzalezdiazi, ((Shansisuchus.shansisuchus, (Vjushkovia.triplocostata, Erythrosuchus.africanus)), (Vancleavea.campi, Euparkeria.capensis, (Tarjadia.ruthae, (Archeopelta.arborensis, (Doswellia.kaltenbachi, Doswellia.sixmilensis))), (Proterochampsa.bonapartei, (Cerritosaurus.binsfeldi, (Tropidosuchus.romeri, (Chanaresuchus.bonapartei, Gualosuchus.reigi)))), (((Parasuchus.hislopi, ((Mystriosuchus.westphali, ((Redondasaurus.bermani, Redondasaurus.gregorii), (Pseudpopalatus.jablonskiae, (Pseudopalatus.mccauleyi, (Pseudopalatus.buceros, Pseudopalatus.pristinus))))), (Paleorhinus.scurriensis, (Paleorhinus.bransoni, (Paleorhinus.sawini, (Angistorhinus.spp, Brachysuchus.megalodon, (Protome.batalaria, Machaeroprosopus.zunii, Rutiodon.carolinensis, (Phytosaurus.doughtyi, (Leptosuchus.studeri, Leptosuchus.crosbiensis), (Pravusuchus.hortus, (Smilosuchus.lithodendrorum, (Smilosuchus.gregorii, Smilosuchus.adamanensis)))))))))))), (((Revueltosaurus.hunti, Revueltosaurus.callenderi, Revueltosaurus.olseni), ((Aetosaurus.arcuatus, Aetosaurus.crassicauda, Aetosaurus.ferratus), ((Aetosauroides.scaglia, Stagonolepis.robertsoni), (Stagonolepis.wellesi, (Coahomasuchus.kahleorum,

((Typothorax.coccinarum, Typothorax.antiquum), (Redondasuchus.reseri,
Redondasuchus.rineharti))), (Neoaetosauroides.engaeus, ((Tecovasuchus.chatterjeei,
(Heliocanthus.chamaensis, Paratypothorax.andressorum)), (Longosuchus.meadei,
(Lucasuchus.hunti, (Acaenasuchus.geoffroyi, (Desmatosuchus.smalli,
Desmatosuchus.haplocerus)))))))))), (Ticinosuchus.ferox, (Riojasuchus.tenuisceps,
Ornithosuchus.longidens), (Gracilisuchus.stipanicicorum, (Erpetosuchus.granti,
(Dromichosuchus.grallator, Hesperosuchus.agilis, (Terrestrisuchus.gracilis,
SPHENOSUCHIDAE, OTHER.CROCODYLIMORPHA)))), (Stagonosuchus.nyassicus,
(Decuriasuchus.quartacolonia, Fasolasuchus.tenax, Saurosuchus.galilei,
Prestosuchus.chiniquensis, Batrachotomus.kupferzellensis, (Postosuchus.alisonae,
Tikisuchus.romeri, Polonosuchus.silesiacus, Teratosaurus.suevicus, Rauisuchus.tiradentes,
Postosuchus.kirkpatricki))), (Diandongosuchus.fuyuanensis, (Qianosuchus.mixtus,
(Lotosaurus.adentus, Poposaurus.gracilis, (Sillosuchus.longicervix, (Effigia.okeeffeae,
Shuvosaurus.inexpectatus)), (Xilousuchus.sapingensis, Bromsgroveia.walkeri,
Ctenosauriscus.koeneni, Hypselorhachis.mirabilis, Arizonasaurus.babbitti))))))),
((ANUROGNATHIDAE, (Sordes.pilosus, (Scaphognathus.crassirostris,
Dorygnathus.bathensis, (Dimorphodon.macronyx, OTHER.BREVIQUARTOSSA,
(Campylognathoides.liasicus, (Carniadactylus.rosenfeldi, Peteinosaurus.zambelli),
(Austriadactylus.cristatus, Eudimorphodon.ranzii)))))), (Scleromochlus.taylori,
((Lagerpeton.chanarenis, (Dromomeron.gregorii, Dromomeron.romeri)),
(Marasuchus.lilloensis, (Pseudolagosuchus.major, ((Lewisuchus.admixtus,
(Asilisaurus.kongwe, (Eucoelophysis.baldwini, (Sacisaurus.agudoensis,
Silesaurus.opolensis)))), ((Pisanosaurus.mertii, GENASAURIA), ((Eoraptor.lunensis,
(Chindesaurus.bryansmalli, (Staurikosaurus.pricei, Herrerasaurus.ischigualastensis)),
(Eodromaeus.murphi, (Cryolophosaurus.ellioti, Tawa.hallae, COELUROSAURIA,
CERATOSAURIA, Dilophosaurus.wetherilli, (Zupaysaurus.rougieri,
Liliensternus.liliensterni, (Megapnosaurus.kayentakatae, (Coelophysis.bauri,
Coelophysis.rhodesiensis)))))), (Panphagia.protos, Guaibasaurus.candelariensis,
Agnosphitys.cromhallensis, (Chromogisaurus.novasi, Saturnalia.tupiniquim),
((Thecodontosaurus.antiquus, Pantydraco.caducus), (Efraasia.minor,
Plateosauruavus.cullingworthi, Ruehleia.bedheimensis, (((Riojasaurus.incertus,
Eucnemesaurus.fortis), ((Coloradisaurus.brevis, (Massospondylus.carinatus,
Massospondylus.kaalae), LUFENGOSAURINAE), (Unaysaurus.tolentinoi,
(Plateosaurus.gracilis, (Plateosaurus.ingens, Plateosaurus.engelhardti))))),
(Anchisaurus.polyzelus, (Euskelosaurus.browni, ((Melanorosaurus.readi,
Melanorosaurus.thabanensis), (Antetonitrus.ingenipes, Isanosaurus.attavipachi,
Kotasaurus.yamanpalliensis, Blikanasaurus.cromptoni,
GRAVISAURIA)))))))))))))))))))))))))))))))))))));
end;

Supplementary Data 5

R function for calculating sampling corrected origination rates. Text within “#” represent notes on what the previous lines do (these do not need to be deleted as they will not be read by R)

```
library(paleotree)

ori.rate<-function(tree,ages,time,tc.method)

###########################################################################
#"tree" is the phylogeny, not time calibrated. "Ages" is a matrix of first and last appearences
#of each taxon in the tree. "time" is a matrix of beginning and end dates of each time bin.
#"tc.method" is the time calibration method being used (options as in timePaleoPhy)
###########################################################################

{
	time.tree<-timePaleoPhy(tree,ages,method=tc.method,add.term=T,inc.term.adj=F)
##################
#time calibrate the tree
###################

	ghost.tree<-timePaleoPhy(tree,ages,method=equal,add.term=T,inc.term.adj=F)
#####################################################################
#time calibrate the tree, but do not include termianl ranges of the tip taxa (this is a #phylogeny
only including the ghost lineages)
#####################################################################

	ghosts<- phyloDiv(ghost.tree,int.times=time,plot=F,drop.zlb=T)
#####################################################################
#find number of ghost linneages in each time bin
	#####################################################################

PDE<- phyloDiv(time.tree,int.times=time,plot=F)
########################################################
#find total number of linneages (observed and ghost) in each time bin
	########################################################

sampling<- ghosts/PDE
########################
#Calculate sampling estimate
	########################

node.dates<-dateNodes(time.tree)
####################
#date origination events
	####################

int.node.dates<-node.dates[length(time.tree$tip.label)+1:length(node.dates)]
###############
# delete tip nodes
```

```
##############

ori.count<-vector(length=nrow(time),mode="numeric")
##############################################################
#create empty vector to store number of origination events in each time bin
	##############################################################

for(i in 1:lenth(ori.count))
	{
		for(j in 1:length(int.node.dates))
		{
			if(int.node.dates[j]<=time[i,1] && int.node.dates[j]>=time[i,2])
			{
				ori.count[i]<-ori.count[i]+1
			}
		}
	}
	##############################################
	#Count number of origination events in each time bin
	##############################################

	perlin.ori<-ori.count/PDE
########################
# per linneage origination rate
########################

	samp.corr.ori<-perlin.ori[2:length(perlin.ori)]*sampling[1:length(sampling)-1]
############################
#multiply by sampling probability
############################

	return(samp.corr.ori)
}
```

Supplementary Data 6

R function for calculating sampling corrected extinction rates. Text within "#" represent notes on what the previous lines do (these do not need to be deleted as they will not be read by R)

```
library(paleotree)

ext.rate<-function(tree,ages,time,tc.method)

################################################################################
#"tree" is the phylogeny, not time calibrated. "Ages" is a matrix of first and last appearences
#of each taxon in the tree. "time" is a matrix of beginning and end dates of each time bin.
#"tc.method" is the time calibration method being used (options as in timePaleoPhy)
################################################################################

{
	time.tree<-timePaleoPhy(tree,ages,method=tc.method,add.term=T,inc.term.adj=F)
###################
#time calibrate the tree
####################

	ghost.tree<-timePaleoPhy(tree,ages,method=equal,add.term=T,inc.term.adj=F)
#########################################################################
#time calibrate the tree, but do not include termianl ranges of the tip taxa (this is a #phylogeny only including the ghost lineages)
#########################################################################

	ghosts<- phyloDiv(ghost.tree,int.times=time,plot=F,drop.zlb=T)
#########################################################################
#find number of ghost linneages in each time bin
	#########################################################################

PDE<- phyloDiv(time.tree,int.times=time,plot=F)
############################################################
#find total number of linneages (observed and ghost) in each time bin
	############################################################

sampling<- ghosts/PDE
#########################
#Calculate sampling estimate
	#########################

ext.count<-vector(length=nrow(time),mode="numeric")
################################################################
#create empty vector to store number of origination events in each time bin
	################################################################

for(i in 1:lenth(ext.count))
{
		for(j in 1:nrow(ages))
		{
```

```
					if(ages[j,2]<=time[i,1] && ages[j,2]>=time[i,2])
					{
						ext.count[i]<-ext.count[i]+1
					}
				}
		}
############################################
		#Count number of extinction events in each time bin
		############################################

		perlin.ext<-ext.count/PDE
########################
# per linneage origination rate
########################

		samp.corr.ext<-perlin.ext[1:length(perlin.ext)-1]*sampling[2:length(sampling)]
############################
#multiply by sampling probability
############################

		return(samp.corr.ori)
}
```
